# Global, regional, and national trends in DALYs for blindness and vision loss in teenagers and young adults, 1990–2019: an age-period-cohort analysis based on the Global Burden of Disease Study 2019

**DOI:** 10.3389/fmed.2025.1624618

**Published:** 2025-10-29

**Authors:** Juan Lu, Xiaoyun Jiang, Hao Yu, Yanghua Tian, Liming Tao

**Affiliations:** ^1^Department of Ophthalmology, The Second Hospital of Anhui Medical University Institution, Hefei, Anhui, China; ^2^Hefei Cancer Hospital, Chinese Academy of Sciences, Hefei, Anhui, China; ^3^School of Mental Health and Psychological Sciences, Anhui Medical University, Hefei, Anhui, China

**Keywords:** blindness and vision loss, disability-adjusted life years (DALYs), age-period-cohort, Global Burden of Disease Study, socio-demographic index

## Abstract

**Purpose:**

To analyze temporal trends in disability-adjusted life years (DALYs) associated with blindness and vision loss in teenagers and young adults at global, regional, and national levels between 1990 and 2019, with particular attention to age, period, and birth cohort.

**Methods:**

Estimates were derived from the Global Burden of Disease Study 2019. An age-period-cohort (APC) model was adopted to estimate overall annual percentage changes in DALYs (net drift), annual percentage changes in DALYs within age groups (local drift), fitted longitudinal age-specific rates adjusted for period deviations (age effects), and period- and birth cohort-relative risks (period/cohort effects) between 1990 and 2019. This facilitated the identification of disparities and treatment gaps in eye care.

**Results:**

In 2019, the global number of DALYs associated with blindness and vision loss in teenagers and young adults was 2085.40 thousand (95% UI: 1340.56 to 3074.23). Among the 204 countries and territories, 40 had DALYs of at least 10,000, with the top five countries (India, China, Brazil, Indonesia, and Pakistan) being responsible for 48% of DALYs globally. The APC model estimated a global net drift at −0.43% (95% CI: −0.45% to −0.42%) per year, ranging from −0.92% (95% CI: −0.94% to −0.91%) in low-middle socio-demographic Index (SDI) regions to −0.03% (95% CI: −0.07% to −0.0005%) in high SDI regions. As reflected by local drift, the DALYs had decreasing trends across all age groups, except in high SDI countries. Age effects illustrated similar patterns across different SDI regions, with risk increasing with age. High SDI region showed generally higher period risks over time, whereas others showed more favorable period risks. High SDI regions presented worsening risks of DALYs, while other regions indicated improving trends of DALYs in successive birth cohorts.

**Conclusion:**

Although an overall favorable trend of DALYs related to blindness and vision loss in teenagers and young adults was observed globally over the past three decades, unfavorable period and cohort effects were found in many countries, raising questions about the adequacy of their eye care across all age groups. Improvements in prevention, treatment, and rehabilitation programs related to blindness and vision loss in teenagers and young adults could reduce the risk for successively younger birth cohorts and for all age groups over time.

## Introduction

Vision impairment is a major public health problem and can lead to a reduced quality of life. In 2020, almost 2.2 billion people around the world were reported to have vision impairment, and nearly 43.3 million people were estimated to be blind (1–3). Globally, cataract and refractive error are major causes of vision impairment, resulting in substantial losses for individuals and society (3).

Although the prevalence of vision impairment increases with age, estimates of vision impairment should not be ignored in young people (4–6). Since many eye diseases are irreversible and progressive, vision impairment in teenagers and young adults may have lifelong effects (7, 8). The severity of visual impairment in younger people is associated with the deterioration of physical development, mental and physical health, personal identity and socialization, which in turn poses significant challenges in many areas of life, such as educational attainment, employment and interpersonal communication (9–13). In this manuscript, we select 15–39 age group as the research subject, because of this age group represents the working-age population, where vision loss can significantly impact productivity, economic participation, and mental health. And most vision studies focus on children (congenital blindness) or older adults (age-related diseases like cataracts, AMD), leaving the 15–39 age group underrepresented in research. Therefore, studying the 15–39 age group helps uncover unique disease patterns, highlights the long-term socioeconomic consequences of vision loss, and supports tailored prevention and treatment strategies.

The Global Burden of Diseases, Injuries, and Risk Factors Study (GBD) has employed disability-adjusted life-years (DALYs) as a composite metric that assesses the disease burden by comparing the current status with an ideal condition in which people live to the age of standard life expectancy in perfect health (3, 14). DALYs is a metric that incorporates years of life lost due to premature death (YLL), as well as years lived with disability (YLD), associated with an illness. One DALY represents 1 year of health life lost (15). A thorough analysis of time trends in blindness and vision loss associated DALYs for all countries and regions is needed to provide information into elucidating epidemiology, tracking disease progression and identifying the priorities of further intervention. DALY risks can be categorized into age, period, and birth cohort effects. It has been reported that the number of visual impairment-associated DALYs increases with advancing age (16, 17). The risks of DALYs from vision impairment not only vary by physical age but may also differ between birth cohorts due to the introduction of new diagnoses and treatments. Advances in medical technology, as well as the coverage and quality of health services related to the prevention and treatment of blindness and vision loss, can also affect all individuals (18), regardless of age and birth cohort over a period of time. Moreover, the risks of DALYs for blindness and vision loss in teenagers and young adults also differ among birth cohorts due to the accumulation of various life events. Thus, an in-depth analysis of global time trends in DALYs associated with blindness and vision loss, with particular attention to age, period, and birth cohort effects, is needed to elucidate our understanding of disease epidemiology, track disease progression, and identify priorities and potential gaps in prevention and intervention.

Age, period, and cohort are the three main intrinsic factors in vision impairment, but there is an absolute linear relationship between them, and the age-period-cohort model enhances our understanding of trends in disability-adjusted life years when adjusting for age, period, and birth cohort. However, the relative outcomes of age, period and birth cohort effects contributing to DALYs have not been thoroughly analyzed. In addition, many areas of the world, including high-, middle- and low-income countries, lack information on DALYs trends for blindness and vision loss in teenagers and young adults, as well as their associations with age, period and birth cohort effects. The Global Burden of Diseases, Injuries, and Risk Factors Study (GBD) 2019 is a cooperative research project that uses up-to-date epidemiological data and advanced statistical methods to generate population health metrics for analyzing temporal trends on a global scale. In this study, we extracted data from GBD 2019 and used age-period-cohort (APC) models to conduct a comprehensive analysis of changes in DALYs associated with blindness and vision loss in teenagers and young adults from 1990 to 2019 across 204 countries and territories.

## Materials and methods

### Data sources

We use the 2019 version of the GBD, a study that provides regularly updated estimates on health loss across 369 diseases, injuries, and impairments, as well as 87 risks by age and sex in 204 countries or territories (14). GBD 2019 compiled data from various sources through systematic data identification and extraction. Detailed information regarding data sources for estimating the burden of blindness and vision loss in different countries and regions around the world can be found in the GBD 2019 data input source tool^1^ (14). GBD 2019 complies with the Guidelines for Accurate and Transparent Health Estimates Reporting (GATHER) statement (19).

Global Burden of Diseases, Injuries, and Risk Factors Study provides multiple interrelated metrics to measure the burden of diseases, including incidence, prevalence, mortality, years-of-life-lost (YLLs), year-lived-with-disability (YLDs), and disability-adjusted life-years (DALYs). DALYs are equal to the sum of YLLs and YLDs, and they are the most commonly used health summary measure. For blindness and vision loss (non-fatal disease), DALYs are equivalent to YLDs. Blindness and vision loss were mapped to the GBD cause list with the following International Classification of Diseases and Injuries codes^2^ : disorders of sclera, cornea, iris and ciliary body (H15–H22.8), disorders of lens (H25–H28.8), disorders of choroid and retina (H31–H36.8), glaucoma (H40–H40.9, H42–H42.8), disorders of optic nerve and visual pathways (H46–H48.8), disorders of ocular muscles, binocular movement, accommodation and refraction (H49–H52.7), and visual disturbances and blindness (H53–H54.9). The World Health Organization criteria for vision impairment severity are based on vision in the better-seeing eye on presentation (3). The associated categories are: mild vision loss (presenting visual acuity 6/12 to 6/18), moderate vision loss (6/18 to 6/60), severe vision loss (6/60 to 3/60), blindness (<3/60 and/or a visual field of no greater than 10° in radius around central fixation), and functional presbyopia (defined as presenting near vision worse than N6 or N8 at 40 cm when best-corrected distance visual acuity was better than 6/12); each category has a corresponding disability weight for calculating DALYs.

All disease estimates from GBD contain 95% uncertainty intervals (UI) for every metric, which are defined by the 25th and 975th values of the ordered 1,000 estimates according to the GBD’s algorithm. All rates are reported per 100,000 people. Data used in this study were downloaded from the Global Health Data Exchange (GHDx) query tool^3^ and in CSV format. Data from multiple GBD files (e.g., by age, location) were merged using a unique location identifier (location_id) and year_id to create a unified panel dataset.

This analysis used the Socio-demographic Index (SDI) for each country or territory, which serves as a summary indicator of social and economic conditions that are strongly linked to treatment outcomes. The index is defined by three components: average level of education among individuals aged 15 or older, total fertility rate among those under age 25, and a composite of income per capita. SDI values are between 0 and 1, with higher values suggesting higher socioeconomic development levels. Based on 2019 SDI values, all countries were categorized into one of the five SDI quintiles: low, low-middle, middle, high-middle, and high.

The burden of blindness and vision loss was estimated by the GBD 2019 Blindness and Vision Impairment Collaborators, which included population-representative studies, Rapid Assessment of Avoidable Blindness (RAAB) surveys, and other available epidemiologic surveys as data sources for modeling of vision impairment (2, 3). The Meta-Regression with Bayesian priors, Regularization and Trimming (MR-BRT) program was used to adjust data bias resulting from alternative case definitions and study methods. In addition, DisMod-MR 2.1, a Bayesian meta-regression tool, was adopted to model the epidemiology of blindness and vision loss (3, 20).

### Analysis of overall temporal trends

To evaluate the overall temporal trends in blindness and vision loss associated DALYs in teenagers and young adults, we analyzed the number of cases and age-standardized rates from 1990 to 2019. For calculating the age-standardized DALY rate of blindness and vision loss in teenagers and young adults, global age-standard population data from GBD 2019 was used. Furthermore, the relative proportion of blindness and vision loss-associated DALYs in teenagers and young adults stratified by five age groups (15–19, 20–24, 25–29, 30–34, 35–39 years) was examined and the temporal changes in the age distribution of DALYs were illustrated.

### Age-period-cohort modeling analysis

In this study, an age-period-cohort (APC) model framework was used to analyze underlying trends in DALYs by age, period, and birth cohort. APC effects measure how humans and societies in which they live change over time. Generally, the APC model fits a log-linear Poisson model over a Lexis diagram of observed rates and quantifies the additive effects of age, period, and birth cohorts. The mathematical relationship between the three variables is essentially age = period − cohort (21). This approach has been applied to epidemiological studies for non-communicable diseases, including congenital heart disease (22). However, because the relationship between age, period and cohort is completely linear, it is statistically impossible to estimate their independent effects; this is known as the APC identification problem (21). To overcome this problem, researchers generate estimable APC parameters and functions without imposing arbitrary constraints on the model parameters (22, 23). The methodological details of the APC model have been described in previous literature (24, 25).

As data inputs for the APC model, we used population data for each country/region, and DALYs estimates for blindness and vision loss in teenagers and young adults from the GBD 2019. In a typical APC model, the age intervals must be equal to period intervals; for example, 5-years age groups should align with 5-years calendar periods. Because the age range for teenagers and young adults were defined as 15–39 years, we divided ages into five groups: 15–19, 20–24, 25–29, 30–34, and 35–39 years. Accordingly, the research period (1990–2019) was divided into six 5-years periods: 1990–1994, 1995–1999, 2000–2004, 2005–2009, 2010–2014, and 2015–2019. The input data included 10 partially overlapping 10-years birth cohorts, that is, 1950–1959, 1955–1964, 1960–1969, 1965–1974, 1970–1979, 1975–1984, 1980–1989, 1985–1994, 1990–1999, and 1995–2004.

We used the APC model to analyze the annual percentage change of DALYs known as the net drift. Net drift indicates the overall temporal trend, and is technically determined based on two components: the component of the trend attributable to calendar time and the component of the trend attributable to the successive cohorts. Additionally, we estimated the temporal trend of DALYs within each age group (local drift), expressed as annual percentage change of age-specific DALYs. Local drift reflects trends in birth cohort effect (24). Even if the value of the drift is slight, it can cause significant change in the fitted rate over a period of 30 years (22).

The Wald chi-squared test was used into the significance of trends in annual percentage change (24). The APC model outputs also included age effects, which were represented by fitted longitudinal age-specific rates in the referent cohort adjusted for period deviations. The period (or cohort) effects are represented by the period (or cohort) relative risks of DALYs, which were computed as the ratio of age-specific rates in each period (or cohort) relative to the reference period (or cohort) (24). Both the period (or cohort) rate ratio curves contained the entire value of the net drift. The choice of the reference period (or cohort) was arbitrary and did not affect the interpretation of results. A two-sided *P*-value < 0.05 was regarded as significant. R software (version 4.3.2) was used for the statistical analyses and visualization. The key R packages including tidyverse (dplyr, tidyr), data.table, ggplot2, ggpubr and so on.

## Results

### Global and regional trends

Table 1 illustrates global and regional populations, number of DALYs, age-standardized DALY rate, and net drift of DALYs. From 1990 to 2019, global population of teenagers and young adults increased from 2.19 billion (95% UI: 2.15 to 2.24) to 2.97 billion (95% UI: 2.87 to 3.07), representing a growth of 35.3%. In that period, the global number of blindness and vision loss-associated DALYs in teenagers and young adults went from 1647.78 thousand (95% UI: 1075.28 to 2385.52) to 2085.40 thousand (95% UI: 1340.56 to 3074.23), indicating an increase of approximately 26.56%. The percentage change in the number of DALYs increased in all SDI regions, especially low SDI regions reaching a large increase of 100.41%. The global age-standardized DALY rate for blindness and vision loss in teenagers and young adults was 278.22 (95% UI: 192.07 to 391.64) per 100,000 people in 2019, indicating an 8.32% decrease from 1990. From 1990 to 2019, the age-standardized DALY rate decreased in all regions except low SDI regions, where it increased by 8.81%. Furthermore, the APC model estimated a global net drift of blindness and vision loss DALYs in teenagers and young adults at −0.43% (95% CI: −0.45% to −0.42%) per year, ranging from −0.92% (95% CI: −0.94% to −0.91%) in low-middle SDI regions to −0.03% (95% CI: −0.07% to −0.0005%) in high SDI regions.

**TABLE 1:** Trends of blindness and low vision associated DALYs in teenagers and young adults by SDI quintiles, 1990–2019.

	Global		High SDI		High-middle SDI		Middle SDI		Low-middle SDI		Low SDI	
	1990	2019	1990	2019	1990	2019	1990	2019	1990	2019	1990	2019
**Population of teenagers and young adults**												
No (×10^6^)	2193.49 (2145.78, 2240.45)	2967.86 (2870.06, 3066.12)	323.06	331.30	483.53	516.74	746.62	935.13	445.33	735.29	193.81	447.72
Percentage of global level (%)	100	100	14.73	11.16	22.05	17.41	34.04	31.51	20.30	24.78	8.84	15.09
**DALYs**												
No (×10^3^)	1647.78 (1075.28, 2385.52)	2085.40 (1340.56, 3074.23)	179.44 (112.60, 267.50)	181.94 (113.45, 271.61)	307.24 (199.26, 450.05)	317.77 (201.80, 472.06)	586.38 (384.83, 840.91)	697.24 (449.39, 1027.76)	415.38 (272.16, 600.38)	569.75 (366.37, 847.89)	158.37 (104.74, 227.68)	317.39 (206.17, 464.93)
Percentage of global level (%)	100	100	10.9	8.72	18.65	15.24	35.59	33.43	25.21	27.32	9.61	15.22
Percentage change of DALYs, 1990–2019 (%)	26.56		1.39		3.43		18.91		37.16		100.41	
**Age-standardized DALYs rate**												
Rate per 100,000	303.48 (212.42, 421.19)	278.22 (192.07, 391.64)	112.70 (77.35, 156.32)	107.81 (73.20, 151.90)	248.34 (171.52, 351.32)	235.36 (160.72, 339.77)	394.98 (278.92, 542.18)	331.92 (229.31, 462.87)	553.15 (394.46, 757.26)	434.74 (301.52, 604.51)	491.19 (347.79, 672.26)	534.48 (304.97, 597.66)
Percentage change of age-standardized DALYs rate, 1990–2019 (%)	−8.32		−4.34		−5.23		−15.97		−21.41		8.81	
**APC model estimates**												
Net drift of DALYs (% per year)	−0.43 (−0.45, −0.42)		−0.03 (−0.07, −0.0005)		−0.33 (−0.36, −0.29)		−0.42 (−0.43, −0.41)		−0.92 (−0.94, −0.91)		−0.61 (−0.63, −0.59)	

Parentheses for GBD estimates denote 95% uncertainty intervals and parentheses for net drift denote 95% CIs. DALYs, disability-adjusted life-years; APC, age period cohort; GBD, Global Burden of Diseases; SDI, sociodemographic index.

Global and regional number of DALYs in different age groups were demonstrated in Table 2. Globally, in 2019, the blindness and vision loss associated DALYs were 314.56 thousand (95% UI: 198.97 to 473.28) in 15–19 years group, and increased along with age groups, reaching 609.42 thousand (95% UI: 399.29 to 902.03) in 35–39 years group. Analyzed by SDI region, DALYs increased between 15–19 and 35–39 years groups from 1990 to 2019, except low SDI regions, in which the highest change occurred in the 30–34 years group (103.53%), and the lowest change occurred in the 25–29 years group (96.5%). From 1990 to 2019, low SDI showed the greatest change across each age group, compared with global and other SDI regions.

**TABLE 2:** Trends of blindness and vision loss associated DALYs in different age groups by SDI quintiles, 1990−2019.

	Global			High SDI			High-middle SDI			Middle SDI			Low-middle SDI			Low SDI		
	1990 (×10^3^)	2019 (×10^3^)	Change (%)	1990 (×10^3^)	2019 (×10^3^)	Change (%)	1990 (×10^3^)	2019 (×10^3^)	Change (%)	1990 (×10^3^)	2019 (×10^3^)	Change (%)	1990 (×10^3^)	2019 (×10^3^)	Change (%)	1990 (×10^3^)	2019 (×10^3^)	Change (%)
15–19 years	293.83 (188.3, 434.13)	314.56 (198.97, 473.28)	7.06	31.52 (19.25, 48.36)	28.46 (17.35, 44.86)	−9.71	51.95 (32.77, 78.04)	41.99 (26.1, 64.65)	−19.18	110.04 (70.82, 161.57)	102.51 (64.5, 154.25)	−6.84	72.65 (46.39, 107.16)	86.58 (54.64, 130.01)	19.16	27.48 (17.81, 39.7)	54.81 (35.25, 80.78)	99.47
20–24 years	301.29 (191.87, 437.88)	333.11 (208.59, 492.73)	10.56	35.42 (21.17, 53.59)	32.78 (19.36, 50.22)	−7.45	54.32 (34.3, 79.76)	46.32 (28.5, 69.35)	−14.72	111.63 (71.4, 161.28)	108.82 (68.21, 160.11)	−2.52	72.56 (46.57, 104.94)	91 (57.44, 133.8)	25.42	27.17 (17.79, 38.72)	53.96 (34.59, 77.46)	98.59
25–29 years	301.91 (199.1, 429.52)	367.84 (237.77, 530.89)	21.84	37.42 (23.81, 54.92)	36.36 (22.88, 53.73)	−2.83	57.13 (37.7, 82.24)	56.29 (36.16, 81.73)	−1.47	107.63 (71.44, 153.52)	123.79 (80.96, 179.14)	15.01	71.89 (47.25, 101.11)	96.82 (62.61, 140.14)	34.67	27.65 (18.4, 38.91)	54.32 (35.34, 76.8)	96.5
30–34 years	328.71 (215.99, 476.28)	460.47 (295.93, 675.31)	40.08	36.96 (23.52, 55.06)	39.69 (25.17, 58.37)	7.39	63.79 (41.69, 93.28)	75.4 (47.71, 111.03)	18.2	112.91 (74.88, 160.96)	156.82 (101.71, 229.97)	38.88	82.65 (54.8, 119.03)	122.73 (78.6, 183.56)	48.49	32.2 (21.47, 46.78)	65.54 (43.19, 96.97)	103.53
35–39 years	422.03 (280.02, 607.71)	609.42 (399.29, 902.03)	44.40	38.13 (24.86, 55.58)	44.64 (28.68, 64.43)	17.07	80.03 (52.8, 116.73)	97.76 (63.34, 145.29)	22.14	144.16 (96.3, 203.56)	205.3 (134.01, 304.28)	42.41	115.62 (77.15, 168.13)	172.62 (113.08, 260.37)	49.3	43.87 (29.27, 63.58)	88.75 (57.8, 132.92)	102.3

### National trends

**FIGURE 1:**
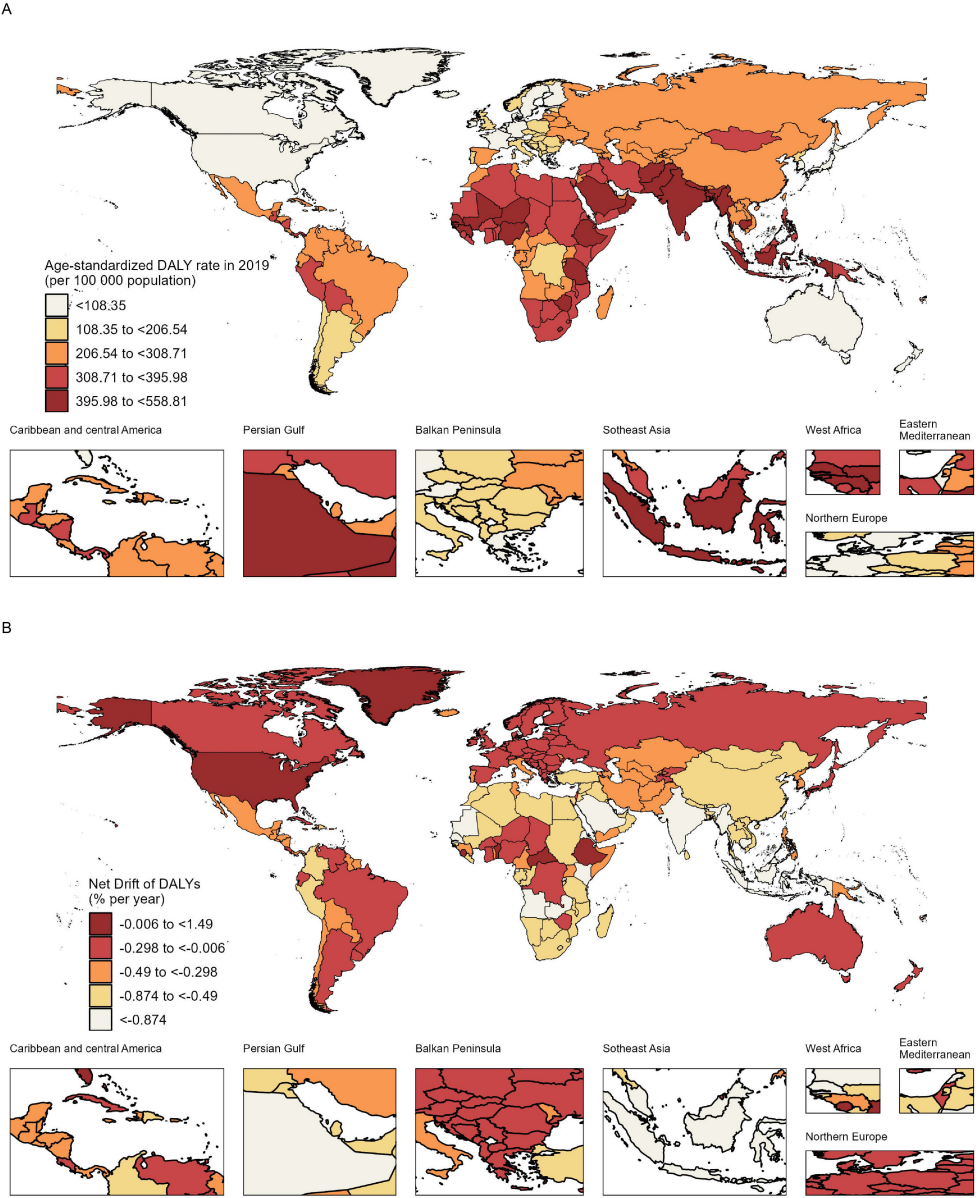
Age-standardized DALY rate in 2019 **(A)** and net drift of DALYs during 1990–2019 **(B)** for blindness and vision loss in teenagers and young adults in 204 countries and territories. In 2019, the global age-standardized rate was 278.22 (95% UI 192.07 to 391.64) per 100,000 population. Globally, the net drift from 1990–2019 was –0.43% (95% CI –0.45% to –0.42%). DALYs, disability-adjusted life-years; UI, uncertainty interval; CI, confidence interval.

Figure 1 and Supplementary Table 1 show national DALYs numbers and age-standardized DALY rate in 2019, as well as net drift of DALYs associated with blindness and vision loss in teenagers and young adults from 1990 to 2019. In 2019, among the 204 countries and territories, 40 had at least 10,000 DALYs. India, China, Brazil, Indonesia, and Pakistan had the top five most DALYs and were responsible for 48% of the total DALYs in teenagers and young adults globally. In 2019, 92 countries had at least the global age-standardized DALY rate, and 19 countries, most of which were low SDI countries, exceeded the global age-standardized DALY rate by more than 1.5-fold. And the top 5 countries with highest DALY rates of blindness and vision loss in 2019 are Indonesia, Pakistan, India, Mali, and Timor-Leste. The low SDI country of Côte d’Ivoire had the most increase in the age-standardized DALY rate (53.96%) during the study with a relatively flat net drift at 0.041% (95% CI: −0.216% to 0.207%) per year. In addition, decreasing net drifts were observed in some middle SDI countries like Equatorial Guinea, which stood out for its notable reduction in age-standardized DALY rate (−46.62%) and net drift of DALYs at −2.678% (−3.285% to −2.067%) per year. Owing to their large populations, India and China had the highest number of DALYs. Their age-standardized DALY rate decreased by −24.38% and −7.45%, respectively, with relatively modest net drifts of DALYs [India: −1.295% −1.322 to −1.267); China: −0.618% −0.716 to −0.52)]. Collectively, these results suggest that DALY trends associated with blindness and vision loss were uneven between countries, and DALY gains were not necessarily commensurate with expectations based on national SDI status. Moreover, the direction of change in DALYs indicated by conventional metrics (age-standardized DALYs) may not be exactly consistent with the change shown by the net drift derived from the APC model, suggesting that it is necessary to distinguish period and cohort trends when analyzing DALYs associated with blindness and vision loss.

### Temporal trends across different age groups

**FIGURE 2:**
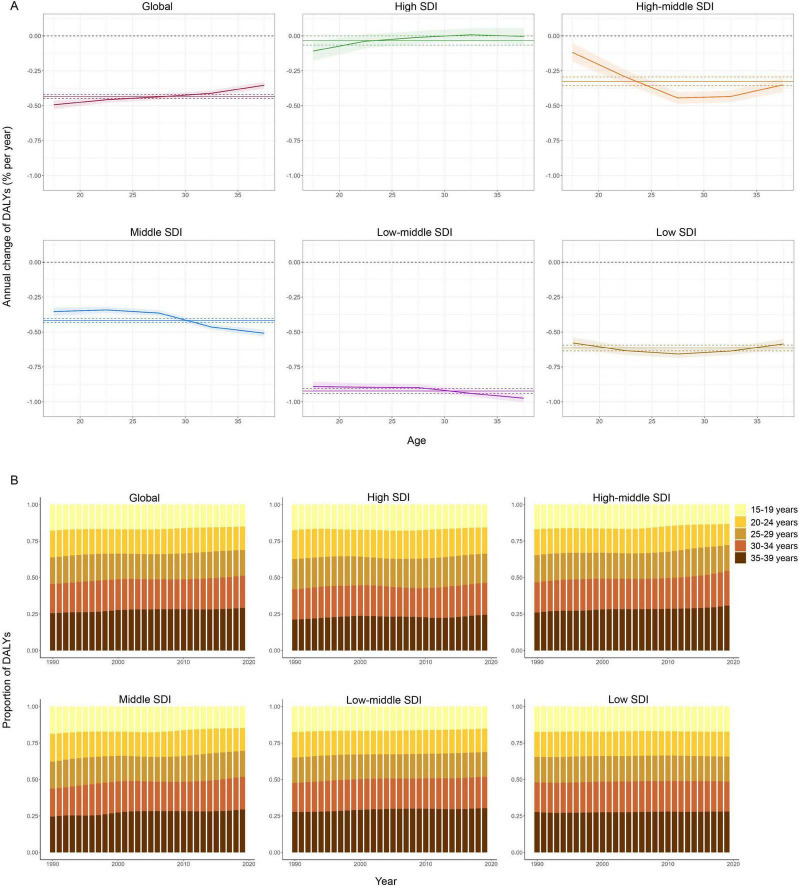
Local drift and age distribution of blindness and vision loss associated DALYs in teenagers and young adults by SDI quintiles, 1990–2019. **(A)** Local drift of DALYs from 1990 to 2019 for five age groups (15–19, 20–24, 25–29, 30–34, 35–39 years). Shaded areas indicate the annual percentage change of DALYs (% per year) and corresponding 95% confidence intervals. **(B)** Temporal changes across age groups, 1990–2019. SDI, socio-demographic index; DALYs, disability-adjusted life-years.

Figure 2A and Supplementary Table 2 present data for the annual percentage change of DALYs within each age group or local drift, calculated from the APC model. Globally, DALYs associated blindness and vision loss in teenagers and young adults showed declining trends across all age groups within the study period. The declining trend attenuated with increasing age, from −0.49% (95% CI: −0.52% to −0.46%) in the 15–19 age group to −0.35% (95 CI: −0.38% to −0.33%) in the 35–39 age group. In high SDI regions, DALYs demonstrated decreasing trends in the age groups 15–19 through 25–29, increased in the 30–34 age group, and trended flat in the 35–39 age group. The declining trend from 15–19 to 25–29 age groups attenuated with increasing age, being lowest in the adolescent stage at 15–19 years (−0.11%, 95% CI: −0.18% to −0.04%) and less evident at 20–29 years [from −0.04% (95% CI: −0.09% to 0.01%) in the 20–24 age group to −0.01% (95% CI: −0.06% to 0.03%) in the 25–29 group]. In high-middle SDI regions, the declining trend from the 15–19 age group (−0.12% per year, 95% CI: −0.18% to −0.05%) to the 25–29 group (−0.44% per year, 95% CI: −0.49% to −0.40%) increased with increasing age, and the trend is gradually faded from the 30–34 age group (−0.43% per year, 95% CI: −0.48% to −0.39%) to the 35–39 group (−0.35% per year, 95% CI: −0.40% to −0.30%). In low SDI countries, DALYs decreased across all age groups, with the steepest DALYs reduction occurring in the 25–29 age group (−0.66% per year, 95% CI: −0.69% to −0.63%). In all age groups, the DALYs consistently decreased in middle and low-middle SDI regions. Supplementary Table 3 shows the local drift of prevalence for each country.

Temporal changes in the age distribution of blindness and vision loss-associated DALYs in teenagers and young adults are illustrated in Figure 2B. Globally, there was a rising trend in DALYs from the adolescent stage (15–19 years) to the young adult stage (20–39 years), and this increase was more apparent in high-middle, and middle SDI regions. In addition, older age groups accounted for a higher proportion of DALYs, with more than 50% concentrated in adults over age 25 in all SDI regions.

### Age, period and birth cohort effects

**FIGURE 3:**
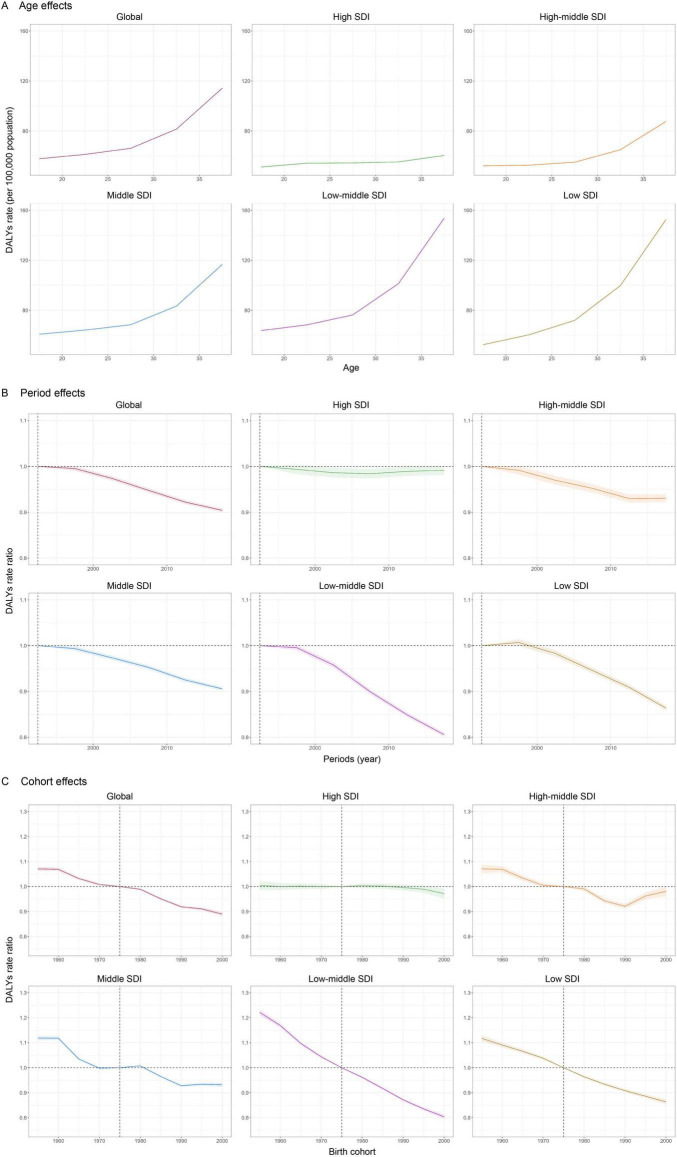
Age, period, and cohort effects on DALYs of blindness and vision loss in teenagers and young adults by SDI quintiles. **(A)** Age effects: fitted longitudinal age curves (per 100,000 person-years) adjusted for period deviations. **(B)** Period effects: period-relative risk, calculated as the ratio of age-specific rates from 1990–1994 (the referent period) to the 2015–2019 period. **(C)** Cohort effects: cohort-relative risk, calculated as the ratio of age-specific rates from the 1950–1959 cohort to the 1995–2004 cohort, with the referent cohort set at 1970–1979. Shaded areas denote DALY rates or rate ratios and their corresponding 95% confidence intervals. SDI, socio-demographic index; DALYs, disability-adjusted life-years.

The age-period-cohort effects derived from the APC model are shown in Figure 3 and Supplementary Tables 4–6. Generally, there were similar patterns in age effects across different SDI regions; in that the lowest risk in the adolescent stage was at 15–19 years and risk increased with age. Compared to other regions, high SDI regions showed overall lower DALYs across all age groups. Additionally, the 35–39 age group showed the greatest risk in low-middle and low SDI regions, at more than twice that of the high SDI regions. Period effects showed a declining trend in DALYs across different SDI regions except in the high SDI regions. For the high SDI regions, the period effect remained nearly steady throughout the study period, indicating little DALYs improvement. Compared with individuals in the reference 1990–1994 period, the relative period risk for individuals in the 2015–2019 period ranged from 0.9916 (95% CI: 0.9819 to 1.0013) in the high SDI region to 0.8057 (95% CI: 0.8014 to 0.81) in the low-middle SDI region and 0.8636 (95% CI: 0.8581 to 0.869) in the low SDI region. Globally, the risk declined overall in successively younger birth cohorts.

As with period effects, declining cohort effects were more evident in the low-middle SDI region. Furthermore, there was a declining risk of DALYs across different SDI regions from the 1950–1959 cohort, while the risk in the high SDI region did not decrease until after 1990–1999 cohort. Compared with individuals born in the reference 1970–1979 cohort, the relative cohort risk for individuals born in the 1995–2004 cohort ranged from 0.8039 (95% CI: 0.7953 to 0.8126) in the low-middle SDI region to 0.981 (95% CI: 0.961 to 1.0015) in the high-middle SDI region.

### Age-period-cohort effects in representative countries

**FIGURE 4:**
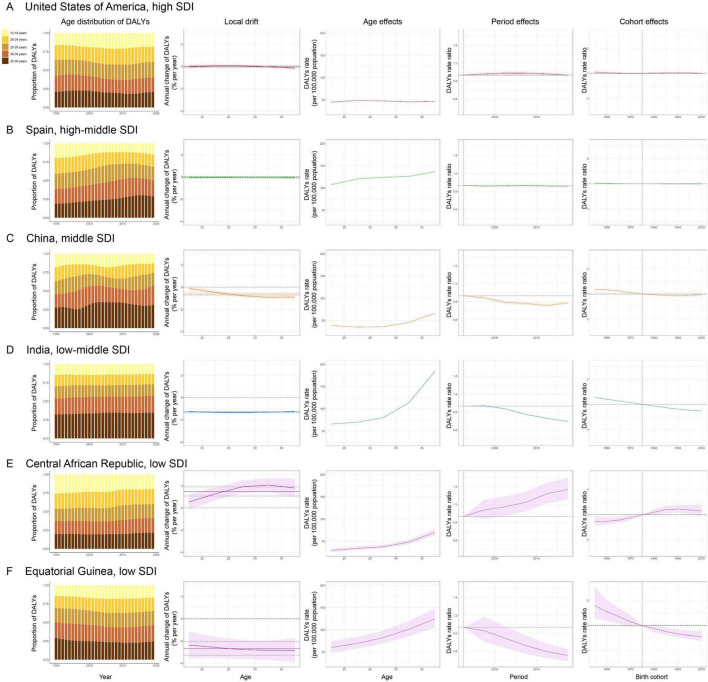
**(A–F)** Age, period, and birth cohort effects on DALYs of blindness and vision loss in teenagers and young adults in representative countries from each SDI. Age distribution of DALYs: changes in relative proportion of DALYs from 1990 to 2019 across five age groups (15–19, 20–24, 25–29, 30–34, 35–39 years). Local drift: annual percentage change across five age groups. Age effects: fitted longitudinal age curves (per 100,000 person-years), adjusted for period deviations. Period effects: period-relative risk, calculated as the ratio of age-specific rates in each period compared to the referent 1990–1994 period. Cohort effects: cohort-relative risk, calculated as the ratio of age-specific rates in each cohort compared to the referent 1970–1979 cohort. Shaded areas indicate the corresponding 95% confidence intervals of each point estimate. SDI, socio-demographic index; DALYs, disability-adjusted life-years.

Supplementary Tables 7–9 show age-period-cohort effects for individual countries. We analyzed countries of different SDI quintiles to better characterize major trends round the world through relatively favorable and unfavorable age-period-cohort effects (Figure 4). The United States is typical of trends in high SDI countries with unfavorable age-period-cohort effects, where an increase was observed across the 15–19 through 30–34 age groups with increased period and cohort effects over the entire study period. Spain had extremely favorable trends among high-middle SDI countries, with local drift of <0% per year for all age groups, and noticeable declining risks over progressive period and in successive birth cohorts. China and India, examples of highly populous middle and low-middle SDI countries, presented a decreasing trend of DALYs across all age groups and favorable declining period and cohort risks. The Central African Republic stood out for its highest net drift among low SDI countries, showing significant DALYs increases in all age groups, with steady deterioration in both period and cohort risks. Equatorial Guinea, another low SDI country, showed a local drift of <0% per year in all age groups, and significant declining risk over progressive periods and in successive birth cohorts.

## Discussion

In a visual-centric world, vision plays a crucial role in every facet of our lives. According to the World Health Organization’s World report on vision (26), eye diseases and vision impairment are widespread, but often go untreated. Globally, at least 2.2 billion people have a vision impairment, and of these, at least 1 billion people have a vision impairment that could have been prevented or is yet to be addressed. Previous studies have reported that vision impairment in teenagers and young adults may have lifelong effects (7, 8), including self-learning ability, athletic ability, working competence and social adaptive capability (9–11). It has also been reported that the number of visual impairment-associated DALYs increases with advancing age (16, 17). Therefore, an in-depth understanding of the trends in DALYs associated with blindness and vision loss in teenagers and young adults can help countries and regions build policy to prevent and manage vision impairment. The lack of a global overview and analysis of DALYs associated with vision impairment in teenagers and young adults suggests an urgent need. It is well known that the estimates of absolute numbers of DALYs provide insights on current situations and are thus useful for policymakers, while age-standardized estimates allow comparisons over time and across countries and regions, adjusting for differences in age distribution in different populations. Therefore, in this study, we used both absolute numbers and age-standardized rates. We further used the APC model to conduct a comprehensive analysis of trends on a global scale, allowing for comparisons between different regions and countries. Traditional epidemiological measures–such as crude prevalence and incidence rates–provide a snapshot of disease burden but fail to account for temporal variations in risk factors, diagnostic practices, or treatment advancements (14, 27). Similarly, while AAPC (average annual percent change, derived from Joinpoint regression) quantifies average trends over time, it does not distinguish whether observed changes are due to aging populations (age effect), changes in screening/diagnosis (period effect), and generational exposures (cohort effect) (23, 28, 29). The APC model’s ability to decompose temporal trends makes it indispensable for understanding blindness and vision loss dynamics. While traditional prevalence/incidence and AAPC analyses provide descriptive summaries, APC guides etiologic research and precision public health strategies.

From 1990 to 2019, the global population increased by 44.63%, and the total number of DALYs associated with blindness and vision loss in teenagers and young adults increased by 26.56%, with the largest increase observed in low SDI regions. It is worth noting that the large increase in low SDI regions was mainly mediated by population growth. The total population increased by 113.71%, while the age-standardized rate of DALYs markedly decreased by −11.34%. For example, Afghanistan had the highest increase in population growth among low SDI regions, and while the DALYs associated with blindness and vision loss has increased with aging, the age-standardized rate actually decreased slightly, by −3.19%. Illiteracy, advanced age, poor economic status, hypertension, and excess weight have been associated with blindness and vision loss in Afghanistan (30, 31). Thus, based on risk factors for vision impairment in Afghanistan, it is recommended to make eye health services more accessible to older and economically deprived individuals, and to apply primary prevention measures for hypertension and overweight (30, 31). We also found that the age-standardized DALY rate varied among different SDI regions, but was negatively correlated with SDI at the regional level. Higher SDI regions have better healthcare infrastructure and economic development, which lead to lower DALYs (20). Therefore, health policymakers should customize measures based on socioeconomic factors in different SDI regions, which includes training healthcare personnel, strengthening supply chains for essential medicines and equipment, and integrating eye health into primary care systems.

Our study analyzed local drift, which examined trends within specific age groups, for DALYs associated with blindness and vision loss. High SDI regions showed attenuated declining trends in more age groups than in other SDI regions, suggesting a potential SDI-related distributive inequity. It is worth noting that trends observed in this study may not be fully consistent with the general assumption that countries with a higher SDI have eye care quality, better medical facilities and advanced medical technology, thus resulting in lower disease burden. On the one hand, this discrepancy may be due to the rapid development of middle and lower SDI countries and the World Health Organization-supported Vision 2020 and Universal Eye Health programs over the past few decades. On the other hand, individuals in high SDI regions are more likely to spend more time indoors and engage in more close work activities, leading to an increase in myopia, and complications such as retinal detachment and cataracts (32). Further, the epidemic of obesity contributes to diabetes in higher SDI regions (33), resulting in risk factors for several eye disorders such as diabetic retinopathy, which can lead to vision impairment. In addition, while the 35–39 age group accounted for over a quarter of blindness and vision loss-associated DALYs in teenagers and young adults across most regions, DALYs in the younger age groups appeared to be more prominent in high SDI regions.

The relative effects of age, period, and birth cohort on DALYs trends were also explored in our study. Age effects showed similar patterns across different SDI regions, with the lowest risk observed in teenagers 15–19 years, and risk increased with age. However, it is notable that the risk increases more slowly with age in high SDI regions, while it increases rapidly with age in low-middle and low SDI regions. In fact, population aging is recognized as one of the most prominent risk factors for vision impairment, potentially resulting in considerable increases in the number of people with eye conditions that cause blindness and vision loss. After adjusting for the effect of population size in each age group, studies have shown that DALYs associated with blindness and vision loss increase with age (17, 18). Thus, blindness and vision loss continue to be a significant public health burden, especially in older populations. Therefore, the development of robust vision care systems that can keep pace with the aging and growth of populations is the need of the hour, including availability and accessibility of surgical services and spectacles. The key measures for protecting the vision of the older populations, including regular comprehensive eye examinations, manage underlying health conditions (such as diabetes, high blood pressure and high cholesterol), adopt a nutrient-rich diet, wear protective eyewear, make lifestyle adjustment, and use proper lighting and reduce glare.

Period risks decreased over the study period globally and across SDI regions, except in the high SDI regions. Unfavorable period effects in the high SDI region may be associated with changes in lifestyle causing vision impairment, including increased of near work, increased rates of urbanization, reduced outdoor activities, and rising obesity (34–37). Regarding cohort effects, individuals born in later periods had a lower risk than those born earlier. On one hand, earlier-born individuals may accumulate risk over their lifetime from factors such as lifestyle, nutrition, occupation, recreational activities, ocular infections, and various health conditions (3, 38–42). On the other hand, in recent years, advances in medical technology, the growth of cataract surgery, and improved refractive error correction methods, have helped to reduce the risk of blindness and vision loss (43, 44). Individuals born in later time periods may have had more effective interventions compared to those born earlier therefore encountered lower risk.

It is worth noting that the net drift represents overall temporal trends, while differentiating age, period, and cohort trends provides the opportunity to recognize the more specific factors driving changes in DALYs. The decline of blindness and vision loss associated-DALYs in teenagers and young adults in high-middle, middle, low-middle and low SDI regions appeared to be primarily driven by favorable cohort effects, while the very slight decreases in the high SDI region appear to be mainly driven by an unfavorable cohort effect. In addition, we identified a strong heterogeneity in age, period, and birth cohort effects across 204 countries and territories. This suggests that distinct disease patterns exist worldwide, requiring individualized attention in the formation of national health policies.

To our knowledge, this is the first use of APC models to comprehensively analyze trends of blindness and vision loss-associated DALYs in teenagers and young adults at global, regional, and national levels. Compared with previous GBD publications regarding blindness and vision loss (17, 18, 45), our study provides a more detailed understanding of disease trends, offering valuable insights into epidemiology and public health policy-making. In particular, the examination of period and cohort effects enables us to differentiate trends by time periods and birth cohorts for each country, providing information that can be used for more effective eye care services. Estimation of overall time trends (net drift) and trends within specific age groups (local drift) enable us to capture trends for each age group, while adjusting for period effects. Moreover, the differentiation of period and birth cohort effects enables us to determine the major factors driving changes in DALYs trends by periods and birth cohorts for each country. This allows for comparisons among different countries and provides guidance for effective interventions in prevention, treatment, and rehabilitation that are specific to each country.

The study has several limitations. First, the GBD relies heavily on modeled data, particularly at the country level, due to the lack of primary data. Thus, estimates from the GBD in some countries have wide uncertainty bounds (14). This might affect the accuracy of estimates of age, period, and birth cohort trends and may overstate the improvements in some countries. Second, because GBD uses different methodologies to estimate the burden of infectious versus systemic diseases, DALYs for conditions like diabetic retinopathy and causes of corneal blindness were unavailable from the database, which led to an underestimation of the total burden of blindness and vision loss. Third, subnational characterizations of DALYs trends cannot be explored due to data unavailability. Further analysis should be conducted at the subnational level to balance differences in national population size.

In conclusion, our APC analysis of DALYs associated with blindness and vision loss found that decreases in DALYs in the past 30 years are not always commensurate with a country’s socioeconomic development. This study found that there were unfavorable increasing trends in some countries worldwide, coupled with deteriorations in period/cohort risks, collectively suggesting that current resources invested in vision care in these countries may be inadequate. To eliminate avoidable blindness and achieve the ultimate goal of the WHO Universal Eye Health program, increased resource investment, especially at adulthood, is urgently needed to reduce the risk of progressively greater DALYs associated with blindness and vision loss for successively younger birth cohorts and across all age groups. Additionally, the region-specific nature of DALYs related to blindness and vision loss indicates that targeted policymaking and resource allocation should be further considered.

## Data Availability

Publicly available datasets were analyzed in this study. This data can be found here: https://ghdx.healthdata.org/gbd-2019.

## Footnotes

1
http://ghdx.healthdata.org/gbd-2019/datainput-sources

2
https://icd.who.int/browse10/2019/en

3
https://vizhub.healthdata.org/gbd-results/
