# Supplementary material for: Global, regional, and national trends in DALYs for blindness and vision loss in teenagers and young adults, 1990–2019: an age-period-cohort analysis based on the Global Burden of Disease Study 2019
Source: Front Med (Lausanne). 2025 Oct 29;12:1624618. doi: 10.3389/fmed.2025.1624618 (PMC12605158; doi:10.3389/fmed.2025.1624618)
Supplement: Supplementary file 1 [file Table_1.docx]

**Supplementary Materials**

**Contents**

Table S12

Table S211

Table S312

Table S436

Table S537

Table S638

Table S740

Table S864

Table S993

**Table S1 The DALYs number and age-standardized DALYs rate in 2019, as well as *net drift* of DALYs from 1990 to 2019 for blindness and vision loss in teenagers and young adults across countries**

| **Location** | **DALYs number** | **Age-standardized DALYs rate** | **Net Drift (%/year)** |
| --- | --- | --- | --- |
| Afghanistan | 16477.31 (10550.62, 24048.92) | 469.63 (332.97, 632.84) | -0.43 (-0.53, -0.32) |
| Albania | 349.53 (211.48, 541.71) | 171.70 (109.97, 261.18) | -0.22 (-0.68, -0.24 |
| Algeria | 15021.85 (9492.64, 22127.67) | 321.38 (227.71, 436.83) | -0.65 (-0.74, -0.57) |
| American Samoa | 8.70 (5.34, 13.27) | 240.50 (165.53, 346.00) | -0.83 (-3.93, 2,34) |
| Andorra | 15.02 (9.08, 22.65) | 104.88 (71.36, 145.38) | -0.20 (-2.64, 2.30) |
| Angola | 5245.55 (3196.82, 7934.82) | 290.05 (196.30, 417.94) | -0.91 (-1.07, -0.75) |
| Antigua and Barbuda | 22.00 (13.61, 33.30) | 193.55 (131.78, 276.98) | -0.39 (-2.49, 1.76) |
| Argentina | 12677.29 (2814.82, 19215.38) | 149.70 (103.52, 206.56) | -0.26 (-0.35, -0.62) |
| Armenia | 632.40 (393.53, 950.41) | 266.75 (181.29, 383.62) | -0.38 (-0.74, -0.01) |
| Australia | 4397.22 (2643.17, 6740.23) | 101.27 (68.68, 141.04) | -0.12 (-0.27, 0.03) |
| Austria | 1667.25 (1014.15, 2547.68) | 106.69 (73.50, 147.65) | -0.16 (-0.39, 0.07) |
| Azerbaijan | 2489.89 (1560.83, 3741.47) | 277.16 (189.65, 394.14) | -0.41 (-0.61, -0.21) |
| Bahamas | 93.63 (57.99, 142.05) | 193.10 (131.87, 277.68) | -0.23 (-1.26, 0.80) |
| Bahrain | 520.73 (327.51, 772.17) | 286.94 (201.31, 392.23) | -0.73 (-1.24, -0.22) |
| Bangladesh | 45961.80 (27424.42, 71864.28) | 447.73 (305.62, 632.60) | -0.77 (-0.82, -0.72) |
| Barbados | 33.87 (19.98, 52.84) | 116.21 (74.64, 178.97) | -0.16 (-1.78, 1.5) |
| Belarus | 1587.75 (962.73, 2455.20) | 235.47 (153.64, 359.61) | -0.26 (-0.50 -0.03) |
| Belgium | 2068.37 (1256.98, 3143.38) | 106.43 (72.69, 147.36) | -0.20 (-0.40, 0.01) |
| Belize | 115.84 (71.31, 173.64) | 213.12 (143.97, 304.06) | -0.47 (-1.55, 0.62) |
| Benin | 2899.03 (1793.42, 4369.52) | 365.77 (252.23, 509.84) | 0.12 (-0.12, 0.35) |
| Bermuda | 11.92 (7.33, 18.05) | 183.94 (125.20, 268.85) | -0.67 (-3.28, 2.02) |
| Bhutan | 177.59 (104.14, 289.54) | 289.52 (191.36, 431.35) | -0.74 (-1.52, 0.05) |
| Bolivia (Plurinational State of) | 4688.52 (2960.56, 7002.90) | 326.73 (230.35, 450.20) | -0.48 (-0.64, -0.33) |
| Bosnia and Herzegovina | 376.32 (224.96, 585.16) | 166.02 (106.42, 250.74) | -0.27 (-0.71, 0.16) |
| Botswana | 786.53 (467.67, 1227.78) | 383.45 (262.70, 564.85) | -0.85 (-1.26, -0.43) |
| Brazil | 103999.95 (66101.00, 153848.10) | 305.78 (214.23, 416.58) | -0.25 (-0.28, -0.22) |
| Brunei Darussalam | 116.46 (71.40, 175.79) | 105.44 (72.86, 146.34) | -0.17 (-1.13, 0.80) |
| Bulgaria | 728.85 (441.10, 1144.69) | 161.65 (102.91, 249.25) | -0.18 (-0.51, 0.16) |
| Burkina Faso | 5133.53 (3195.46, 7625.98) | 393.66 (275.59, 544.76) | -0.06 (-0.24, 0.12) |
| Burundi | 1499.39 (909.73, 2387.61) | 216.57 (140.36, 330.46) | -0.65 (-0.96, -0.34) |
| Cabo Verde | 112.80 (69.06, 173.511) | 282.34 (193.64, 401.96) | -0.81 (-1.83, 0.23) |
| Cambodia | 5947.67 (3714.39, 8831.12) | 447.35 (317.02, 611.82) | -1.80 (-1.92, -1.67) |
| Cameroon | 5971.81 (3646.60, 9062.19) | 290.28 (200.24, 407.94) | -0.34 (-0.51, -0.17) |
| Canada | 5022.63 (3027.00, 7779.37) | 81.25 (55.90, 112.94) | -0.10 (-0.23, 0.04) |
| Central African Republic | 1004.40 (608.95, 1521.02) | 230.94 (152.85, 339.11) | 1.49 (1.08, 1.89) |
| Chad | 3628.06 (2276.12, 5422.71) | 374.88 (259.54, 517.98) | -0.08 (-0.28, 0.13) |
| Chile | 4632.38 (2789.20, 7047.79) | 133.87 (92.05, 184.57) | -0.39 (-0.53, -0.25) |
| China | 230597.55 (147372.96, 347420.39) | 247.45 (167.99, 361.90) | -0.62 (-0.72, -0.52) |
| Colombia | 17536.79 (10852.00, 26158.11) | 291.65 (200.88, 410.83) | -0.54 (-0.61, -0.46) |
| Comoros | 141.92 (86.16, 214.64) | 327.00 (224.82, 462.67) | -0.98 (-1.85, -0.11) |
| Congo | 959.90 (575.74, 1470.16) | 264.50 (177.64, 384.90) | -0.79 (-1.15, -0.43) |
| Cook Islands | 2.76 (1.70, 4.17) | 260.65 (179.10, 366.15) | -0.19 (-4.72, 4.56) |
| Costa Rica | 1684.51 (1053.04, 2527.33) | 265.49 (181.87, 370.89) | -0.24 (-0.48, 0.02) |
| Croatia | 459.46 (274.20, 721.47) | 158.42 (100.46, 245.48) | -0.19 (-0.61, 0.24) |
| Cuba | 3294.69 (2075.29, 4897.99) | 265.93 (184.18, 369.26) | -0.20 (-0.36, -0.05) |
| Cyprus | 297.90 (181.92, 451.82) | 105.24 (72.08, 145.91) | -0.28 (-0.90, 0.33) |
| Czechia | 1087.76 (648.77, 1687.40) | 154.71 (97.65, 239.52) | -0.20 (-0.49, 0.08) |
| Côte d'Ivoire | 6522.24 (4051.76, 9736.41) | 374.07 (259.81, 520.66) | 0.04 (-0.22, 0.21) |
| Democratic People's Republic of Korea | 2427.02 (1344.62, 4183.39) | 154.61 (91.70, 257.36) | -0.46 (-0.66, -0.26) |
| Democratic Republic of the Congo | 11569.73 (6778.79, 18378.50) | 190.53 (121.30, 297.07) | -0.16 (-0.27, -0.05) |
| Denmark | 1049.15 (638.71, 1602.82) | 105.45 (72.41, 145.33) | -0.16 (-0.44, 0.13) |
| Djibouti | 263.06 (162.03, 397.03) | 318.78 (219.33, 444.84) | -1.14 (-1.89, -0.39) |
| Dominica | 16.14 (10.01, 24.36) | 200.86 (136.65, 284.82) | -0.17 (-2.39, 2.10) |
| Dominican Republic | 3723.38 (2294.24, 5602.65) | 256.85 (176.62, 362.26) | -0.56 (-0.72, -0.40) |
| Ecuador | 5230.78 (3210.50, 7903.85) | 243.97 (167.39, 344.62) | -0.26 (-0.41, -0.11) |
| Egypt | 37914.34 (23370.74, 56854.11) | 330.49 (230.59, 453.36) | -0.63 (-0.69, -0.58) |
| El Salvador | 2781.69 (1745.56, 4122.93) | 334.46 (231.47, 460.83) | -0.48 (-0.66, -0.30) |
| Equatorial Guinea | 357.41 (218.33, 535.33) | 333.72 (232.69, 462.62) | -2.68 (-3.29, -2.07) |
| Eritrea | 1689.45 (1045.72, 2514.30) | 385.56 (267.61, 535.23) | -0.98 (-1.26, -0.71) |
| Estonia | 149.00 (88.95, 231.33) | 180.54 (112.15, 289.43) | -0.20 (-0.97, 0.58) |
| Eswatini | 365.60 (218.68, 575.08) | 391.39 (265.78, 576.41) | -0.62 (-1.17, -0.06) |
| Ethiopia | 32867.00 (21657.80, 46965.41) | 420.96 (303.10, 568.56) | 0.01 (-0.06, 0.07) |
| Fiji | 185.85 (115.05, 277.75) | 307.71 (214.796, 430.42) | -0.48 (-1.18, 0.23) |
| Finland | 992.33 (599.87, 1511.09) | 104.08 (71.22, 143.99) | -0.20 (-0.49, 0.09) |
| France | 10892.07 (6455.27, 16975.14) | 94.64 (63.74, 134.02) | -0.15 (-0.24, -0.06) |
| Gabon | 301.15 (179.96, 465.08) | 251.66 (169.25, 363.37) | -0.83 (-1.44, -0.21) |
| Gambia | 520.07 (321.97, 787.85) | 363.16 (250.63, 510.36) | -1.19 (-1.67, -0.71) |
| Georgia | 677.71 (424.70, 1019.34) | 270.81 (184.99, 386.09) | -2.18 (-2.50, -1.86) |
| Germany | 14792.49 (8955.24, 22592.48) | 103.64 (71.34, 144.49) | -0.14 (-0.22, -0.07) |
| Ghana | 7699.97 (4742.17, 11749.21) | 328.30 (224.09, 469.07) | -0.06 (-0.20, 0.07) |
| Greece | 1519.39 (919.62, 2342.04) | 98.44 (67.96, 136.31) | -0.28 (-0.50, -0.06) |
| Greenland | 9.62 (5.88, 14.58) | 91.06 (63.39, 125.87) | 0.11 (-2.72, 3.02) |
| Grenada | 25.46 (15.51, 38.54) | 201.73 (137.07, 289.18) | -0.37 (-2.21, 1.51) |
| Guam | 23.64 (14.37, 36.19) | 229.56 (157.27, 330.69) | -0.30 (-2.16, 1.60) |
| Guatemala | 9418.01 (5931.24, 13909.39) | 379.61 (264.91, 520.56) | -0.38 (-0.50, -0.25) |
| Guinea | 3142.86 (1966.98, 4658.72) | 403.86 (279.99, 560.01) | -0.36 (-0.57, -0.16) |
| Guinea-Bissau | 492.43 (308.05, 732.04) | 418.36 (294.03, 581.93) | -0.65 (-1.16, -0.15) |
| Guyana | 207.18 (128.03, 311.87) | 212.83 (146.47, 301.80 | -0.31 (-0.95, 0.33) |
| Haiti | 4151.21 (2599.05, 6101.23) | 259.77 (181.32, 362.33) | -0.42 (-0.59, -0.24) |
| Honduras | 3818.68 (2376.87, 5697.22) | 288.83 (197.49, 410.70) | -0.39 (-0.58, -0.20) |
| Hungary | 1057.70 (631.60, 1647.76) | 165.77 (106.51, 250.45) | -0.19 (-0.47, 0.09) |
| Iceland | 67.18 (41.26, 101.62) | 108.34 (75.47, 147.92) | -0.32 (-1.49, 0.86) |
| India | 489920.41 (308647.52, 748483.65) | 525.44 (365.60, 727.03) | -1.30 (-1.32, -1.27) |
| Indonesia | 103968.78 (68839.89, 148181.26) | 558.80 (404.12, 747.91) | -0.93 (-0.96, -0.90) |
| Iran (Islamic Republic of) | 41018.59 (26956.55, 58846.61) | 376.08 (267.11, 505.76) | -0.42 (-0.47, -0.36) |
| Iraq | 15416.07 (9619.71, 23054.87) | 316.43 (224.05, 429.55) | -0.69 (-0.78, -0.59) |
| Ireland | 946.75 (581.92, 1440.36) | 105.56 (72.66, 147.07) | -0.26 (-0.57, 0.05) |
| Israel | 1918.06 (1161.61, 2926.63) | 107.92 (73.82, 148.42) | -0.18 (-0.42, 0.06) |
| Italy | 15054.75 (9587.48, 22134.27) | 173.88 (122.60, 235.93) | -0.33 (-0.40, -0.26) |
| Jamaica | 731.19 (443.51, 1110.88) | 195.667 (131.47, 282.75) | -0.42 (-0.77, -0.06) |
| Japan | 15832.90 (9944.21, 23516.00) | 93.40 (64.81, 128.77) | -0.24 (-0.31, -0.17) |
| Jordan | 3170.23 (1942.52, 4803.62) | 240.68 (167.71, 334.31) | -0.62 (-0.85, -0.39) |
| Kazakhstan | 3990.90 (2469.61, 5985.79) | 268.36 (183.69, 384.99) | -0.34 (-0.50, -0.19) |
| Kenya | 12193.46 (8035.04, 17344.01) | 367.36 (257.62, 503.53) | -1.22 (-1.32, -1.12) |
| Kiribati | 24.70 (15.19, 36.67) | 287.51 (197.03, 404.96) | -0.35 (-2.45, 1.79) |
| Kuwait | 1613.04 (1009.58, 2444.97) | 271.72 (191.10, 373.77) | -0.50 (-0.81, -0.19) |
| Kyrgyzstan | 1242.77 (763.45, 1881.74) | 257.42 (174.65, 364.79) | -0.28 (-0.57, 0.01) |
| Lao People's Democratic Republic | 1405.80 (844.52, 2155.19) | 260.55 (177.95, 368.38) | -0.81 (-1.09, -0.52) |
| Latvia | 282.15 (171.63, 434.24) | 228.84 (148.22, 348.73) | -0.25 (-0.77, 0.27) |
| Lebanon | 1842.68 (1152.65, 2758.24) | 307.68 (215.62, 419.23) | -0.93 (-1.16, -0.69) |
| Lesotho | 751.80 (460.08, 1160.84) | 422.77 (287.90, 610.91) | -0.51 (-0.89, -0.14) |
| Liberia | 1163.57 (722.07, 1736.28) | 341.47 (238.63, 478.02) | -0.78 (-1.13, -0.42) |
| Libya | 2556.22 (1607.90, 3772.41) | 330.18 (233.90, 448.10) | -0.61 (-0.82, -0.40) |
| Lithuania | 414.58 (249.97, 640.20) | 229.10 (149.79, 345.64) | -0.23 (-0.64, 0.19) |
| Luxembourg | 124.80 (75.98, 190.13) | 103.90 (71.33, 144.03) | -0.19 (-1.13, 0.77) |
| Madagascar | 4686.34 (2891.99, 7218.89) | 288.36 (196.21, 414.54) | -0.73 (-0.90, -0.56) |
| Malawi | 4213.70 (2618.14, 6308.01) | 372.38 (261.02, 519.80) | -0.84 (-1.01, -0.67) |
| Malaysia | 8311.30 (5097.19, 12515.98) | 314.36 (219.82, 435.39) | -0.95 (-1.06, -0.83) |
| Maldives | 124.48 (74.66, 191.83) | 263.72 (178.60, 378.23) | -0.95 (-2.07, 0.18) |
| Mali | 6205.86 (3919.12, 9200.90) | 512.99 (361.86, 701.65) | -0.84 (-0.99, -0.69) |
| Malta | 82.50 (50.19, 125.67) | 107.96 (73.79, 148.86) | -0.16 (-1.18, 0.86) |
| Marshall Islands | 11.63 (7.07, 17.27) | 269.89 (184.95, 379.70) | -0.51 (-3.32, 2.37) |
| Mauritania | 842.72 (518.23, 1290.00) | 347.16 (240.51, 485.41) | -1.03 (-1.39, -0.66) |
| Mauritius | 259.72 (157.66, 399.91) | 295.51 (202.54, 417.86) | -0.61 (-1.16, -0.06) |
| Mexico | 44116.99 (27910.26, 65555.60) | 269.09 (185.40, 373.54) | -0.40 (-0.45, -0.35) |
| Micronesia (Federated States of) | 18.69 (11.27, 28.05) | 262.26 (179.62, 372.99) | -0.86 (-2.91, 1.23) |
| Monaco | 5.40 (3.30, 8.26) | 104.33 (71.07, 145.15) | -0.04 (-4.15, 4.24) |
| Mongolia | 855.89 (535.77, 1269.44) | 311.85 (215.82, 439.86) | -0.69 (-1.04, -0.33) |
| Montenegro | 75.11 (44.64, 116.77) | 161.17 (102.60, 247.76) | -0.16 (-1.23, 0.92) |
| Morocco | 10595.56 (6542.17, 15907.52) | 275.36 (193.02, 379.92) | -0.62 (-0.71, -0.52) |
| Mozambique | 6334.39 (3951.48, 9336.55) | 380.51 (265.92, 528.03) | -0.83 (-0.97, -0.69) |
| Myanmar | 17999.62 (11292.60, 26414.41) | 421.10 (296.33, 575.19) | -1.13 (-1.20, -1.06) |
| Namibia | 682.53 (407.09, 1086.04) | 374.13 (252.76, 551.27) | -0.81 (-1.23, -0.4) |
| Nauru | 1.95 (1.18, 2.97) | 248.33 (167.43, 354.96) | -0.42 (-6.20, 5.71) |
| Nepal | 10231.80 (5997.75, 16454.56) | 478.28 (313.59, 719.67) | -0.51 (-0.62, -0.40) |
| Netherlands | 2652.74 (1604.70, 4037.33) | 88.05 (59.50, 124.73) | -0.20 (-0.37, -0.02) |
| New Zealand | 782.04 (477.83, 1190.04) | 104.63 (71.85, 146.05) | -0.25 (-0.58, 0.08) |
| Nicaragua | 2738.04 (1718.84, 4084.76) | 312.16 (216.53, 437.18) | -0.47 (-0.68, -0.27) |
| Niger | 5789.31 (3539.95, 8854.86) | 434.20 (299.14, 604.58) | -0.24 (-0.41, -0.08) |
| Nigeria | 67298.50 (44640.15, 96324.23) | 467.75 (329.60, 646.68) | -0.18 (-0.23, -0.14) |
| Niue | 0.22 (0.13, 0.34) | 229.06 (155.96, 329.60) | -0.40 (-17.05, 19.58) |
| North Macedonia | 283.33 (170.47, 436.80) | 164.34 (104.86, 251.34) | -0.26 (-0.84, 0.29) |
| Northern Mariana Islands | 5.17 (3.10, 7.95) | 228.88 (155.72, 329.01) | -0.18 (-3.75, 3.52) |
| Norway | 1161.07 (712.59, 1761.23) | 114.76 (78.56, 159.75) | -0.08 (-0.37, 0.20) |
| Oman | 3706.33 (2344.65, 5429.12) | 455.03 (325.68, 615.30) | -0.54 (-0.76, -0.33) |
| Pakistan | 79657.74 (51705.75, 115945.42) | 539.49 (385.43, 730.28) | -0.43 (-0.47, -0.39) |
| Palau | 2.51 (1.53, 3.87) | 227.97 (154.47, 325.85) | -1.18 (-6.09, 3.99) |
| Palestine | 1668.63 (1037.80, 2509.95) | 299.41 (210.74, 410.28) | -0.66 (-0.94, -0.37) |
| Panama | 1511.61 (945.61, 2235.90) | 313.04 (219.85, 433.58) | -0.34 (-0.61, -0.07) |
| Papua New Guinea | 2679.55 (1678.80, 3982.98) | 360.98 (250.84, 502.27) | -0.34 (-0.57, -0.11) |
| Paraguay | 3707.23 (2308.12, 5556.13) | 307.34 (212.62, 431.56) | -0.31 (-0.49, -0.14) |
| Peru | 15135.91 (9365.27, 22670.90) | 353.89 (247.05, 490.87) | -0.54 (-0.63, -0.46) |
| Philippines | 27378.40 (17272.66, 40552.91) | 315.92 (220.09, 434.36) | -0.45 (-0.51, -0.38) |
| Poland | 4744.86 (2965.17, 7139.89) | 149.52 (97.95, 222.88) | -0.08 (-0.22, 0.06) |
| Portugal | 1847.71 (1126.03, 2824.08) | 110.71 (76.34, 154.90) | -0.31 (-0.51, -0.11) |
| Puerto Rico | 696.78 (421.60, 1067.79) | 179.89 (121.26, 262.40) | -0.33 (-0.65, 0.004) |
| Qatar | 1377.77 (854.08, 2067.44) | 287.43 (202.38, 394.39) | -0.87 (-1.36, -0.38) |
| Republic of Korea | 10006.08 (6217.21, 15123.79) | 104.36 (71.75, 145.46) | -0.47 (-0.56, -0.39) |
| Republic of Moldova | 862.21 (532.61, 1324.63) | 299.78 (202.80, 437.92) | -0.31 (-0.62, 0.01) |
| Romania | 2089.76 (1260.36, 3251.36) | 166.58 (106.73, 253.88) | -0.21 (-0.40, -0.01) |
| Russian Federation | 29701.19 (18553.94, 45050.43) | 259.69 (171.97, 385.74) | -0.23 (-0.28, 0.17) |
| Rwanda | 1982.59 (1205.15, 3076.60) | 239.31 (158.21, 352.40) | -1.03 (-1.28, -0.77) |
| Saint Kitts and Nevis | 14.25 (8.69, 21.52) | 190.46 (129.91, 271.79) | -0.43 (-0.37, 2.27) |
| Saint Lucia | 43.57 (26.60, 66.03) | 201.38 (137.66, 288.97) | -0.54 (-1.99, 0.93) |
| Saint Vincent and the Grenadines | 27.32 (16.75, 40.80) | 206.54 (140.12, 295.77) | -0.36 (-2.09, 1.40) |
| Samoa | 35.32 (21.00, 53.42) | 256.98 (175.17, 365.60) | -0.37 (-2.00, 1.28) |
| San Marino | 5.99 (3.61, 9.16) | 104.99 (71.38 145.62) | -0.25 (-4.44, 4.03) |
| Sao Tome and Principe | 45.58 (27.92, 69.69) | 341.75 (237.96, 475.28) | -0.98 (-2.57, 0.64) |
| Saudi Arabia | 22494.41 (14658.34, 32500.18) | 464.76 (332.00, 621.94) | -1.69 (-1.76, -1.61) |
| Senegal | 3466.48 (2156.23, 5216.23) | 395.98 (279.42, 547.05) | -1.05 (-1.23, -0.87) |
| Serbia | 1024.55 (610.81, 1582.18) | 162.97 (104.31, 249.56) | -0.17 (-0.46, 0.11) |
| Seychelles | 22.29 (13.57, 33.96) | 312.17 (216.05, 433.44) | -0.55 (-2.53, 1.48) |
| Sierra Leone | 2122.17 (1324.30, 3210.84) | 326.96 (227.12, 461.10) | 0.32 (0.04, 0.61) |
| Singapore | 1446.01 (884.01, 2205.99) | 123.71 (84.46, 173.55) | -0.25 (-0.52, 0.01) |
| Slovakia | 660.03 (393.05, 1025.05) | 160.21 (102.65, 244.84) | -0.17 (-0.53, 0.21) |
| Slovenia | 212.79 (127.52, 333.72) | 154.80 (98.75, 239.17) | -0.18 (-0.82, 0.47) |
| Solomon Islands | 129.05 (78.84, 193.43) | 281.90 (192.49, 398.25) | -0.48 (-1.44, 0.48) |
| Somalia | 4241.56 (2636.72, 6282.52) | 345.22 (238.20, 487.09) | -0.34 (-0.53, -0.14) |
| South Africa | 17796.98 (10838.92, 28316.40) | 354.83 (236.64, 531.95) | -0.67 (-0.75, -0.58) |
| South Sudan | 2168.63 (1357.19, 3282.32) | 386.81 (270.10, 534.66) | -0.40 (-0.61, -0.18) |
| Spain | 15894.55 (9965.46, 23576.37) | 217.73 (152.09, 297.47) | -0.05 (-0.12, 0.02) |
| Sri Lanka | 4775.11 (2864.68, 7453.81) | 313.60 (216.14, 443.57) | -0.57 (-0.71, -0.44) |
| Sudan | 15397.10 (9718.15, 22502.40) | 373.37 (266.50, 503.08) | -0.82 (-0.91, -0.74) |
| Suriname | 169.19 (105.48, 251.97) | 250.96 (174.48, 351.73) | -0.35 (-1.11, 0.42) |
| Sweden | 1390.41 (848.55, 2106.76) | 86.85 (60.59, 120.43) | -0.05 (-0.31, 0.21) |
| Switzerland | 1619.62 (979.50, 2485.09) | 103.54 (70.90, 144.49) | -0.12 (-0.36, 0.13) |
| Syrian Arab Republic | 4925.13 (3099.18, 7306.41) | 331.18 (233.76, 451.03) | -0.61 (-0.74, -0.48) |
| Taiwan (Province of China) | 1629.58 (884.32, 2875.42) | 114.64 (65.18, 199.35) | -0.32 (-0.55, -0.09) |
| Tajikistan | 2313.57 (1430.49, 3428.13) | 300.49 (206.33, 433.11) | -0.24 (-0.47, -0.01) |
| Thailand | 15198.22 (9254.98, 23352.02) | 283.38 (191.69, 410.44) | -0.60 (-0.67, -0.53) |
| Timor-Leste | 435.82 (271.19, 651.63) | 499.07 (352.21, 680.66) | -1.11 (-1.59, -0.63) |
| Togo | 1890.39 (1162.81, 2849.45) | 372.10 (255.78, 518.19) | -0.36 (-0.63, -0.09) |
| Tokelau | 0.20 (0.120837121, 0.31) | 243.03 (165.78, 349.56) | -0.75 (-18.62, 21.04) |
| Tonga | 11.92 (7.13, 18.47) | 189.80 (125.05, 278.63) | -0.64 (-3.30, 2.10) |
| Trinidad and Tobago | 365.24 (224.83, 556.06) | 202.93 (137.47, 296.62) | -0.28 (-0.79, 0.23) |
| Tunisia | 3116.42 (1982.51, 4638.17) | 278.74 (196.61, 377.97) | -0.43 (-0.60, -0.26) |
| Turkey | 19592.56 (12129.02, 29435.15) | 210.68 (145.34, 290.88) | -0.55 (-0.62, -0.48) |
| Turkmenistan | 1288.22 (789.96, 1964.52) | 300.34 (202.34, 432.45) | -0.41 (-0.69, -0.14) |
| Tuvalu | 2.11 (1.28, 3.19) | 258.12 (176.32, 365.63) | -0.66 (-6.96, 6.07) |
| Uganda | 6223.09 (3820.69, 9627.88) | 264.43 (180.05, 382.02) | -0.49 (-0.64, -0.34) |
| Ukraine | 7659.18 (4835.91, 11655.59) | 223.27 (149.49, 325.78) | -0.16 (-0.26, -0.05) |
| United Arab Emirates | 3858.09 (2427.22, 5745.32) | 288.46 (202.19, 392.07) | -0.60 (-0.85, -0.36) |
| United Kingdom | 15013.60 (9293.93, 22790.89) | 121.41 (83.51, 168.22) | -0.07 (-0.15, 0.01) |
| United Republic of Tanzania | 15156.70 (9297.20, 22929.87) | 475.20 (320.91, 673.11) | -0.81 (-0.90, -0.72) |
| United States of America | 49992.56 (30837.30, 76214.44) | 84.14 (57.97, 118.07) | -0.01 (-0.08, 0.07) |
| United States Virgin Islands | 19.73 (12.15, 29.86) | 190.01 (129.17, 272.26) | -0.55 (-2.54, 1.49) |
| Uruguay | 745.76 (449.32, 1146.43) | 120.42 (81.92, 169.39) | -0.14 (-0.48, 0.21) |
| Uzbekistan | 7845.24 (4858.20, 11766.67) | 279.79 (190.23, 398.58) | -0.39 (-0.51, -0.28) |
| Vanuatu | 43.28 (26.16, 67.20) | 225.34 (152.21, 323.75) | -0.52 (-2.20, 1.18) |
| Venezuela (Bolivarian Republic of) | 9070.08 (5700.27, 13493.32) | 279.50 (193.36, 391.20) | -0.29 (-0.39, -0.19) |
| Viet Nam | 24914.57 (15390.26, 37715.46) | 308.71 (215.49, 434.28) | -0.66 (-0.77, -0.55) |
| Yemen | 9971.04 (6243.77, 14820.59) | 335.54 (237.99, 452.86) | -0.48 (-0.61, -0.36) |
| Zambia | 3055.65 (1865.08, 4680.73) | 295.70 (201.80, 421.57) | -1.00 (-1.21, -0.79) |
| Zimbabwe | 4726.84 (2880.77, 7390.96) | 420.00 (286.97, 620.24) | -0.13 (-0.29, 0.03) |

**Abbreviations:** DALYs, Disability adjusted life years.

**Table S2 The *local drift* of DALYs from 1990 to 2019 for blindness and vision loss in teenagers and young adults for five age groups across SDI quintiles**

| **Location** | **Age** | ***Local drift* (%/year)** |
| --- | --- | --- |
| Global | 15 to 19 | -0.49 (-0.52, -0.46) |
| Global | 20 to 24 | -0.46 (-0.48, -0.43) |
| Global | 25 to 29 | -0.44 (-0.46, -0.42) |
| Global | 30 to 34 | -0.41 (-0.43, -0.39) |
| Global | 35 to 39 | -0.35 (-0.38, -0.33) |
| High SDI | 15 to 19 | -0.11 (-0.18, -0.04) |
| High SDI | 20 to 24 | -0.04 (-0.09, 0.01) |
| High SDI | 25 to 29 | -0.01 (-0.06, 0.03) |
| High SDI | 30 to 34 | 0.01 (-0.04, 0.05) |
| High SDI | 35 to 39 | -0.004 (-0.06, 0.06) |
| High-middle SDI | 15 to 19 | -0.12 (-0.18, -0.05) |
| High-middle SDI | 20 to 24 | -0.29 (-0.34, -0.24) |
| High-middle SDI | 25 to 29 | -0.44 (-0.49, -0.40) |
| High-middle SDI | 30 to 34 | -0.43 (-0.48, -0.39) |
| High-middle SDI | 35 to 39 | -0.35 (-0.40, -0.30) |
| Middle SDI | 15 to 19 | -0.35 (-0.38, -0.32) |
| Middle SDI | 20 to 24 | -0.34 (-0.36, -0.32) |
| Middle SDI | 25 to 29 | -0.36 (-0.38, -0.34) |
| Middle SDI | 30 to 34 | -0.46 (-0.48, -0.44) |
| Middle SDI | 35 to 39 | -0.51 (-0.53, -0.48) |
| Low-middle SDI | 15 to 19 | -0.89 (-0.93, -0.86) |
| Low-middle SDI | 20 to 24 | -0.90 (-0.92, -0.87) |
| Low-middle SDI | 25 to 29 | -0.90 (-0.92, -0.87) |
| Low-middle SDI | 30 to 34 | -0.94 (-0.96, -0.92) |
| Low-middle SDI | 35 to 39 | -0.97 (-1.00, -0.95) |
| Low SDI | 15 to 19 | -0.58 (-0.62, -0.54) |
| Low SDI | 20 to 24 | -0.63 (-0.67, -0.60) |
| Low SDI | 25 to 29 | -0.66 (-0.69, -0.63) |
| Low SDI | 30 to 34 | -0.64 (-0.66, -0.61) |
| Low SDI | 35 to 39 | -0.59 (-0.62, -0.55) |

**Abbreviations:** DALYs, Disability adjusted life years; SDI, sociodemographic index.

**Table S3 The *local drift* of DALYs from 1990 to 2019 for blindness and vision loss in teenagers and young adults for five age groups across countries**

| **Location** | **Age** | ***Local drift* (%/year)** |
| --- | --- | --- |
| Afghanistan | 15 to 19 | -0.44 (-0.6, -0.29) |
| Afghanistan | 20 to 24 | -0.43 (-0.55, -0.3) |
| Afghanistan | 25 to 29 | -0.42 (-0.55, -0.28) |
| Afghanistan | 30 to 34 | -0.42 (-0.6, -0.25) |
| Afghanistan | 35 to 39 | -0.43 (-0.68, -0.17) |
| Albania | 15 to 19 | -0.13 (-1.09, 0.84) |
| Albania | 20 to 24 | -0.19 (-0.9, 0.52) |
| Albania | 25 to 29 | -0.23 (-0.87, 0.41) |
| Albania | 30 to 34 | -0.27 (-0.9, 0.37) |
| Albania | 35 to 39 | -0.3 (-1.05, 0.46) |
| Algeria | 15 to 19 | -0.56 (-0.72, -0.4) |
| Algeria | 20 to 24 | -0.6 (-0.72, -0.48) |
| Algeria | 25 to 29 | -0.65 (-0.76, -0.54) |
| Algeria | 30 to 34 | -0.7 (-0.83, -0.58) |
| Algeria | 35 to 39 | -0.75 (-0.92, -0.58) |
| American Samoa | 15 to 19 | -0.85 (-6.42, 5.05) |
| American Samoa | 20 to 24 | -1.12 (-5.56, 3.52) |
| American Samoa | 25 to 29 | -1.39 (-5.91, 3.35) |
| American Samoa | 30 to 34 | -1.25 (-5.77, 3.49) |
| American Samoa | 35 to 39 | 0.12 (-5.36, 5.91) |
| Andorra | 15 to 19 | -0.44 (-5.8, 5.23) |
| Andorra | 20 to 24 | -0.23 (-4.18, 3.88) |
| Andorra | 25 to 29 | 0.13 (-3.15, 3.53) |
| Andorra | 30 to 34 | 0.1 (-3.14, 3.45) |
| Andorra | 35 to 39 | -0.55 (-4.52, 3.59) |
| Angola | 15 to 19 | -0.9 (-1.22, -0.59) |
| Angola | 20 to 24 | -0.93 (-1.18, -0.67) |
| Angola | 25 to 29 | -0.92 (-1.15, -0.7) |
| Angola | 30 to 34 | -0.91 (-1.13, -0.69) |
| Angola | 35 to 39 | -0.89 (-1.17, -0.62) |
| Antigua and Barbuda | 15 to 19 | -0.38 (-4.8, 4.25) |
| Antigua and Barbuda | 20 to 24 | -0.26 (-3.55, 3.13) |
| Antigua and Barbuda | 25 to 29 | -0.45 (-3.29, 2.47) |
| Antigua and Barbuda | 30 to 34 | -0.53 (-3.35, 2.36) |
| Antigua and Barbuda | 35 to 39 | -0.24 (-3.93, 3.6) |
| Argentina | 15 to 19 | -0.21 (-0.38, -0.05) |
| Argentina | 20 to 24 | -0.22 (-0.35, -0.09) |
| Argentina | 25 to 29 | -0.25 (-0.38, -0.13) |
| Argentina | 30 to 34 | -0.28 (-0.42, -0.15) |
| Argentina | 35 to 39 | -0.32 (-0.49, -0.14) |
| Armenia | 15 to 19 | -0.3 (-1.13, 0.53) |
| Armenia | 20 to 24 | -0.34 (-0.93, 0.24) |
| Armenia | 25 to 29 | -0.38 (-0.87, 0.11) |
| Armenia | 30 to 34 | -0.42 (-0.88, 0.05) |
| Armenia | 35 to 39 | -0.43 (-0.99, 0.13) |
| Australia | 15 to 19 | -0.07 (-0.36, 0.22) |
| Australia | 20 to 24 | -0.09 (-0.32, 0.13) |
| Australia | 25 to 29 | -0.1 (-0.31, 0.1) |
| Australia | 30 to 34 | -0.14 (-0.35, 0.08) |
| Australia | 35 to 39 | -0.18 (-0.46, 0.1) |
| Austria | 15 to 19 | -0.11 (-0.61, 0.39) |
| Austria | 20 to 24 | -0.12 (-0.47, 0.24) |
| Austria | 25 to 29 | -0.15 (-0.46, 0.15) |
| Austria | 30 to 34 | -0.2 (-0.51, 0.12) |
| Austria | 35 to 39 | -0.22 (-0.64, 0.2) |
| Azerbaijan | 15 to 19 | -0.32 (-0.76, 0.12) |
| Azerbaijan | 20 to 24 | -0.39 (-0.7, -0.07) |
| Azerbaijan | 25 to 29 | -0.43 (-0.7, -0.16) |
| Azerbaijan | 30 to 34 | -0.45 (-0.72, -0.18) |
| Azerbaijan | 35 to 39 | -0.46 (-0.81, -0.12) |
| Bahamas | 15 to 19 | 0.01 (-2.13, 2.2) |
| Bahamas | 20 to 24 | -0.22 (-1.82, 1.4) |
| Bahamas | 25 to 29 | -0.28 (-1.68, 1.14) |
| Bahamas | 30 to 34 | -0.28 (-1.69, 1.14) |
| Bahamas | 35 to 39 | -0.39 (-2.16, 1.41) |
| Bahrain | 15 to 19 | -0.63 (-1.69, 0.45) |
| Bahrain | 20 to 24 | -0.66 (-1.49, 0.17) |
| Bahrain | 25 to 29 | -0.75 (-1.44, -0.05) |
| Bahrain | 30 to 34 | -0.78 (-1.44, -0.12) |
| Bahrain | 35 to 39 | -0.84 (-1.73, 0.06) |
| Bangladesh | 15 to 19 | -0.81 (-0.91, -0.71) |
| Bangladesh | 20 to 24 | -0.84 (-0.91, -0.76) |
| Bangladesh | 25 to 29 | -0.82 (-0.88, -0.75) |
| Bangladesh | 30 to 34 | -0.74 (-0.81, -0.68) |
| Bangladesh | 35 to 39 | -0.66 (-0.74, -0.58) |
| Barbados | 15 to 19 | 0.17 (-3.32, 3.79) |
| Barbados | 20 to 24 | -0.01 (-2.65, 2.69) |
| Barbados | 25 to 29 | -0.23 (-2.49, 2.09) |
| Barbados | 30 to 34 | -0.37 (-2.53, 1.84) |
| Barbados | 35 to 39 | -0.38 (-2.92, 2.23) |
| Belarus | 15 to 19 | -0.22 (-0.77, 0.34) |
| Belarus | 20 to 24 | -0.25 (-0.65, 0.14) |
| Belarus | 25 to 29 | -0.27 (-0.59, 0.04) |
| Belarus | 30 to 34 | -0.28 (-0.57, 0) |
| Belarus | 35 to 39 | -0.29 (-0.62, 0.05) |
| Belgium | 15 to 19 | -0.16 (-0.59, 0.27) |
| Belgium | 20 to 24 | -0.17 (-0.48, 0.15) |
| Belgium | 25 to 29 | -0.19 (-0.47, 0.09) |
| Belgium | 30 to 34 | -0.22 (-0.51, 0.07) |
| Belgium | 35 to 39 | -0.25 (-0.61, 0.12) |
| Belize | 15 to 19 | -0.44 (-2.42, 1.58) |
| Belize | 20 to 24 | -0.38 (-1.98, 1.24) |
| Belize | 25 to 29 | -0.51 (-2, 1) |
| Belize | 30 to 34 | -0.54 (-2.12, 1.07) |
| Belize | 35 to 39 | -0.5 (-2.62, 1.66) |
| Benin | 15 to 19 | -0.58 (-1.02, -0.14) |
| Benin | 20 to 24 | -0.39 (-0.74, -0.04) |
| Benin | 25 to 29 | -0.03 (-0.35, 0.3) |
| Benin | 30 to 34 | 0.48 (0.14, 0.82) |
| Benin | 35 to 39 | 1.03 (0.57, 1.5) |
| Bermuda | 15 to 19 | -1.46 (-7.97, 5.52) |
| Bermuda | 20 to 24 | 0 (-4.07, 4.24) |
| Bermuda | 25 to 29 | -0.44 (-3.9, 3.13) |
| Bermuda | 30 to 34 | -0.83 (-3.98, 2.42) |
| Bermuda | 35 to 39 | -0.45 (-4.26, 3.51) |
| Bhutan | 15 to 19 | -0.72 (-2.41, 1) |
| Bhutan | 20 to 24 | -0.71 (-1.97, 0.56) |
| Bhutan | 25 to 29 | -0.71 (-1.81, 0.4) |
| Bhutan | 30 to 34 | -0.76 (-1.8, 0.28) |
| Bhutan | 35 to 39 | -0.79 (-2, 0.44) |
| Bolivia (Plurinational State of) | 15 to 19 | -0.44 (-0.74, -0.14) |
| Bolivia (Plurinational State of) | 20 to 24 | -0.47 (-0.7, -0.23) |
| Bolivia (Plurinational State of) | 25 to 29 | -0.48 (-0.7, -0.26) |
| Bolivia (Plurinational State of) | 30 to 34 | -0.5 (-0.73, -0.27) |
| Bolivia (Plurinational State of) | 35 to 39 | -0.52 (-0.82, -0.22) |
| Bosnia and Herzegovina | 15 to 19 | -0.15 (-1.12, 0.83) |
| Bosnia and Herzegovina | 20 to 24 | -0.21 (-0.92, 0.51) |
| Bosnia and Herzegovina | 25 to 29 | -0.25 (-0.86, 0.36) |
| Bosnia and Herzegovina | 30 to 34 | -0.34 (-0.9, 0.23) |
| Bosnia and Herzegovina | 35 to 39 | -0.41 (-1.05, 0.23) |
| Botswana | 15 to 19 | -0.86 (-1.77, 0.06) |
| Botswana | 20 to 24 | -0.88 (-1.56, -0.2) |
| Botswana | 25 to 29 | -0.87 (-1.44, -0.29) |
| Botswana | 30 to 34 | -0.84 (-1.36, -0.31) |
| Botswana | 35 to 39 | -0.79 (-1.42, -0.16) |
| Brazil | 15 to 19 | -0.23 (-0.29, -0.17) |
| Brazil | 20 to 24 | -0.22 (-0.26, -0.17) |
| Brazil | 25 to 29 | -0.21 (-0.25, -0.17) |
| Brazil | 30 to 34 | -0.24 (-0.28, -0.19) |
| Brazil | 35 to 39 | -0.32 (-0.37, -0.26) |
| Brunei Darussalam | 15 to 19 | -0.23 (-2.06, 1.63) |
| Brunei Darussalam | 20 to 24 | -0.25 (-1.66, 1.17) |
| Brunei Darussalam | 25 to 29 | -0.16 (-1.46, 1.16) |
| Brunei Darussalam | 30 to 34 | -0.09 (-1.51, 1.35) |
| Brunei Darussalam | 35 to 39 | -0.11 (-2.07, 1.89) |
| Bulgaria | 15 to 19 | -0.13 (-0.9, 0.65) |
| Bulgaria | 20 to 24 | -0.15 (-0.71, 0.41) |
| Bulgaria | 25 to 29 | -0.18 (-0.63, 0.28) |
| Bulgaria | 30 to 34 | -0.2 (-0.62, 0.22) |
| Bulgaria | 35 to 39 | -0.22 (-0.69, 0.26) |
| Burkina Faso | 15 to 19 | -0.55 (-0.88, -0.23) |
| Burkina Faso | 20 to 24 | -0.44 (-0.7, -0.18) |
| Burkina Faso | 25 to 29 | -0.25 (-0.49, -0.01) |
| Burkina Faso | 30 to 34 | 0.12 (-0.13, 0.37) |
| Burkina Faso | 35 to 39 | 0.76 (0.43, 1.09) |
| Burundi | 15 to 19 | -0.77 (-1.39, -0.15) |
| Burundi | 20 to 24 | -0.76 (-1.26, -0.26) |
| Burundi | 25 to 29 | -0.68 (-1.11, -0.24) |
| Burundi | 30 to 34 | -0.58 (-0.98, -0.17) |
| Burundi | 35 to 39 | -0.47 (-0.96, 0.02) |
| Cabo Verde | 15 to 19 | -0.63 (-2.8, 1.58) |
| Cabo Verde | 20 to 24 | -0.73 (-2.34, 0.91) |
| Cabo Verde | 25 to 29 | -0.85 (-2.23, 0.54) |
| Cabo Verde | 30 to 34 | -0.88 (-2.22, 0.47) |
| Cabo Verde | 35 to 39 | -0.94 (-2.69, 0.84) |
| Cambodia | 15 to 19 | -1.83 (-2.07, -1.6) |
| Cambodia | 20 to 24 | -1.84 (-2.03, -1.66) |
| Cambodia | 25 to 29 | -1.79 (-1.96, -1.62) |
| Cambodia | 30 to 34 | -1.76 (-1.94, -1.58) |
| Cambodia | 35 to 39 | -1.75 (-1.98, -1.52) |
| Cameroon | 15 to 19 | -0.99 (-1.29, -0.68) |
| Cameroon | 20 to 24 | -0.69 (-0.94, -0.44) |
| Cameroon | 25 to 29 | -0.3 (-0.53, -0.07) |
| Cameroon | 30 to 34 | 0.03 (-0.21, 0.28) |
| Cameroon | 35 to 39 | 0.26 (-0.06, 0.58) |
| Canada | 15 to 19 | -0.08 (-0.36, 0.19) |
| Canada | 20 to 24 | -0.08 (-0.28, 0.13) |
| Canada | 25 to 29 | -0.09 (-0.27, 0.09) |
| Canada | 30 to 34 | -0.1 (-0.29, 0.09) |
| Canada | 35 to 39 | -0.12 (-0.37, 0.12) |
| Central African Republic | 15 to 19 | 0.54 (-0.14, 1.22) |
| Central African Republic | 20 to 24 | 1.27 (0.7, 1.84) |
| Central African Republic | 25 to 29 | 1.89 (1.33, 2.46) |
| Central African Republic | 30 to 34 | 2.06 (1.44, 2.69) |
| Central African Republic | 35 to 39 | 1.8 (0.97, 2.63) |
| Chad | 15 to 19 | -0.6 (-0.97, -0.23) |
| Chad | 20 to 24 | -0.31 (-0.61, 0) |
| Chad | 25 to 29 | 0.03 (-0.26, 0.32) |
| Chad | 30 to 34 | 0.24 (-0.06, 0.55) |
| Chad | 35 to 39 | 0.27 (-0.12, 0.67) |
| Chile | 15 to 19 | -0.29 (-0.57, -0.02) |
| Chile | 20 to 24 | -0.33 (-0.54, -0.13) |
| Chile | 25 to 29 | -0.38 (-0.57, -0.19) |
| Chile | 30 to 34 | -0.43 (-0.64, -0.22) |
| Chile | 35 to 39 | -0.49 (-0.77, -0.22) |
| China | 15 to 19 | -0.11 (-0.33, 0.11) |
| China | 20 to 24 | -0.46 (-0.62, -0.31) |
| China | 25 to 29 | -0.76 (-0.89, -0.63) |
| China | 30 to 34 | -0.91 (-1.04, -0.78) |
| China | 35 to 39 | -0.88 (-1.03, -0.72) |
| Colombia | 15 to 19 | -0.51 (-0.66, -0.35) |
| Colombia | 20 to 24 | -0.52 (-0.64, -0.41) |
| Colombia | 25 to 29 | -0.54 (-0.64, -0.43) |
| Colombia | 30 to 34 | -0.55 (-0.65, -0.44) |
| Colombia | 35 to 39 | -0.57 (-0.71, -0.44) |
| Comoros | 15 to 19 | -0.87 (-2.71, 1) |
| Comoros | 20 to 24 | -0.97 (-2.37, 0.44) |
| Comoros | 25 to 29 | -1.04 (-2.24, 0.19) |
| Comoros | 30 to 34 | -1.05 (-2.21, 0.12) |
| Comoros | 35 to 39 | -1.01 (-2.39, 0.4) |
| Congo | 15 to 19 | -0.68 (-1.4, 0.05) |
| Congo | 20 to 24 | -0.77 (-1.34, -0.2) |
| Congo | 25 to 29 | -0.82 (-1.32, -0.32) |
| Congo | 30 to 34 | -0.84 (-1.34, -0.34) |
| Congo | 35 to 39 | -0.85 (-1.47, -0.22) |
| Cook Islands | 15 to 19 | -1.61 (-11.13, 8.95) |
| Cook Islands | 20 to 24 | -0.14 (-6.95, 7.16) |
| Cook Islands | 25 to 29 | 0.41 (-5.68, 6.9) |
| Cook Islands | 30 to 34 | 0.78 (-5.37, 7.32) |
| Cook Islands | 35 to 39 | -0.02 (-7.82, 8.43) |
| Costa Rica | 15 to 19 | -0.2 (-0.72, 0.33) |
| Costa Rica | 20 to 24 | -0.21 (-0.59, 0.18) |
| Costa Rica | 25 to 29 | -0.22 (-0.56, 0.12) |
| Costa Rica | 30 to 34 | -0.25 (-0.6, 0.1) |
| Costa Rica | 35 to 39 | -0.3 (-0.75, 0.16) |
| Croatia | 15 to 19 | -0.16 (-1.11, 0.8) |
| Croatia | 20 to 24 | -0.17 (-0.87, 0.54) |
| Croatia | 25 to 29 | -0.19 (-0.78, 0.41) |
| Croatia | 30 to 34 | -0.2 (-0.74, 0.35) |
| Croatia | 35 to 39 | -0.22 (-0.83, 0.4) |
| Cuba | 15 to 19 | -0.15 (-0.49, 0.19) |
| Cuba | 20 to 24 | -0.18 (-0.42, 0.07) |
| Cuba | 25 to 29 | -0.2 (-0.4, 0) |
| Cuba | 30 to 34 | -0.22 (-0.43, -0.02) |
| Cuba | 35 to 39 | -0.26 (-0.52, 0.01) |
| Cyprus | 15 to 19 | -0.27 (-1.55, 1.03) |
| Cyprus | 20 to 24 | -0.26 (-1.19, 0.68) |
| Cyprus | 25 to 29 | -0.25 (-1.07, 0.58) |
| Cyprus | 30 to 34 | -0.29 (-1.15, 0.58) |
| Cyprus | 35 to 39 | -0.34 (-1.5, 0.83) |
| Czechia | 15 to 19 | -0.17 (-0.82, 0.48) |
| Czechia | 20 to 24 | -0.18 (-0.65, 0.29) |
| Czechia | 25 to 29 | -0.2 (-0.59, 0.19) |
| Czechia | 30 to 34 | -0.22 (-0.59, 0.15) |
| Czechia | 35 to 39 | -0.24 (-0.65, 0.17) |
| Côte d'Ivoire | 15 to 19 | -0.51 (-0.83, -0.18) |
| Côte d'Ivoire | 20 to 24 | -0.38 (-0.63, -0.13) |
| Côte d'Ivoire | 25 to 29 | -0.15 (-0.38, 0.07) |
| Côte d'Ivoire | 30 to 34 | 0.24 (0.01, 0.47) |
| Côte d'Ivoire | 35 to 39 | 0.93 (0.62, 1.25) |
| Democratic People's Republic of Korea | 15 to 19 | -0.41 (-0.82, 0.01) |
| Democratic People's Republic of Korea | 20 to 24 | -0.46 (-0.78, -0.15) |
| Democratic People's Republic of Korea | 25 to 29 | -0.5 (-0.78, -0.22) |
| Democratic People's Republic of Korea | 30 to 34 | -0.49 (-0.75, -0.22) |
| Democratic People's Republic of Korea | 35 to 39 | -0.46 (-0.78, -0.15) |
| Democratic Republic of the Congo | 15 to 19 | -0.43 (-0.63, -0.22) |
| Democratic Republic of the Congo | 20 to 24 | -0.32 (-0.48, -0.15) |
| Democratic Republic of the Congo | 25 to 29 | -0.16 (-0.31, -0.01) |
| Democratic Republic of the Congo | 30 to 34 | 0 (-0.16, 0.16) |
| Democratic Republic of the Congo | 35 to 39 | 0.12 (-0.09, 0.33) |
| Denmark | 15 to 19 | -0.11 (-0.7, 0.48) |
| Denmark | 20 to 24 | -0.13 (-0.56, 0.3) |
| Denmark | 25 to 29 | -0.15 (-0.54, 0.24) |
| Denmark | 30 to 34 | -0.18 (-0.6, 0.24) |
| Denmark | 35 to 39 | -0.2 (-0.73, 0.32) |
| Djibouti | 15 to 19 | -1.21 (-2.78, 0.38) |
| Djibouti | 20 to 24 | -1.21 (-2.4, 0) |
| Djibouti | 25 to 29 | -1.18 (-2.2, -0.14) |
| Djibouti | 30 to 34 | -1.11 (-2.09, -0.12) |
| Djibouti | 35 to 39 | -1.01 (-2.2, 0.19) |
| Dominica | 15 to 19 | 0.39 (-4.14, 5.13) |
| Dominica | 20 to 24 | -0.1 (-3.5, 3.42) |
| Dominica | 25 to 29 | -0.25 (-3.3, 2.89) |
| Dominica | 30 to 34 | -0.46 (-3.53, 2.71) |
| Dominica | 35 to 39 | -0.4 (-4.37, 3.73) |
| Dominican Republic | 15 to 19 | -0.48 (-0.8, -0.15) |
| Dominican Republic | 20 to 24 | -0.52 (-0.77, -0.27) |
| Dominican Republic | 25 to 29 | -0.56 (-0.79, -0.34) |
| Dominican Republic | 30 to 34 | -0.6 (-0.84, -0.36) |
| Dominican Republic | 35 to 39 | -0.64 (-0.95, -0.34) |
| Ecuador | 15 to 19 | -0.23 (-0.51, 0.06) |
| Ecuador | 20 to 24 | -0.24 (-0.46, -0.02) |
| Ecuador | 25 to 29 | -0.26 (-0.46, -0.05) |
| Ecuador | 30 to 34 | -0.27 (-0.48, -0.05) |
| Ecuador | 35 to 39 | -0.29 (-0.57, -0.01) |
| Egypt | 15 to 19 | -0.51 (-0.61, -0.42) |
| Egypt | 20 to 24 | -0.56 (-0.64, -0.48) |
| Egypt | 25 to 29 | -0.62 (-0.7, -0.55) |
| Egypt | 30 to 34 | -0.7 (-0.78, -0.62) |
| Egypt | 35 to 39 | -0.76 (-0.88, -0.65) |
| El Salvador | 15 to 19 | -0.45 (-0.81, -0.09) |
| El Salvador | 20 to 24 | -0.44 (-0.71, -0.17) |
| El Salvador | 25 to 29 | -0.45 (-0.7, -0.2) |
| El Salvador | 30 to 34 | -0.49 (-0.76, -0.22) |
| El Salvador | 35 to 39 | -0.57 (-0.91, -0.22) |
| Equatorial Guinea | 15 to 19 | -2.37 (-3.56, -1.17) |
| Equatorial Guinea | 20 to 24 | -2.58 (-3.52, -1.63) |
| Equatorial Guinea | 25 to 29 | -2.78 (-3.62, -1.93) |
| Equatorial Guinea | 30 to 34 | -2.83 (-3.69, -1.97) |
| Equatorial Guinea | 35 to 39 | -2.84 (-3.91, -1.75) |
| Eritrea | 15 to 19 | -1.07 (-1.62, -0.51) |
| Eritrea | 20 to 24 | -1.08 (-1.51, -0.64) |
| Eritrea | 25 to 29 | -1.04 (-1.43, -0.65) |
| Eritrea | 30 to 34 | -0.94 (-1.32, -0.57) |
| Eritrea | 35 to 39 | -0.8 (-1.26, -0.33) |
| Estonia | 15 to 19 | -0.2 (-2.02, 1.65) |
| Estonia | 20 to 24 | -0.15 (-1.44, 1.16) |
| Estonia | 25 to 29 | -0.16 (-1.2, 0.89) |
| Estonia | 30 to 34 | -0.19 (-1.14, 0.77) |
| Estonia | 35 to 39 | -0.28 (-1.35, 0.8) |
| Eswatini | 15 to 19 | -0.64 (-1.8, 0.53) |
| Eswatini | 20 to 24 | -0.62 (-1.5, 0.27) |
| Eswatini | 25 to 29 | -0.6 (-1.36, 0.18) |
| Eswatini | 30 to 34 | -0.6 (-1.34, 0.14) |
| Eswatini | 35 to 39 | -0.6 (-1.49, 0.3) |
| Ethiopia | 15 to 19 | 0.52 (0.39, 0.65) |
| Ethiopia | 20 to 24 | 0.23 (0.13, 0.33) |
| Ethiopia | 25 to 29 | -0.06 (-0.15, 0.03) |
| Ethiopia | 30 to 34 | -0.26 (-0.35, -0.17) |
| Ethiopia | 35 to 39 | -0.39 (-0.5, -0.28) |
| Fiji | 15 to 19 | -0.55 (-2.01, 0.93) |
| Fiji | 20 to 24 | -0.59 (-1.7, 0.53) |
| Fiji | 25 to 29 | -0.55 (-1.53, 0.43) |
| Fiji | 30 to 34 | -0.47 (-1.42, 0.49) |
| Fiji | 35 to 39 | -0.25 (-1.4, 0.91) |
| Finland | 15 to 19 | -0.17 (-0.78, 0.45) |
| Finland | 20 to 24 | -0.18 (-0.63, 0.28) |
| Finland | 25 to 29 | -0.19 (-0.59, 0.21) |
| Finland | 30 to 34 | -0.22 (-0.63, 0.2) |
| Finland | 35 to 39 | -0.24 (-0.75, 0.28) |
| France | 15 to 19 | -0.11 (-0.29, 0.06) |
| France | 20 to 24 | -0.13 (-0.26, 0) |
| France | 25 to 29 | -0.15 (-0.28, -0.03) |
| France | 30 to 34 | -0.17 (-0.3, -0.04) |
| France | 35 to 39 | -0.18 (-0.34, -0.02) |
| Gabon | 15 to 19 | -0.67 (-1.93, 0.6) |
| Gabon | 20 to 24 | -0.79 (-1.76, 0.18) |
| Gabon | 25 to 29 | -0.89 (-1.74, -0.04) |
| Gabon | 30 to 34 | -0.9 (-1.74, -0.06) |
| Gabon | 35 to 39 | -0.88 (-1.91, 0.16) |
| Gambia | 15 to 19 | -1.34 (-2.29, -0.38) |
| Gambia | 20 to 24 | -1.3 (-2.05, -0.56) |
| Gambia | 25 to 29 | -1.23 (-1.9, -0.57) |
| Gambia | 30 to 34 | -1.13 (-1.79, -0.45) |
| Gambia | 35 to 39 | -0.97 (-1.82, -0.11) |
| Georgia | 15 to 19 | -3.05 (-3.76, -2.32) |
| Georgia | 20 to 24 | -2.25 (-2.77, -1.73) |
| Georgia | 25 to 29 | -1.8 (-2.23, -1.36) |
| Georgia | 30 to 34 | -1.79 (-2.2, -1.37) |
| Georgia | 35 to 39 | -1.88 (-2.37, -1.4) |
| Germany | 15 to 19 | -0.1 (-0.26, 0.07) |
| Germany | 20 to 24 | -0.11 (-0.23, 0.01) |
| Germany | 25 to 29 | -0.14 (-0.24, -0.04) |
| Germany | 30 to 34 | -0.17 (-0.28, -0.07) |
| Germany | 35 to 39 | -0.2 (-0.33, -0.06) |
| Ghana | 15 to 19 | -0.31 (-0.57, -0.05) |
| Ghana | 20 to 24 | -0.3 (-0.51, -0.1) |
| Ghana | 25 to 29 | -0.19 (-0.38, 0) |
| Ghana | 30 to 34 | 0.04 (-0.16, 0.23) |
| Ghana | 35 to 39 | 0.39 (0.14, 0.64) |
| Greece | 15 to 19 | -0.25 (-0.72, 0.23) |
| Greece | 20 to 24 | -0.25 (-0.59, 0.1) |
| Greece | 25 to 29 | -0.27 (-0.57, 0.03) |
| Greece | 30 to 34 | -0.3 (-0.61, 0) |
| Greece | 35 to 39 | -0.33 (-0.71, 0.05) |
| Greenland | 15 to 19 | -0.61 (-6.12, 5.23) |
| Greenland | 20 to 24 | -0.16 (-4.46, 4.33) |
| Greenland | 25 to 29 | 0.13 (-3.69, 4.1) |
| Greenland | 30 to 34 | 0.31 (-3.65, 4.43) |
| Greenland | 35 to 39 | 0.86 (-4.65, 6.69) |
| Grenada | 15 to 19 | -0.54 (-4.24, 3.31) |
| Grenada | 20 to 24 | -0.44 (-3.15, 2.34) |
| Grenada | 25 to 29 | -0.26 (-2.79, 2.34) |
| Grenada | 30 to 34 | -0.46 (-3.12, 2.28) |
| Grenada | 35 to 39 | -0.2 (-3.63, 3.36) |
| Guam | 15 to 19 | -0.42 (-4.29, 3.6) |
| Guam | 20 to 24 | -0.29 (-3.19, 2.7) |
| Guam | 25 to 29 | -0.24 (-2.86, 2.46) |
| Guam | 30 to 34 | -0.14 (-2.68, 2.46) |
| Guam | 35 to 39 | -0.26 (-3.24, 2.81) |
| Guatemala | 15 to 19 | -0.46 (-0.68, -0.23) |
| Guatemala | 20 to 24 | -0.42 (-0.6, -0.24) |
| Guatemala | 25 to 29 | -0.37 (-0.54, -0.19) |
| Guatemala | 30 to 34 | -0.32 (-0.51, -0.13) |
| Guatemala | 35 to 39 | -0.31 (-0.56, -0.06) |
| Guinea | 15 to 19 | -0.8 (-1.19, -0.41) |
| Guinea | 20 to 24 | -0.67 (-0.97, -0.36) |
| Guinea | 25 to 29 | -0.46 (-0.74, -0.18) |
| Guinea | 30 to 34 | -0.15 (-0.44, 0.13) |
| Guinea | 35 to 39 | 0.22 (-0.15, 0.59) |
| Guinea-Bissau | 15 to 19 | -0.96 (-1.95, 0.05) |
| Guinea-Bissau | 20 to 24 | -0.84 (-1.61, -0.07) |
| Guinea-Bissau | 25 to 29 | -0.7 (-1.4, 0) |
| Guinea-Bissau | 30 to 34 | -0.51 (-1.21, 0.2) |
| Guinea-Bissau | 35 to 39 | -0.28 (-1.17, 0.63) |
| Guyana | 15 to 19 | -0.27 (-1.56, 1.03) |
| Guyana | 20 to 24 | -0.27 (-1.22, 0.69) |
| Guyana | 25 to 29 | -0.31 (-1.2, 0.59) |
| Guyana | 30 to 34 | -0.32 (-1.24, 0.61) |
| Guyana | 35 to 39 | -0.38 (-1.51, 0.77) |
| Haiti | 15 to 19 | -0.4 (-0.74, -0.06) |
| Haiti | 20 to 24 | -0.4 (-0.66, -0.13) |
| Haiti | 25 to 29 | -0.41 (-0.65, -0.16) |
| Haiti | 30 to 34 | -0.42 (-0.67, -0.17) |
| Haiti | 35 to 39 | -0.45 (-0.78, -0.13) |
| Honduras | 15 to 19 | -0.34 (-0.68, 0) |
| Honduras | 20 to 24 | -0.35 (-0.62, -0.08) |
| Honduras | 25 to 29 | -0.37 (-0.63, -0.11) |
| Honduras | 30 to 34 | -0.41 (-0.7, -0.12) |
| Honduras | 35 to 39 | -0.45 (-0.83, -0.07) |
| Hungary | 15 to 19 | -0.13 (-0.76, 0.5) |
| Hungary | 20 to 24 | -0.16 (-0.62, 0.3) |
| Hungary | 25 to 29 | -0.18 (-0.58, 0.22) |
| Hungary | 30 to 34 | -0.21 (-0.58, 0.16) |
| Hungary | 35 to 39 | -0.27 (-0.66, 0.13) |
| Iceland | 15 to 19 | -0.21 (-2.58, 2.22) |
| Iceland | 20 to 24 | -0.41 (-2.18, 1.4) |
| Iceland | 25 to 29 | -0.36 (-1.96, 1.27) |
| Iceland | 30 to 34 | -0.3 (-1.99, 1.43) |
| Iceland | 35 to 39 | -0.36 (-2.5, 1.82) |
| India | 15 to 19 | -1.25 (-1.31, -1.19) |
| India | 20 to 24 | -1.34 (-1.38, -1.29) |
| India | 25 to 29 | -1.35 (-1.39, -1.32) |
| India | 30 to 34 | -1.31 (-1.35, -1.27) |
| India | 35 to 39 | -1.25 (-1.29, -1.2) |
| Indonesia | 15 to 19 | -0.86 (-0.92, -0.79) |
| Indonesia | 20 to 24 | -0.93 (-0.98, -0.88) |
| Indonesia | 25 to 29 | -0.97 (-1.01, -0.93) |
| Indonesia | 30 to 34 | -0.97 (-1.01, -0.92) |
| Indonesia | 35 to 39 | -0.95 (-0.99, -0.9) |
| Iran (Islamic Republic of) | 15 to 19 | -0.3 (-0.4, -0.2) |
| Iran (Islamic Republic of) | 20 to 24 | -0.34 (-0.41, -0.27) |
| Iran (Islamic Republic of) | 25 to 29 | -0.38 (-0.45, -0.31) |
| Iran (Islamic Republic of) | 30 to 34 | -0.46 (-0.54, -0.39) |
| Iran (Islamic Republic of) | 35 to 39 | -0.58 (-0.69, -0.48) |
| Iraq | 15 to 19 | -0.59 (-0.75, -0.43) |
| Iraq | 20 to 24 | -0.64 (-0.76, -0.51) |
| Iraq | 25 to 29 | -0.69 (-0.82, -0.56) |
| Iraq | 30 to 34 | -0.74 (-0.89, -0.6) |
| Iraq | 35 to 39 | -0.78 (-0.98, -0.57) |
| Ireland | 15 to 19 | -0.21 (-0.82, 0.4) |
| Ireland | 20 to 24 | -0.23 (-0.7, 0.25) |
| Ireland | 25 to 29 | -0.26 (-0.7, 0.17) |
| Ireland | 30 to 34 | -0.29 (-0.74, 0.16) |
| Ireland | 35 to 39 | -0.31 (-0.89, 0.27) |
| Israel | 15 to 19 | -0.14 (-0.58, 0.3) |
| Israel | 20 to 24 | -0.15 (-0.5, 0.2) |
| Israel | 25 to 29 | -0.17 (-0.51, 0.17) |
| Israel | 30 to 34 | -0.2 (-0.57, 0.16) |
| Israel | 35 to 39 | -0.24 (-0.72, 0.24) |
| Italy | 15 to 19 | -0.3 (-0.45, -0.14) |
| Italy | 20 to 24 | -0.3 (-0.41, -0.19) |
| Italy | 25 to 29 | -0.31 (-0.41, -0.21) |
| Italy | 30 to 34 | -0.34 (-0.44, -0.24) |
| Italy | 35 to 39 | -0.4 (-0.52, -0.28) |
| Jamaica | 15 to 19 | -0.33 (-1.06, 0.4) |
| Jamaica | 20 to 24 | -0.37 (-0.9, 0.17) |
| Jamaica | 25 to 29 | -0.4 (-0.89, 0.09) |
| Jamaica | 30 to 34 | -0.44 (-0.95, 0.07) |
| Jamaica | 35 to 39 | -0.53 (-1.19, 0.13) |
| Japan | 15 to 19 | -0.19 (-0.33, -0.05) |
| Japan | 20 to 24 | -0.2 (-0.31, -0.1) |
| Japan | 25 to 29 | -0.22 (-0.32, -0.13) |
| Japan | 30 to 34 | -0.26 (-0.36, -0.17) |
| Japan | 35 to 39 | -0.31 (-0.43, -0.19) |
| Jordan | 15 to 19 | -0.51 (-0.89, -0.13) |
| Jordan | 20 to 24 | -0.56 (-0.87, -0.25) |
| Jordan | 25 to 29 | -0.62 (-0.93, -0.31) |
| Jordan | 30 to 34 | -0.67 (-1.04, -0.31) |
| Jordan | 35 to 39 | -0.73 (-1.25, -0.21) |
| Kazakhstan | 15 to 19 | -0.25 (-0.58, 0.08) |
| Kazakhstan | 20 to 24 | -0.31 (-0.56, -0.07) |
| Kazakhstan | 25 to 29 | -0.36 (-0.57, -0.15) |
| Kazakhstan | 30 to 34 | -0.39 (-0.59, -0.19) |
| Kazakhstan | 35 to 39 | -0.41 (-0.66, -0.16) |
| Kenya | 15 to 19 | -1.04 (-1.24, -0.85) |
| Kenya | 20 to 24 | -1.14 (-1.3, -0.99) |
| Kenya | 25 to 29 | -1.25 (-1.38, -1.11) |
| Kenya | 30 to 34 | -1.31 (-1.45, -1.17) |
| Kenya | 35 to 39 | -1.33 (-1.51, -1.16) |
| Kiribati | 15 to 19 | -0.37 (-4.49, 3.92) |
| Kiribati | 20 to 24 | -0.39 (-3.62, 2.94) |
| Kiribati | 25 to 29 | -0.31 (-3.2, 2.66) |
| Kiribati | 30 to 34 | -0.45 (-3.34, 2.53) |
| Kiribati | 35 to 39 | -0.24 (-3.96, 3.64) |
| Kuwait | 15 to 19 | -0.44 (-1.13, 0.25) |
| Kuwait | 20 to 24 | -0.45 (-0.95, 0.05) |
| Kuwait | 25 to 29 | -0.47 (-0.89, -0.06) |
| Kuwait | 30 to 34 | -0.53 (-0.94, -0.12) |
| Kuwait | 35 to 39 | -0.59 (-1.14, -0.04) |
| Kyrgyzstan | 15 to 19 | -0.23 (-0.83, 0.37) |
| Kyrgyzstan | 20 to 24 | -0.27 (-0.72, 0.18) |
| Kyrgyzstan | 25 to 29 | -0.3 (-0.7, 0.11) |
| Kyrgyzstan | 30 to 34 | -0.31 (-0.71, 0.09) |
| Kyrgyzstan | 35 to 39 | -0.31 (-0.81, 0.2) |
| Lao People's Democratic Republic | 15 to 19 | -0.66 (-1.16, -0.14) |
| Lao People's Democratic Republic | 20 to 24 | -0.8 (-1.22, -0.38) |
| Lao People's Democratic Republic | 25 to 29 | -0.88 (-1.28, -0.48) |
| Lao People's Democratic Republic | 30 to 34 | -0.89 (-1.3, -0.47) |
| Lao People's Democratic Republic | 35 to 39 | -0.83 (-1.35, -0.3) |
| Latvia | 15 to 19 | -0.17 (-1.4, 1.07) |
| Latvia | 20 to 24 | -0.24 (-1.09, 0.63) |
| Latvia | 25 to 29 | -0.26 (-0.95, 0.44) |
| Latvia | 30 to 34 | -0.29 (-0.93, 0.36) |
| Latvia | 35 to 39 | -0.3 (-1.04, 0.45) |
| Lebanon | 15 to 19 | -0.97 (-1.43, -0.5) |
| Lebanon | 20 to 24 | -0.91 (-1.25, -0.56) |
| Lebanon | 25 to 29 | -0.88 (-1.2, -0.56) |
| Lebanon | 30 to 34 | -0.91 (-1.26, -0.55) |
| Lebanon | 35 to 39 | -0.96 (-1.45, -0.48) |
| Lesotho | 15 to 19 | -0.55 (-1.37, 0.28) |
| Lesotho | 20 to 24 | -0.53 (-1.14, 0.09) |
| Lesotho | 25 to 29 | -0.52 (-1.04, 0.01) |
| Lesotho | 30 to 34 | -0.49 (-0.98, 0) |
| Lesotho | 35 to 39 | -0.48 (-1.06, 0.11) |
| Liberia | 15 to 19 | -1.02 (-1.69, -0.35) |
| Liberia | 20 to 24 | -0.94 (-1.48, -0.4) |
| Liberia | 25 to 29 | -0.79 (-1.3, -0.29) |
| Liberia | 30 to 34 | -0.64 (-1.14, -0.15) |
| Liberia | 35 to 39 | -0.48 (-1.09, 0.13) |
| Libya | 15 to 19 | -0.55 (-0.94, -0.17) |
| Libya | 20 to 24 | -0.58 (-0.88, -0.28) |
| Libya | 25 to 29 | -0.61 (-0.9, -0.32) |
| Libya | 30 to 34 | -0.64 (-0.96, -0.32) |
| Libya | 35 to 39 | -0.66 (-1.1, -0.22) |
| Lithuania | 15 to 19 | -0.17 (-1.12, 0.78) |
| Lithuania | 20 to 24 | -0.22 (-0.89, 0.45) |
| Lithuania | 25 to 29 | -0.23 (-0.8, 0.34) |
| Lithuania | 30 to 34 | -0.26 (-0.8, 0.29) |
| Lithuania | 35 to 39 | -0.26 (-0.88, 0.37) |
| Luxembourg | 15 to 19 | -0.15 (-2.16, 1.89) |
| Luxembourg | 20 to 24 | -0.14 (-1.61, 1.35) |
| Luxembourg | 25 to 29 | -0.22 (-1.49, 1.06) |
| Luxembourg | 30 to 34 | -0.25 (-1.54, 1.06) |
| Luxembourg | 35 to 39 | -0.18 (-1.86, 1.53) |
| Madagascar | 15 to 19 | -0.74 (-1.08, -0.4) |
| Madagascar | 20 to 24 | -0.78 (-1.04, -0.51) |
| Madagascar | 25 to 29 | -0.76 (-1, -0.52) |
| Madagascar | 30 to 34 | -0.72 (-0.95, -0.49) |
| Madagascar | 35 to 39 | -0.67 (-0.95, -0.39) |
| Malawi | 15 to 19 | -0.86 (-1.2, -0.52) |
| Malawi | 20 to 24 | -0.88 (-1.15, -0.6) |
| Malawi | 25 to 29 | -0.85 (-1.09, -0.61) |
| Malawi | 30 to 34 | -0.81 (-1.05, -0.58) |
| Malawi | 35 to 39 | -0.81 (-1.09, -0.52) |
| Malaysia | 15 to 19 | -0.57 (-0.79, -0.35) |
| Malaysia | 20 to 24 | -0.86 (-1.03, -0.68) |
| Malaysia | 25 to 29 | -1.05 (-1.2, -0.89) |
| Malaysia | 30 to 34 | -1.14 (-1.3, -0.98) |
| Malaysia | 35 to 39 | -1.14 (-1.34, -0.95) |
| Maldives | 15 to 19 | -0.53 (-2.66, 1.64) |
| Maldives | 20 to 24 | -0.79 (-2.44, 0.88) |
| Maldives | 25 to 29 | -0.95 (-2.48, 0.59) |
| Maldives | 30 to 34 | -1.19 (-2.81, 0.45) |
| Maldives | 35 to 39 | -1.27 (-3.43, 0.93) |
| Mali | 15 to 19 | -1.15 (-1.43, -0.87) |
| Mali | 20 to 24 | -1.05 (-1.28, -0.83) |
| Mali | 25 to 29 | -0.92 (-1.12, -0.71) |
| Mali | 30 to 34 | -0.7 (-0.91, -0.49) |
| Mali | 35 to 39 | -0.41 (-0.68, -0.13) |
| Malta | 15 to 19 | -0.08 (-2.22, 2.11) |
| Malta | 20 to 24 | -0.05 (-1.61, 1.54) |
| Malta | 25 to 29 | -0.09 (-1.49, 1.33) |
| Malta | 30 to 34 | -0.23 (-1.66, 1.21) |
| Malta | 35 to 39 | -0.36 (-2.15, 1.46) |
| Marshall Islands | 15 to 19 | 0.1 (-5.42, 5.94) |
| Marshall Islands | 20 to 24 | -0.49 (-4.77, 3.97) |
| Marshall Islands | 25 to 29 | -0.98 (-4.84, 3.04) |
| Marshall Islands | 30 to 34 | -1.1 (-4.95, 2.91) |
| Marshall Islands | 35 to 39 | -0.22 (-5.38, 5.22) |
| Mauritania | 15 to 19 | -1.06 (-1.79, -0.32) |
| Mauritania | 20 to 24 | -1.07 (-1.64, -0.5) |
| Mauritania | 25 to 29 | -1.06 (-1.57, -0.54) |
| Mauritania | 30 to 34 | -1.01 (-1.51, -0.5) |
| Mauritania | 35 to 39 | -0.94 (-1.56, -0.32) |
| Mauritius | 15 to 19 | -0.32 (-1.43, 0.79) |
| Mauritius | 20 to 24 | -0.46 (-1.32, 0.41) |
| Mauritius | 25 to 29 | -0.61 (-1.38, 0.18) |
| Mauritius | 30 to 34 | -0.75 (-1.52, 0.02) |
| Mauritius | 35 to 39 | -0.89 (-1.78, 0.01) |
| Mexico | 15 to 19 | -0.46 (-0.56, -0.37) |
| Mexico | 20 to 24 | -0.45 (-0.52, -0.38) |
| Mexico | 25 to 29 | -0.4 (-0.46, -0.33) |
| Mexico | 30 to 34 | -0.35 (-0.42, -0.29) |
| Mexico | 35 to 39 | -0.33 (-0.42, -0.24) |
| Micronesia (Federated States of) | 15 to 19 | -1 (-4.83, 2.98) |
| Micronesia (Federated States of) | 20 to 24 | -1.31 (-4.43, 1.92) |
| Micronesia (Federated States of) | 25 to 29 | -1.09 (-4.01, 1.92) |
| Micronesia (Federated States of) | 30 to 34 | -0.61 (-3.54, 2.4) |
| Micronesia (Federated States of) | 35 to 39 | -0.35 (-3.94, 3.38) |
| Monaco | 15 to 19 | -0.93 (-8.6, 7.39) |
| Monaco | 20 to 24 | -0.46 (-6.45, 5.93) |
| Monaco | 25 to 29 | -0.24 (-5.8, 5.64) |
| Monaco | 30 to 34 | 0.46 (-5.58, 6.89) |
| Monaco | 35 to 39 | 0.97 (-6.81, 9.4) |
| Mongolia | 15 to 19 | -0.65 (-1.39, 0.1) |
| Mongolia | 20 to 24 | -0.68 (-1.22, -0.13) |
| Mongolia | 25 to 29 | -0.7 (-1.17, -0.23) |
| Mongolia | 30 to 34 | -0.71 (-1.18, -0.23) |
| Mongolia | 35 to 39 | -0.7 (-1.32, -0.07) |
| Montenegro | 15 to 19 | -0.14 (-2.41, 2.17) |
| Montenegro | 20 to 24 | -0.17 (-1.9, 1.58) |
| Montenegro | 25 to 29 | -0.17 (-1.67, 1.37) |
| Montenegro | 30 to 34 | -0.12 (-1.54, 1.32) |
| Montenegro | 35 to 39 | -0.19 (-1.82, 1.48) |
| Morocco | 15 to 19 | -0.51 (-0.69, -0.33) |
| Morocco | 20 to 24 | -0.56 (-0.69, -0.42) |
| Morocco | 25 to 29 | -0.62 (-0.75, -0.49) |
| Morocco | 30 to 34 | -0.67 (-0.81, -0.53) |
| Morocco | 35 to 39 | -0.72 (-0.91, -0.54) |
| Mozambique | 15 to 19 | -0.86 (-1.13, -0.58) |
| Mozambique | 20 to 24 | -0.87 (-1.09, -0.65) |
| Mozambique | 25 to 29 | -0.87 (-1.07, -0.67) |
| Mozambique | 30 to 34 | -0.82 (-1.02, -0.63) |
| Mozambique | 35 to 39 | -0.74 (-0.97, -0.5) |
| Myanmar | 15 to 19 | -0.9 (-1.04, -0.77) |
| Myanmar | 20 to 24 | -1.09 (-1.19, -0.98) |
| Myanmar | 25 to 29 | -1.2 (-1.29, -1.1) |
| Myanmar | 30 to 34 | -1.24 (-1.34, -1.14) |
| Myanmar | 35 to 39 | -1.23 (-1.35, -1.11) |
| Namibia | 15 to 19 | -0.87 (-1.75, 0.02) |
| Namibia | 20 to 24 | -0.86 (-1.52, -0.19) |
| Namibia | 25 to 29 | -0.83 (-1.4, -0.25) |
| Namibia | 30 to 34 | -0.78 (-1.32, -0.24) |
| Namibia | 35 to 39 | -0.74 (-1.39, -0.09) |
| Nauru | 15 to 19 | -0.13 (-8.45, 8.96) |
| Nauru | 20 to 24 | -0.43 (-8.71, 8.6) |
| Nauru | 25 to 29 | -0.55 (-8.92, 8.59) |
| Nauru | 30 to 34 | -0.56 (-9.31, 9.03) |
| Nauru | 35 to 39 | -0.47 (-10.87, 11.14) |
| Nepal | 15 to 19 | -0.67 (-0.9, -0.45) |
| Nepal | 20 to 24 | -0.61 (-0.79, -0.43) |
| Nepal | 25 to 29 | -0.56 (-0.71, -0.4) |
| Nepal | 30 to 34 | -0.46 (-0.6, -0.31) |
| Nepal | 35 to 39 | -0.25 (-0.41, -0.09) |
| Netherlands | 15 to 19 | -0.15 (-0.52, 0.22) |
| Netherlands | 20 to 24 | -0.17 (-0.44, 0.1) |
| Netherlands | 25 to 29 | -0.19 (-0.43, 0.05) |
| Netherlands | 30 to 34 | -0.22 (-0.48, 0.03) |
| Netherlands | 35 to 39 | -0.25 (-0.57, 0.08) |
| New Zealand | 15 to 19 | -0.19 (-0.81, 0.45) |
| New Zealand | 20 to 24 | -0.22 (-0.7, 0.27) |
| New Zealand | 25 to 29 | -0.25 (-0.7, 0.22) |
| New Zealand | 30 to 34 | -0.29 (-0.78, 0.2) |
| New Zealand | 35 to 39 | -0.33 (-0.96, 0.3) |
| Nicaragua | 15 to 19 | -0.42 (-0.81, -0.03) |
| Nicaragua | 20 to 24 | -0.43 (-0.73, -0.13) |
| Nicaragua | 25 to 29 | -0.46 (-0.74, -0.17) |
| Nicaragua | 30 to 34 | -0.5 (-0.8, -0.19) |
| Nicaragua | 35 to 39 | -0.56 (-0.96, -0.15) |
| Niger | 15 to 19 | -0.34 (-0.66, -0.02) |
| Niger | 20 to 24 | -0.31 (-0.57, -0.05) |
| Niger | 25 to 29 | -0.28 (-0.52, -0.05) |
| Niger | 30 to 34 | -0.22 (-0.44, 0) |
| Niger | 35 to 39 | -0.07 (-0.34, 0.21) |
| Nigeria | 15 to 19 | -0.34 (-0.42, -0.26) |
| Nigeria | 20 to 24 | -0.27 (-0.34, -0.21) |
| Nigeria | 25 to 29 | -0.17 (-0.23, -0.11) |
| Nigeria | 30 to 34 | -0.1 (-0.16, -0.03) |
| Nigeria | 35 to 39 | -0.03 (-0.12, 0.05) |
| Niue | 15 to 19 | -0.18 (-31.7, 45.89) |
| Niue | 20 to 24 | -0.28 (-25.68, 33.8) |
| Niue | 25 to 29 | -0.07 (-22.67, 29.14) |
| Niue | 30 to 34 | -0.7 (-23.2, 28.38) |
| Niue | 35 to 39 | -0.76 (-25.85, 32.81) |
| North Macedonia | 15 to 19 | -0.19 (-1.44, 1.07) |
| North Macedonia | 20 to 24 | -0.27 (-1.18, 0.65) |
| North Macedonia | 25 to 29 | -0.28 (-1.06, 0.51) |
| North Macedonia | 30 to 34 | -0.31 (-1.06, 0.44) |
| North Macedonia | 35 to 39 | -0.33 (-1.2, 0.55) |
| Northern Mariana Islands | 15 to 19 | -0.7 (-8.25, 7.47) |
| Northern Mariana Islands | 20 to 24 | 0.08 (-5.62, 6.12) |
| Northern Mariana Islands | 25 to 29 | 0.38 (-4.6, 5.61) |
| Northern Mariana Islands | 30 to 34 | -0.09 (-4.84, 4.9) |
| Northern Mariana Islands | 35 to 39 | -0.51 (-6.01, 5.32) |
| Norway | 15 to 19 | -0.07 (-0.65, 0.51) |
| Norway | 20 to 24 | -0.06 (-0.49, 0.37) |
| Norway | 25 to 29 | -0.07 (-0.46, 0.31) |
| Norway | 30 to 34 | -0.09 (-0.51, 0.32) |
| Norway | 35 to 39 | -0.11 (-0.63, 0.42) |
| Oman | 15 to 19 | -0.66 (-1.1, -0.22) |
| Oman | 20 to 24 | -0.66 (-0.98, -0.33) |
| Oman | 25 to 29 | -0.6 (-0.89, -0.32) |
| Oman | 30 to 34 | -0.5 (-0.81, -0.19) |
| Oman | 35 to 39 | -0.31 (-0.74, 0.12) |
| Pakistan | 15 to 19 | -0.47 (-0.55, -0.4) |
| Pakistan | 20 to 24 | -0.46 (-0.52, -0.4) |
| Pakistan | 25 to 29 | -0.43 (-0.49, -0.38) |
| Pakistan | 30 to 34 | -0.41 (-0.47, -0.35) |
| Pakistan | 35 to 39 | -0.37 (-0.44, -0.3) |
| Palau | 15 to 19 | -3.47 (-14.18, 8.57) |
| Palau | 20 to 24 | -2.25 (-10.13, 6.33) |
| Palau | 25 to 29 | -0.35 (-6.85, 6.6) |
| Palau | 30 to 34 | 0.6 (-5.55, 7.15) |
| Palau | 35 to 39 | -0.03 (-7.84, 8.44) |
| Palestine | 15 to 19 | -0.53 (-1, -0.05) |
| Palestine | 20 to 24 | -0.59 (-0.97, -0.2) |
| Palestine | 25 to 29 | -0.64 (-1.03, -0.26) |
| Palestine | 30 to 34 | -0.71 (-1.17, -0.25) |
| Palestine | 35 to 39 | -0.81 (-1.46, -0.15) |
| Panama | 15 to 19 | -0.3 (-0.83, 0.25) |
| Panama | 20 to 24 | -0.32 (-0.73, 0.09) |
| Panama | 25 to 29 | -0.33 (-0.7, 0.04) |
| Panama | 30 to 34 | -0.35 (-0.73, 0.03) |
| Panama | 35 to 39 | -0.39 (-0.87, 0.1) |
| Papua New Guinea | 15 to 19 | -0.34 (-0.76, 0.08) |
| Papua New Guinea | 20 to 24 | -0.36 (-0.7, -0.02) |
| Papua New Guinea | 25 to 29 | -0.36 (-0.68, -0.04) |
| Papua New Guinea | 30 to 34 | -0.34 (-0.67, -0.01) |
| Papua New Guinea | 35 to 39 | -0.3 (-0.73, 0.13) |
| Paraguay | 15 to 19 | -0.27 (-0.6, 0.06) |
| Paraguay | 20 to 24 | -0.29 (-0.55, -0.03) |
| Paraguay | 25 to 29 | -0.31 (-0.55, -0.06) |
| Paraguay | 30 to 34 | -0.33 (-0.6, -0.07) |
| Paraguay | 35 to 39 | -0.38 (-0.73, -0.02) |
| Peru | 15 to 19 | -0.45 (-0.61, -0.29) |
| Peru | 20 to 24 | -0.48 (-0.6, -0.35) |
| Peru | 25 to 29 | -0.53 (-0.64, -0.41) |
| Peru | 30 to 34 | -0.6 (-0.72, -0.48) |
| Peru | 35 to 39 | -0.66 (-0.81, -0.5) |
| Philippines | 15 to 19 | -0.31 (-0.42, -0.19) |
| Philippines | 20 to 24 | -0.42 (-0.51, -0.33) |
| Philippines | 25 to 29 | -0.49 (-0.58, -0.4) |
| Philippines | 30 to 34 | -0.52 (-0.61, -0.42) |
| Philippines | 35 to 39 | -0.51 (-0.63, -0.4) |
| Poland | 15 to 19 | -0.08 (-0.4, 0.23) |
| Poland | 20 to 24 | -0.09 (-0.31, 0.14) |
| Poland | 25 to 29 | -0.08 (-0.27, 0.12) |
| Poland | 30 to 34 | -0.07 (-0.25, 0.11) |
| Poland | 35 to 39 | -0.08 (-0.28, 0.13) |
| Portugal | 15 to 19 | -0.25 (-0.66, 0.17) |
| Portugal | 20 to 24 | -0.27 (-0.58, 0.04) |
| Portugal | 25 to 29 | -0.31 (-0.59, -0.03) |
| Portugal | 30 to 34 | -0.35 (-0.63, -0.06) |
| Portugal | 35 to 39 | -0.38 (-0.74, -0.02) |
| Puerto Rico | 15 to 19 | -0.22 (-0.91, 0.48) |
| Puerto Rico | 20 to 24 | -0.26 (-0.77, 0.25) |
| Puerto Rico | 25 to 29 | -0.31 (-0.77, 0.15) |
| Puerto Rico | 30 to 34 | -0.38 (-0.84, 0.08) |
| Puerto Rico | 35 to 39 | -0.45 (-1, 0.11) |
| Qatar | 15 to 19 | -0.81 (-1.87, 0.26) |
| Qatar | 20 to 24 | -0.83 (-1.61, -0.04) |
| Qatar | 25 to 29 | -0.85 (-1.5, -0.2) |
| Qatar | 30 to 34 | -0.9 (-1.51, -0.29) |
| Qatar | 35 to 39 | -0.96 (-1.78, -0.13) |
| Republic of Korea | 15 to 19 | -0.35 (-0.53, -0.16) |
| Republic of Korea | 20 to 24 | -0.38 (-0.51, -0.25) |
| Republic of Korea | 25 to 29 | -0.44 (-0.56, -0.32) |
| Republic of Korea | 30 to 34 | -0.53 (-0.66, -0.41) |
| Republic of Korea | 35 to 39 | -0.65 (-0.81, -0.49) |
| Republic of Moldova | 15 to 19 | -0.22 (-0.94, 0.49) |
| Republic of Moldova | 20 to 24 | -0.28 (-0.8, 0.24) |
| Republic of Moldova | 25 to 29 | -0.32 (-0.74, 0.11) |
| Republic of Moldova | 30 to 34 | -0.34 (-0.73, 0.05) |
| Republic of Moldova | 35 to 39 | -0.36 (-0.82, 0.09) |
| Romania | 15 to 19 | -0.16 (-0.59, 0.27) |
| Romania | 20 to 24 | -0.18 (-0.5, 0.13) |
| Romania | 25 to 29 | -0.2 (-0.47, 0.07) |
| Romania | 30 to 34 | -0.23 (-0.48, 0.02) |
| Romania | 35 to 39 | -0.26 (-0.53, 0.02) |
| Russian Federation | 15 to 19 | -0.2 (-0.33, -0.07) |
| Russian Federation | 20 to 24 | -0.24 (-0.34, -0.15) |
| Russian Federation | 25 to 29 | -0.26 (-0.34, -0.19) |
| Russian Federation | 30 to 34 | -0.25 (-0.32, -0.18) |
| Russian Federation | 35 to 39 | -0.19 (-0.27, -0.11) |
| Rwanda | 15 to 19 | -0.96 (-1.48, -0.44) |
| Rwanda | 20 to 24 | -1.06 (-1.47, -0.65) |
| Rwanda | 25 to 29 | -1.09 (-1.45, -0.73) |
| Rwanda | 30 to 34 | -1.05 (-1.39, -0.71) |
| Rwanda | 35 to 39 | -0.99 (-1.4, -0.58) |
| Saint Kitts and Nevis | 15 to 19 | -0.24 (-5.7, 5.54) |
| Saint Kitts and Nevis | 20 to 24 | -0.4 (-4.59, 3.98) |
| Saint Kitts and Nevis | 25 to 29 | -0.45 (-4.07, 3.31) |
| Saint Kitts and Nevis | 30 to 34 | -0.53 (-4.07, 3.15) |
| Saint Kitts and Nevis | 35 to 39 | -0.57 (-4.98, 4.04) |
| Saint Lucia | 15 to 19 | -0.71 (-3.71, 2.39) |
| Saint Lucia | 20 to 24 | -0.44 (-2.62, 1.8) |
| Saint Lucia | 25 to 29 | -0.44 (-2.42, 1.57) |
| Saint Lucia | 30 to 34 | -0.58 (-2.62, 1.5) |
| Saint Lucia | 35 to 39 | -0.53 (-3.16, 2.17) |
| Saint Vincent and the Grenadines | 15 to 19 | -0.35 (-3.98, 3.41) |
| Saint Vincent and the Grenadines | 20 to 24 | -0.08 (-2.72, 2.62) |
| Saint Vincent and the Grenadines | 25 to 29 | -0.31 (-2.7, 2.13) |
| Saint Vincent and the Grenadines | 30 to 34 | -0.39 (-2.83, 2.11) |
| Saint Vincent and the Grenadines | 35 to 39 | -0.65 (-3.65, 2.45) |
| Samoa | 15 to 19 | -0.41 (-3.46, 2.74) |
| Samoa | 20 to 24 | -0.36 (-2.81, 2.15) |
| Samoa | 25 to 29 | -0.52 (-2.82, 1.84) |
| Samoa | 30 to 34 | -0.41 (-2.77, 2) |
| Samoa | 35 to 39 | -0.23 (-3.15, 2.77) |
| San Marino | 15 to 19 | -0.14 (-7.89, 8.25) |
| San Marino | 20 to 24 | -0.24 (-6.27, 6.17) |
| San Marino | 25 to 29 | 0.06 (-5.61, 6.06) |
| San Marino | 30 to 34 | -0.14 (-6.19, 6.3) |
| San Marino | 35 to 39 | -0.62 (-8.27, 7.68) |
| Sao Tome and Principe | 15 to 19 | -0.97 (-4.18, 2.35) |
| Sao Tome and Principe | 20 to 24 | -0.84 (-3.31, 1.69) |
| Sao Tome and Principe | 25 to 29 | -0.98 (-3.18, 1.28) |
| Sao Tome and Principe | 30 to 34 | -1.01 (-3.21, 1.23) |
| Sao Tome and Principe | 35 to 39 | -1.13 (-3.89, 1.7) |
| Saudi Arabia | 15 to 19 | -1.65 (-1.79, -1.51) |
| Saudi Arabia | 20 to 24 | -1.68 (-1.79, -1.58) |
| Saudi Arabia | 25 to 29 | -1.69 (-1.79, -1.59) |
| Saudi Arabia | 30 to 34 | -1.7 (-1.8, -1.59) |
| Saudi Arabia | 35 to 39 | -1.71 (-1.85, -1.56) |
| Senegal | 15 to 19 | -1.2 (-1.56, -0.84) |
| Senegal | 20 to 24 | -1.15 (-1.43, -0.86) |
| Senegal | 25 to 29 | -1.07 (-1.32, -0.81) |
| Senegal | 30 to 34 | -0.97 (-1.22, -0.72) |
| Senegal | 35 to 39 | -0.86 (-1.18, -0.54) |
| Serbia | 15 to 19 | -0.1 (-0.73, 0.53) |
| Serbia | 20 to 24 | -0.15 (-0.61, 0.32) |
| Serbia | 25 to 29 | -0.18 (-0.58, 0.23) |
| Serbia | 30 to 34 | -0.21 (-0.58, 0.17) |
| Serbia | 35 to 39 | -0.23 (-0.66, 0.19) |
| Seychelles | 15 to 19 | -0.09 (-4.04, 4.01) |
| Seychelles | 20 to 24 | -0.66 (-3.8, 2.57) |
| Seychelles | 25 to 29 | -0.66 (-3.39, 2.15) |
| Seychelles | 30 to 34 | -0.57 (-3.33, 2.27) |
| Seychelles | 35 to 39 | -0.76 (-4.14, 2.74) |
| Sierra Leone | 15 to 19 | -0.39 (-0.92, 0.13) |
| Sierra Leone | 20 to 24 | -0.1 (-0.52, 0.33) |
| Sierra Leone | 25 to 29 | 0.34 (-0.06, 0.73) |
| Sierra Leone | 30 to 34 | 0.75 (0.34, 1.16) |
| Sierra Leone | 35 to 39 | 1 (0.47, 1.53) |
| Singapore | 15 to 19 | -0.23 (-0.82, 0.35) |
| Singapore | 20 to 24 | -0.24 (-0.64, 0.16) |
| Singapore | 25 to 29 | -0.26 (-0.61, 0.09) |
| Singapore | 30 to 34 | -0.27 (-0.63, 0.1) |
| Singapore | 35 to 39 | -0.27 (-0.75, 0.22) |
| Slovakia | 15 to 19 | -0.13 (-0.97, 0.71) |
| Slovakia | 20 to 24 | -0.14 (-0.75, 0.47) |
| Slovakia | 25 to 29 | -0.16 (-0.67, 0.35) |
| Slovakia | 30 to 34 | -0.18 (-0.65, 0.3) |
| Slovakia | 35 to 39 | -0.21 (-0.74, 0.33) |
| Slovenia | 15 to 19 | -0.16 (-1.63, 1.33) |
| Slovenia | 20 to 24 | -0.15 (-1.22, 0.94) |
| Slovenia | 25 to 29 | -0.15 (-1.04, 0.74) |
| Slovenia | 30 to 34 | -0.19 (-1, 0.63) |
| Slovenia | 35 to 39 | -0.22 (-1.13, 0.7) |
| Solomon Islands | 15 to 19 | -0.39 (-2.15, 1.4) |
| Solomon Islands | 20 to 24 | -0.45 (-1.87, 0.98) |
| Solomon Islands | 25 to 29 | -0.51 (-1.85, 0.85) |
| Solomon Islands | 30 to 34 | -0.57 (-1.96, 0.84) |
| Solomon Islands | 35 to 39 | -0.5 (-2.28, 1.32) |
| Somalia | 15 to 19 | -0.48 (-0.84, -0.11) |
| Somalia | 20 to 24 | -0.43 (-0.73, -0.12) |
| Somalia | 25 to 29 | -0.35 (-0.64, -0.06) |
| Somalia | 30 to 34 | -0.25 (-0.53, 0.03) |
| Somalia | 35 to 39 | -0.17 (-0.49, 0.15) |
| South Africa | 15 to 19 | -0.71 (-0.9, -0.51) |
| South Africa | 20 to 24 | -0.75 (-0.89, -0.61) |
| South Africa | 25 to 29 | -0.75 (-0.86, -0.64) |
| South Africa | 30 to 34 | -0.67 (-0.77, -0.57) |
| South Africa | 35 to 39 | -0.47 (-0.59, -0.35) |
| South Sudan | 15 to 19 | -0.42 (-0.78, -0.05) |
| South Sudan | 20 to 24 | -0.51 (-0.82, -0.2) |
| South Sudan | 25 to 29 | -0.51 (-0.81, -0.21) |
| South Sudan | 30 to 34 | -0.4 (-0.71, -0.08) |
| South Sudan | 35 to 39 | -0.18 (-0.58, 0.23) |
| Spain | 15 to 19 | -0.03 (-0.19, 0.12) |
| Spain | 20 to 24 | -0.03 (-0.14, 0.08) |
| Spain | 25 to 29 | -0.04 (-0.14, 0.06) |
| Spain | 30 to 34 | -0.06 (-0.16, 0.04) |
| Spain | 35 to 39 | -0.09 (-0.21, 0.04) |
| Sri Lanka | 15 to 19 | -0.36 (-0.62, -0.11) |
| Sri Lanka | 20 to 24 | -0.5 (-0.71, -0.3) |
| Sri Lanka | 25 to 29 | -0.62 (-0.81, -0.43) |
| Sri Lanka | 30 to 34 | -0.69 (-0.88, -0.5) |
| Sri Lanka | 35 to 39 | -0.7 (-0.93, -0.47) |
| Sudan | 15 to 19 | -0.88 (-1.03, -0.73) |
| Sudan | 20 to 24 | -0.83 (-0.96, -0.71) |
| Sudan | 25 to 29 | -0.8 (-0.92, -0.68) |
| Sudan | 30 to 34 | -0.8 (-0.94, -0.66) |
| Sudan | 35 to 39 | -0.79 (-0.98, -0.6) |
| Suriname | 15 to 19 | -0.21 (-1.76, 1.36) |
| Suriname | 20 to 24 | -0.28 (-1.45, 0.9) |
| Suriname | 25 to 29 | -0.35 (-1.39, 0.69) |
| Suriname | 30 to 34 | -0.43 (-1.48, 0.63) |
| Suriname | 35 to 39 | -0.47 (-1.84, 0.93) |
| Sweden | 15 to 19 | 0.01 (-0.54, 0.57) |
| Sweden | 20 to 24 | -0.02 (-0.43, 0.38) |
| Sweden | 25 to 29 | -0.05 (-0.4, 0.3) |
| Sweden | 30 to 34 | -0.09 (-0.46, 0.28) |
| Sweden | 35 to 39 | -0.11 (-0.58, 0.36) |
| Switzerland | 15 to 19 | -0.11 (-0.63, 0.42) |
| Switzerland | 20 to 24 | -0.1 (-0.47, 0.28) |
| Switzerland | 25 to 29 | -0.11 (-0.44, 0.21) |
| Switzerland | 30 to 34 | -0.13 (-0.46, 0.2) |
| Switzerland | 35 to 39 | -0.13 (-0.56, 0.3) |
| Syrian Arab Republic | 15 to 19 | -0.55 (-0.76, -0.34) |
| Syrian Arab Republic | 20 to 24 | -0.56 (-0.74, -0.38) |
| Syrian Arab Republic | 25 to 29 | -0.59 (-0.77, -0.41) |
| Syrian Arab Republic | 30 to 34 | -0.65 (-0.84, -0.46) |
| Syrian Arab Republic | 35 to 39 | -0.7 (-0.96, -0.44) |
| Taiwan (Province of China) | 15 to 19 | -0.3 (-0.8, 0.19) |
| Taiwan (Province of China) | 20 to 24 | -0.31 (-0.68, 0.07) |
| Taiwan (Province of China) | 25 to 29 | -0.31 (-0.64, 0.02) |
| Taiwan (Province of China) | 30 to 34 | -0.32 (-0.62, -0.02) |
| Taiwan (Province of China) | 35 to 39 | -0.34 (-0.67, -0.01) |
| Tajikistan | 15 to 19 | -0.2 (-0.64, 0.25) |
| Tajikistan | 20 to 24 | -0.23 (-0.58, 0.12) |
| Tajikistan | 25 to 29 | -0.25 (-0.57, 0.07) |
| Tajikistan | 30 to 34 | -0.27 (-0.6, 0.06) |
| Tajikistan | 35 to 39 | -0.26 (-0.69, 0.18) |
| Thailand | 15 to 19 | -0.41 (-0.56, -0.26) |
| Thailand | 20 to 24 | -0.53 (-0.64, -0.41) |
| Thailand | 25 to 29 | -0.63 (-0.73, -0.53) |
| Thailand | 30 to 34 | -0.7 (-0.8, -0.6) |
| Thailand | 35 to 39 | -0.73 (-0.84, -0.61) |
| Timor-Leste | 15 to 19 | -0.94 (-1.81, -0.06) |
| Timor-Leste | 20 to 24 | -1.07 (-1.78, -0.34) |
| Timor-Leste | 25 to 29 | -1.18 (-1.86, -0.49) |
| Timor-Leste | 30 to 34 | -1.21 (-1.92, -0.5) |
| Timor-Leste | 35 to 39 | -1.17 (-2.04, -0.29) |
| Togo | 15 to 19 | -0.62 (-1.15, -0.09) |
| Togo | 20 to 24 | -0.53 (-0.94, -0.12) |
| Togo | 25 to 29 | -0.41 (-0.78, -0.04) |
| Togo | 30 to 34 | -0.25 (-0.61, 0.13) |
| Togo | 35 to 39 | -0.01 (-0.49, 0.47) |
| Tokelau | 15 to 19 | -0.59 (-33.22, 48) |
| Tokelau | 20 to 24 | -0.68 (-27.15, 35.41) |
| Tokelau | 25 to 29 | -0.77 (-25.35, 31.9) |
| Tokelau | 30 to 34 | -0.84 (-24.89, 30.92) |
| Tokelau | 35 to 39 | -0.9 (-29.24, 38.78) |
| Tonga | 15 to 19 | -0.88 (-5.99, 4.51) |
| Tonga | 20 to 24 | -0.56 (-4.41, 3.44) |
| Tonga | 25 to 29 | -0.44 (-4.21, 3.48) |
| Tonga | 30 to 34 | -0.58 (-4.53, 3.54) |
| Tonga | 35 to 39 | -0.69 (-5.3, 4.15) |
| Trinidad and Tobago | 15 to 19 | -0.23 (-1.36, 0.91) |
| Trinidad and Tobago | 20 to 24 | -0.24 (-1.06, 0.58) |
| Trinidad and Tobago | 25 to 29 | -0.31 (-0.99, 0.38) |
| Trinidad and Tobago | 30 to 34 | -0.34 (-0.99, 0.32) |
| Trinidad and Tobago | 35 to 39 | -0.29 (-1.11, 0.54) |
| Tunisia | 15 to 19 | -0.38 (-0.73, -0.04) |
| Tunisia | 20 to 24 | -0.43 (-0.68, -0.17) |
| Tunisia | 25 to 29 | -0.45 (-0.68, -0.22) |
| Tunisia | 30 to 34 | -0.45 (-0.7, -0.2) |
| Tunisia | 35 to 39 | -0.46 (-0.79, -0.13) |
| Turkey | 15 to 19 | -0.36 (-0.49, -0.22) |
| Turkey | 20 to 24 | -0.42 (-0.52, -0.32) |
| Turkey | 25 to 29 | -0.52 (-0.61, -0.42) |
| Turkey | 30 to 34 | -0.65 (-0.75, -0.54) |
| Turkey | 35 to 39 | -0.79 (-0.93, -0.66) |
| Turkmenistan | 15 to 19 | -0.34 (-0.89, 0.22) |
| Turkmenistan | 20 to 24 | -0.39 (-0.81, 0.03) |
| Turkmenistan | 25 to 29 | -0.42 (-0.8, -0.04) |
| Turkmenistan | 30 to 34 | -0.45 (-0.83, -0.07) |
| Turkmenistan | 35 to 39 | -0.47 (-0.95, 0.02) |
| Tuvalu | 15 to 19 | -0.69 (-13.32, 13.78) |
| Tuvalu | 20 to 24 | -0.74 (-10.83, 10.49) |
| Tuvalu | 25 to 29 | -0.73 (-9.67, 9.09) |
| Tuvalu | 30 to 34 | -0.64 (-8.85, 8.3) |
| Tuvalu | 35 to 39 | -0.52 (-8.83, 8.56) |
| Uganda | 15 to 19 | -0.73 (-1.01, -0.45) |
| Uganda | 20 to 24 | -0.71 (-0.94, -0.48) |
| Uganda | 25 to 29 | -0.59 (-0.8, -0.38) |
| Uganda | 30 to 34 | -0.36 (-0.57, -0.15) |
| Uganda | 35 to 39 | -0.09 (-0.37, 0.18) |
| Ukraine | 15 to 19 | -0.13 (-0.38, 0.12) |
| Ukraine | 20 to 24 | -0.16 (-0.33, 0.02) |
| Ukraine | 25 to 29 | -0.17 (-0.31, -0.02) |
| Ukraine | 30 to 34 | -0.17 (-0.3, -0.04) |
| Ukraine | 35 to 39 | -0.16 (-0.32, -0.01) |
| United Arab Emirates | 15 to 19 | -0.52 (-1.05, 0.01) |
| United Arab Emirates | 20 to 24 | -0.55 (-0.95, -0.14) |
| United Arab Emirates | 25 to 29 | -0.6 (-0.91, -0.29) |
| United Arab Emirates | 30 to 34 | -0.65 (-0.95, -0.35) |
| United Arab Emirates | 35 to 39 | -0.69 (-1.08, -0.29) |
| United Kingdom | 15 to 19 | -0.06 (-0.23, 0.1) |
| United Kingdom | 20 to 24 | -0.06 (-0.18, 0.06) |
| United Kingdom | 25 to 29 | -0.07 (-0.17, 0.04) |
| United Kingdom | 30 to 34 | -0.08 (-0.19, 0.03) |
| United Kingdom | 35 to 39 | -0.09 (-0.23, 0.06) |
| United Republic of Tanzania | 15 to 19 | -1.02 (-1.21, -0.83) |
| United Republic of Tanzania | 20 to 24 | -0.91 (-1.06, -0.76) |
| United Republic of Tanzania | 25 to 29 | -0.8 (-0.93, -0.67) |
| United Republic of Tanzania | 30 to 34 | -0.68 (-0.81, -0.56) |
| United Republic of Tanzania | 35 to 39 | -0.62 (-0.77, -0.47) |
| United States of America | 15 to 19 | 0.01 (-0.14, 0.16) |
| United States of America | 20 to 24 | 0.08 (-0.03, 0.19) |
| United States of America | 25 to 29 | 0.07 (-0.03, 0.17) |
| United States of America | 30 to 34 | -0.01 (-0.12, 0.09) |
| United States of America | 35 to 39 | -0.15 (-0.29, -0.01) |
| United States Virgin Islands | 15 to 19 | -0.56 (-4.75, 3.83) |
| United States Virgin Islands | 20 to 24 | -0.52 (-3.75, 2.82) |
| United States Virgin Islands | 25 to 29 | -0.55 (-3.35, 2.32) |
| United States Virgin Islands | 30 to 34 | -0.48 (-3.14, 2.24) |
| United States Virgin Islands | 35 to 39 | -0.54 (-3.61, 2.63) |
| Uruguay | 15 to 19 | -0.09 (-0.73, 0.55) |
| Uruguay | 20 to 24 | -0.11 (-0.6, 0.39) |
| Uruguay | 25 to 29 | -0.13 (-0.61, 0.35) |
| Uruguay | 30 to 34 | -0.16 (-0.68, 0.36) |
| Uruguay | 35 to 39 | -0.19 (-0.86, 0.48) |
| Uzbekistan | 15 to 19 | -0.33 (-0.57, -0.1) |
| Uzbekistan | 20 to 24 | -0.38 (-0.56, -0.19) |
| Uzbekistan | 25 to 29 | -0.41 (-0.57, -0.24) |
| Uzbekistan | 30 to 34 | -0.42 (-0.59, -0.26) |
| Uzbekistan | 35 to 39 | -0.43 (-0.64, -0.22) |
| Vanuatu | 15 to 19 | -0.57 (-3.75, 2.7) |
| Vanuatu | 20 to 24 | -0.46 (-3.02, 2.17) |
| Vanuatu | 25 to 29 | -0.35 (-2.73, 2.07) |
| Vanuatu | 30 to 34 | -0.41 (-2.81, 2.05) |
| Vanuatu | 35 to 39 | -0.73 (-3.6, 2.21) |
| Venezuela (Bolivarian Republic of) | 15 to 19 | -0.31 (-0.53, -0.1) |
| Venezuela (Bolivarian Republic of) | 20 to 24 | -0.31 (-0.47, -0.15) |
| Venezuela (Bolivarian Republic of) | 25 to 29 | -0.3 (-0.44, -0.16) |
| Venezuela (Bolivarian Republic of) | 30 to 34 | -0.27 (-0.42, -0.13) |
| Venezuela (Bolivarian Republic of) | 35 to 39 | -0.25 (-0.44, -0.07) |
| Viet Nam | 15 to 19 | -0.18 (-0.4, 0.03) |
| Viet Nam | 20 to 24 | -0.41 (-0.57, -0.25) |
| Viet Nam | 25 to 29 | -0.65 (-0.79, -0.5) |
| Viet Nam | 30 to 34 | -0.91 (-1.06, -0.76) |
| Viet Nam | 35 to 39 | -1.13 (-1.32, -0.95) |
| Yemen | 15 to 19 | -0.56 (-0.77, -0.36) |
| Yemen | 20 to 24 | -0.58 (-0.75, -0.4) |
| Yemen | 25 to 29 | -0.55 (-0.72, -0.38) |
| Yemen | 30 to 34 | -0.46 (-0.65, -0.27) |
| Yemen | 35 to 39 | -0.29 (-0.55, -0.03) |
| Zambia | 15 to 19 | -0.98 (-1.4, -0.56) |
| Zambia | 20 to 24 | -1.04 (-1.37, -0.71) |
| Zambia | 25 to 29 | -1.05 (-1.34, -0.75) |
| Zambia | 30 to 34 | -0.99 (-1.27, -0.7) |
| Zambia | 35 to 39 | -0.93 (-1.29, -0.58) |
| Zimbabwe | 15 to 19 | -0.14 (-0.47, 0.18) |
| Zimbabwe | 20 to 24 | -0.15 (-0.4, 0.11) |
| Zimbabwe | 25 to 29 | -0.13 (-0.36, 0.09) |
| Zimbabwe | 30 to 34 | -0.12 (-0.33, 0.09) |
| Zimbabwe | 35 to 39 | -0.12 (-0.37, 0.14) |

**Abbreviations:** DALYs, Disability adjusted life years.

**Table S4 Age effects on blindness and vision loss DALYs in teenagers and young adults across SDI quintiles**

| **Location** | **Age** | **DALYs rate (per 100,000 population)** |
| --- | --- | --- |
| Global | 15 to 19 | 57.76 (57.50, 58.02) |
| Global | 20 to 24 | 61.25 (60.99, 61.50) |
| Global | 25 to 29 | 66.14 (65.87, 66.40) |
| Global | 30 to 34 | 81.50 (81.19, 81.82) |
| Global | 35 to 39 | 114.25 (113.82, 114.68) |
| High SDI | 15 to 19 | 51.19 (50.67, 51.71) |
| High SDI | 20 to 24 | 54.20 (53.70, 54.71) |
| High SDI | 25 to 29 | 54.40 (53.91, 54.90) |
| High SDI | 30 to 34 | 55.26 (54.76, 55.76) |
| High SDI | 35 to 39 | 60.41 (59.85, 60.97) |
| High-middle SDI | 15 to 19 | 52.11 (51.61, 52.61) |
| High-middle SDI | 20 to 24 | 52.63 (52.17, 53.10) |
| High-middle SDI | 25 to 29 | 55.01 (54.54, 55.48) |
| High-middle SDI | 30 to 34 | 64.98 (64.45, 65.51) |
| High-middle SDI | 35 to 39 | 87.70 (87.00, 88.41) |
| Middle SDI | 15 to 19 | 60.86 (60.59, 61.13) |
| Middle SDI | 20 to 24 | 64.15 (63.89, 64.41) |
| Middle SDI | 25 to 29 | 68.43 (68.16, 68.70) |
| Middle SDI | 30 to 34 | 83.39 (83.07, 83.71) |
| Middle SDI | 35 to 39 | 116.70 (116.27, 117.13) |
| Low-middle SDI | 15 to 19 | 63.77 (63.43, 64.12) |
| Low-middle SDI | 20 to 24 | 68.35 (68.00, 68.69) |
| Low-middle SDI | 25 to 29 | 76.28 (75.91, 76.65) |
| Low-middle SDI | 30 to 34 | 101.33 (100.86, 101.80) |
| Low-middle SDI | 35 to 39 | 153.72 (153.04, 154.40) |
| Low SDI | 15 to 19 | 52.48 (52.13, 52.83) |
| Low SDI | 20 to 24 | 60.36 (60.00, 60.72) |
| Low SDI | 25 to 29 | 71.91 (71.50, 72.32) |
| Low SDI | 30 to 34 | 99.74 (99.20, 100.28) |
| Low SDI | 35 to 39 | 152.73 (151.94, 153.52) |

**Abbreviations:** DALYs, Disability adjusted life years; SDI, sociodemographic index.

**Table S5 Period effects on blindness and vision loss DALYs in teenagers and young adults across SDI quintiles**

| **Location** | **Period** | **DALYs rate ratio** |
| --- | --- | --- |
| Global | 1990 to 1994 | 1.000 (1.000, 1.000) |
| Global | 1995 to 1999 | 0.996 (0.991, 1.000) |
| Global | 2000 to 2004 | 0.974 (0.970, 0.979) |
| Global | 2005 to 2009 | 0.948 (0.944, 0.952) |
| Global | 2010 to 2014 | 0.922 (0.918, 0.926) |
| Global | 2015 to 2019 | 0.904 (0.900, 0.908) |
| High SDI | 1990 to 1994 | 1.000 (1.000, 1.000) |
| High SDI | 1995 to 1999 | 0.994 (0.984, 1.004) |
| High SDI | 2000 to 2004 | 0.986 (0.976, 0.997) |
| High SDI | 2005 to 2009 | 0.984 (0.974, 0.995) |
| High SDI | 2010 to 2014 | 0.989 (0.979, 0.999) |
| High SDI | 2015 to 2019 | 0.992 (0.982, 1.001) |
| High-middle SDI | 1990 to 1994 | 1.000 (1.000, 1.000) |
| High-middle SDI | 1995 to 1999 | 0.992 (0.982, 1.001) |
| High-middle SDI | 2000 to 2004 | 0.970 (0.9602, 0.9795) |
| High-middle SDI | 2005 to 2009 | 0.952 (0.943, 0.962) |
| High-middle SDI | 2010 to 2014 | 0.930 (0.921, 0.939) |
| High-middle SDI | 2015 to 2019 | 0.93071 (0.922, 0.939) |
| Middle SDI | 1990 to 1994 | 1.000 (1.000, 1.000) |
| Middle SDI | 1995 to 1999 | 0.994 (0.989, 0.998) |
| Middle SDI | 2000 to 2004 | 0.974 (0.969, 0.978) |
| Middle SDI | 2005 to 2009 | 0.953 (0.948, 0.957) |
| Middle SDI | 2010 to 2014 | 0.925 (0.921, 0.929) |
| Middle SDI | 2015 to 2019 | 0.906 (0.902, 0.910) |
| Low-middle SDI | 1990 to 1994 | 1.000 (1.000, 1.000) |
| Low-middle SDI | 1995 to 1999 | 0.996 (0.990, 1.001) |
| Low-middle SDI | 2000 to 2004 | 0.958 (0.952, 0.963) |
| Low-middle SDI | 2005 to 2009 | 0.899 (0.894, 0.904) |
| Low-middle SDI | 2010 to 2014 | 0.849 (0.844, 0.854) |
| Low-middle SDI | 2015 to 2019 | 0.806 (0.801, 0.810) |
| Low SDI | 1990 to 1994 | 1.000 (1.000, 1.000) |
| Low SDI | 1995 to 1999 | 1.007 (1.000, 1.014) |
| Low SDI | 2000 to 2004 | 0.983 (0.976, 0.990) |
| Low SDI | 2005 to 2009 | 0.946 (0.939, 0.953) |
| Low SDI | 2010 to 2014 | 0.909 (0.903, 0.915) |
| Low SDI | 2015 to 2019 | 0.864 (0.858, 0.869) |

**Abbreviations:** DALYs, Disability adjusted life years; SDI, sociodemographic index.

**Table S6 Cohort effects on blindness and vision loss DALYs in teenagers and young adults across SDI quintiles**

| **Location** | **Cohort** | **DALYs rate ratio** |
| --- | --- | --- |
| Global | 1950 to 1959 | 1.071 (1.063, 1.079) |
| Global | 1955 to 1964 | 1.069 (1.063, 1.075) |
| Global | 1960 to 1969 | 1.032 (1.027, 1.037) |
| Global | 1965 to 1974 | 1.008 (1.004, 1.013) |
| Global | 1970 to 1979 | 1.000 (1.000, 1.000) |
| Global | 1975 to 1984 | 0.989 (0.985, 0.993) |
| Global | 1980 to 1989 | 0.951 (0.947, 0.955) |
| Global | 1985 to 1994 | 0.919 (0.914, 0.924) |
| Global | 1990 to 1999 | 0.911 (0.904, 0.916) |
| Global | 1995 to 2004 | 0.890 (0.882, 0.898) |
| High SDI | 1950 to 1959 | 1.00 (0.986, 1.023) |
| High SDI | 1955 to 1964 | 1.000 (0.987, 1.013) |
| High SDI | 1960 to 1969 | 1.001 (0.990, 1.013) |
| High SDI | 1965 to 1974 | 1.000 (0.990, 1.011) |
| High SDI | 1970 to 1979 | 1.000 (1.000, 1.000) |
| High SDI | 1975 to 1984 | 1.003 (0.994, 1.013) |
| High SDI | 1980 to 1989 | 1.002 (0.991, 1.012) |
| High SDI | 1985 to 1994 | 0.996 (0.985, 1.008) |
| High SDI | 1990 to 1999 | 0.989 (0.975, 1.004) |
| High SDI | 1995 to 2004 | 0.972 (0.952, 0.992) |
| High-middle SDI | 1950 to 1959 | 1.071 (1.054, 1.087) |
| High-middle SDI | 1955 to 1964 | 1.069 (1.056, 1.082) |
| High-middle SDI | 1960 to 1969 | 1.034 (1.023, 1.045) |
| High-middle SDI | 1965 to 1974 | 1.005 (0.995, 1.014) |
| High-middle SDI | 1970 to 1979 | 1.000 (1.000, 1.000) |
| High-middle SDI | 1975 to 1984 | 0.991 (0.982, 1.000) |
| High-middle SDI | 1980 to 1989 | 0.943 (0.933, 0.952) |
| High-middle SDI | 1985 to 1994 | 0.921 (0.911, 0.931) |
| High-middle SDI | 1990 to 1999 | 0.962 (0.949, 0.976) |
| High-middle SDI | 1995 to 2004 | 0.981 (0.961, 1.002) |
| Middle SDI | 1950 to 1959 | 1.118 (1.110, 1.127) |
| Middle SDI | 1955 to 1964 | 1.118 (1.112, 1.125) |
| Middle SDI | 1960 to 1969 | 1.035 (1.030, 1.040) |
| Middle SDI | 1965 to 1974 | 0.998 (0.994, 1.003) |
| Middle SDI | 1970 to 1979 | 1.000 (1.000, 1.000) |
| Middle SDI | 1975 to 1984 | 1.008 (1.003, 1.012) |
| Middle SDI | 1980 to 1989 | 0.965 (0.961, 0.969) |
| Middle SDI | 1985 to 1994 | 0.928 (0.923, 0.933) |
| Middle SDI | 1990 to 1999 | 0.934 (0.928, 0.940) |
| Middle SDI | 1995 to 2004 | 0.932 (0.923, 0.940) |
| Low-middle SDI | 1950 to 1959 | 1.221 (1.210, 1.232) |
| Low-middle SDI | 1955 to 1964 | 1.169 (1.161, 1.177) |
| Low-middle SDI | 1960 to 1969 | 1.097 (1.091, 1.104) |
| Low-middle SDI | 1965 to 1974 | 1.044 (1.038, 1.049) |
| Low-middle SDI | 1970 to 1979 | 1.000 (1.000, 1.000) |
| Low-middle SDI | 1975 to 1984 | 0.962 (0.957, 0.967) |
| Low-middle SDI | 1980 to 1989 | 0.917 (0.912, 0.922) |
| Low-middle SDI | 1985 to 1994 | 0.872 (0.866, 0.878) |
| Low-middle SDI | 1990 to 1999 | 0.835 (0.829, 0.842) |
| Low-middle SDI | 1995 to 2004 | 0.804 (0.795, 0.813) |
| Low SDI | 1950 to 1959 | 1.117 (1.105, 1.130) |
| Low SDI | 1955 to 1964 | 1.091 (1.082, 1.100) |
| Low SDI | 1960 to 1969 | 1.066 (1.058, 1.074) |
| Low SDI | 1965 to 1974 | 1.038 (1.031, 1.045) |
| Low SDI | 1970 to 1979 | 1.000 (1.000, 1.000) |
| Low SDI | 1975 to 1984 | 0.964 (0.958, 0.969) |
| Low SDI | 1980 to 1989 | 0.934 (0.928, 0.940) |
| Low SDI | 1985 to 1994 | 0.908 (0.902, 0.915) |
| Low SDI | 1990 to 1999 | 0.886 (0.878, 0.894) |
| Low SDI | 1995 to 2004 | 0.863 (0.853, 0.874) |

**Abbreviations:** DALYs, Disability adjusted life years; SDI, sociodemographic index.

**Table S7 Age effects on blindness and vision loss DALYs in teenagers and young adults across countries**

| **Location** | **Age** | **DALYs rate** |
| --- | --- | --- |
| Afghanistan | 15 to 19 | 110.86 (107.81, 113.99) |
| Afghanistan | 20 to 24 | 118.64 (115.71, 121.65) |
| Afghanistan | 25 to 29 | 121.87 (118.83, 124.98) |
| Afghanistan | 30 to 34 | 127.87 (124.58, 131.24) |
| Afghanistan | 35 to 39 | 143.3 (139.46, 147.24) |
| Albania | 15 to 19 | 28.43 (24.87, 32.49) |
| Albania | 20 to 24 | 28.99 (25.45, 33.02) |
| Albania | 25 to 29 | 32.01 (28.04, 36.54) |
| Albania | 30 to 34 | 40.97 (36.01, 46.62) |
| Albania | 35 to 39 | 59.43 (52.29, 67.55) |
| Algeria | 15 to 19 | 88.18 (86.13, 90.29) |
| Algeria | 20 to 24 | 93.44 (91.4, 95.52) |
| Algeria | 25 to 29 | 94.22 (92.17, 96.32) |
| Algeria | 30 to 34 | 97.34 (95.18, 99.55) |
| Algeria | 35 to 39 | 108.85 (106.33, 111.42) |
| American Samoa | 15 to 19 | 44.01 (17.99, 107.66) |
| American Samoa | 20 to 24 | 43.65 (18.72, 101.76) |
| American Samoa | 25 to 29 | 43.53 (18.56, 102.06) |
| American Samoa | 30 to 34 | 52.2 (22.46, 121.32) |
| American Samoa | 35 to 39 | 78.44 (34.98, 175.89) |
| Andorra | 15 to 19 | 54.63 (24.25, 123.07) |
| Andorra | 20 to 24 | 56.68 (28.62, 112.26) |
| Andorra | 25 to 29 | 60.55 (32.13, 114.12) |
| Andorra | 30 to 34 | 59.65 (32.89, 108.21) |
| Andorra | 35 to 39 | 62.17 (33.55, 115.2) |
| Angola | 15 to 19 | 36.98 (35.05, 39.02) |
| Angola | 20 to 24 | 43.45 (41.44, 45.56) |
| Angola | 25 to 29 | 52.21 (49.96, 54.57) |
| Angola | 30 to 34 | 71.18 (68.28, 74.21) |
| Angola | 35 to 39 | 102.87 (98.84, 107.07) |
| Antigua and Barbuda | 15 to 19 | 45.56 (23, 90.25) |
| Antigua and Barbuda | 20 to 24 | 56.39 (30.45, 104.44) |
| Antigua and Barbuda | 25 to 29 | 59.07 (33.33, 104.71) |
| Antigua and Barbuda | 30 to 34 | 69.89 (40.39, 120.96) |
| Antigua and Barbuda | 35 to 39 | 90.04 (52.01, 155.88) |
| Argentina | 15 to 19 | 74.37 (72.47, 76.31) |
| Argentina | 20 to 24 | 75.8 (73.97, 77.68) |
| Argentina | 25 to 29 | 74.44 (72.63, 76.29) |
| Argentina | 30 to 34 | 74.29 (72.44, 76.18) |
| Argentina | 35 to 39 | 81.28 (79.2, 83.42) |
| Armenia | 15 to 19 | 45.98 (41.25, 51.24) |
| Armenia | 20 to 24 | 45.65 (41.12, 50.69) |
| Armenia | 25 to 29 | 49.12 (44.32, 54.44) |
| Armenia | 30 to 34 | 60.77 (54.97, 67.17) |
| Armenia | 35 to 39 | 86.8 (78.64, 95.82) |
| Australia | 15 to 19 | 54.13 (51.7, 56.68) |
| Australia | 20 to 24 | 54.02 (51.75, 56.39) |
| Australia | 25 to 29 | 52.78 (50.59, 55.06) |
| Australia | 30 to 34 | 52.49 (50.32, 54.76) |
| Australia | 35 to 39 | 56.53 (54.14, 59.03) |
| Austria | 15 to 19 | 54.75 (50.84, 58.97) |
| Austria | 20 to 24 | 59.63 (55.8, 63.72) |
| Austria | 25 to 29 | 60.26 (56.54, 64.23) |
| Austria | 30 to 34 | 60.46 (56.73, 64.44) |
| Austria | 35 to 39 | 65.09 (60.94, 69.53) |
| Azerbaijan | 15 to 19 | 48.83 (45.78, 52.09) |
| Azerbaijan | 20 to 24 | 49.11 (46.23, 52.17) |
| Azerbaijan | 25 to 29 | 53.21 (50.21, 56.38) |
| Azerbaijan | 30 to 34 | 65.77 (62.2, 69.54) |
| Azerbaijan | 35 to 39 | 93.31 (88.35, 98.54) |
| Bahamas | 15 to 19 | 45.38 (32.37, 63.61) |
| Bahamas | 20 to 24 | 54.04 (40.11, 72.8) |
| Bahamas | 25 to 29 | 59.24 (44.64, 78.61) |
| Bahamas | 30 to 34 | 70.54 (53.76, 92.56) |
| Bahamas | 35 to 39 | 90.36 (69.07, 118.22) |
| Bahrain | 15 to 19 | 87.04 (73.14, 103.6) |
| Bahrain | 20 to 24 | 89.95 (77.57, 104.29) |
| Bahrain | 25 to 29 | 88.86 (77.88, 101.39) |
| Bahrain | 30 to 34 | 89.04 (78.85, 100.55) |
| Bahrain | 35 to 39 | 96.29 (85.66, 108.23) |
| Bangladesh | 15 to 19 | 49.97 (49.22, 50.74) |
| Bangladesh | 20 to 24 | 53.29 (52.55, 54.04) |
| Bangladesh | 25 to 29 | 61.5 (60.69, 62.33) |
| Bangladesh | 30 to 34 | 89.95 (88.84, 91.08) |
| Bangladesh | 35 to 39 | 148.78 (147.05, 150.54) |
| Barbados | 15 to 19 | 24.99 (14.6, 42.77) |
| Barbados | 20 to 24 | 27.56 (17.12, 44.36) |
| Barbados | 25 to 29 | 31.36 (19.86, 49.53) |
| Barbados | 30 to 34 | 38.43 (24.94, 59.22) |
| Barbados | 35 to 39 | 50.37 (32.85, 77.25) |
| Belarus | 15 to 19 | 39.19 (36.5, 42.08) |
| Belarus | 20 to 24 | 39.02 (36.48, 41.73) |
| Belarus | 25 to 29 | 42.42 (39.8, 45.22) |
| Belarus | 30 to 34 | 54.78 (51.58, 58.18) |
| Belarus | 35 to 39 | 81.23 (76.63, 86.09) |
| Belgium | 15 to 19 | 55.12 (51.65, 58.83) |
| Belgium | 20 to 24 | 60.02 (56.59, 63.65) |
| Belgium | 25 to 29 | 60.64 (57.29, 64.19) |
| Belgium | 30 to 34 | 60.78 (57.42, 64.33) |
| Belgium | 35 to 39 | 65.2 (61.5, 69.12) |
| Belize | 15 to 19 | 52.54 (37.34, 73.94) |
| Belize | 20 to 24 | 61.05 (44.88, 83.06) |
| Belize | 25 to 29 | 67.14 (49.83, 90.45) |
| Belize | 30 to 34 | 79 (59.02, 105.76) |
| Belize | 35 to 39 | 101.69 (76.38, 135.39) |
| Benin | 15 to 19 | 42.14 (39.09, 45.43) |
| Benin | 20 to 24 | 50.99 (47.73, 54.47) |
| Benin | 25 to 29 | 62.61 (58.91, 66.55) |
| Benin | 30 to 34 | 86.83 (81.93, 92.02) |
| Benin | 35 to 39 | 127.17 (120.19, 134.55) |
| Bermuda | 15 to 19 | 54.33 (23.42, 126.05) |
| Bermuda | 20 to 24 | 55.39 (25.86, 118.62) |
| Bermuda | 25 to 29 | 61.48 (30.96, 122.09) |
| Bermuda | 30 to 34 | 65.02 (33.21, 127.32) |
| Bermuda | 35 to 39 | 81.51 (41.85, 158.75) |
| Bhutan | 15 to 19 | 32.3 (25.14, 41.5) |
| Bhutan | 20 to 24 | 35.31 (28.04, 44.47) |
| Bhutan | 25 to 29 | 42.2 (33.77, 52.74) |
| Bhutan | 30 to 34 | 66 (53.65, 81.21) |
| Bhutan | 35 to 39 | 112.28 (92.51, 136.28) |
| Bolivia (Plurinational State of) | 15 to 19 | 82.21 (78.35, 86.27) |
| Bolivia (Plurinational State of) | 20 to 24 | 93.79 (89.74, 98.02) |
| Bolivia (Plurinational State of) | 25 to 29 | 101.91 (97.62, 106.4) |
| Bolivia (Plurinational State of) | 30 to 34 | 116.03 (111.15, 121.12) |
| Bolivia (Plurinational State of) | 35 to 39 | 144.87 (138.78, 151.24) |
| Bosnia and Herzegovina | 15 to 19 | 27.75 (24.41, 31.55) |
| Bosnia and Herzegovina | 20 to 24 | 28.1 (24.85, 31.78) |
| Bosnia and Herzegovina | 25 to 29 | 30.8 (27.4, 34.62) |
| Bosnia and Herzegovina | 30 to 34 | 39.34 (35.15, 44.02) |
| Bosnia and Herzegovina | 35 to 39 | 56.75 (50.79, 63.41) |
| Botswana | 15 to 19 | 41.84 (36.5, 47.97) |
| Botswana | 20 to 24 | 50.34 (44.61, 56.8) |
| Botswana | 25 to 29 | 63.98 (57.26, 71.5) |
| Botswana | 30 to 34 | 102.22 (92.45, 113.03) |
| Botswana | 35 to 39 | 165.73 (150.79, 182.15) |
| Brazil | 15 to 19 | 111.2 (110.2, 112.21) |
| Brazil | 20 to 24 | 123.06 (122.05, 124.09) |
| Brazil | 25 to 29 | 130.27 (129.22, 131.33) |
| Brazil | 30 to 34 | 141.25 (140.11, 142.4) |
| Brazil | 35 to 39 | 164.74 (163.39, 166.1) |
| Brunei Darussalam | 15 to 19 | 59.22 (43.92, 79.84) |
| Brunei Darussalam | 20 to 24 | 61.88 (47.25, 81.03) |
| Brunei Darussalam | 25 to 29 | 59.56 (45.82, 77.42) |
| Brunei Darussalam | 30 to 34 | 58.15 (44.63, 75.76) |
| Brunei Darussalam | 35 to 39 | 59.77 (45.28, 78.91) |
| Bulgaria | 15 to 19 | 27.46 (24.93, 30.23) |
| Bulgaria | 20 to 24 | 27.84 (25.44, 30.46) |
| Bulgaria | 25 to 29 | 30.48 (27.97, 33.22) |
| Bulgaria | 30 to 34 | 38.64 (35.61, 41.92) |
| Bulgaria | 35 to 39 | 55.86 (51.61, 60.45) |
| Burkina Faso | 15 to 19 | 39.71 (37.57, 41.98) |
| Burkina Faso | 20 to 24 | 49.09 (46.7, 51.6) |
| Burkina Faso | 25 to 29 | 60.71 (57.91, 63.65) |
| Burkina Faso | 30 to 34 | 85.61 (81.9, 89.49) |
| Burkina Faso | 35 to 39 | 130.62 (125.21, 136.26) |
| Burundi | 15 to 19 | 20.62 (18.55, 22.92) |
| Burundi | 20 to 24 | 24.5 (22.25, 26.98) |
| Burundi | 25 to 29 | 31.41 (28.69, 34.39) |
| Burundi | 30 to 34 | 48.09 (44.25, 52.27) |
| Burundi | 35 to 39 | 76.48 (70.74, 82.7) |
| Cabo Verde | 15 to 19 | 29.64 (21.18, 41.49) |
| Cabo Verde | 20 to 24 | 35.29 (25.99, 47.93) |
| Cabo Verde | 25 to 29 | 43.5 (32.59, 58.05) |
| Cabo Verde | 30 to 34 | 62.43 (47.82, 81.5) |
| Cabo Verde | 35 to 39 | 92.29 (71.18, 119.66) |
| Cambodia | 15 to 19 | 114.88 (110.75, 119.16) |
| Cambodia | 20 to 24 | 100.63 (97.07, 104.32) |
| Cambodia | 25 to 29 | 96.9 (93.45, 100.48) |
| Cambodia | 30 to 34 | 109.44 (105.56, 113.47) |
| Cambodia | 35 to 39 | 146.69 (141.5, 152.07) |
| Cameroon | 15 to 19 | 37.38 (35.48, 39.39) |
| Cameroon | 20 to 24 | 43.91 (41.91, 46.01) |
| Cameroon | 25 to 29 | 51.31 (49.09, 53.64) |
| Cameroon | 30 to 34 | 68.01 (65.19, 70.95) |
| Cameroon | 35 to 39 | 96.41 (92.53, 100.46) |
| Canada | 15 to 19 | 41.66 (39.93, 43.46) |
| Canada | 20 to 24 | 45.29 (43.56, 47.08) |
| Canada | 25 to 29 | 44.67 (43, 46.41) |
| Canada | 30 to 34 | 43.54 (41.9, 45.23) |
| Canada | 35 to 39 | 46.3 (44.52, 48.16) |
| Central African Republic | 15 to 19 | 29.45 (26.2, 33.09) |
| Central African Republic | 20 to 24 | 33.95 (30.5, 37.8) |
| Central African Republic | 25 to 29 | 37.6 (33.82, 41.81) |
| Central African Republic | 30 to 34 | 48.22 (43.45, 53.51) |
| Central African Republic | 35 to 39 | 69.76 (62.94, 77.32) |
| Chad | 15 to 19 | 46.8 (43.88, 49.92) |
| Chad | 20 to 24 | 56.15 (52.98, 59.52) |
| Chad | 25 to 29 | 66.27 (62.66, 70.08) |
| Chad | 30 to 34 | 87.66 (83.05, 92.53) |
| Chad | 35 to 39 | 124.21 (117.83, 130.94) |
| Chile | 15 to 19 | 71.06 (68.16, 74.07) |
| Chile | 20 to 24 | 72.7 (69.92, 75.59) |
| Chile | 25 to 29 | 70.77 (68.08, 73.57) |
| Chile | 30 to 34 | 69.56 (66.85, 72.37) |
| Chile | 35 to 39 | 74.55 (71.54, 77.69) |
| China | 15 to 19 | 38.76 (37.6, 39.94) |
| China | 20 to 24 | 35.52 (34.52, 36.55) |
| China | 25 to 29 | 36.12 (35.14, 37.14) |
| China | 30 to 34 | 45.69 (44.51, 46.91) |
| China | 35 to 39 | 66.63 (64.95, 68.35) |
| Colombia | 15 to 19 | 72.07 (70.39, 73.79) |
| Colombia | 20 to 24 | 88 (86.16, 89.88) |
| Colombia | 25 to 29 | 96.77 (94.81, 98.78) |
| Colombia | 30 to 34 | 109.1 (106.9, 111.35) |
| Colombia | 35 to 39 | 135.54 (132.79, 138.35) |
| Comoros | 15 to 19 | 32.21 (24.26, 42.77) |
| Comoros | 20 to 24 | 38.53 (29.89, 49.68) |
| Comoros | 25 to 29 | 48.68 (38.43, 61.66) |
| Comoros | 30 to 34 | 72.98 (58.59, 90.91) |
| Comoros | 35 to 39 | 112.7 (91.36, 139.02) |
| Congo | 15 to 19 | 32.65 (29.05, 36.7) |
| Congo | 20 to 24 | 38.15 (34.4, 42.32) |
| Congo | 25 to 29 | 45.74 (41.51, 50.4) |
| Congo | 30 to 34 | 62.32 (56.9, 68.25) |
| Congo | 35 to 39 | 90.68 (83.12, 98.93) |
| Cook Islands | 15 to 19 | 56.31 (16.07, 197.38) |
| Cook Islands | 20 to 24 | 58.63 (17.95, 191.5) |
| Cook Islands | 25 to 29 | 63.99 (19.69, 207.92) |
| Cook Islands | 30 to 34 | 76.46 (23.6, 247.71) |
| Cook Islands | 35 to 39 | 82.07 (23.63, 285.06) |
| Costa Rica | 15 to 19 | 63.5 (58.6, 68.81) |
| Costa Rica | 20 to 24 | 78.61 (73.2, 84.43) |
| Costa Rica | 25 to 29 | 87.51 (81.72, 93.71) |
| Costa Rica | 30 to 34 | 99.2 (92.73, 106.13) |
| Costa Rica | 35 to 39 | 123.6 (115.48, 132.3) |
| Croatia | 15 to 19 | 27.15 (23.83, 30.94) |
| Croatia | 20 to 24 | 27.33 (24.18, 30.89) |
| Croatia | 25 to 29 | 29.67 (26.36, 33.4) |
| Croatia | 30 to 34 | 37.41 (33.44, 41.86) |
| Croatia | 35 to 39 | 54.04 (48.49, 60.22) |
| Cuba | 15 to 19 | 70.38 (67.11, 73.81) |
| Cuba | 20 to 24 | 79.35 (76.05, 82.79) |
| Cuba | 25 to 29 | 87.54 (84.08, 91.14) |
| Cuba | 30 to 34 | 102.58 (98.62, 106.7) |
| Cuba | 35 to 39 | 131.07 (125.95, 136.41) |
| Cyprus | 15 to 19 | 56.53 (46.6, 68.56) |
| Cyprus | 20 to 24 | 61.56 (51.75, 73.23) |
| Cyprus | 25 to 29 | 61.45 (52.02, 72.6) |
| Cyprus | 30 to 34 | 61.02 (51.67, 72.05) |
| Cyprus | 35 to 39 | 64.34 (54.26, 76.29) |
| Czechia | 15 to 19 | 26.82 (24.7, 29.12) |
| Czechia | 20 to 24 | 27.04 (25.05, 29.18) |
| Czechia | 25 to 29 | 29.36 (27.31, 31.56) |
| Czechia | 30 to 34 | 37.02 (34.57, 39.64) |
| Czechia | 35 to 39 | 53.07 (49.73, 56.64) |
| Côte d'Ivoire | 15 to 19 | 39.69 (37.67, 41.83) |
| Côte d'Ivoire | 20 to 24 | 48.34 (46.19, 50.58) |
| Côte d'Ivoire | 25 to 29 | 59.63 (57.17, 62.18) |
| Côte d'Ivoire | 30 to 34 | 84.11 (80.83, 87.52) |
| Côte d'Ivoire | 35 to 39 | 128.06 (123.16, 133.16) |
| Democratic People's Republic of Korea | 15 to 19 | 21.78 (20.46, 23.19) |
| Democratic People's Republic of Korea | 20 to 24 | 19.12 (18.02, 20.3) |
| Democratic People's Republic of Korea | 25 to 29 | 19.46 (18.38, 20.62) |
| Democratic People's Republic of Korea | 30 to 34 | 27.27 (25.86, 28.75) |
| Democratic People's Republic of Korea | 35 to 39 | 43.44 (41.33, 45.66) |
| Democratic Republic of the Congo | 15 to 19 | 24.68 (23.83, 25.57) |
| Democratic Republic of the Congo | 20 to 24 | 28.92 (28.02, 29.86) |
| Democratic Republic of the Congo | 25 to 29 | 33.63 (32.61, 34.67) |
| Democratic Republic of the Congo | 30 to 34 | 44.52 (43.23, 45.85) |
| Democratic Republic of the Congo | 35 to 39 | 64.35 (62.53, 66.22) |
| Denmark | 15 to 19 | 53.89 (49.27, 58.95) |
| Denmark | 20 to 24 | 58.99 (54.43, 63.93) |
| Denmark | 25 to 29 | 59.72 (55.23, 64.56) |
| Denmark | 30 to 34 | 60.04 (55.48, 64.97) |
| Denmark | 35 to 39 | 64.47 (59.41, 69.95) |
| Djibouti | 15 to 19 | 35.57 (27.72, 45.64) |
| Djibouti | 20 to 24 | 41.09 (33.03, 51.13) |
| Djibouti | 25 to 29 | 51.06 (41.89, 62.24) |
| Djibouti | 30 to 34 | 73.78 (61.58, 88.4) |
| Djibouti | 35 to 39 | 113.36 (95.87, 134.05) |
| Dominica | 15 to 19 | 44.81 (22.01, 91.23) |
| Dominica | 20 to 24 | 55.58 (28.79, 107.27) |
| Dominica | 25 to 29 | 62.54 (32.8, 119.25) |
| Dominica | 30 to 34 | 68.75 (36.2, 130.59) |
| Dominica | 35 to 39 | 89.79 (47.28, 170.52) |
| Dominican Republic | 15 to 19 | 69.21 (65.79, 72.81) |
| Dominican Republic | 20 to 24 | 78.39 (74.84, 82.1) |
| Dominican Republic | 25 to 29 | 85.31 (81.53, 89.27) |
| Dominican Republic | 30 to 34 | 98.49 (94.15, 103.02) |
| Dominican Republic | 35 to 39 | 124.51 (119.02, 130.26) |
| Ecuador | 15 to 19 | 57.6 (55.01, 60.32) |
| Ecuador | 20 to 24 | 66.65 (63.91, 69.51) |
| Ecuador | 25 to 29 | 73.41 (70.47, 76.48) |
| Ecuador | 30 to 34 | 84.36 (81.01, 87.85) |
| Ecuador | 35 to 39 | 106.25 (102.04, 110.65) |
| Egypt | 15 to 19 | 97.12 (95.61, 98.66) |
| Egypt | 20 to 24 | 101.92 (100.43, 103.44) |
| Egypt | 25 to 29 | 101.34 (99.85, 102.85) |
| Egypt | 30 to 34 | 102.98 (101.42, 104.56) |
| Egypt | 35 to 39 | 113.9 (112.11, 115.73) |
| El Salvador | 15 to 19 | 84.93 (80.44, 89.67) |
| El Salvador | 20 to 24 | 104.56 (99.47, 109.91) |
| El Salvador | 25 to 29 | 115.09 (109.5, 120.96) |
| El Salvador | 30 to 34 | 129.32 (122.93, 136.04) |
| El Salvador | 35 to 39 | 160.17 (152.12, 168.64) |
| Equatorial Guinea | 15 to 19 | 60.5 (48.93, 74.82) |
| Equatorial Guinea | 20 to 24 | 69.5 (57.67, 83.75) |
| Equatorial Guinea | 25 to 29 | 82.48 (69.31, 98.15) |
| Equatorial Guinea | 30 to 34 | 101.34 (85.6, 119.96) |
| Equatorial Guinea | 35 to 39 | 124.72 (105.35, 147.65) |
| Eritrea | 15 to 19 | 42.2 (38.5, 46.26) |
| Eritrea | 20 to 24 | 49.62 (45.73, 53.83) |
| Eritrea | 25 to 29 | 61.92 (57.39, 66.82) |
| Eritrea | 30 to 34 | 90.19 (84.03, 96.8) |
| Eritrea | 35 to 39 | 138.39 (129.33, 148.08) |
| Estonia | 15 to 19 | 26.37 (20.88, 33.31) |
| Estonia | 20 to 24 | 26.57 (21.33, 33.1) |
| Estonia | 25 to 29 | 29.37 (23.85, 36.17) |
| Estonia | 30 to 34 | 39.22 (32.24, 47.7) |
| Estonia | 35 to 39 | 58.7 (48.61, 70.89) |
| Eswatini | 15 to 19 | 43.44 (36.41, 51.82) |
| Eswatini | 20 to 24 | 52.11 (44.36, 61.22) |
| Eswatini | 25 to 29 | 65.91 (56.53, 76.83) |
| Eswatini | 30 to 34 | 104.68 (90.69, 120.82) |
| Eswatini | 35 to 39 | 168.66 (146.77, 193.81) |
| Ethiopia | 15 to 19 | 40.22 (39.33, 41.12) |
| Ethiopia | 20 to 24 | 54.73 (53.68, 55.81) |
| Ethiopia | 25 to 29 | 74.85 (73.5, 76.22) |
| Ethiopia | 30 to 34 | 104.66 (102.87, 106.48) |
| Ethiopia | 35 to 39 | 153.28 (150.74, 155.87) |
| Fiji | 15 to 19 | 40 (32.28, 49.57) |
| Fiji | 20 to 24 | 43 (35.15, 52.62) |
| Fiji | 25 to 29 | 47.66 (39.16, 58.01) |
| Fiji | 30 to 34 | 60.65 (50.2, 73.27) |
| Fiji | 35 to 39 | 89.97 (74.93, 108.03) |
| Finland | 15 to 19 | 55.46 (50.6, 60.78) |
| Finland | 20 to 24 | 60.58 (55.69, 65.89) |
| Finland | 25 to 29 | 61.09 (56.28, 66.31) |
| Finland | 30 to 34 | 61.02 (56.19, 66.27) |
| Finland | 35 to 39 | 64.61 (59.31, 70.39) |
| France | 15 to 19 | 51.95 (50.58, 53.35) |
| France | 20 to 24 | 56.08 (54.72, 57.48) |
| France | 25 to 29 | 56.29 (54.95, 57.67) |
| France | 30 to 34 | 56.12 (54.78, 57.5) |
| France | 35 to 39 | 59.51 (58.04, 61.03) |
| Gabon | 15 to 19 | 29.8 (24.32, 36.53) |
| Gabon | 20 to 24 | 34.97 (29.17, 41.92) |
| Gabon | 25 to 29 | 41.74 (35.19, 49.49) |
| Gabon | 30 to 34 | 57.58 (49.02, 67.63) |
| Gabon | 35 to 39 | 84.5 (72.29, 98.78) |
| Gambia | 15 to 19 | 45.18 (38.54, 52.95) |
| Gambia | 20 to 24 | 52.5 (45.59, 60.47) |
| Gambia | 25 to 29 | 63.06 (55.16, 72.1) |
| Gambia | 30 to 34 | 84.95 (74.71, 96.6) |
| Gambia | 35 to 39 | 121.1 (106.8, 137.32) |
| Georgia | 15 to 19 | 40.07 (36.47, 44.02) |
| Georgia | 20 to 24 | 35.73 (32.63, 39.12) |
| Georgia | 25 to 29 | 34.25 (31.34, 37.43) |
| Georgia | 30 to 34 | 38.04 (34.9, 41.47) |
| Georgia | 35 to 39 | 49.93 (45.85, 54.38) |
| Germany | 15 to 19 | 53.56 (52.24, 54.91) |
| Germany | 20 to 24 | 58.6 (57.32, 59.92) |
| Germany | 25 to 29 | 59.17 (57.91, 60.44) |
| Germany | 30 to 34 | 59.23 (57.98, 60.52) |
| Germany | 35 to 39 | 63.2 (61.81, 64.62) |
| Ghana | 15 to 19 | 40.55 (38.83, 42.35) |
| Ghana | 20 to 24 | 48.48 (46.64, 50.38) |
| Ghana | 25 to 29 | 58.48 (56.41, 60.64) |
| Ghana | 30 to 34 | 79.01 (76.35, 81.77) |
| Ghana | 35 to 39 | 113.95 (110.21, 117.82) |
| Greece | 15 to 19 | 48.12 (45.16, 51.27) |
| Greece | 20 to 24 | 52.14 (49.23, 55.23) |
| Greece | 25 to 29 | 53.21 (50.33, 56.25) |
| Greece | 30 to 34 | 54.07 (51.12, 57.19) |
| Greece | 35 to 39 | 58.73 (55.42, 62.24) |
| Greenland | 15 to 19 | 58.43 (24.29, 140.55) |
| Greenland | 20 to 24 | 59.54 (26.07, 135.98) |
| Greenland | 25 to 29 | 58.85 (26.28, 131.75) |
| Greenland | 30 to 34 | 59.14 (26.28, 133.09) |
| Greenland | 35 to 39 | 62.52 (26.5, 147.49) |
| Grenada | 15 to 19 | 52.73 (30.67, 90.68) |
| Grenada | 20 to 24 | 58.5 (35.9, 95.33) |
| Grenada | 25 to 29 | 62.9 (38.82, 101.9) |
| Grenada | 30 to 34 | 76.66 (46.85, 125.46) |
| Grenada | 35 to 39 | 97.81 (58.62, 163.21) |
| Guam | 15 to 19 | 31.28 (17.04, 57.41) |
| Guam | 20 to 24 | 30.59 (17.5, 53.45) |
| Guam | 25 to 29 | 33.47 (19.36, 57.88) |
| Guam | 30 to 34 | 43.45 (25.79, 73.2) |
| Guam | 35 to 39 | 64.35 (38.7, 107.03) |
| Guatemala | 15 to 19 | 95.07 (91.45, 98.84) |
| Guatemala | 20 to 24 | 116.43 (112.43, 120.56) |
| Guatemala | 25 to 29 | 128.14 (123.85, 132.57) |
| Guatemala | 30 to 34 | 144.4 (139.57, 149.39) |
| Guatemala | 35 to 39 | 180 (173.96, 186.24) |
| Guinea | 15 to 19 | 47.73 (44.7, 50.96) |
| Guinea | 20 to 24 | 57.24 (53.96, 60.72) |
| Guinea | 25 to 29 | 69.96 (66.11, 74.02) |
| Guinea | 30 to 34 | 95.82 (90.79, 101.13) |
| Guinea | 35 to 39 | 138.76 (131.64, 146.25) |
| Guinea-Bissau | 15 to 19 | 45.01 (38.32, 52.86) |
| Guinea-Bissau | 20 to 24 | 54.13 (46.82, 62.57) |
| Guinea-Bissau | 25 to 29 | 66.33 (57.8, 76.12) |
| Guinea-Bissau | 30 to 34 | 91.4 (80.07, 104.33) |
| Guinea-Bissau | 35 to 39 | 134.55 (118.03, 153.39) |
| Guyana | 15 to 19 | 50.05 (41.23, 60.75) |
| Guyana | 20 to 24 | 58.46 (48.86, 69.95) |
| Guyana | 25 to 29 | 65.05 (54.45, 77.72) |
| Guyana | 30 to 34 | 77.42 (64.87, 92.4) |
| Guyana | 35 to 39 | 100.24 (83.84, 119.86) |
| Haiti | 15 to 19 | 60.35 (57.09, 63.81) |
| Haiti | 20 to 24 | 70.28 (66.83, 73.92) |
| Haiti | 25 to 29 | 78.94 (75.21, 82.86) |
| Haiti | 30 to 34 | 95.2 (90.79, 99.82) |
| Haiti | 35 to 39 | 124.69 (118.96, 130.71) |
| Honduras | 15 to 19 | 70.41 (66.43, 74.62) |
| Honduras | 20 to 24 | 87.23 (82.74, 91.96) |
| Honduras | 25 to 29 | 96.19 (91.33, 101.3) |
| Honduras | 30 to 34 | 108.56 (103.05, 114.36) |
| Honduras | 35 to 39 | 135.33 (128.45, 142.59) |
| Hungary | 15 to 19 | 27.52 (25.34, 29.9) |
| Hungary | 20 to 24 | 28.04 (25.97, 30.28) |
| Hungary | 25 to 29 | 30.91 (28.72, 33.28) |
| Hungary | 30 to 34 | 39.55 (36.9, 42.39) |
| Hungary | 35 to 39 | 57.19 (53.55, 61.08) |
| Iceland | 15 to 19 | 51.64 (35.75, 74.6) |
| Iceland | 20 to 24 | 57.12 (40.85, 79.88) |
| Iceland | 25 to 29 | 58.42 (42.1, 81.08) |
| Iceland | 30 to 34 | 58.97 (42.43, 81.96) |
| Iceland | 35 to 39 | 65.12 (46.4, 91.4) |
| India | 15 to 19 | 65.57 (64.98, 66.17) |
| India | 20 to 24 | 69.62 (69.05, 70.19) |
| India | 25 to 29 | 79.67 (79.06, 80.29) |
| India | 30 to 34 | 113.5 (112.69, 114.31) |
| India | 35 to 39 | 183.89 (182.66, 185.13) |
| Indonesia | 15 to 19 | 83.77 (82.99, 84.55) |
| Indonesia | 20 to 24 | 89.9 (89.14, 90.67) |
| Indonesia | 25 to 29 | 99.27 (98.46, 100.08) |
| Indonesia | 30 to 34 | 123.73 (122.77, 124.69) |
| Indonesia | 35 to 39 | 177.88 (176.55, 179.22) |
| Iran (Islamic Republic of) | 15 to 19 | 111.8 (110.23, 113.39) |
| Iran (Islamic Republic of) | 20 to 24 | 118.84 (117.26, 120.44) |
| Iran (Islamic Republic of) | 25 to 29 | 121.39 (119.8, 122.99) |
| Iran (Islamic Republic of) | 30 to 34 | 125.47 (123.81, 127.15) |
| Iran (Islamic Republic of) | 35 to 39 | 138.49 (136.58, 140.43) |
| Iraq | 15 to 19 | 86.84 (84.52, 89.22) |
| Iraq | 20 to 24 | 92.02 (89.77, 94.34) |
| Iraq | 25 to 29 | 92.43 (90.19, 94.73) |
| Iraq | 30 to 34 | 95.23 (92.84, 97.68) |
| Iraq | 35 to 39 | 106.01 (103.26, 108.85) |
| Ireland | 15 to 19 | 56.32 (51.49, 61.61) |
| Ireland | 20 to 24 | 61.08 (56.15, 66.45) |
| Ireland | 25 to 29 | 61.4 (56.57, 66.65) |
| Ireland | 30 to 34 | 61.31 (56.53, 66.51) |
| Ireland | 35 to 39 | 65.46 (60.23, 71.14) |
| Israel | 15 to 19 | 55.54 (51.71, 59.65) |
| Israel | 20 to 24 | 60.66 (56.81, 64.78) |
| Israel | 25 to 29 | 61.25 (57.41, 65.35) |
| Israel | 30 to 34 | 61.22 (57.33, 65.36) |
| Israel | 35 to 39 | 65.37 (61.09, 69.94) |
| Italy | 15 to 19 | 86.31 (84.48, 88.19) |
| Italy | 20 to 24 | 93.89 (92.09, 95.72) |
| Italy | 25 to 29 | 96 (94.25, 97.79) |
| Italy | 30 to 34 | 98.28 (96.5, 100.1) |
| Italy | 35 to 39 | 107.19 (105.21, 109.21) |
| Jamaica | 15 to 19 | 48.63 (43.57, 54.29) |
| Jamaica | 20 to 24 | 56.05 (50.67, 62) |
| Jamaica | 25 to 29 | 61.81 (55.98, 68.24) |
| Jamaica | 30 to 34 | 72.06 (65.3, 79.53) |
| Jamaica | 35 to 39 | 91.15 (82.51, 100.7) |
| Japan | 15 to 19 | 47.08 (46.19, 47.99) |
| Japan | 20 to 24 | 48.24 (47.38, 49.1) |
| Japan | 25 to 29 | 48.18 (47.35, 49.03) |
| Japan | 30 to 34 | 49.05 (48.19, 49.92) |
| Japan | 35 to 39 | 53.72 (52.76, 54.7) |
| Jordan | 15 to 19 | 64.13 (59.93, 68.63) |
| Jordan | 20 to 24 | 67.8 (63.71, 72.16) |
| Jordan | 25 to 29 | 68.18 (64.07, 72.55) |
| Jordan | 30 to 34 | 70.6 (66.28, 75.19) |
| Jordan | 35 to 39 | 79.09 (74.23, 84.28) |
| Kazakhstan | 15 to 19 | 47.41 (45.21, 49.71) |
| Kazakhstan | 20 to 24 | 47.71 (45.6, 49.91) |
| Kazakhstan | 25 to 29 | 51.77 (49.56, 54.09) |
| Kazakhstan | 30 to 34 | 64.1 (61.46, 66.86) |
| Kazakhstan | 35 to 39 | 91.15 (87.48, 94.96) |
| Kenya | 15 to 19 | 43.55 (42.16, 44.99) |
| Kenya | 20 to 24 | 51.15 (49.68, 52.67) |
| Kenya | 25 to 29 | 62.36 (60.65, 64.11) |
| Kenya | 30 to 34 | 85.25 (83.02, 87.54) |
| Kenya | 35 to 39 | 124.01 (120.87, 127.25) |
| Kiribati | 15 to 19 | 39.51 (20.07, 77.81) |
| Kiribati | 20 to 24 | 42.64 (22.68, 80.17) |
| Kiribati | 25 to 29 | 48.85 (26.46, 90.18) |
| Kiribati | 30 to 34 | 63.31 (35.22, 113.79) |
| Kiribati | 35 to 39 | 88.82 (50.35, 156.66) |
| Kuwait | 15 to 19 | 74.99 (66.96, 83.98) |
| Kuwait | 20 to 24 | 79.14 (72.04, 86.93) |
| Kuwait | 25 to 29 | 79.22 (72.99, 85.99) |
| Kuwait | 30 to 34 | 80.88 (74.85, 87.39) |
| Kuwait | 35 to 39 | 90.53 (83.83, 97.75) |
| Kyrgyzstan | 15 to 19 | 35.96 (32.78, 39.45) |
| Kyrgyzstan | 20 to 24 | 38.06 (34.92, 41.48) |
| Kyrgyzstan | 25 to 29 | 42.66 (39.26, 46.35) |
| Kyrgyzstan | 30 to 34 | 55.4 (51.13, 60.03) |
| Kyrgyzstan | 35 to 39 | 81.83 (75.66, 88.51) |
| Lao People's Democratic Republic | 15 to 19 | 46.13 (42.42, 50.17) |
| Lao People's Democratic Republic | 20 to 24 | 42.99 (39.64, 46.62) |
| Lao People's Democratic Republic | 25 to 29 | 43.72 (40.31, 47.42) |
| Lao People's Democratic Republic | 30 to 34 | 52.9 (48.86, 57.27) |
| Lao People's Democratic Republic | 35 to 39 | 76.85 (71.13, 83.02) |
| Latvia | 15 to 19 | 38.3 (32.92, 44.56) |
| Latvia | 20 to 24 | 37.98 (32.92, 43.82) |
| Latvia | 25 to 29 | 41.39 (36.05, 47.51) |
| Latvia | 30 to 34 | 53.14 (46.59, 60.61) |
| Latvia | 35 to 39 | 78.5 (69, 89.31) |
| Lebanon | 15 to 19 | 103.62 (96.7, 111.04) |
| Lebanon | 20 to 24 | 103.38 (96.8, 110.4) |
| Lebanon | 25 to 29 | 100.07 (93.73, 106.85) |
| Lebanon | 30 to 34 | 100.06 (93.58, 106.99) |
| Lebanon | 35 to 39 | 108.8 (101.48, 116.65) |
| Lesotho | 15 to 19 | 45.37 (40.23, 51.17) |
| Lesotho | 20 to 24 | 55.06 (49.29, 61.51) |
| Lesotho | 25 to 29 | 70.66 (63.6, 78.51) |
| Lesotho | 30 to 34 | 112.62 (102.09, 124.24) |
| Lesotho | 35 to 39 | 183.03 (166.49, 201.21) |
| Liberia | 15 to 19 | 44.02 (39.35, 49.24) |
| Liberia | 20 to 24 | 52.63 (47.69, 58.08) |
| Liberia | 25 to 29 | 62.83 (57.3, 68.89) |
| Liberia | 30 to 34 | 84.52 (77.42, 92.26) |
| Liberia | 35 to 39 | 119.5 (109.92, 129.93) |
| Libya | 15 to 19 | 82.47 (77.76, 87.48) |
| Libya | 20 to 24 | 87.5 (82.84, 92.42) |
| Libya | 25 to 29 | 88.26 (83.65, 93.12) |
| Libya | 30 to 34 | 91.35 (86.55, 96.4) |
| Libya | 35 to 39 | 102.43 (96.92, 108.26) |
| Lithuania | 15 to 19 | 38.53 (34.05, 43.6) |
| Lithuania | 20 to 24 | 38.45 (34.2, 43.23) |
| Lithuania | 25 to 29 | 41.78 (37.29, 46.82) |
| Lithuania | 30 to 34 | 53.9 (48.34, 60.1) |
| Lithuania | 35 to 39 | 79.64 (71.56, 88.64) |
| Luxembourg | 15 to 19 | 54.21 (39.03, 75.3) |
| Luxembourg | 20 to 24 | 58.72 (44.14, 78.11) |
| Luxembourg | 25 to 29 | 58.89 (45.13, 76.85) |
| Luxembourg | 30 to 34 | 59.6 (45.96, 77.28) |
| Luxembourg | 35 to 39 | 63.92 (49.22, 83.02) |
| Madagascar | 15 to 19 | 28.73 (27.14, 30.41) |
| Madagascar | 20 to 24 | 33.66 (32, 35.4) |
| Madagascar | 25 to 29 | 42.71 (40.75, 44.76) |
| Madagascar | 30 to 34 | 64.2 (61.47, 67.04) |
| Madagascar | 35 to 39 | 101.67 (97.59, 105.92) |
| Malawi | 15 to 19 | 39.53 (37.33, 41.86) |
| Malawi | 20 to 24 | 46.26 (43.96, 48.68) |
| Malawi | 25 to 29 | 58.66 (55.94, 61.52) |
| Malawi | 30 to 34 | 85.93 (82.14, 89.89) |
| Malawi | 35 to 39 | 132.44 (126.77, 138.37) |
| Malaysia | 15 to 19 | 60.53 (58.44, 62.69) |
| Malaysia | 20 to 24 | 57.41 (55.55, 59.33) |
| Malaysia | 25 to 29 | 58.33 (56.48, 60.23) |
| Malaysia | 30 to 34 | 69.43 (67.3, 71.63) |
| Malaysia | 35 to 39 | 98.2 (95.25, 101.24) |
| Maldives | 15 to 19 | 51.17 (36.48, 71.77) |
| Maldives | 20 to 24 | 45.66 (32.98, 63.22) |
| Maldives | 25 to 29 | 44.75 (32.37, 61.84) |
| Maldives | 30 to 34 | 51.92 (37.91, 71.09) |
| Maldives | 35 to 39 | 76.12 (56.46, 102.64) |
| Mali | 15 to 19 | 62.43 (59.46, 65.54) |
| Mali | 20 to 24 | 73.98 (70.82, 77.28) |
| Mali | 25 to 29 | 88.39 (84.78, 92.16) |
| Mali | 30 to 34 | 116.69 (112.11, 121.45) |
| Mali | 35 to 39 | 163.82 (157.56, 170.32) |
| Malta | 15 to 19 | 55.65 (41.07, 75.41) |
| Malta | 20 to 24 | 61.49 (46.48, 81.33) |
| Malta | 25 to 29 | 62.31 (47.39, 81.94) |
| Malta | 30 to 34 | 61.55 (46.66, 81.19) |
| Malta | 35 to 39 | 66 (49.56, 87.91) |
| Marshall Islands | 15 to 19 | 35.55 (14.93, 84.63) |
| Marshall Islands | 20 to 24 | 41.17 (18.29, 92.65) |
| Marshall Islands | 25 to 29 | 45.78 (20.57, 101.91) |
| Marshall Islands | 30 to 34 | 58.74 (26.66, 129.42) |
| Marshall Islands | 35 to 39 | 82.72 (38.23, 179) |
| Mauritania | 15 to 19 | 41.23 (36.56, 46.51) |
| Mauritania | 20 to 24 | 49.15 (44.11, 54.76) |
| Mauritania | 25 to 29 | 59.64 (53.84, 66.06) |
| Mauritania | 30 to 34 | 81.05 (73.53, 89.33) |
| Mauritania | 35 to 39 | 116.78 (106.19, 128.42) |
| Mauritius | 15 to 19 | 56.63 (48.07, 66.71) |
| Mauritius | 20 to 24 | 50.89 (43.46, 59.6) |
| Mauritius | 25 to 29 | 50.21 (42.94, 58.72) |
| Mauritius | 30 to 34 | 59.31 (50.94, 69.06) |
| Mauritius | 35 to 39 | 84.97 (73.28, 98.53) |
| Mexico | 15 to 19 | 66.99 (66.03, 67.96) |
| Mexico | 20 to 24 | 83.42 (82.35, 84.51) |
| Mexico | 25 to 29 | 94.08 (92.89, 95.28) |
| Mexico | 30 to 34 | 107.59 (106.26, 108.95) |
| Mexico | 35 to 39 | 132.47 (130.82, 134.15) |
| Micronesia (Federated States of) | 15 to 19 | 41.79 (23.35, 74.8) |
| Micronesia (Federated States of) | 20 to 24 | 43.18 (24.19, 77.08) |
| Micronesia (Federated States of) | 25 to 29 | 46.96 (26.26, 84) |
| Micronesia (Federated States of) | 30 to 34 | 57.98 (32.61, 103.08) |
| Micronesia (Federated States of) | 35 to 39 | 83.81 (47.19, 148.86) |
| Monaco | 15 to 19 | 71.7 (20.74, 247.89) |
| Monaco | 20 to 24 | 67.87 (21.2, 217.28) |
| Monaco | 25 to 29 | 57.63 (18.29, 181.63) |
| Monaco | 30 to 34 | 51.41 (15.98, 165.38) |
| Monaco | 35 to 39 | 62.25 (18.75, 206.71) |
| Mongolia | 15 to 19 | 52.71 (47.4, 58.61) |
| Mongolia | 20 to 24 | 54.3 (49.19, 59.95) |
| Mongolia | 25 to 29 | 59.08 (53.74, 64.96) |
| Mongolia | 30 to 34 | 73.35 (66.93, 80.38) |
| Mongolia | 35 to 39 | 103.7 (94.9, 113.31) |
| Montenegro | 15 to 19 | 27.27 (19.65, 37.84) |
| Montenegro | 20 to 24 | 27.77 (20.43, 37.75) |
| Montenegro | 25 to 29 | 30.12 (22.32, 40.66) |
| Montenegro | 30 to 34 | 38.26 (28.74, 50.93) |
| Montenegro | 35 to 39 | 55.62 (42.16, 73.37) |
| Morocco | 15 to 19 | 72.76 (70.8, 74.77) |
| Morocco | 20 to 24 | 77.32 (75.36, 79.32) |
| Morocco | 25 to 29 | 78.26 (76.29, 80.29) |
| Morocco | 30 to 34 | 81.44 (79.34, 83.6) |
| Morocco | 35 to 39 | 92.58 (90.11, 95.12) |
| Mozambique | 15 to 19 | 41.78 (39.89, 43.75) |
| Mozambique | 20 to 24 | 48.74 (46.78, 50.78) |
| Mozambique | 25 to 29 | 60.84 (58.54, 63.23) |
| Mozambique | 30 to 34 | 87.85 (84.73, 91.09) |
| Mozambique | 35 to 39 | 134.42 (129.79, 139.21) |
| Myanmar | 15 to 19 | 87.98 (86.2, 89.8) |
| Myanmar | 20 to 24 | 84.16 (82.54, 85.81) |
| Myanmar | 25 to 29 | 86.51 (84.86, 88.18) |
| Myanmar | 30 to 34 | 102.51 (100.61, 104.46) |
| Myanmar | 35 to 39 | 142.26 (139.66, 144.91) |
| Namibia | 15 to 19 | 41.45 (36.2, 47.45) |
| Namibia | 20 to 24 | 49.19 (43.63, 55.46) |
| Namibia | 25 to 29 | 62.4 (55.78, 69.8) |
| Namibia | 30 to 34 | 99.56 (89.79, 110.39) |
| Namibia | 35 to 39 | 162.4 (147.15, 179.24) |
| Nauru | 15 to 19 | 150.71 (34.94, 650.12) |
| Nauru | 20 to 24 | 37.85 (6.39, 224.18) |
| Nauru | 25 to 29 | 42.1 (7.15, 248) |
| Nauru | 30 to 34 | 52.99 (9.12, 307.97) |
| Nauru | 35 to 39 | 78.76 (14.09, 440.26) |
| Nepal | 15 to 19 | 47.2 (45.51, 48.95) |
| Nepal | 20 to 24 | 52.07 (50.37, 53.82) |
| Nepal | 25 to 29 | 63.86 (61.91, 65.88) |
| Nepal | 30 to 34 | 111.77 (108.71, 114.91) |
| Nepal | 35 to 39 | 206.62 (201.4, 211.97) |
| Netherlands | 15 to 19 | 47.67 (45.07, 50.41) |
| Netherlands | 20 to 24 | 51.74 (49.21, 54.41) |
| Netherlands | 25 to 29 | 52.14 (49.67, 54.74) |
| Netherlands | 30 to 34 | 51.89 (49.4, 54.5) |
| Netherlands | 35 to 39 | 55.11 (52.36, 58.02) |
| New Zealand | 15 to 19 | 58.62 (53.15, 64.65) |
| New Zealand | 20 to 24 | 57.53 (52.39, 63.18) |
| New Zealand | 25 to 29 | 55.82 (50.8, 61.34) |
| New Zealand | 30 to 34 | 55.42 (50.44, 60.89) |
| New Zealand | 35 to 39 | 59.57 (54.07, 65.63) |
| Nicaragua | 15 to 19 | 76.65 (72.05, 81.55) |
| Nicaragua | 20 to 24 | 93.7 (88.57, 99.12) |
| Nicaragua | 25 to 29 | 103.66 (98.08, 109.55) |
| Nicaragua | 30 to 34 | 117.68 (111.31, 124.41) |
| Nicaragua | 35 to 39 | 147.01 (138.96, 155.53) |
| Niger | 15 to 19 | 42.35 (40.02, 44.81) |
| Niger | 20 to 24 | 53.61 (51.03, 56.33) |
| Niger | 25 to 29 | 68.47 (65.4, 71.69) |
| Niger | 30 to 34 | 109.09 (104.61, 113.77) |
| Niger | 35 to 39 | 179.48 (172.53, 186.72) |
| Nigeria | 15 to 19 | 58.13 (57.3, 58.98) |
| Nigeria | 20 to 24 | 70.41 (69.51, 71.32) |
| Nigeria | 25 to 29 | 83.55 (82.53, 84.57) |
| Nigeria | 30 to 34 | 108.05 (106.78, 109.33) |
| Nigeria | 35 to 39 | 151.28 (149.53, 153.04) |
| Niue | 15 to 19 | 31.6 (0.18, 5606.25) |
| Niue | 20 to 24 | 35.65 (0.2, 6394.08) |
| Niue | 25 to 29 | 35.08 (0.17, 7072.03) |
| Niue | 30 to 34 | 45.5 (0.26, 7984.02) |
| Niue | 35 to 39 | 66.63 (0.45, 9977.73) |
| North Macedonia | 15 to 19 | 28.29 (23.72, 33.73) |
| North Macedonia | 20 to 24 | 28.64 (24.33, 33.7) |
| North Macedonia | 25 to 29 | 31.16 (26.59, 36.51) |
| North Macedonia | 30 to 34 | 39.24 (33.75, 45.64) |
| North Macedonia | 35 to 39 | 56.48 (48.84, 65.32) |
| Northern Mariana Islands | 15 to 19 | 27.11 (8.69, 84.59) |
| Northern Mariana Islands | 20 to 24 | 34.64 (14.01, 85.68) |
| Northern Mariana Islands | 25 to 29 | 37.11 (17.76, 77.52) |
| Northern Mariana Islands | 30 to 34 | 47 (21.67, 101.9) |
| Northern Mariana Islands | 35 to 39 | 67.42 (29.14, 155.98) |
| Norway | 15 to 19 | 61.64 (56.33, 67.45) |
| Norway | 20 to 24 | 66.28 (61.1, 71.9) |
| Norway | 25 to 29 | 66.93 (61.85, 72.43) |
| Norway | 30 to 34 | 67.5 (62.38, 73.05) |
| Norway | 35 to 39 | 72.41 (66.78, 78.52) |
| Oman | 15 to 19 | 153.15 (143.07, 163.92) |
| Oman | 20 to 24 | 159.74 (150.02, 170.08) |
| Oman | 25 to 29 | 154.66 (145.84, 164.02) |
| Oman | 30 to 34 | 153.09 (144.3, 162.42) |
| Oman | 35 to 39 | 167.26 (157.67, 177.43) |
| Pakistan | 15 to 19 | 64.92 (64.08, 65.76) |
| Pakistan | 20 to 24 | 70.65 (69.82, 71.5) |
| Pakistan | 25 to 29 | 83.23 (82.28, 84.18) |
| Pakistan | 30 to 34 | 115.48 (114.24, 116.73) |
| Pakistan | 35 to 39 | 182.83 (180.97, 184.71) |
| Palau | 15 to 19 | 59.49 (15.71, 225.23) |
| Palau | 20 to 24 | 60.71 (18.1, 203.66) |
| Palau | 25 to 29 | 60.54 (18.4, 199.18) |
| Palau | 30 to 34 | 62.3 (19.13, 202.87) |
| Palau | 35 to 39 | 64.38 (18.37, 225.67) |
| Palestine | 15 to 19 | 83.82 (77.18, 91.03) |
| Palestine | 20 to 24 | 89.11 (82.54, 96.21) |
| Palestine | 25 to 29 | 89.43 (82.85, 96.54) |
| Palestine | 30 to 34 | 91.68 (84.71, 99.23) |
| Palestine | 35 to 39 | 100.84 (92.83, 109.53) |
| Panama | 15 to 19 | 71.97 (66.06, 78.41) |
| Panama | 20 to 24 | 88.41 (81.97, 95.36) |
| Panama | 25 to 29 | 98.58 (91.69, 105.98) |
| Panama | 30 to 34 | 113.36 (105.6, 121.69) |
| Panama | 35 to 39 | 144 (134.2, 154.52) |
| Papua New Guinea | 15 to 19 | 55.1 (51.28, 59.2) |
| Papua New Guinea | 20 to 24 | 57.4 (53.76, 61.29) |
| Papua New Guinea | 25 to 29 | 62.5 (58.68, 66.57) |
| Papua New Guinea | 30 to 34 | 77.76 (73.18, 82.63) |
| Papua New Guinea | 35 to 39 | 111.87 (105.52, 118.61) |
| Paraguay | 15 to 19 | 107.98 (102.27, 114.01) |
| Paraguay | 20 to 24 | 121.56 (115.6, 127.84) |
| Paraguay | 25 to 29 | 129.21 (122.97, 135.77) |
| Paraguay | 30 to 34 | 141.63 (134.75, 148.85) |
| Paraguay | 35 to 39 | 168.34 (160.01, 177.11) |
| Peru | 15 to 19 | 94.68 (92.34, 97.08) |
| Peru | 20 to 24 | 107.63 (105.18, 110.14) |
| Peru | 25 to 29 | 115.89 (113.3, 118.54) |
| Peru | 30 to 34 | 129.47 (126.58, 132.42) |
| Peru | 35 to 39 | 159.53 (155.96, 163.18) |
| Philippines | 15 to 19 | 56.72 (55.65, 57.81) |
| Philippines | 20 to 24 | 54.29 (53.31, 55.29) |
| Philippines | 25 to 29 | 55.94 (54.93, 56.96) |
| Philippines | 30 to 34 | 67.78 (66.59, 68.99) |
| Philippines | 35 to 39 | 98.38 (96.72, 100.07) |
| Poland | 15 to 19 | 28.64 (27.48, 29.84) |
| Poland | 20 to 24 | 28.67 (27.57, 29.82) |
| Poland | 25 to 29 | 31.11 (29.96, 32.31) |
| Poland | 30 to 34 | 38.21 (36.86, 39.61) |
| Poland | 35 to 39 | 53.65 (51.82, 55.55) |
| Portugal | 15 to 19 | 57.6 (54.4, 61) |
| Portugal | 20 to 24 | 62.59 (59.38, 65.97) |
| Portugal | 25 to 29 | 63.13 (59.95, 66.48) |
| Portugal | 30 to 34 | 63.11 (59.9, 66.5) |
| Portugal | 35 to 39 | 67.38 (63.85, 71.1) |
| Puerto Rico | 15 to 19 | 49.19 (44.63, 54.21) |
| Puerto Rico | 20 to 24 | 55.35 (50.55, 60.6) |
| Puerto Rico | 25 to 29 | 60.36 (55.22, 65.99) |
| Puerto Rico | 30 to 34 | 69 (63.17, 75.38) |
| Puerto Rico | 35 to 39 | 85.96 (78.62, 93.97) |
| Qatar | 15 to 19 | 89.43 (73.8, 108.36) |
| Qatar | 20 to 24 | 88.17 (75.88, 102.45) |
| Qatar | 25 to 29 | 86.45 (76.37, 97.85) |
| Qatar | 30 to 34 | 87.05 (78.18, 96.93) |
| Qatar | 35 to 39 | 94.69 (85.79, 104.51) |
| Republic of Korea | 15 to 19 | 59.34 (57.83, 60.88) |
| Republic of Korea | 20 to 24 | 61.28 (59.84, 62.76) |
| Republic of Korea | 25 to 29 | 60.35 (58.94, 61.79) |
| Republic of Korea | 30 to 34 | 59.48 (58.07, 60.92) |
| Republic of Korea | 35 to 39 | 62.9 (61.37, 64.47) |
| Republic of Moldova | 15 to 19 | 52.74 (48.13, 57.79) |
| Republic of Moldova | 20 to 24 | 52.19 (47.79, 57) |
| Republic of Moldova | 25 to 29 | 56 (51.43, 60.97) |
| Republic of Moldova | 30 to 34 | 70.17 (64.71, 76.09) |
| Republic of Moldova | 35 to 39 | 101.99 (94.24, 110.37) |
| Romania | 15 to 19 | 27.85 (26.38, 29.4) |
| Romania | 20 to 24 | 28.29 (26.89, 29.76) |
| Romania | 25 to 29 | 31.15 (29.65, 32.74) |
| Romania | 30 to 34 | 39.75 (37.92, 41.67) |
| Romania | 35 to 39 | 57.71 (55.13, 60.41) |
| Russian Federation | 15 to 19 | 47.72 (46.93, 48.52) |
| Russian Federation | 20 to 24 | 47.4 (46.66, 48.14) |
| Russian Federation | 25 to 29 | 51.08 (50.32, 51.85) |
| Russian Federation | 30 to 34 | 64.3 (63.39, 65.21) |
| Russian Federation | 35 to 39 | 93.62 (92.34, 94.91) |
| Rwanda | 15 to 19 | 25.71 (23.61, 28) |
| Rwanda | 20 to 24 | 29.63 (27.43, 32.01) |
| Rwanda | 25 to 29 | 37.02 (34.43, 39.8) |
| Rwanda | 30 to 34 | 55.06 (51.46, 58.9) |
| Rwanda | 35 to 39 | 86.34 (80.95, 92.09) |
| Saint Kitts and Nevis | 15 to 19 | 46.32 (19.78, 108.49) |
| Saint Kitts and Nevis | 20 to 24 | 48.92 (22.24, 107.57) |
| Saint Kitts and Nevis | 25 to 29 | 58.14 (27.18, 124.36) |
| Saint Kitts and Nevis | 30 to 34 | 73.74 (36.43, 149.26) |
| Saint Kitts and Nevis | 35 to 39 | 92.72 (46.43, 185.18) |
| Saint Lucia | 15 to 19 | 51.61 (33.42, 79.7) |
| Saint Lucia | 20 to 24 | 58.6 (39.05, 87.92) |
| Saint Lucia | 25 to 29 | 62.25 (41.78, 92.77) |
| Saint Lucia | 30 to 34 | 73.54 (49.67, 108.89) |
| Saint Lucia | 35 to 39 | 94.5 (64.24, 139.02) |
| Saint Vincent and the Grenadines | 15 to 19 | 49.49 (29.62, 82.71) |
| Saint Vincent and the Grenadines | 20 to 24 | 57.67 (35.71, 93.16) |
| Saint Vincent and the Grenadines | 25 to 29 | 65.55 (40.93, 104.96) |
| Saint Vincent and the Grenadines | 30 to 34 | 76.45 (47.73, 122.44) |
| Saint Vincent and the Grenadines | 35 to 39 | 99.08 (62.05, 158.23) |
| Samoa | 15 to 19 | 34.43 (21.09, 56.2) |
| Samoa | 20 to 24 | 35.52 (22.09, 57.12) |
| Samoa | 25 to 29 | 40.91 (25.29, 66.18) |
| Samoa | 30 to 34 | 52.25 (32.8, 83.23) |
| Samoa | 35 to 39 | 80.07 (51.13, 125.39) |
| San Marino | 15 to 19 | 50.92 (14.72, 176.16) |
| San Marino | 20 to 24 | 46.66 (14.57, 149.4) |
| San Marino | 25 to 29 | 44.68 (14.13, 141.3) |
| San Marino | 30 to 34 | 43.1 (13.36, 139.01) |
| San Marino | 35 to 39 | 49.76 (14.77, 167.64) |
| Sao Tome and Principe | 15 to 19 | 37.56 (22.56, 62.53) |
| Sao Tome and Principe | 20 to 24 | 47.07 (29.73, 74.52) |
| Sao Tome and Principe | 25 to 29 | 57.98 (37.53, 89.57) |
| Sao Tome and Principe | 30 to 34 | 77.73 (50.99, 118.47) |
| Sao Tome and Principe | 35 to 39 | 116.13 (77.45, 174.12) |
| Saudi Arabia | 15 to 19 | 164.07 (160.48, 167.75) |
| Saudi Arabia | 20 to 24 | 160.19 (156.94, 163.51) |
| Saudi Arabia | 25 to 29 | 152.02 (148.99, 155.11) |
| Saudi Arabia | 30 to 34 | 147.46 (144.57, 150.4) |
| Saudi Arabia | 35 to 39 | 152.54 (149.54, 155.6) |
| Senegal | 15 to 19 | 44.44 (41.9, 47.13) |
| Senegal | 20 to 24 | 53.01 (50.29, 55.87) |
| Senegal | 25 to 29 | 63.98 (60.87, 67.25) |
| Senegal | 30 to 34 | 87.31 (83.24, 91.58) |
| Senegal | 35 to 39 | 125.91 (120.15, 131.94) |
| Serbia | 15 to 19 | 27.61 (25.32, 30.11) |
| Serbia | 20 to 24 | 27.99 (25.81, 30.36) |
| Serbia | 25 to 29 | 30.79 (28.47, 33.3) |
| Serbia | 30 to 34 | 39.18 (36.34, 42.24) |
| Serbia | 35 to 39 | 56.6 (52.62, 60.88) |
| Seychelles | 15 to 19 | 56.09 (30.45, 103.35) |
| Seychelles | 20 to 24 | 53.24 (30.13, 94.09) |
| Seychelles | 25 to 29 | 52.8 (30.25, 92.16) |
| Seychelles | 30 to 34 | 63.35 (37.16, 107.99) |
| Seychelles | 35 to 39 | 88.3 (52.63, 148.15) |
| Sierra Leone | 15 to 19 | 41.52 (37.9, 45.49) |
| Sierra Leone | 20 to 24 | 50.28 (46.36, 54.52) |
| Sierra Leone | 25 to 29 | 61 (56.6, 65.74) |
| Sierra Leone | 30 to 34 | 83.17 (77.53, 89.21) |
| Sierra Leone | 35 to 39 | 119.42 (111.52, 127.87) |
| Singapore | 15 to 19 | 68.57 (63.04, 74.58) |
| Singapore | 20 to 24 | 70.35 (65.45, 75.63) |
| Singapore | 25 to 29 | 69.15 (64.62, 74) |
| Singapore | 30 to 34 | 68.53 (63.98, 73.41) |
| Singapore | 35 to 39 | 72.76 (67.82, 78.06) |
| Slovakia | 15 to 19 | 27.36 (24.52, 30.53) |
| Slovakia | 20 to 24 | 27.7 (25, 30.7) |
| Slovakia | 25 to 29 | 30.36 (27.53, 33.48) |
| Slovakia | 30 to 34 | 38.64 (35.22, 42.4) |
| Slovakia | 35 to 39 | 55.75 (51, 60.93) |
| Slovenia | 15 to 19 | 26.6 (21.85, 32.37) |
| Slovenia | 20 to 24 | 26.89 (22.43, 32.24) |
| Slovenia | 25 to 29 | 29.07 (24.47, 34.52) |
| Slovenia | 30 to 34 | 36.6 (31.11, 43.06) |
| Slovenia | 35 to 39 | 52.79 (45.19, 61.67) |
| Solomon Islands | 15 to 19 | 40.21 (30.16, 53.6) |
| Solomon Islands | 20 to 24 | 43.21 (33.09, 56.43) |
| Solomon Islands | 25 to 29 | 48.33 (37.31, 62.61) |
| Solomon Islands | 30 to 34 | 62.07 (48.34, 79.69) |
| Solomon Islands | 35 to 39 | 88.98 (69.84, 113.38) |
| Somalia | 15 to 19 | 34.79 (32.69, 37.01) |
| Somalia | 20 to 24 | 42.04 (39.8, 44.4) |
| Somalia | 25 to 29 | 54.65 (51.97, 57.47) |
| Somalia | 30 to 34 | 81.47 (77.78, 85.34) |
| Somalia | 35 to 39 | 124.03 (118.69, 129.6) |
| South Africa | 15 to 19 | 39.28 (38.2, 40.39) |
| South Africa | 20 to 24 | 47.71 (46.56, 48.88) |
| South Africa | 25 to 29 | 60.93 (59.6, 62.3) |
| South Africa | 30 to 34 | 101.08 (99.09, 103.12) |
| South Africa | 35 to 39 | 169.78 (166.59, 173.02) |
| South Sudan | 15 to 19 | 61.13 (57.57, 64.92) |
| South Sudan | 20 to 24 | 56.24 (53.05, 59.62) |
| South Sudan | 25 to 29 | 59.93 (56.51, 63.56) |
| South Sudan | 30 to 34 | 79.11 (74.73, 83.76) |
| South Sudan | 35 to 39 | 115 (108.73, 121.62) |
| Spain | 15 to 19 | 107.44 (105.22, 109.7) |
| Spain | 20 to 24 | 120.45 (118.23, 122.72) |
| Spain | 25 to 29 | 124.09 (121.91, 126.31) |
| Spain | 30 to 34 | 126.12 (123.93, 128.34) |
| Spain | 35 to 39 | 136.91 (134.49, 139.37) |
| Sri Lanka | 15 to 19 | 63.29 (60.88, 65.79) |
| Sri Lanka | 20 to 24 | 54.6 (52.56, 56.71) |
| Sri Lanka | 25 to 29 | 52.37 (50.4, 54.42) |
| Sri Lanka | 30 to 34 | 61.78 (59.51, 64.14) |
| Sri Lanka | 35 to 39 | 92.37 (89.1, 95.76) |
| Sudan | 15 to 19 | 98.8 (96.32, 101.34) |
| Sudan | 20 to 24 | 102.83 (100.39, 105.34) |
| Sudan | 25 to 29 | 101.8 (99.34, 104.31) |
| Sudan | 30 to 34 | 104.73 (102.13, 107.39) |
| Sudan | 35 to 39 | 116.86 (113.84, 119.96) |
| Suriname | 15 to 19 | 60.16 (47.25, 76.6) |
| Suriname | 20 to 24 | 70.06 (56.43, 86.99) |
| Suriname | 25 to 29 | 78.27 (63.74, 96.13) |
| Suriname | 30 to 34 | 93.59 (76.68, 114.23) |
| Suriname | 35 to 39 | 121.13 (99.27, 147.8) |
| Sweden | 15 to 19 | 37.54 (34.5, 40.85) |
| Sweden | 20 to 24 | 41.23 (38.22, 44.47) |
| Sweden | 25 to 29 | 43.11 (40.1, 46.35) |
| Sweden | 30 to 34 | 45.07 (41.94, 48.44) |
| Sweden | 35 to 39 | 49.61 (46.07, 53.42) |
| Switzerland | 15 to 19 | 53.54 (49.43, 58) |
| Switzerland | 20 to 24 | 58.39 (54.39, 62.68) |
| Switzerland | 25 to 29 | 58.94 (55.1, 63.05) |
| Switzerland | 30 to 34 | 58.97 (55.16, 63.03) |
| Switzerland | 35 to 39 | 63.19 (59.02, 67.65) |
| Syrian Arab Republic | 15 to 19 | 91.24 (88.3, 94.28) |
| Syrian Arab Republic | 20 to 24 | 95.76 (92.8, 98.81) |
| Syrian Arab Republic | 25 to 29 | 96.46 (93.4, 99.63) |
| Syrian Arab Republic | 30 to 34 | 99.6 (96.36, 102.94) |
| Syrian Arab Republic | 35 to 39 | 111.17 (107.36, 115.12) |
| Taiwan (Province of China) | 15 to 19 | 17.6 (16.43, 18.86) |
| Taiwan (Province of China) | 20 to 24 | 15.22 (14.24, 16.26) |
| Taiwan (Province of China) | 25 to 29 | 15.22 (14.26, 16.24) |
| Taiwan (Province of China) | 30 to 34 | 21.2 (19.96, 22.51) |
| Taiwan (Province of China) | 35 to 39 | 33.93 (32.08, 35.88) |
| Tajikistan | 15 to 19 | 45.18 (42, 48.61) |
| Tajikistan | 20 to 24 | 47.22 (44.1, 50.56) |
| Tajikistan | 25 to 29 | 52.49 (49.11, 56.11) |
| Tajikistan | 30 to 34 | 67.1 (62.88, 71.59) |
| Tajikistan | 35 to 39 | 98.5 (92.48, 104.92) |
| Thailand | 15 to 19 | 64.37 (63.02, 65.74) |
| Thailand | 20 to 24 | 56.06 (54.92, 57.21) |
| Thailand | 25 to 29 | 53.93 (52.84, 55.04) |
| Thailand | 30 to 34 | 63.66 (62.43, 64.91) |
| Thailand | 35 to 39 | 95.4 (93.65, 97.18) |
| Timor-Leste | 15 to 19 | 87.66 (75.34, 102.01) |
| Timor-Leste | 20 to 24 | 83.47 (72.34, 96.31) |
| Timor-Leste | 25 to 29 | 86.73 (75.21, 100.01) |
| Timor-Leste | 30 to 34 | 104.88 (90.9, 121) |
| Timor-Leste | 35 to 39 | 150.63 (130.75, 173.55) |
| Togo | 15 to 19 | 40.33 (37.01, 43.95) |
| Togo | 20 to 24 | 48.99 (45.39, 52.86) |
| Togo | 25 to 29 | 60.76 (56.6, 65.21) |
| Togo | 30 to 34 | 85.21 (79.74, 91.05) |
| Togo | 35 to 39 | 126.41 (118.57, 134.76) |
| Tokelau | 15 to 19 | 36.01 (0.12, 10749.13) |
| Tokelau | 20 to 24 | 37.33 (0.14, 9903.42) |
| Tokelau | 25 to 29 | 40.78 (0.14, 11526.67) |
| Tokelau | 30 to 34 | 51.62 (0.19, 14264.22) |
| Tokelau | 35 to 39 | 74.78 (0.29, 19027.77) |
| Tonga | 15 to 19 | 26.82 (12.68, 56.76) |
| Tonga | 20 to 24 | 23.15 (10.77, 49.75) |
| Tonga | 25 to 29 | 25.83 (11.94, 55.86) |
| Tonga | 30 to 34 | 37.71 (18.25, 77.89) |
| Tonga | 35 to 39 | 51.99 (24.78, 109.08) |
| Trinidad and Tobago | 15 to 19 | 50.27 (43.04, 58.72) |
| Trinidad and Tobago | 20 to 24 | 58.18 (50.43, 67.13) |
| Trinidad and Tobago | 25 to 29 | 65.33 (56.94, 74.94) |
| Trinidad and Tobago | 30 to 34 | 79.9 (69.99, 91.21) |
| Trinidad and Tobago | 35 to 39 | 106.26 (93.19, 121.17) |
| Tunisia | 15 to 19 | 65.58 (62.43, 68.9) |
| Tunisia | 20 to 24 | 70.92 (67.73, 74.26) |
| Tunisia | 25 to 29 | 73.06 (69.79, 76.47) |
| Tunisia | 30 to 34 | 77.75 (74.26, 81.41) |
| Tunisia | 35 to 39 | 89.52 (85.42, 93.81) |
| Turkey | 15 to 19 | 57.49 (56.34, 58.66) |
| Turkey | 20 to 24 | 60.28 (59.15, 61.42) |
| Turkey | 25 to 29 | 60.97 (59.84, 62.11) |
| Turkey | 30 to 34 | 63.2 (62.02, 64.4) |
| Turkey | 35 to 39 | 70.95 (69.58, 72.34) |
| Turkmenistan | 15 to 19 | 54.31 (49.98, 59.01) |
| Turkmenistan | 20 to 24 | 54.45 (50.32, 58.91) |
| Turkmenistan | 25 to 29 | 58.44 (54.1, 63.12) |
| Turkmenistan | 30 to 34 | 71.72 (66.53, 77.32) |
| Turkmenistan | 35 to 39 | 102.09 (94.84, 109.9) |
| Tuvalu | 15 to 19 | 39.13 (4.46, 343.52) |
| Tuvalu | 20 to 24 | 39.84 (5.28, 300.51) |
| Tuvalu | 25 to 29 | 43.84 (6.22, 309.29) |
| Tuvalu | 30 to 34 | 55.78 (8.74, 356.07) |
| Tuvalu | 35 to 39 | 150.45 (31.23, 724.94) |
| Uganda | 15 to 19 | 26.28 (25.04, 27.59) |
| Uganda | 20 to 24 | 31.21 (29.88, 32.6) |
| Uganda | 25 to 29 | 39.52 (37.93, 41.19) |
| Uganda | 30 to 34 | 58.69 (56.44, 61.04) |
| Uganda | 35 to 39 | 91.8 (88.4, 95.34) |
| Ukraine | 15 to 19 | 40.76 (39.49, 42.07) |
| Ukraine | 20 to 24 | 40.75 (39.55, 41.99) |
| Ukraine | 25 to 29 | 44.04 (42.79, 45.32) |
| Ukraine | 30 to 34 | 54.57 (53.1, 56.08) |
| Ukraine | 35 to 39 | 77.76 (75.71, 79.85) |
| United Arab Emirates | 15 to 19 | 83.79 (76.95, 91.24) |
| United Arab Emirates | 20 to 24 | 86.48 (81.07, 92.26) |
| United Arab Emirates | 25 to 29 | 85.93 (81.53, 90.57) |
| United Arab Emirates | 30 to 34 | 86.98 (83.15, 90.99) |
| United Arab Emirates | 35 to 39 | 95.73 (91.72, 99.93) |
| United Kingdom | 15 to 19 | 63.08 (61.53, 64.68) |
| United Kingdom | 20 to 24 | 67.94 (66.41, 69.5) |
| United Kingdom | 25 to 29 | 68.76 (67.27, 70.28) |
| United Kingdom | 30 to 34 | 69.48 (67.97, 71.02) |
| United Kingdom | 35 to 39 | 75.03 (73.36, 76.75) |
| United Republic of Tanzania | 15 to 19 | 42.79 (41.48, 44.15) |
| United Republic of Tanzania | 20 to 24 | 52.51 (51.09, 53.97) |
| United Republic of Tanzania | 25 to 29 | 68.27 (66.57, 70.03) |
| United Republic of Tanzania | 30 to 34 | 110.13 (107.62, 112.71) |
| United Republic of Tanzania | 35 to 39 | 182.07 (178.15, 186.07) |
| United States of America | 15 to 19 | 44.85 (43.82, 45.9) |
| United States of America | 20 to 24 | 48.52 (47.51, 49.56) |
| United States of America | 25 to 29 | 47.56 (46.58, 48.55) |
| United States of America | 30 to 34 | 45.42 (44.46, 46.39) |
| United States of America | 35 to 39 | 46.51 (45.48, 47.55) |
| United States Virgin Islands | 15 to 19 | 50.73 (28.23, 91.16) |
| United States Virgin Islands | 20 to 24 | 61.05 (34.48, 108.12) |
| United States Virgin Islands | 25 to 29 | 65.63 (37.56, 114.7) |
| United States Virgin Islands | 30 to 34 | 74.04 (43.47, 126.12) |
| United States Virgin Islands | 35 to 39 | 95.54 (56.21, 162.4) |
| Uruguay | 15 to 19 | 62.76 (56.91, 69.21) |
| Uruguay | 20 to 24 | 63.96 (58.28, 70.19) |
| Uruguay | 25 to 29 | 62.18 (56.6, 68.32) |
| Uruguay | 30 to 34 | 61.04 (55.42, 67.23) |
| Uruguay | 35 to 39 | 65.93 (59.63, 72.91) |
| Uzbekistan | 15 to 19 | 45.27 (43.61, 46.98) |
| Uzbekistan | 20 to 24 | 46.59 (45, 48.24) |
| Uzbekistan | 25 to 29 | 51.15 (49.45, 52.9) |
| Uzbekistan | 30 to 34 | 64.39 (62.33, 66.52) |
| Uzbekistan | 35 to 39 | 92.86 (89.97, 95.85) |
| Vanuatu | 15 to 19 | 29.36 (17.33, 49.76) |
| Vanuatu | 20 to 24 | 30.7 (18.81, 50.11) |
| Vanuatu | 25 to 29 | 32.96 (20.39, 53.29) |
| Vanuatu | 30 to 34 | 44.26 (28.03, 69.89) |
| Vanuatu | 35 to 39 | 64.95 (41.82, 100.87) |
| Venezuela (Bolivarian Republic of) | 15 to 19 | 62.26 (60.3, 64.3) |
| Venezuela (Bolivarian Republic of) | 20 to 24 | 76.52 (74.35, 78.75) |
| Venezuela (Bolivarian Republic of) | 25 to 29 | 85.1 (82.78, 87.48) |
| Venezuela (Bolivarian Republic of) | 30 to 34 | 97.18 (94.58, 99.85) |
| Venezuela (Bolivarian Republic of) | 35 to 39 | 122.83 (119.56, 126.2) |
| Viet Nam | 15 to 19 | 65.79 (63.74, 67.9) |
| Viet Nam | 20 to 24 | 60.61 (58.8, 62.47) |
| Viet Nam | 25 to 29 | 60.73 (58.96, 62.56) |
| Viet Nam | 30 to 34 | 71.84 (69.79, 73.96) |
| Viet Nam | 35 to 39 | 100.18 (97.39, 103.06) |
| Yemen | 15 to 19 | 75.33 (72.74, 78.01) |
| Yemen | 20 to 24 | 81.19 (78.56, 83.91) |
| Yemen | 25 to 29 | 82.56 (79.92, 85.29) |
| Yemen | 30 to 34 | 87.18 (84.35, 90.09) |
| Yemen | 35 to 39 | 101.22 (97.84, 104.72) |
| Zambia | 15 to 19 | 27.65 (25.78, 29.66) |
| Zambia | 20 to 24 | 33.19 (31.19, 35.32) |
| Zambia | 25 to 29 | 43.03 (40.6, 45.61) |
| Zambia | 30 to 34 | 65.01 (61.58, 68.64) |
| Zambia | 35 to 39 | 103.48 (98.29, 108.94) |
| Zimbabwe | 15 to 19 | 41.11 (39.1, 43.23) |
| Zimbabwe | 20 to 24 | 50.18 (47.95, 52.52) |
| Zimbabwe | 25 to 29 | 66.09 (63.29, 69.01) |
| Zimbabwe | 30 to 34 | 108.35 (104.09, 112.78) |
| Zimbabwe | 35 to 39 | 181.03 (174.23, 188.09) |

**Abbreviations:** DALYs, Disability adjusted life years.

**Table S8 Period effects on blindness and vision loss DALYs in teenagers and young adults across countries**

| **Location** | **Period** | **DALYs rate ratio** |
| --- | --- | --- |
| Afghanistan | 1990 to 1994 | 1 (1, 1) |
| Afghanistan | 1995 to 1999 | 1 (0.967, 1.034) |
| Afghanistan | 2000 to 2004 | 0.994 (0.962, 1.027) |
| Afghanistan | 2005 to 2009 | 0.969 (0.939, 1) |
| Afghanistan | 2010 to 2014 | 0.934 (0.906, 0.963) |
| Afghanistan | 2015 to 2019 | 0.902 (0.874, 0.93) |
| Albania | 1990 to 1994 | 1 (1, 1) |
| Albania | 1995 to 1999 | 0.993 (0.87, 1.134) |
| Albania | 2000 to 2004 | 0.982 (0.851, 1.133) |
| Albania | 2005 to 2009 | 0.964 (0.833, 1.115) |
| Albania | 2010 to 2014 | 0.96 (0.833, 1.106) |
| Albania | 2015 to 2019 | 0.947 (0.824, 1.089) |
| Algeria | 1990 to 1994 | 1 (1, 1) |
| Algeria | 1995 to 1999 | 0.971 (0.945, 0.998) |
| Algeria | 2000 to 2004 | 0.939 (0.914, 0.964) |
| Algeria | 2005 to 2009 | 0.909 (0.886, 0.933) |
| Algeria | 2010 to 2014 | 0.88 (0.858, 0.902) |
| Algeria | 2015 to 2019 | 0.849 (0.828, 0.871) |
| American Samoa | 1990 to 1994 | 1 (1, 1) |
| American Samoa | 1995 to 1999 | 0.943 (0.376, 2.365) |
| American Samoa | 2000 to 2004 | 0.985 (0.385, 2.524) |
| American Samoa | 2005 to 2009 | 0.863 (0.319, 2.333) |
| American Samoa | 2010 to 2014 | 0.875 (0.333, 2.3) |
| American Samoa | 2015 to 2019 | 0.799 (0.302, 2.116) |
| Andorra | 1990 to 1994 | 1 (1, 1) |
| Andorra | 1995 to 1999 | 1.036 (0.502, 2.137) |
| Andorra | 2000 to 2004 | 0.94 (0.433, 2.04) |
| Andorra | 2005 to 2009 | 1.002 (0.476, 2.111) |
| Andorra | 2010 to 2014 | 0.989 (0.464, 2.108) |
| Andorra | 2015 to 2019 | 0.946 (0.455, 1.966) |
| Angola | 1990 to 1994 | 1 (1, 1) |
| Angola | 1995 to 1999 | 0.984 (0.929, 1.042) |
| Angola | 2000 to 2004 | 0.955 (0.902, 1.011) |
| Angola | 2005 to 2009 | 0.92 (0.87, 0.971) |
| Angola | 2010 to 2014 | 0.864 (0.821, 0.911) |
| Angola | 2015 to 2019 | 0.79 (0.752, 0.83) |
| Antigua and Barbuda | 1990 to 1994 | 1 (1, 1) |
| Antigua and Barbuda | 1995 to 1999 | 0.982 (0.499, 1.932) |
| Antigua and Barbuda | 2000 to 2004 | 0.971 (0.49, 1.923) |
| Antigua and Barbuda | 2005 to 2009 | 0.963 (0.486, 1.91) |
| Antigua and Barbuda | 2010 to 2014 | 0.919 (0.472, 1.788) |
| Antigua and Barbuda | 2015 to 2019 | 0.91 (0.48, 1.724) |
| Argentina | 1990 to 1994 | 1 (1, 1) |
| Argentina | 1995 to 1999 | 0.986 (0.957, 1.015) |
| Argentina | 2000 to 2004 | 0.972 (0.944, 1.001) |
| Argentina | 2005 to 2009 | 0.963 (0.936, 0.991) |
| Argentina | 2010 to 2014 | 0.951 (0.926, 0.978) |
| Argentina | 2015 to 2019 | 0.935 (0.91, 0.961) |
| Armenia | 1990 to 1994 | 1 (1, 1) |
| Armenia | 1995 to 1999 | 0.981 (0.881, 1.091) |
| Armenia | 2000 to 2004 | 0.954 (0.851, 1.069) |
| Armenia | 2005 to 2009 | 0.937 (0.836, 1.051) |
| Armenia | 2010 to 2014 | 0.925 (0.828, 1.033) |
| Armenia | 2015 to 2019 | 0.911 (0.818, 1.015) |
| Australia | 1990 to 1994 | 1 (1, 1) |
| Australia | 1995 to 1999 | 0.989 (0.942, 1.038) |
| Australia | 2000 to 2004 | 0.977 (0.93, 1.027) |
| Australia | 2005 to 2009 | 0.976 (0.929, 1.024) |
| Australia | 2010 to 2014 | 0.977 (0.932, 1.023) |
| Australia | 2015 to 2019 | 0.967 (0.925, 1.011) |
| Austria | 1990 to 1994 | 1 (1, 1) |
| Austria | 1995 to 1999 | 0.992 (0.926, 1.063) |
| Austria | 2000 to 2004 | 0.984 (0.915, 1.057) |
| Austria | 2005 to 2009 | 0.976 (0.907, 1.05) |
| Austria | 2010 to 2014 | 0.968 (0.901, 1.039) |
| Austria | 2015 to 2019 | 0.961 (0.898, 1.028) |
| Azerbaijan | 1990 to 1994 | 1 (1, 1) |
| Azerbaijan | 1995 to 1999 | 0.998 (0.935, 1.066) |
| Azerbaijan | 2000 to 2004 | 0.992 (0.928, 1.061) |
| Azerbaijan | 2005 to 2009 | 0.971 (0.908, 1.037) |
| Azerbaijan | 2010 to 2014 | 0.932 (0.875, 0.993) |
| Azerbaijan | 2015 to 2019 | 0.906 (0.853, 0.963) |
| Bahamas | 1990 to 1994 | 1 (1, 1) |
| Bahamas | 1995 to 1999 | 1.013 (0.729, 1.408) |
| Bahamas | 2000 to 2004 | 0.981 (0.7, 1.374) |
| Bahamas | 2005 to 2009 | 0.978 (0.7, 1.366) |
| Bahamas | 2010 to 2014 | 0.975 (0.706, 1.347) |
| Bahamas | 2015 to 2019 | 0.943 (0.692, 1.285) |
| Bahrain | 1990 to 1994 | 1 (1, 1) |
| Bahrain | 1995 to 1999 | 0.967 (0.809, 1.156) |
| Bahrain | 2000 to 2004 | 0.922 (0.771, 1.102) |
| Bahrain | 2005 to 2009 | 0.884 (0.749, 1.043) |
| Bahrain | 2010 to 2014 | 0.856 (0.732, 1.001) |
| Bahrain | 2015 to 2019 | 0.839 (0.72, 0.978) |
| Bangladesh | 1990 to 1994 | 1 (1, 1) |
| Bangladesh | 1995 to 1999 | 0.966 (0.951, 0.982) |
| Bangladesh | 2000 to 2004 | 0.936 (0.921, 0.951) |
| Bangladesh | 2005 to 2009 | 0.902 (0.888, 0.917) |
| Bangladesh | 2010 to 2014 | 0.86 (0.847, 0.873) |
| Bangladesh | 2015 to 2019 | 0.824 (0.812, 0.837) |
| Barbados | 1990 to 1994 | 1 (1, 1) |
| Barbados | 1995 to 1999 | 0.996 (0.603, 1.646) |
| Barbados | 2000 to 2004 | 0.99 (0.584, 1.68) |
| Barbados | 2005 to 2009 | 1.032 (0.61, 1.745) |
| Barbados | 2010 to 2014 | 0.968 (0.579, 1.616) |
| Barbados | 2015 to 2019 | 0.956 (0.585, 1.561) |
| Belarus | 1990 to 1994 | 1 (1, 1) |
| Belarus | 1995 to 1999 | 0.992 (0.926, 1.062) |
| Belarus | 2000 to 2004 | 0.987 (0.918, 1.061) |
| Belarus | 2005 to 2009 | 0.97 (0.902, 1.044) |
| Belarus | 2010 to 2014 | 0.952 (0.887, 1.023) |
| Belarus | 2015 to 2019 | 0.938 (0.874, 1.006) |
| Belgium | 1990 to 1994 | 1 (1, 1) |
| Belgium | 1995 to 1999 | 0.99 (0.93, 1.054) |
| Belgium | 2000 to 2004 | 0.977 (0.915, 1.043) |
| Belgium | 2005 to 2009 | 0.968 (0.907, 1.034) |
| Belgium | 2010 to 2014 | 0.96 (0.901, 1.023) |
| Belgium | 2015 to 2019 | 0.952 (0.896, 1.012) |
| Belize | 1990 to 1994 | 1 (1, 1) |
| Belize | 1995 to 1999 | 0.997 (0.682, 1.46) |
| Belize | 2000 to 2004 | 0.952 (0.653, 1.387) |
| Belize | 2005 to 2009 | 0.943 (0.657, 1.354) |
| Belize | 2010 to 2014 | 0.911 (0.646, 1.285) |
| Belize | 2015 to 2019 | 0.896 (0.644, 1.246) |
| Benin | 1990 to 1994 | 1 (1, 1) |
| Benin | 1995 to 1999 | 1.121 (1.029, 1.221) |
| Benin | 2000 to 2004 | 1.123 (1.034, 1.221) |
| Benin | 2005 to 2009 | 1.089 (1.005, 1.179) |
| Benin | 2010 to 2014 | 1.09 (1.01, 1.176) |
| Benin | 2015 to 2019 | 1.066 (0.99, 1.147) |
| Bermuda | 1990 to 1994 | 1 (1, 1) |
| Bermuda | 1995 to 1999 | 0.883 (0.43, 1.815) |
| Bermuda | 2000 to 2004 | 0.915 (0.424, 1.971) |
| Bermuda | 2005 to 2009 | 0.896 (0.405, 1.984) |
| Bermuda | 2010 to 2014 | 0.816 (0.366, 1.818) |
| Bermuda | 2015 to 2019 | 0.834 (0.385, 1.805) |
| Bhutan | 1990 to 1994 | 1 (1, 1) |
| Bhutan | 1995 to 1999 | 0.98 (0.754, 1.274) |
| Bhutan | 2000 to 2004 | 0.94 (0.719, 1.229) |
| Bhutan | 2005 to 2009 | 0.897 (0.691, 1.166) |
| Bhutan | 2010 to 2014 | 0.864 (0.674, 1.107) |
| Bhutan | 2015 to 2019 | 0.84 (0.661, 1.067) |
| Bolivia (Plurinational State of) | 1990 to 1994 | 1 (1, 1) |
| Bolivia (Plurinational State of) | 1995 to 1999 | 0.981 (0.929, 1.035) |
| Bolivia (Plurinational State of) | 2000 to 2004 | 0.958 (0.908, 1.011) |
| Bolivia (Plurinational State of) | 2005 to 2009 | 0.934 (0.887, 0.984) |
| Bolivia (Plurinational State of) | 2010 to 2014 | 0.915 (0.871, 0.962) |
| Bolivia (Plurinational State of) | 2015 to 2019 | 0.884 (0.843, 0.928) |
| Bosnia and Herzegovina | 1990 to 1994 | 1 (1, 1) |
| Bosnia and Herzegovina | 1995 to 1999 | 0.996 (0.879, 1.128) |
| Bosnia and Herzegovina | 2000 to 2004 | 0.962 (0.845, 1.096) |
| Bosnia and Herzegovina | 2005 to 2009 | 0.95 (0.833, 1.084) |
| Bosnia and Herzegovina | 2010 to 2014 | 0.947 (0.831, 1.08) |
| Bosnia and Herzegovina | 2015 to 2019 | 0.939 (0.824, 1.071) |
| Botswana | 1990 to 1994 | 1 (1, 1) |
| Botswana | 1995 to 1999 | 0.956 (0.832, 1.1) |
| Botswana | 2000 to 2004 | 0.925 (0.803, 1.065) |
| Botswana | 2005 to 2009 | 0.894 (0.779, 1.025) |
| Botswana | 2010 to 2014 | 0.847 (0.743, 0.966) |
| Botswana | 2015 to 2019 | 0.804 (0.709, 0.913) |
| Brazil | 1990 to 1994 | 1 (1, 1) |
| Brazil | 1995 to 1999 | 1.009 (1, 1.019) |
| Brazil | 2000 to 2004 | 1.019 (1.009, 1.029) |
| Brazil | 2005 to 2009 | 0.987 (0.978, 0.997) |
| Brazil | 2010 to 2014 | 0.972 (0.963, 0.981) |
| Brazil | 2015 to 2019 | 0.944 (0.936, 0.953) |
| Brunei Darussalam | 1990 to 1994 | 1 (1, 1) |
| Brunei Darussalam | 1995 to 1999 | 0.993 (0.721, 1.367) |
| Brunei Darussalam | 2000 to 2004 | 0.968 (0.703, 1.333) |
| Brunei Darussalam | 2005 to 2009 | 0.966 (0.708, 1.319) |
| Brunei Darussalam | 2010 to 2014 | 0.955 (0.708, 1.289) |
| Brunei Darussalam | 2015 to 2019 | 0.964 (0.723, 1.287) |
| Bulgaria | 1990 to 1994 | 1 (1, 1) |
| Bulgaria | 1995 to 1999 | 0.997 (0.906, 1.097) |
| Bulgaria | 2000 to 2004 | 0.989 (0.895, 1.093) |
| Bulgaria | 2005 to 2009 | 0.979 (0.885, 1.082) |
| Bulgaria | 2010 to 2014 | 0.969 (0.877, 1.072) |
| Bulgaria | 2015 to 2019 | 0.959 (0.867, 1.06) |
| Burkina Faso | 1990 to 1994 | 1 (1, 1) |
| Burkina Faso | 1995 to 1999 | 1.037 (0.973, 1.104) |
| Burkina Faso | 2000 to 2004 | 1.022 (0.961, 1.087) |
| Burkina Faso | 2005 to 2009 | 1.008 (0.95, 1.069) |
| Burkina Faso | 2010 to 2014 | 1.014 (0.959, 1.072) |
| Burkina Faso | 2015 to 2019 | 0.995 (0.943, 1.05) |
| Burundi | 1990 to 1994 | 1 (1, 1) |
| Burundi | 1995 to 1999 | 0.982 (0.881, 1.096) |
| Burundi | 2000 to 2004 | 0.952 (0.852, 1.063) |
| Burundi | 2005 to 2009 | 0.918 (0.825, 1.02) |
| Burundi | 2010 to 2014 | 0.889 (0.805, 0.981) |
| Burundi | 2015 to 2019 | 0.852 (0.776, 0.935) |
| Cabo Verde | 1990 to 1994 | 1 (1, 1) |
| Cabo Verde | 1995 to 1999 | 0.982 (0.696, 1.385) |
| Cabo Verde | 2000 to 2004 | 0.928 (0.655, 1.315) |
| Cabo Verde | 2005 to 2009 | 0.894 (0.636, 1.258) |
| Cabo Verde | 2010 to 2014 | 0.867 (0.626, 1.199) |
| Cabo Verde | 2015 to 2019 | 0.818 (0.599, 1.117) |
| Cambodia | 1990 to 1994 | 1 (1, 1) |
| Cambodia | 1995 to 1999 | 0.959 (0.921, 0.999) |
| Cambodia | 2000 to 2004 | 0.918 (0.881, 0.956) |
| Cambodia | 2005 to 2009 | 0.821 (0.788, 0.854) |
| Cambodia | 2010 to 2014 | 0.699 (0.672, 0.727) |
| Cambodia | 2015 to 2019 | 0.656 (0.631, 0.682) |
| Cameroon | 1990 to 1994 | 1 (1, 1) |
| Cameroon | 1995 to 1999 | 0.988 (0.93, 1.05) |
| Cameroon | 2000 to 2004 | 0.956 (0.901, 1.014) |
| Cameroon | 2005 to 2009 | 0.931 (0.881, 0.985) |
| Cameroon | 2010 to 2014 | 0.942 (0.893, 0.993) |
| Cameroon | 2015 to 2019 | 0.919 (0.873, 0.967) |
| Canada | 1990 to 1994 | 1 (1, 1) |
| Canada | 1995 to 1999 | 0.998 (0.956, 1.041) |
| Canada | 2000 to 2004 | 0.993 (0.95, 1.037) |
| Canada | 2005 to 2009 | 0.986 (0.944, 1.029) |
| Canada | 2010 to 2014 | 0.98 (0.94, 1.022) |
| Canada | 2015 to 2019 | 0.979 (0.941, 1.019) |
| Central African Republic | 1990 to 1994 | 1 (1, 1) |
| Central African Republic | 1995 to 1999 | 1.11 (0.962, 1.281) |
| Central African Republic | 2000 to 2004 | 1.162 (1.011, 1.335) |
| Central African Republic | 2005 to 2009 | 1.237 (1.085, 1.411) |
| Central African Republic | 2010 to 2014 | 1.382 (1.22, 1.566) |
| Central African Republic | 2015 to 2019 | 1.451 (1.282, 1.641) |
| Chad | 1990 to 1994 | 1 (1, 1) |
| Chad | 1995 to 1999 | 1.03 (0.956, 1.109) |
| Chad | 2000 to 2004 | 1.026 (0.954, 1.103) |
| Chad | 2005 to 2009 | 1.009 (0.942, 1.082) |
| Chad | 2010 to 2014 | 1.013 (0.948, 1.081) |
| Chad | 2015 to 2019 | 0.987 (0.926, 1.051) |
| Chile | 1990 to 1994 | 1 (1, 1) |
| Chile | 1995 to 1999 | 0.981 (0.939, 1.025) |
| Chile | 2000 to 2004 | 0.959 (0.917, 1.003) |
| Chile | 2005 to 2009 | 0.941 (0.9, 0.984) |
| Chile | 2010 to 2014 | 0.926 (0.887, 0.966) |
| Chile | 2015 to 2019 | 0.908 (0.87, 0.946) |
| China | 1990 to 1994 | 1 (1, 1) |
| China | 1995 to 1999 | 0.966 (0.937, 0.995) |
| China | 2000 to 2004 | 0.891 (0.864, 0.918) |
| China | 2005 to 2009 | 0.871 (0.844, 0.898) |
| China | 2010 to 2014 | 0.839 (0.814, 0.865) |
| China | 2015 to 2019 | 0.88 (0.854, 0.906) |
| Colombia | 1990 to 1994 | 1 (1, 1) |
| Colombia | 1995 to 1999 | 0.974 (0.951, 0.998) |
| Colombia | 2000 to 2004 | 0.947 (0.924, 0.97) |
| Colombia | 2005 to 2009 | 0.924 (0.902, 0.946) |
| Colombia | 2010 to 2014 | 0.896 (0.875, 0.917) |
| Colombia | 2015 to 2019 | 0.876 (0.856, 0.895) |
| Comoros | 1990 to 1994 | 1 (1, 1) |
| Comoros | 1995 to 1999 | 0.971 (0.728, 1.294) |
| Comoros | 2000 to 2004 | 0.934 (0.698, 1.25) |
| Comoros | 2005 to 2009 | 0.895 (0.672, 1.192) |
| Comoros | 2010 to 2014 | 0.841 (0.638, 1.109) |
| Comoros | 2015 to 2019 | 0.778 (0.596, 1.016) |
| Congo | 1990 to 1994 | 1 (1, 1) |
| Congo | 1995 to 1999 | 0.989 (0.873, 1.121) |
| Congo | 2000 to 2004 | 0.951 (0.84, 1.077) |
| Congo | 2005 to 2009 | 0.917 (0.813, 1.034) |
| Congo | 2010 to 2014 | 0.875 (0.78, 0.982) |
| Congo | 2015 to 2019 | 0.822 (0.735, 0.918) |
| Cook Islands | 1990 to 1994 | 1 (1, 1) |
| Cook Islands | 1995 to 1999 | 0.984 (0.263, 3.688) |
| Cook Islands | 2000 to 2004 | 0.992 (0.256, 3.841) |
| Cook Islands | 2005 to 2009 | 0.982 (0.254, 3.793) |
| Cook Islands | 2010 to 2014 | 1.11 (0.297, 4.146) |
| Cook Islands | 2015 to 2019 | 0.873 (0.209, 3.645) |
| Costa Rica | 1990 to 1994 | 1 (1, 1) |
| Costa Rica | 1995 to 1999 | 0.995 (0.916, 1.08) |
| Costa Rica | 2000 to 2004 | 0.976 (0.899, 1.061) |
| Costa Rica | 2005 to 2009 | 0.965 (0.889, 1.047) |
| Costa Rica | 2010 to 2014 | 0.953 (0.881, 1.031) |
| Costa Rica | 2015 to 2019 | 0.947 (0.879, 1.021) |
| Croatia | 1990 to 1994 | 1 (1, 1) |
| Croatia | 1995 to 1999 | 0.995 (0.878, 1.127) |
| Croatia | 2000 to 2004 | 0.971 (0.85, 1.109) |
| Croatia | 2005 to 2009 | 0.96 (0.839, 1.098) |
| Croatia | 2010 to 2014 | 0.961 (0.844, 1.096) |
| Croatia | 2015 to 2019 | 0.958 (0.844, 1.089) |
| Cuba | 1990 to 1994 | 1 (1, 1) |
| Cuba | 1995 to 1999 | 1.001 (0.958, 1.046) |
| Cuba | 2000 to 2004 | 0.99 (0.946, 1.036) |
| Cuba | 2005 to 2009 | 0.98 (0.935, 1.027) |
| Cuba | 2010 to 2014 | 0.969 (0.925, 1.016) |
| Cuba | 2015 to 2019 | 0.952 (0.909, 0.996) |
| Cyprus | 1990 to 1994 | 1 (1, 1) |
| Cyprus | 1995 to 1999 | 0.99 (0.806, 1.214) |
| Cyprus | 2000 to 2004 | 0.965 (0.785, 1.185) |
| Cyprus | 2005 to 2009 | 0.95 (0.779, 1.16) |
| Cyprus | 2010 to 2014 | 0.944 (0.78, 1.141) |
| Cyprus | 2015 to 2019 | 0.935 (0.779, 1.122) |
| Czechia | 1990 to 1994 | 1 (1, 1) |
| Czechia | 1995 to 1999 | 0.978 (0.898, 1.065) |
| Czechia | 2000 to 2004 | 0.963 (0.882, 1.051) |
| Czechia | 2005 to 2009 | 0.951 (0.872, 1.038) |
| Czechia | 2010 to 2014 | 0.949 (0.871, 1.034) |
| Czechia | 2015 to 2019 | 0.951 (0.873, 1.036) |
| Côte d'Ivoire | 1990 to 1994 | 1 (1, 1) |
| Côte d'Ivoire | 1995 to 1999 | 1.106 (1.045, 1.172) |
| Côte d'Ivoire | 2000 to 2004 | 1.122 (1.06, 1.187) |
| Côte d'Ivoire | 2005 to 2009 | 1.099 (1.04, 1.16) |
| Côte d'Ivoire | 2010 to 2014 | 1.07 (1.014, 1.128) |
| Côte d'Ivoire | 2015 to 2019 | 1.04 (0.988, 1.094) |
| Democratic People's Republic of Korea | 1990 to 1994 | 1 (1, 1) |
| Democratic People's Republic of Korea | 1995 to 1999 | 0.995 (0.934, 1.06) |
| Democratic People's Republic of Korea | 2000 to 2004 | 0.983 (0.922, 1.049) |
| Democratic People's Republic of Korea | 2005 to 2009 | 0.949 (0.89, 1.013) |
| Democratic People's Republic of Korea | 2010 to 2014 | 0.921 (0.865, 0.98) |
| Democratic People's Republic of Korea | 2015 to 2019 | 0.898 (0.845, 0.954) |
| Democratic Republic of the Congo | 1990 to 1994 | 1 (1, 1) |
| Democratic Republic of the Congo | 1995 to 1999 | 1 (0.962, 1.04) |
| Democratic Republic of the Congo | 2000 to 2004 | 0.999 (0.961, 1.038) |
| Democratic Republic of the Congo | 2005 to 2009 | 1.002 (0.966, 1.04) |
| Democratic Republic of the Congo | 2010 to 2014 | 0.998 (0.964, 1.034) |
| Democratic Republic of the Congo | 2015 to 2019 | 0.946 (0.915, 0.979) |
| Denmark | 1990 to 1994 | 1 (1, 1) |
| Denmark | 1995 to 1999 | 0.988 (0.905, 1.078) |
| Denmark | 2000 to 2004 | 0.976 (0.891, 1.069) |
| Denmark | 2005 to 2009 | 0.967 (0.882, 1.06) |
| Denmark | 2010 to 2014 | 0.966 (0.884, 1.056) |
| Denmark | 2015 to 2019 | 0.962 (0.883, 1.047) |
| Djibouti | 1990 to 1994 | 1 (1, 1) |
| Djibouti | 1995 to 1999 | 0.969 (0.744, 1.263) |
| Djibouti | 2000 to 2004 | 0.925 (0.713, 1.2) |
| Djibouti | 2005 to 2009 | 0.874 (0.679, 1.124) |
| Djibouti | 2010 to 2014 | 0.81 (0.637, 1.03) |
| Djibouti | 2015 to 2019 | 0.753 (0.598, 0.948) |
| Dominica | 1990 to 1994 | 1 (1, 1) |
| Dominica | 1995 to 1999 | 1.01 (0.511, 1.996) |
| Dominica | 2000 to 2004 | 0.992 (0.488, 2.017) |
| Dominica | 2005 to 2009 | 0.978 (0.477, 2.006) |
| Dominica | 2010 to 2014 | 0.963 (0.476, 1.946) |
| Dominica | 2015 to 2019 | 0.973 (0.498, 1.899) |
| Dominican Republic | 1990 to 1994 | 1 (1, 1) |
| Dominican Republic | 1995 to 1999 | 0.978 (0.927, 1.031) |
| Dominican Republic | 2000 to 2004 | 0.951 (0.901, 1.004) |
| Dominican Republic | 2005 to 2009 | 0.922 (0.875, 0.973) |
| Dominican Republic | 2010 to 2014 | 0.896 (0.851, 0.943) |
| Dominican Republic | 2015 to 2019 | 0.871 (0.829, 0.916) |
| Ecuador | 1990 to 1994 | 1 (1, 1) |
| Ecuador | 1995 to 1999 | 0.984 (0.936, 1.035) |
| Ecuador | 2000 to 2004 | 0.973 (0.926, 1.023) |
| Ecuador | 2005 to 2009 | 0.965 (0.919, 1.013) |
| Ecuador | 2010 to 2014 | 0.953 (0.91, 0.999) |
| Ecuador | 2015 to 2019 | 0.933 (0.892, 0.976) |
| Egypt | 1990 to 1994 | 1 (1, 1) |
| Egypt | 1995 to 1999 | 0.971 (0.954, 0.989) |
| Egypt | 2000 to 2004 | 0.942 (0.925, 0.959) |
| Egypt | 2005 to 2009 | 0.914 (0.899, 0.93) |
| Egypt | 2010 to 2014 | 0.887 (0.873, 0.902) |
| Egypt | 2015 to 2019 | 0.851 (0.837, 0.865) |
| El Salvador | 1990 to 1994 | 1 (1, 1) |
| El Salvador | 1995 to 1999 | 0.975 (0.92, 1.033) |
| El Salvador | 2000 to 2004 | 0.94 (0.887, 0.997) |
| El Salvador | 2005 to 2009 | 0.917 (0.865, 0.972) |
| El Salvador | 2010 to 2014 | 0.901 (0.852, 0.953) |
| El Salvador | 2015 to 2019 | 0.89 (0.843, 0.94) |
| Equatorial Guinea | 1990 to 1994 | 1 (1, 1) |
| Equatorial Guinea | 1995 to 1999 | 0.942 (0.755, 1.174) |
| Equatorial Guinea | 2000 to 2004 | 0.817 (0.655, 1.019) |
| Equatorial Guinea | 2005 to 2009 | 0.693 (0.559, 0.859) |
| Equatorial Guinea | 2010 to 2014 | 0.594 (0.485, 0.727) |
| Equatorial Guinea | 2015 to 2019 | 0.527 (0.436, 0.638) |
| Eritrea | 1990 to 1994 | 1 (1, 1) |
| Eritrea | 1995 to 1999 | 0.955 (0.867, 1.052) |
| Eritrea | 2000 to 2004 | 0.904 (0.821, 0.996) |
| Eritrea | 2005 to 2009 | 0.869 (0.791, 0.954) |
| Eritrea | 2010 to 2014 | 0.83 (0.76, 0.907) |
| Eritrea | 2015 to 2019 | 0.776 (0.713, 0.845) |
| Estonia | 1990 to 1994 | 1 (1, 1) |
| Estonia | 1995 to 1999 | 0.996 (0.795, 1.247) |
| Estonia | 2000 to 2004 | 0.988 (0.78, 1.253) |
| Estonia | 2005 to 2009 | 0.975 (0.767, 1.237) |
| Estonia | 2010 to 2014 | 0.968 (0.766, 1.224) |
| Estonia | 2015 to 2019 | 0.951 (0.756, 1.196) |
| Eswatini | 1990 to 1994 | 1 (1, 1) |
| Eswatini | 1995 to 1999 | 0.974 (0.81, 1.173) |
| Eswatini | 2000 to 2004 | 0.956 (0.793, 1.153) |
| Eswatini | 2005 to 2009 | 0.938 (0.781, 1.126) |
| Eswatini | 2010 to 2014 | 0.899 (0.754, 1.072) |
| Eswatini | 2015 to 2019 | 0.849 (0.716, 1.006) |
| Ethiopia | 1990 to 1994 | 1 (1, 1) |
| Ethiopia | 1995 to 1999 | 1.008 (0.984, 1.032) |
| Ethiopia | 2000 to 2004 | 1.04 (1.016, 1.065) |
| Ethiopia | 2005 to 2009 | 1.069 (1.045, 1.093) |
| Ethiopia | 2010 to 2014 | 1.048 (1.026, 1.07) |
| Ethiopia | 2015 to 2019 | 0.974 (0.955, 0.994) |
| Fiji | 1990 to 1994 | 1 (1, 1) |
| Fiji | 1995 to 1999 | 0.948 (0.76, 1.183) |
| Fiji | 2000 to 2004 | 0.927 (0.739, 1.163) |
| Fiji | 2005 to 2009 | 0.917 (0.732, 1.147) |
| Fiji | 2010 to 2014 | 0.901 (0.725, 1.12) |
| Fiji | 2015 to 2019 | 0.874 (0.707, 1.081) |
| Finland | 1990 to 1994 | 1 (1, 1) |
| Finland | 1995 to 1999 | 0.987 (0.901, 1.081) |
| Finland | 2000 to 2004 | 0.974 (0.886, 1.071) |
| Finland | 2005 to 2009 | 0.963 (0.876, 1.058) |
| Finland | 2010 to 2014 | 0.956 (0.873, 1.047) |
| Finland | 2015 to 2019 | 0.953 (0.873, 1.04) |
| France | 1990 to 1994 | 1 (1, 1) |
| France | 1995 to 1999 | 0.993 (0.967, 1.02) |
| France | 2000 to 2004 | 0.984 (0.957, 1.012) |
| France | 2005 to 2009 | 0.976 (0.95, 1.004) |
| France | 2010 to 2014 | 0.97 (0.944, 0.996) |
| France | 2015 to 2019 | 0.964 (0.939, 0.99) |
| Gabon | 1990 to 1994 | 1 (1, 1) |
| Gabon | 1995 to 1999 | 0.971 (0.789, 1.195) |
| Gabon | 2000 to 2004 | 0.941 (0.763, 1.161) |
| Gabon | 2005 to 2009 | 0.916 (0.747, 1.125) |
| Gabon | 2010 to 2014 | 0.868 (0.714, 1.056) |
| Gabon | 2015 to 2019 | 0.804 (0.667, 0.97) |
| Gambia | 1990 to 1994 | 1 (1, 1) |
| Gambia | 1995 to 1999 | 0.928 (0.787, 1.094) |
| Gambia | 2000 to 2004 | 0.845 (0.716, 0.998) |
| Gambia | 2005 to 2009 | 0.816 (0.694, 0.959) |
| Gambia | 2010 to 2014 | 0.779 (0.668, 0.908) |
| Gambia | 2015 to 2019 | 0.735 (0.635, 0.851) |
| Georgia | 1990 to 1994 | 1 (1, 1) |
| Georgia | 1995 to 1999 | 0.883 (0.807, 0.966) |
| Georgia | 2000 to 2004 | 0.772 (0.7, 0.85) |
| Georgia | 2005 to 2009 | 0.686 (0.621, 0.758) |
| Georgia | 2010 to 2014 | 0.631 (0.572, 0.696) |
| Georgia | 2015 to 2019 | 0.579 (0.525, 0.638) |
| Germany | 1990 to 1994 | 1 (1, 1) |
| Germany | 1995 to 1999 | 0.991 (0.969, 1.013) |
| Germany | 2000 to 2004 | 0.982 (0.959, 1.006) |
| Germany | 2005 to 2009 | 0.974 (0.95, 0.998) |
| Germany | 2010 to 2014 | 0.969 (0.947, 0.993) |
| Germany | 2015 to 2019 | 0.966 (0.945, 0.988) |
| Ghana | 1990 to 1994 | 1 (1, 1) |
| Ghana | 1995 to 1999 | 1.112 (1.059, 1.167) |
| Ghana | 2000 to 2004 | 1.124 (1.072, 1.179) |
| Ghana | 2005 to 2009 | 1.106 (1.056, 1.157) |
| Ghana | 2010 to 2014 | 1.067 (1.022, 1.114) |
| Ghana | 2015 to 2019 | 1.006 (0.965, 1.049) |
| Greece | 1990 to 1994 | 1 (1, 1) |
| Greece | 1995 to 1999 | 0.982 (0.922, 1.047) |
| Greece | 2000 to 2004 | 0.959 (0.899, 1.024) |
| Greece | 2005 to 2009 | 0.948 (0.887, 1.013) |
| Greece | 2010 to 2014 | 0.938 (0.878, 1.003) |
| Greece | 2015 to 2019 | 0.934 (0.874, 0.998) |
| Greenland | 1990 to 1994 | 1 (1, 1) |
| Greenland | 1995 to 1999 | 1.032 (0.441, 2.411) |
| Greenland | 2000 to 2004 | 1.037 (0.421, 2.554) |
| Greenland | 2005 to 2009 | 1.035 (0.415, 2.579) |
| Greenland | 2010 to 2014 | 1.031 (0.428, 2.485) |
| Greenland | 2015 to 2019 | 1.039 (0.443, 2.436) |
| Grenada | 1990 to 1994 | 1 (1, 1) |
| Grenada | 1995 to 1999 | 0.963 (0.535, 1.732) |
| Grenada | 2000 to 2004 | 0.921 (0.511, 1.662) |
| Grenada | 2005 to 2009 | 0.929 (0.52, 1.66) |
| Grenada | 2010 to 2014 | 0.922 (0.52, 1.634) |
| Grenada | 2015 to 2019 | 0.901 (0.512, 1.587) |
| Guam | 1990 to 1994 | 1 (1, 1) |
| Guam | 1995 to 1999 | 0.965 (0.541, 1.72) |
| Guam | 2000 to 2004 | 0.942 (0.515, 1.724) |
| Guam | 2005 to 2009 | 0.912 (0.496, 1.677) |
| Guam | 2010 to 2014 | 0.951 (0.528, 1.71) |
| Guam | 2015 to 2019 | 0.915 (0.517, 1.619) |
| Guatemala | 1990 to 1994 | 1 (1, 1) |
| Guatemala | 1995 to 1999 | 0.987 (0.944, 1.032) |
| Guatemala | 2000 to 2004 | 0.965 (0.923, 1.008) |
| Guatemala | 2005 to 2009 | 0.945 (0.906, 0.985) |
| Guatemala | 2010 to 2014 | 0.927 (0.891, 0.964) |
| Guatemala | 2015 to 2019 | 0.914 (0.88, 0.95) |
| Guinea | 1990 to 1994 | 1 (1, 1) |
| Guinea | 1995 to 1999 | 1.024 (0.955, 1.099) |
| Guinea | 2000 to 2004 | 0.999 (0.931, 1.072) |
| Guinea | 2005 to 2009 | 0.968 (0.904, 1.036) |
| Guinea | 2010 to 2014 | 0.954 (0.894, 1.018) |
| Guinea | 2015 to 2019 | 0.925 (0.869, 0.984) |
| Guinea-Bissau | 1990 to 1994 | 1 (1, 1) |
| Guinea-Bissau | 1995 to 1999 | 1.011 (0.849, 1.203) |
| Guinea-Bissau | 2000 to 2004 | 0.996 (0.838, 1.184) |
| Guinea-Bissau | 2005 to 2009 | 0.962 (0.814, 1.137) |
| Guinea-Bissau | 2010 to 2014 | 0.907 (0.773, 1.065) |
| Guinea-Bissau | 2015 to 2019 | 0.854 (0.733, 0.996) |
| Guyana | 1990 to 1994 | 1 (1, 1) |
| Guyana | 1995 to 1999 | 0.977 (0.804, 1.186) |
| Guyana | 2000 to 2004 | 0.964 (0.788, 1.179) |
| Guyana | 2005 to 2009 | 0.957 (0.781, 1.173) |
| Guyana | 2010 to 2014 | 0.944 (0.774, 1.151) |
| Guyana | 2015 to 2019 | 0.917 (0.757, 1.11) |
| Haiti | 1990 to 1994 | 1 (1, 1) |
| Haiti | 1995 to 1999 | 0.994 (0.934, 1.057) |
| Haiti | 2000 to 2004 | 0.969 (0.911, 1.03) |
| Haiti | 2005 to 2009 | 0.95 (0.895, 1.007) |
| Haiti | 2010 to 2014 | 0.932 (0.881, 0.985) |
| Haiti | 2015 to 2019 | 0.902 (0.855, 0.952) |
| Honduras | 1990 to 1994 | 1 (1, 1) |
| Honduras | 1995 to 1999 | 0.983 (0.92, 1.051) |
| Honduras | 2000 to 2004 | 0.962 (0.901, 1.028) |
| Honduras | 2005 to 2009 | 0.945 (0.888, 1.007) |
| Honduras | 2010 to 2014 | 0.924 (0.871, 0.981) |
| Honduras | 2015 to 2019 | 0.91 (0.859, 0.964) |
| Hungary | 1990 to 1994 | 1 (1, 1) |
| Hungary | 1995 to 1999 | 0.986 (0.906, 1.073) |
| Hungary | 2000 to 2004 | 0.97 (0.889, 1.059) |
| Hungary | 2005 to 2009 | 0.961 (0.881, 1.049) |
| Hungary | 2010 to 2014 | 0.958 (0.879, 1.044) |
| Hungary | 2015 to 2019 | 0.953 (0.875, 1.038) |
| Iceland | 1990 to 1994 | 1 (1, 1) |
| Iceland | 1995 to 1999 | 0.989 (0.679, 1.439) |
| Iceland | 2000 to 2004 | 0.946 (0.643, 1.391) |
| Iceland | 2005 to 2009 | 0.943 (0.645, 1.378) |
| Iceland | 2010 to 2014 | 0.933 (0.646, 1.346) |
| Iceland | 2015 to 2019 | 0.925 (0.651, 1.315) |
| India | 1990 to 1994 | 1 (1, 1) |
| India | 1995 to 1999 | 1.005 (0.996, 1.015) |
| India | 2000 to 2004 | 0.956 (0.947, 0.964) |
| India | 2005 to 2009 | 0.861 (0.853, 0.869) |
| India | 2010 to 2014 | 0.796 (0.789, 0.803) |
| India | 2015 to 2019 | 0.745 (0.738, 0.751) |
| Indonesia | 1990 to 1994 | 1 (1, 1) |
| Indonesia | 1995 to 1999 | 0.947 (0.939, 0.956) |
| Indonesia | 2000 to 2004 | 0.911 (0.902, 0.92) |
| Indonesia | 2005 to 2009 | 0.88 (0.872, 0.889) |
| Indonesia | 2010 to 2014 | 0.834 (0.827, 0.842) |
| Indonesia | 2015 to 2019 | 0.783 (0.776, 0.79) |
| Iran (Islamic Republic of) | 1990 to 1994 | 1 (1, 1) |
| Iran (Islamic Republic of) | 1995 to 1999 | 0.983 (0.967, 1) |
| Iran (Islamic Republic of) | 2000 to 2004 | 0.98 (0.965, 0.996) |
| Iran (Islamic Republic of) | 2005 to 2009 | 0.955 (0.94, 0.97) |
| Iran (Islamic Republic of) | 2010 to 2014 | 0.939 (0.924, 0.953) |
| Iran (Islamic Republic of) | 2015 to 2019 | 0.894 (0.88, 0.907) |
| Iraq | 1990 to 1994 | 1 (1, 1) |
| Iraq | 1995 to 1999 | 0.986 (0.955, 1.018) |
| Iraq | 2000 to 2004 | 0.957 (0.927, 0.987) |
| Iraq | 2005 to 2009 | 0.922 (0.895, 0.951) |
| Iraq | 2010 to 2014 | 0.884 (0.859, 0.91) |
| Iraq | 2015 to 2019 | 0.846 (0.822, 0.87) |
| Ireland | 1990 to 1994 | 1 (1, 1) |
| Ireland | 1995 to 1999 | 0.992 (0.898, 1.095) |
| Ireland | 2000 to 2004 | 0.977 (0.885, 1.078) |
| Ireland | 2005 to 2009 | 0.967 (0.878, 1.065) |
| Ireland | 2010 to 2014 | 0.952 (0.866, 1.047) |
| Ireland | 2015 to 2019 | 0.937 (0.853, 1.03) |
| Israel | 1990 to 1994 | 1 (1, 1) |
| Israel | 1995 to 1999 | 0.989 (0.912, 1.073) |
| Israel | 2000 to 2004 | 0.978 (0.902, 1.059) |
| Israel | 2005 to 2009 | 0.968 (0.896, 1.047) |
| Israel | 2010 to 2014 | 0.962 (0.893, 1.037) |
| Israel | 2015 to 2019 | 0.956 (0.888, 1.028) |
| Italy | 1990 to 1994 | 1 (1, 1) |
| Italy | 1995 to 1999 | 0.973 (0.954, 0.993) |
| Italy | 2000 to 2004 | 0.951 (0.931, 0.971) |
| Italy | 2005 to 2009 | 0.938 (0.917, 0.958) |
| Italy | 2010 to 2014 | 0.927 (0.907, 0.947) |
| Italy | 2015 to 2019 | 0.92 (0.9, 0.939) |
| Jamaica | 1990 to 1994 | 1 (1, 1) |
| Jamaica | 1995 to 1999 | 0.989 (0.883, 1.107) |
| Jamaica | 2000 to 2004 | 0.963 (0.858, 1.081) |
| Jamaica | 2005 to 2009 | 0.941 (0.839, 1.056) |
| Jamaica | 2010 to 2014 | 0.923 (0.826, 1.032) |
| Jamaica | 2015 to 2019 | 0.904 (0.812, 1.007) |
| Japan | 1990 to 1994 | 1 (1, 1) |
| Japan | 1995 to 1999 | 0.989 (0.969, 1.009) |
| Japan | 2000 to 2004 | 0.975 (0.956, 0.996) |
| Japan | 2005 to 2009 | 0.961 (0.941, 0.981) |
| Japan | 2010 to 2014 | 0.949 (0.93, 0.969) |
| Japan | 2015 to 2019 | 0.945 (0.926, 0.965) |
| Jordan | 1990 to 1994 | 1 (1, 1) |
| Jordan | 1995 to 1999 | 0.98 (0.905, 1.061) |
| Jordan | 2000 to 2004 | 0.95 (0.879, 1.028) |
| Jordan | 2005 to 2009 | 0.92 (0.854, 0.991) |
| Jordan | 2010 to 2014 | 0.886 (0.826, 0.951) |
| Jordan | 2015 to 2019 | 0.86 (0.803, 0.921) |
| Kazakhstan | 1990 to 1994 | 1 (1, 1) |
| Kazakhstan | 1995 to 1999 | 1 (0.953, 1.05) |
| Kazakhstan | 2000 to 2004 | 0.993 (0.944, 1.044) |
| Kazakhstan | 2005 to 2009 | 0.975 (0.928, 1.025) |
| Kazakhstan | 2010 to 2014 | 0.948 (0.904, 0.994) |
| Kazakhstan | 2015 to 2019 | 0.919 (0.878, 0.962) |
| Kenya | 1990 to 1994 | 1 (1, 1) |
| Kenya | 1995 to 1999 | 0.931 (0.899, 0.963) |
| Kenya | 2000 to 2004 | 0.849 (0.82, 0.879) |
| Kenya | 2005 to 2009 | 0.83 (0.802, 0.858) |
| Kenya | 2010 to 2014 | 0.787 (0.763, 0.813) |
| Kenya | 2015 to 2019 | 0.724 (0.702, 0.746) |
| Kiribati | 1990 to 1994 | 1 (1, 1) |
| Kiribati | 1995 to 1999 | 0.96 (0.47, 1.961) |
| Kiribati | 2000 to 2004 | 0.992 (0.481, 2.05) |
| Kiribati | 2005 to 2009 | 0.948 (0.47, 1.909) |
| Kiribati | 2010 to 2014 | 0.938 (0.482, 1.826) |
| Kiribati | 2015 to 2019 | 0.905 (0.477, 1.715) |
| Kuwait | 1990 to 1994 | 1 (1, 1) |
| Kuwait | 1995 to 1999 | 0.982 (0.881, 1.094) |
| Kuwait | 2000 to 2004 | 0.954 (0.855, 1.063) |
| Kuwait | 2005 to 2009 | 0.931 (0.839, 1.033) |
| Kuwait | 2010 to 2014 | 0.903 (0.819, 0.996) |
| Kuwait | 2015 to 2019 | 0.886 (0.809, 0.971) |
| Kyrgyzstan | 1990 to 1994 | 1 (1, 1) |
| Kyrgyzstan | 1995 to 1999 | 0.994 (0.902, 1.095) |
| Kyrgyzstan | 2000 to 2004 | 0.988 (0.895, 1.091) |
| Kyrgyzstan | 2005 to 2009 | 0.976 (0.886, 1.075) |
| Kyrgyzstan | 2010 to 2014 | 0.953 (0.869, 1.045) |
| Kyrgyzstan | 2015 to 2019 | 0.931 (0.852, 1.017) |
| Lao People's Democratic Republic | 1990 to 1994 | 1 (1, 1) |
| Lao People's Democratic Republic | 1995 to 1999 | 0.977 (0.887, 1.077) |
| Lao People's Democratic Republic | 2000 to 2004 | 0.949 (0.862, 1.046) |
| Lao People's Democratic Republic | 2005 to 2009 | 0.915 (0.834, 1.004) |
| Lao People's Democratic Republic | 2010 to 2014 | 0.865 (0.791, 0.945) |
| Lao People's Democratic Republic | 2015 to 2019 | 0.817 (0.749, 0.892) |
| Latvia | 1990 to 1994 | 1 (1, 1) |
| Latvia | 1995 to 1999 | 0.994 (0.861, 1.148) |
| Latvia | 2000 to 2004 | 0.98 (0.84, 1.143) |
| Latvia | 2005 to 2009 | 0.965 (0.825, 1.129) |
| Latvia | 2010 to 2014 | 0.953 (0.815, 1.114) |
| Latvia | 2015 to 2019 | 0.943 (0.808, 1.102) |
| Lebanon | 1990 to 1994 | 1 (1, 1) |
| Lebanon | 1995 to 1999 | 0.968 (0.895, 1.046) |
| Lebanon | 2000 to 2004 | 0.93 (0.86, 1.005) |
| Lebanon | 2005 to 2009 | 0.88 (0.815, 0.95) |
| Lebanon | 2010 to 2014 | 0.825 (0.767, 0.888) |
| Lebanon | 2015 to 2019 | 0.803 (0.748, 0.862) |
| Lesotho | 1990 to 1994 | 1 (1, 1) |
| Lesotho | 1995 to 1999 | 0.981 (0.868, 1.109) |
| Lesotho | 2000 to 2004 | 0.967 (0.853, 1.096) |
| Lesotho | 2005 to 2009 | 0.947 (0.836, 1.072) |
| Lesotho | 2010 to 2014 | 0.915 (0.812, 1.03) |
| Lesotho | 2015 to 2019 | 0.875 (0.78, 0.981) |
| Liberia | 1990 to 1994 | 1 (1, 1) |
| Liberia | 1995 to 1999 | 0.986 (0.867, 1.121) |
| Liberia | 2000 to 2004 | 0.943 (0.831, 1.07) |
| Liberia | 2005 to 2009 | 0.889 (0.788, 1.004) |
| Liberia | 2010 to 2014 | 0.867 (0.773, 0.972) |
| Liberia | 2015 to 2019 | 0.832 (0.745, 0.93) |
| Libya | 1990 to 1994 | 1 (1, 1) |
| Libya | 1995 to 1999 | 0.965 (0.9, 1.034) |
| Libya | 2000 to 2004 | 0.936 (0.875, 1.001) |
| Libya | 2005 to 2009 | 0.897 (0.84, 0.957) |
| Libya | 2010 to 2014 | 0.87 (0.816, 0.927) |
| Libya | 2015 to 2019 | 0.867 (0.813, 0.923) |
| Lithuania | 1990 to 1994 | 1 (1, 1) |
| Lithuania | 1995 to 1999 | 0.992 (0.884, 1.114) |
| Lithuania | 2000 to 2004 | 0.977 (0.862, 1.107) |
| Lithuania | 2005 to 2009 | 0.964 (0.848, 1.095) |
| Lithuania | 2010 to 2014 | 0.953 (0.839, 1.082) |
| Lithuania | 2015 to 2019 | 0.949 (0.837, 1.076) |
| Luxembourg | 1990 to 1994 | 1 (1, 1) |
| Luxembourg | 1995 to 1999 | 0.971 (0.714, 1.322) |
| Luxembourg | 2000 to 2004 | 0.966 (0.704, 1.326) |
| Luxembourg | 2005 to 2009 | 0.958 (0.699, 1.314) |
| Luxembourg | 2010 to 2014 | 0.958 (0.71, 1.293) |
| Luxembourg | 2015 to 2019 | 0.946 (0.716, 1.25) |
| Madagascar | 1990 to 1994 | 1 (1, 1) |
| Madagascar | 1995 to 1999 | 0.979 (0.923, 1.038) |
| Madagascar | 2000 to 2004 | 0.935 (0.881, 0.992) |
| Madagascar | 2005 to 2009 | 0.902 (0.852, 0.956) |
| Madagascar | 2010 to 2014 | 0.88 (0.834, 0.929) |
| Madagascar | 2015 to 2019 | 0.83 (0.788, 0.874) |
| Malawi | 1990 to 1994 | 1 (1, 1) |
| Malawi | 1995 to 1999 | 0.974 (0.918, 1.033) |
| Malawi | 2000 to 2004 | 0.938 (0.884, 0.995) |
| Malawi | 2005 to 2009 | 0.908 (0.858, 0.962) |
| Malawi | 2010 to 2014 | 0.863 (0.817, 0.911) |
| Malawi | 2015 to 2019 | 0.805 (0.765, 0.848) |
| Malaysia | 1990 to 1994 | 1 (1, 1) |
| Malaysia | 1995 to 1999 | 0.939 (0.904, 0.976) |
| Malaysia | 2000 to 2004 | 0.888 (0.855, 0.923) |
| Malaysia | 2005 to 2009 | 0.85 (0.819, 0.882) |
| Malaysia | 2010 to 2014 | 0.821 (0.792, 0.851) |
| Malaysia | 2015 to 2019 | 0.785 (0.758, 0.812) |
| Maldives | 1990 to 1994 | 1 (1, 1) |
| Maldives | 1995 to 1999 | 0.98 (0.656, 1.464) |
| Maldives | 2000 to 2004 | 0.947 (0.637, 1.407) |
| Maldives | 2005 to 2009 | 0.904 (0.62, 1.319) |
| Maldives | 2010 to 2014 | 0.839 (0.588, 1.197) |
| Maldives | 2015 to 2019 | 0.794 (0.564, 1.119) |
| Mali | 1990 to 1994 | 1 (1, 1) |
| Mali | 1995 to 1999 | 0.967 (0.918, 1.019) |
| Mali | 2000 to 2004 | 0.901 (0.855, 0.95) |
| Mali | 2005 to 2009 | 0.863 (0.82, 0.907) |
| Mali | 2010 to 2014 | 0.853 (0.813, 0.895) |
| Mali | 2015 to 2019 | 0.809 (0.773, 0.847) |
| Malta | 1990 to 1994 | 1 (1, 1) |
| Malta | 1995 to 1999 | 1.004 (0.728, 1.385) |
| Malta | 2000 to 2004 | 0.992 (0.713, 1.379) |
| Malta | 2005 to 2009 | 0.977 (0.706, 1.352) |
| Malta | 2010 to 2014 | 0.974 (0.712, 1.333) |
| Malta | 2015 to 2019 | 0.964 (0.711, 1.309) |
| Marshall Islands | 1990 to 1994 | 1 (1, 1) |
| Marshall Islands | 1995 to 1999 | 0.934 (0.374, 2.333) |
| Marshall Islands | 2000 to 2004 | 0.871 (0.343, 2.212) |
| Marshall Islands | 2005 to 2009 | 0.845 (0.335, 2.131) |
| Marshall Islands | 2010 to 2014 | 0.903 (0.374, 2.182) |
| Marshall Islands | 2015 to 2019 | 0.858 (0.363, 2.027) |
| Mauritania | 1990 to 1994 | 1 (1, 1) |
| Mauritania | 1995 to 1999 | 0.959 (0.847, 1.086) |
| Mauritania | 2000 to 2004 | 0.917 (0.809, 1.04) |
| Mauritania | 2005 to 2009 | 0.882 (0.78, 0.996) |
| Mauritania | 2010 to 2014 | 0.827 (0.736, 0.93) |
| Mauritania | 2015 to 2019 | 0.768 (0.687, 0.859) |
| Mauritius | 1990 to 1994 | 1 (1, 1) |
| Mauritius | 1995 to 1999 | 0.981 (0.829, 1.16) |
| Mauritius | 2000 to 2004 | 0.962 (0.808, 1.146) |
| Mauritius | 2005 to 2009 | 0.94 (0.789, 1.12) |
| Mauritius | 2010 to 2014 | 0.896 (0.755, 1.062) |
| Mauritius | 2015 to 2019 | 0.857 (0.724, 1.015) |
| Mexico | 1990 to 1994 | 1 (1, 1) |
| Mexico | 1995 to 1999 | 0.973 (0.959, 0.988) |
| Mexico | 2000 to 2004 | 0.953 (0.938, 0.968) |
| Mexico | 2005 to 2009 | 0.937 (0.923, 0.951) |
| Mexico | 2010 to 2014 | 0.921 (0.908, 0.935) |
| Mexico | 2015 to 2019 | 0.902 (0.889, 0.915) |
| Micronesia (Federated States of) | 1990 to 1994 | 1 (1, 1) |
| Micronesia (Federated States of) | 1995 to 1999 | 0.969 (0.514, 1.826) |
| Micronesia (Federated States of) | 2000 to 2004 | 0.908 (0.474, 1.739) |
| Micronesia (Federated States of) | 2005 to 2009 | 0.849 (0.44, 1.638) |
| Micronesia (Federated States of) | 2010 to 2014 | 0.83 (0.436, 1.581) |
| Micronesia (Federated States of) | 2015 to 2019 | 0.821 (0.434, 1.556) |
| Monaco | 1990 to 1994 | 1 (1, 1) |
| Monaco | 1995 to 1999 | 0.961 (0.257, 3.586) |
| Monaco | 2000 to 2004 | 1.103 (0.296, 4.112) |
| Monaco | 2005 to 2009 | 1.13 (0.306, 4.171) |
| Monaco | 2010 to 2014 | 0.966 (0.265, 3.516) |
| Monaco | 2015 to 2019 | 0.978 (0.277, 3.456) |
| Mongolia | 1990 to 1994 | 1 (1, 1) |
| Mongolia | 1995 to 1999 | 0.985 (0.879, 1.104) |
| Mongolia | 2000 to 2004 | 0.953 (0.849, 1.068) |
| Mongolia | 2005 to 2009 | 0.911 (0.814, 1.02) |
| Mongolia | 2010 to 2014 | 0.878 (0.787, 0.979) |
| Mongolia | 2015 to 2019 | 0.85 (0.764, 0.945) |
| Montenegro | 1990 to 1994 | 1 (1, 1) |
| Montenegro | 1995 to 1999 | 1.018 (0.734, 1.412) |
| Montenegro | 2000 to 2004 | 1.004 (0.714, 1.413) |
| Montenegro | 2005 to 2009 | 0.974 (0.691, 1.371) |
| Montenegro | 2010 to 2014 | 0.986 (0.708, 1.374) |
| Montenegro | 2015 to 2019 | 0.97 (0.701, 1.342) |
| Morocco | 1990 to 1994 | 1 (1, 1) |
| Morocco | 1995 to 1999 | 0.981 (0.952, 1.011) |
| Morocco | 2000 to 2004 | 0.954 (0.925, 0.983) |
| Morocco | 2005 to 2009 | 0.924 (0.897, 0.952) |
| Morocco | 2010 to 2014 | 0.891 (0.865, 0.917) |
| Morocco | 2015 to 2019 | 0.859 (0.835, 0.884) |
| Mozambique | 1990 to 1994 | 1 (1, 1) |
| Mozambique | 1995 to 1999 | 0.969 (0.922, 1.018) |
| Mozambique | 2000 to 2004 | 0.934 (0.889, 0.981) |
| Mozambique | 2005 to 2009 | 0.905 (0.862, 0.949) |
| Mozambique | 2010 to 2014 | 0.861 (0.823, 0.901) |
| Mozambique | 2015 to 2019 | 0.807 (0.772, 0.843) |
| Myanmar | 1990 to 1994 | 1 (1, 1) |
| Myanmar | 1995 to 1999 | 0.982 (0.961, 1.003) |
| Myanmar | 2000 to 2004 | 0.947 (0.927, 0.968) |
| Myanmar | 2005 to 2009 | 0.898 (0.878, 0.918) |
| Myanmar | 2010 to 2014 | 0.827 (0.809, 0.845) |
| Myanmar | 2015 to 2019 | 0.753 (0.737, 0.769) |
| Namibia | 1990 to 1994 | 1 (1, 1) |
| Namibia | 1995 to 1999 | 0.976 (0.851, 1.12) |
| Namibia | 2000 to 2004 | 0.944 (0.821, 1.084) |
| Namibia | 2005 to 2009 | 0.912 (0.795, 1.045) |
| Namibia | 2010 to 2014 | 0.863 (0.757, 0.984) |
| Namibia | 2015 to 2019 | 0.814 (0.718, 0.924) |
| Nauru | 1990 to 1994 | 1 (1, 1) |
| Nauru | 1995 to 1999 | 0.976 (0.148, 6.434) |
| Nauru | 2000 to 2004 | 0.98 (0.146, 6.563) |
| Nauru | 2005 to 2009 | 0.967 (0.15, 6.228) |
| Nauru | 2010 to 2014 | 0.953 (0.16, 5.697) |
| Nauru | 2015 to 2019 | 0.877 (0.13, 5.917) |
| Nepal | 1990 to 1994 | 1 (1, 1) |
| Nepal | 1995 to 1999 | 0.999 (0.962, 1.037) |
| Nepal | 2000 to 2004 | 0.976 (0.94, 1.014) |
| Nepal | 2005 to 2009 | 0.942 (0.907, 0.977) |
| Nepal | 2010 to 2014 | 0.913 (0.881, 0.946) |
| Nepal | 2015 to 2019 | 0.89 (0.86, 0.92) |
| Netherlands | 1990 to 1994 | 1 (1, 1) |
| Netherlands | 1995 to 1999 | 0.99 (0.939, 1.044) |
| Netherlands | 2000 to 2004 | 0.982 (0.929, 1.038) |
| Netherlands | 2005 to 2009 | 0.971 (0.918, 1.027) |
| Netherlands | 2010 to 2014 | 0.96 (0.909, 1.014) |
| Netherlands | 2015 to 2019 | 0.954 (0.905, 1.005) |
| New Zealand | 1990 to 1994 | 1 (1, 1) |
| New Zealand | 1995 to 1999 | 0.982 (0.886, 1.088) |
| New Zealand | 2000 to 2004 | 0.966 (0.868, 1.074) |
| New Zealand | 2005 to 2009 | 0.956 (0.861, 1.062) |
| New Zealand | 2010 to 2014 | 0.949 (0.858, 1.05) |
| New Zealand | 2015 to 2019 | 0.935 (0.847, 1.034) |
| Nicaragua | 1990 to 1994 | 1 (1, 1) |
| Nicaragua | 1995 to 1999 | 0.991 (0.925, 1.062) |
| Nicaragua | 2000 to 2004 | 0.973 (0.909, 1.042) |
| Nicaragua | 2005 to 2009 | 0.948 (0.887, 1.014) |
| Nicaragua | 2010 to 2014 | 0.916 (0.859, 0.977) |
| Nicaragua | 2015 to 2019 | 0.893 (0.839, 0.95) |
| Niger | 1990 to 1994 | 1 (1, 1) |
| Niger | 1995 to 1999 | 1.039 (0.979, 1.103) |
| Niger | 2000 to 2004 | 1.02 (0.961, 1.082) |
| Niger | 2005 to 2009 | 0.993 (0.938, 1.052) |
| Niger | 2010 to 2014 | 0.978 (0.926, 1.033) |
| Niger | 2015 to 2019 | 0.958 (0.91, 1.009) |
| Nigeria | 1990 to 1994 | 1 (1, 1) |
| Nigeria | 1995 to 1999 | 1.008 (0.992, 1.024) |
| Nigeria | 2000 to 2004 | 1.003 (0.987, 1.019) |
| Nigeria | 2005 to 2009 | 0.998 (0.983, 1.013) |
| Nigeria | 2010 to 2014 | 0.984 (0.97, 0.998) |
| Nigeria | 2015 to 2019 | 0.952 (0.939, 0.965) |
| Niue | 1990 to 1994 | 1 (1, 1) |
| Niue | 1995 to 1999 | 0.986 (0.005, 182.487) |
| Niue | 2000 to 2004 | 0.963 (0.003, 287.808) |
| Niue | 2005 to 2009 | 0.915 (0.003, 310.492) |
| Niue | 2010 to 2014 | 1.001 (0.004, 249.136) |
| Niue | 2015 to 2019 | 0.869 (0.003, 232.066) |
| North Macedonia | 1990 to 1994 | 1 (1, 1) |
| North Macedonia | 1995 to 1999 | 0.985 (0.827, 1.175) |
| North Macedonia | 2000 to 2004 | 0.965 (0.803, 1.16) |
| North Macedonia | 2005 to 2009 | 0.95 (0.792, 1.14) |
| North Macedonia | 2010 to 2014 | 0.941 (0.789, 1.123) |
| North Macedonia | 2015 to 2019 | 0.936 (0.789, 1.111) |
| Northern Mariana Islands | 1990 to 1994 | 1 (1, 1) |
| Northern Mariana Islands | 1995 to 1999 | 0.944 (0.365, 2.44) |
| Northern Mariana Islands | 2000 to 2004 | 1.042 (0.389, 2.787) |
| Northern Mariana Islands | 2005 to 2009 | 0.989 (0.329, 2.974) |
| Northern Mariana Islands | 2010 to 2014 | 0.937 (0.303, 2.902) |
| Northern Mariana Islands | 2015 to 2019 | 0.953 (0.312, 2.912) |
| Norway | 1990 to 1994 | 1 (1, 1) |
| Norway | 1995 to 1999 | 0.992 (0.907, 1.086) |
| Norway | 2000 to 2004 | 0.981 (0.894, 1.076) |
| Norway | 2005 to 2009 | 0.981 (0.894, 1.075) |
| Norway | 2010 to 2014 | 0.983 (0.899, 1.073) |
| Norway | 2015 to 2019 | 0.978 (0.899, 1.064) |
| Oman | 1990 to 1994 | 1 (1, 1) |
| Oman | 1995 to 1999 | 0.973 (0.901, 1.05) |
| Oman | 2000 to 2004 | 0.925 (0.857, 0.998) |
| Oman | 2005 to 2009 | 0.907 (0.842, 0.976) |
| Oman | 2010 to 2014 | 0.893 (0.835, 0.955) |
| Oman | 2015 to 2019 | 0.874 (0.821, 0.931) |
| Pakistan | 1990 to 1994 | 1 (1, 1) |
| Pakistan | 1995 to 1999 | 1.003 (0.989, 1.018) |
| Pakistan | 2000 to 2004 | 0.986 (0.972, 1) |
| Pakistan | 2005 to 2009 | 0.972 (0.959, 0.985) |
| Pakistan | 2010 to 2014 | 0.936 (0.923, 0.948) |
| Pakistan | 2015 to 2019 | 0.9 (0.889, 0.912) |
| Palau | 1990 to 1994 | 1 (1, 1) |
| Palau | 1995 to 1999 | 0.762 (0.197, 2.954) |
| Palau | 2000 to 2004 | 0.723 (0.184, 2.849) |
| Palau | 2005 to 2009 | 0.768 (0.193, 3.057) |
| Palau | 2010 to 2014 | 0.704 (0.164, 3.03) |
| Palau | 2015 to 2019 | 0.684 (0.152, 3.073) |
| Palestine | 1990 to 1994 | 1 (1, 1) |
| Palestine | 1995 to 1999 | 0.976 (0.885, 1.078) |
| Palestine | 2000 to 2004 | 0.957 (0.87, 1.053) |
| Palestine | 2005 to 2009 | 0.931 (0.85, 1.021) |
| Palestine | 2010 to 2014 | 0.886 (0.811, 0.967) |
| Palestine | 2015 to 2019 | 0.847 (0.776, 0.924) |
| Panama | 1990 to 1994 | 1 (1, 1) |
| Panama | 1995 to 1999 | 0.994 (0.91, 1.085) |
| Panama | 2000 to 2004 | 0.981 (0.898, 1.072) |
| Panama | 2005 to 2009 | 0.969 (0.889, 1.057) |
| Panama | 2010 to 2014 | 0.947 (0.87, 1.03) |
| Panama | 2015 to 2019 | 0.917 (0.846, 0.993) |
| Papua New Guinea | 1990 to 1994 | 1 (1, 1) |
| Papua New Guinea | 1995 to 1999 | 0.985 (0.908, 1.068) |
| Papua New Guinea | 2000 to 2004 | 0.971 (0.897, 1.051) |
| Papua New Guinea | 2005 to 2009 | 0.962 (0.891, 1.038) |
| Papua New Guinea | 2010 to 2014 | 0.942 (0.877, 1.013) |
| Papua New Guinea | 2015 to 2019 | 0.913 (0.852, 0.979) |
| Paraguay | 1990 to 1994 | 1 (1, 1) |
| Paraguay | 1995 to 1999 | 0.989 (0.931, 1.051) |
| Paraguay | 2000 to 2004 | 0.979 (0.921, 1.04) |
| Paraguay | 2005 to 2009 | 0.966 (0.911, 1.024) |
| Paraguay | 2010 to 2014 | 0.946 (0.895, 1) |
| Paraguay | 2015 to 2019 | 0.923 (0.874, 0.974) |
| Peru | 1990 to 1994 | 1 (1, 1) |
| Peru | 1995 to 1999 | 0.979 (0.953, 1.006) |
| Peru | 2000 to 2004 | 0.948 (0.922, 0.974) |
| Peru | 2005 to 2009 | 0.928 (0.904, 0.953) |
| Peru | 2010 to 2014 | 0.902 (0.879, 0.926) |
| Peru | 2015 to 2019 | 0.872 (0.85, 0.893) |
| Philippines | 1990 to 1994 | 1 (1, 1) |
| Philippines | 1995 to 1999 | 0.977 (0.956, 0.999) |
| Philippines | 2000 to 2004 | 0.965 (0.944, 0.986) |
| Philippines | 2005 to 2009 | 0.959 (0.94, 0.98) |
| Philippines | 2010 to 2014 | 0.932 (0.913, 0.95) |
| Philippines | 2015 to 2019 | 0.881 (0.864, 0.898) |
| Poland | 1990 to 1994 | 1 (1, 1) |
| Poland | 1995 to 1999 | 0.989 (0.947, 1.032) |
| Poland | 2000 to 2004 | 0.979 (0.936, 1.023) |
| Poland | 2005 to 2009 | 0.976 (0.934, 1.02) |
| Poland | 2010 to 2014 | 0.978 (0.937, 1.021) |
| Poland | 2015 to 2019 | 0.979 (0.939, 1.021) |
| Portugal | 1990 to 1994 | 1 (1, 1) |
| Portugal | 1995 to 1999 | 0.982 (0.926, 1.042) |
| Portugal | 2000 to 2004 | 0.964 (0.907, 1.024) |
| Portugal | 2005 to 2009 | 0.948 (0.891, 1.008) |
| Portugal | 2010 to 2014 | 0.938 (0.882, 0.997) |
| Portugal | 2015 to 2019 | 0.925 (0.871, 0.983) |
| Puerto Rico | 1990 to 1994 | 1 (1, 1) |
| Puerto Rico | 1995 to 1999 | 0.989 (0.896, 1.09) |
| Puerto Rico | 2000 to 2004 | 0.968 (0.874, 1.072) |
| Puerto Rico | 2005 to 2009 | 0.951 (0.858, 1.054) |
| Puerto Rico | 2010 to 2014 | 0.937 (0.847, 1.036) |
| Puerto Rico | 2015 to 2019 | 0.925 (0.837, 1.022) |
| Qatar | 1990 to 1994 | 1 (1, 1) |
| Qatar | 1995 to 1999 | 0.971 (0.803, 1.173) |
| Qatar | 2000 to 2004 | 0.93 (0.769, 1.124) |
| Qatar | 2005 to 2009 | 0.877 (0.739, 1.04) |
| Qatar | 2010 to 2014 | 0.835 (0.715, 0.975) |
| Qatar | 2015 to 2019 | 0.815 (0.705, 0.941) |
| Republic of Korea | 1990 to 1994 | 1 (1, 1) |
| Republic of Korea | 1995 to 1999 | 0.981 (0.956, 1.006) |
| Republic of Korea | 2000 to 2004 | 0.973 (0.948, 0.999) |
| Republic of Korea | 2005 to 2009 | 0.934 (0.909, 0.959) |
| Republic of Korea | 2010 to 2014 | 0.908 (0.884, 0.933) |
| Republic of Korea | 2015 to 2019 | 0.895 (0.872, 0.918) |
| Republic of Moldova | 1990 to 1994 | 1 (1, 1) |
| Republic of Moldova | 1995 to 1999 | 0.999 (0.912, 1.095) |
| Republic of Moldova | 2000 to 2004 | 0.99 (0.898, 1.091) |
| Republic of Moldova | 2005 to 2009 | 0.975 (0.885, 1.075) |
| Republic of Moldova | 2010 to 2014 | 0.956 (0.869, 1.051) |
| Republic of Moldova | 2015 to 2019 | 0.925 (0.843, 1.016) |
| Romania | 1990 to 1994 | 1 (1, 1) |
| Romania | 1995 to 1999 | 0.992 (0.939, 1.048) |
| Romania | 2000 to 2004 | 0.976 (0.922, 1.033) |
| Romania | 2005 to 2009 | 0.964 (0.91, 1.021) |
| Romania | 2010 to 2014 | 0.958 (0.905, 1.015) |
| Romania | 2015 to 2019 | 0.952 (0.899, 1.009) |
| Russian Federation | 1990 to 1994 | 1 (1, 1) |
| Russian Federation | 1995 to 1999 | 1.005 (0.988, 1.021) |
| Russian Federation | 2000 to 2004 | 0.996 (0.979, 1.014) |
| Russian Federation | 2005 to 2009 | 0.981 (0.964, 0.998) |
| Russian Federation | 2010 to 2014 | 0.962 (0.946, 0.979) |
| Russian Federation | 2015 to 2019 | 0.95 (0.935, 0.966) |
| Rwanda | 1990 to 1994 | 1 (1, 1) |
| Rwanda | 1995 to 1999 | 0.984 (0.9, 1.077) |
| Rwanda | 2000 to 2004 | 0.945 (0.864, 1.034) |
| Rwanda | 2005 to 2009 | 0.892 (0.817, 0.973) |
| Rwanda | 2010 to 2014 | 0.833 (0.767, 0.905) |
| Rwanda | 2015 to 2019 | 0.779 (0.72, 0.843) |
| Saint Kitts and Nevis | 1990 to 1994 | 1 (1, 1) |
| Saint Kitts and Nevis | 1995 to 1999 | 0.929 (0.391, 2.208) |
| Saint Kitts and Nevis | 2000 to 2004 | 0.918 (0.377, 2.232) |
| Saint Kitts and Nevis | 2005 to 2009 | 0.866 (0.36, 2.082) |
| Saint Kitts and Nevis | 2010 to 2014 | 0.911 (0.396, 2.097) |
| Saint Kitts and Nevis | 2015 to 2019 | 0.88 (0.394, 1.964) |
| Saint Lucia | 1990 to 1994 | 1 (1, 1) |
| Saint Lucia | 1995 to 1999 | 0.97 (0.614, 1.534) |
| Saint Lucia | 2000 to 2004 | 0.93 (0.584, 1.482) |
| Saint Lucia | 2005 to 2009 | 0.917 (0.578, 1.455) |
| Saint Lucia | 2010 to 2014 | 0.884 (0.563, 1.389) |
| Saint Lucia | 2015 to 2019 | 0.877 (0.565, 1.364) |
| Saint Vincent and the Grenadines | 1990 to 1994 | 1 (1, 1) |
| Saint Vincent and the Grenadines | 1995 to 1999 | 1.055 (0.622, 1.789) |
| Saint Vincent and the Grenadines | 2000 to 2004 | 1.008 (0.583, 1.741) |
| Saint Vincent and the Grenadines | 2005 to 2009 | 1.002 (0.579, 1.734) |
| Saint Vincent and the Grenadines | 2010 to 2014 | 0.971 (0.567, 1.664) |
| Saint Vincent and the Grenadines | 2015 to 2019 | 0.928 (0.547, 1.575) |
| Samoa | 1990 to 1994 | 1 (1, 1) |
| Samoa | 1995 to 1999 | 0.937 (0.554, 1.585) |
| Samoa | 2000 to 2004 | 0.908 (0.53, 1.554) |
| Samoa | 2005 to 2009 | 0.874 (0.512, 1.493) |
| Samoa | 2010 to 2014 | 0.89 (0.532, 1.488) |
| Samoa | 2015 to 2019 | 0.912 (0.555, 1.497) |
| San Marino | 1990 to 1994 | 1 (1, 1) |
| San Marino | 1995 to 1999 | 0.953 (0.255, 3.559) |
| San Marino | 2000 to 2004 | 0.919 (0.24, 3.52) |
| San Marino | 2005 to 2009 | 1.078 (0.292, 3.979) |
| San Marino | 2010 to 2014 | 0.918 (0.252, 3.343) |
| San Marino | 2015 to 2019 | 0.909 (0.257, 3.212) |
| Sao Tome and Principe | 1990 to 1994 | 1 (1, 1) |
| Sao Tome and Principe | 1995 to 1999 | 1.049 (0.608, 1.808) |
| Sao Tome and Principe | 2000 to 2004 | 0.973 (0.561, 1.686) |
| Sao Tome and Principe | 2005 to 2009 | 0.911 (0.533, 1.557) |
| Sao Tome and Principe | 2010 to 2014 | 0.867 (0.52, 1.445) |
| Sao Tome and Principe | 2015 to 2019 | 0.805 (0.492, 1.316) |
| Saudi Arabia | 1990 to 1994 | 1 (1, 1) |
| Saudi Arabia | 1995 to 1999 | 0.917 (0.894, 0.94) |
| Saudi Arabia | 2000 to 2004 | 0.836 (0.815, 0.857) |
| Saudi Arabia | 2005 to 2009 | 0.753 (0.735, 0.771) |
| Saudi Arabia | 2010 to 2014 | 0.701 (0.685, 0.717) |
| Saudi Arabia | 2015 to 2019 | 0.662 (0.648, 0.677) |
| Senegal | 1990 to 1994 | 1 (1, 1) |
| Senegal | 1995 to 1999 | 0.968 (0.91, 1.03) |
| Senegal | 2000 to 2004 | 0.918 (0.862, 0.977) |
| Senegal | 2005 to 2009 | 0.867 (0.816, 0.922) |
| Senegal | 2010 to 2014 | 0.82 (0.774, 0.869) |
| Senegal | 2015 to 2019 | 0.773 (0.731, 0.817) |
| Serbia | 1990 to 1994 | 1 (1, 1) |
| Serbia | 1995 to 1999 | 0.997 (0.916, 1.086) |
| Serbia | 2000 to 2004 | 0.99 (0.905, 1.082) |
| Serbia | 2005 to 2009 | 0.977 (0.892, 1.069) |
| Serbia | 2010 to 2014 | 0.966 (0.884, 1.055) |
| Serbia | 2015 to 2019 | 0.962 (0.883, 1.048) |
| Seychelles | 1990 to 1994 | 1 (1, 1) |
| Seychelles | 1995 to 1999 | 1.02 (0.539, 1.933) |
| Seychelles | 2000 to 2004 | 0.975 (0.51, 1.864) |
| Seychelles | 2005 to 2009 | 0.941 (0.495, 1.79) |
| Seychelles | 2010 to 2014 | 0.938 (0.504, 1.747) |
| Seychelles | 2015 to 2019 | 0.875 (0.472, 1.621) |
| Sierra Leone | 1990 to 1994 | 1 (1, 1) |
| Sierra Leone | 1995 to 1999 | 1.084 (0.977, 1.204) |
| Sierra Leone | 2000 to 2004 | 1.093 (0.989, 1.209) |
| Sierra Leone | 2005 to 2009 | 1.096 (0.996, 1.206) |
| Sierra Leone | 2010 to 2014 | 1.114 (1.018, 1.22) |
| Sierra Leone | 2015 to 2019 | 1.1 (1.009, 1.199) |
| Singapore | 1990 to 1994 | 1 (1, 1) |
| Singapore | 1995 to 1999 | 0.988 (0.908, 1.074) |
| Singapore | 2000 to 2004 | 0.974 (0.894, 1.061) |
| Singapore | 2005 to 2009 | 0.959 (0.882, 1.043) |
| Singapore | 2010 to 2014 | 0.946 (0.873, 1.025) |
| Singapore | 2015 to 2019 | 0.942 (0.871, 1.019) |
| Slovakia | 1990 to 1994 | 1 (1, 1) |
| Slovakia | 1995 to 1999 | 0.99 (0.884, 1.108) |
| Slovakia | 2000 to 2004 | 0.978 (0.87, 1.099) |
| Slovakia | 2005 to 2009 | 0.972 (0.866, 1.092) |
| Slovakia | 2010 to 2014 | 0.966 (0.862, 1.081) |
| Slovakia | 2015 to 2019 | 0.959 (0.858, 1.072) |
| Slovenia | 1990 to 1994 | 1 (1, 1) |
| Slovenia | 1995 to 1999 | 0.993 (0.822, 1.2) |
| Slovenia | 2000 to 2004 | 0.978 (0.802, 1.193) |
| Slovenia | 2005 to 2009 | 0.964 (0.789, 1.178) |
| Slovenia | 2010 to 2014 | 0.958 (0.787, 1.167) |
| Slovenia | 2015 to 2019 | 0.963 (0.794, 1.168) |
| Solomon Islands | 1990 to 1994 | 1 (1, 1) |
| Solomon Islands | 1995 to 1999 | 0.985 (0.71, 1.367) |
| Solomon Islands | 2000 to 2004 | 0.966 (0.699, 1.335) |
| Solomon Islands | 2005 to 2009 | 0.965 (0.707, 1.318) |
| Solomon Islands | 2010 to 2014 | 0.925 (0.685, 1.249) |
| Solomon Islands | 2015 to 2019 | 0.876 (0.653, 1.177) |
| Somalia | 1990 to 1994 | 1 (1, 1) |
| Somalia | 1995 to 1999 | 0.988 (0.918, 1.062) |
| Somalia | 2000 to 2004 | 0.988 (0.92, 1.061) |
| Somalia | 2005 to 2009 | 0.979 (0.916, 1.047) |
| Somalia | 2010 to 2014 | 0.954 (0.895, 1.016) |
| Somalia | 2015 to 2019 | 0.909 (0.855, 0.967) |
| South Africa | 1990 to 1994 | 1 (1, 1) |
| South Africa | 1995 to 1999 | 1.002 (0.976, 1.029) |
| South Africa | 2000 to 2004 | 0.984 (0.957, 1.011) |
| South Africa | 2005 to 2009 | 0.941 (0.916, 0.967) |
| South Africa | 2010 to 2014 | 0.895 (0.872, 0.919) |
| South Africa | 2015 to 2019 | 0.854 (0.833, 0.876) |
| South Sudan | 1990 to 1994 | 1 (1, 1) |
| South Sudan | 1995 to 1999 | 0.984 (0.918, 1.055) |
| South Sudan | 2000 to 2004 | 0.942 (0.88, 1.01) |
| South Sudan | 2005 to 2009 | 0.934 (0.874, 0.998) |
| South Sudan | 2010 to 2014 | 0.939 (0.881, 1.001) |
| South Sudan | 2015 to 2019 | 0.897 (0.84, 0.959) |
| Spain | 1990 to 1994 | 1 (1, 1) |
| Spain | 1995 to 1999 | 0.987 (0.966, 1.008) |
| Spain | 2000 to 2004 | 0.992 (0.971, 1.013) |
| Spain | 2005 to 2009 | 0.999 (0.978, 1.021) |
| Spain | 2010 to 2014 | 0.988 (0.967, 1.009) |
| Spain | 2015 to 2019 | 0.98 (0.96, 1.001) |
| Sri Lanka | 1990 to 1994 | 1 (1, 1) |
| Sri Lanka | 1995 to 1999 | 0.982 (0.942, 1.024) |
| Sri Lanka | 2000 to 2004 | 0.967 (0.926, 1.009) |
| Sri Lanka | 2005 to 2009 | 0.942 (0.902, 0.983) |
| Sri Lanka | 2010 to 2014 | 0.903 (0.867, 0.942) |
| Sri Lanka | 2015 to 2019 | 0.864 (0.829, 0.901) |
| Sudan | 1990 to 1994 | 1 (1, 1) |
| Sudan | 1995 to 1999 | 0.958 (0.93, 0.987) |
| Sudan | 2000 to 2004 | 0.921 (0.894, 0.948) |
| Sudan | 2005 to 2009 | 0.885 (0.86, 0.91) |
| Sudan | 2010 to 2014 | 0.848 (0.825, 0.871) |
| Sudan | 2015 to 2019 | 0.812 (0.791, 0.834) |
| Suriname | 1990 to 1994 | 1 (1, 1) |
| Suriname | 1995 to 1999 | 1.003 (0.783, 1.284) |
| Suriname | 2000 to 2004 | 1.01 (0.789, 1.292) |
| Suriname | 2005 to 2009 | 0.976 (0.765, 1.245) |
| Suriname | 2010 to 2014 | 0.946 (0.746, 1.2) |
| Suriname | 2015 to 2019 | 0.923 (0.733, 1.162) |
| Sweden | 1990 to 1994 | 1 (1, 1) |
| Sweden | 1995 to 1999 | 1.009 (0.929, 1.097) |
| Sweden | 2000 to 2004 | 1.008 (0.925, 1.098) |
| Sweden | 2005 to 2009 | 1.012 (0.93, 1.102) |
| Sweden | 2010 to 2014 | 0.996 (0.917, 1.081) |
| Sweden | 2015 to 2019 | 0.989 (0.915, 1.07) |
| Switzerland | 1990 to 1994 | 1 (1, 1) |
| Switzerland | 1995 to 1999 | 0.991 (0.919, 1.068) |
| Switzerland | 2000 to 2004 | 0.983 (0.909, 1.063) |
| Switzerland | 2005 to 2009 | 0.977 (0.904, 1.057) |
| Switzerland | 2010 to 2014 | 0.974 (0.904, 1.05) |
| Switzerland | 2015 to 2019 | 0.971 (0.905, 1.043) |
| Syrian Arab Republic | 1990 to 1994 | 1 (1, 1) |
| Syrian Arab Republic | 1995 to 1999 | 0.964 (0.928, 1.001) |
| Syrian Arab Republic | 2000 to 2004 | 0.924 (0.89, 0.96) |
| Syrian Arab Republic | 2005 to 2009 | 0.892 (0.86, 0.925) |
| Syrian Arab Republic | 2010 to 2014 | 0.874 (0.843, 0.907) |
| Syrian Arab Republic | 2015 to 2019 | 0.861 (0.827, 0.897) |
| Taiwan (Province of China) | 1990 to 1994 | 1 (1, 1) |
| Taiwan (Province of China) | 1995 to 1999 | 0.99 (0.924, 1.062) |
| Taiwan (Province of China) | 2000 to 2004 | 0.974 (0.905, 1.048) |
| Taiwan (Province of China) | 2005 to 2009 | 0.955 (0.888, 1.028) |
| Taiwan (Province of China) | 2010 to 2014 | 0.942 (0.878, 1.012) |
| Taiwan (Province of China) | 2015 to 2019 | 0.926 (0.863, 0.993) |
| Tajikistan | 1990 to 1994 | 1 (1, 1) |
| Tajikistan | 1995 to 1999 | 0.993 (0.917, 1.076) |
| Tajikistan | 2000 to 2004 | 0.988 (0.912, 1.071) |
| Tajikistan | 2005 to 2009 | 0.975 (0.902, 1.053) |
| Tajikistan | 2010 to 2014 | 0.963 (0.895, 1.036) |
| Tajikistan | 2015 to 2019 | 0.939 (0.875, 1.007) |
| Thailand | 1990 to 1994 | 1 (1, 1) |
| Thailand | 1995 to 1999 | 0.975 (0.954, 0.997) |
| Thailand | 2000 to 2004 | 0.942 (0.921, 0.964) |
| Thailand | 2005 to 2009 | 0.919 (0.898, 0.94) |
| Thailand | 2010 to 2014 | 0.893 (0.874, 0.914) |
| Thailand | 2015 to 2019 | 0.859 (0.84, 0.878) |
| Timor-Leste | 1990 to 1994 | 1 (1, 1) |
| Timor-Leste | 1995 to 1999 | 0.972 (0.829, 1.138) |
| Timor-Leste | 2000 to 2004 | 0.934 (0.794, 1.098) |
| Timor-Leste | 2005 to 2009 | 0.876 (0.746, 1.028) |
| Timor-Leste | 2010 to 2014 | 0.806 (0.692, 0.939) |
| Timor-Leste | 2015 to 2019 | 0.766 (0.662, 0.888) |
| Togo | 1990 to 1994 | 1 (1, 1) |
| Togo | 1995 to 1999 | 1.048 (0.954, 1.15) |
| Togo | 2000 to 2004 | 1.037 (0.945, 1.137) |
| Togo | 2005 to 2009 | 1.01 (0.924, 1.104) |
| Togo | 2010 to 2014 | 0.979 (0.899, 1.066) |
| Togo | 2015 to 2019 | 0.923 (0.85, 1.002) |
| Tokelau | 1990 to 1994 | 1 (1, 1) |
| Tokelau | 1995 to 1999 | 0.971 (0.003, 328.797) |
| Tokelau | 2000 to 2004 | 0.938 (0.002, 431.223) |
| Tokelau | 2005 to 2009 | 0.902 (0.001, 560.944) |
| Tokelau | 2010 to 2014 | 0.866 (0.002, 405.395) |
| Tokelau | 2015 to 2019 | 0.829 (0.002, 341.399) |
| Tonga | 1990 to 1994 | 1 (1, 1) |
| Tonga | 1995 to 1999 | 0.888 (0.378, 2.088) |
| Tonga | 2000 to 2004 | 0.911 (0.391, 2.125) |
| Tonga | 2005 to 2009 | 0.921 (0.405, 2.094) |
| Tonga | 2010 to 2014 | 0.888 (0.393, 2.009) |
| Tonga | 2015 to 2019 | 0.798 (0.347, 1.837) |
| Trinidad and Tobago | 1990 to 1994 | 1 (1, 1) |
| Trinidad and Tobago | 1995 to 1999 | 1.011 (0.866, 1.181) |
| Trinidad and Tobago | 2000 to 2004 | 0.993 (0.846, 1.166) |
| Trinidad and Tobago | 2005 to 2009 | 0.971 (0.828, 1.14) |
| Trinidad and Tobago | 2010 to 2014 | 0.957 (0.818, 1.118) |
| Trinidad and Tobago | 2015 to 2019 | 0.941 (0.809, 1.096) |
| Tunisia | 1990 to 1994 | 1 (1, 1) |
| Tunisia | 1995 to 1999 | 0.957 (0.906, 1.01) |
| Tunisia | 2000 to 2004 | 0.941 (0.892, 0.994) |
| Tunisia | 2005 to 2009 | 0.949 (0.9, 1.001) |
| Tunisia | 2010 to 2014 | 0.913 (0.867, 0.962) |
| Tunisia | 2015 to 2019 | 0.883 (0.838, 0.929) |
| Turkey | 1990 to 1994 | 1 (1, 1) |
| Turkey | 1995 to 1999 | 1.002 (0.98, 1.024) |
| Turkey | 2000 to 2004 | 0.996 (0.974, 1.018) |
| Turkey | 2005 to 2009 | 0.948 (0.927, 0.969) |
| Turkey | 2010 to 2014 | 0.914 (0.895, 0.934) |
| Turkey | 2015 to 2019 | 0.88 (0.862, 0.898) |
| Turkmenistan | 1990 to 1994 | 1 (1, 1) |
| Turkmenistan | 1995 to 1999 | 0.983 (0.9, 1.075) |
| Turkmenistan | 2000 to 2004 | 0.965 (0.882, 1.057) |
| Turkmenistan | 2005 to 2009 | 0.954 (0.873, 1.042) |
| Turkmenistan | 2010 to 2014 | 0.928 (0.852, 1.01) |
| Turkmenistan | 2015 to 2019 | 0.898 (0.826, 0.976) |
| Tuvalu | 1990 to 1994 | 1 (1, 1) |
| Tuvalu | 1995 to 1999 | 0.945 (0.109, 8.17) |
| Tuvalu | 2000 to 2004 | 0.942 (0.101, 8.739) |
| Tuvalu | 2005 to 2009 | 0.903 (0.1, 8.171) |
| Tuvalu | 2010 to 2014 | 0.878 (0.109, 7.081) |
| Tuvalu | 2015 to 2019 | 0.836 (0.113, 6.177) |
| Uganda | 1990 to 1994 | 1 (1, 1) |
| Uganda | 1995 to 1999 | 0.989 (0.938, 1.043) |
| Uganda | 2000 to 2004 | 0.951 (0.903, 1.002) |
| Uganda | 2005 to 2009 | 0.905 (0.86, 0.952) |
| Uganda | 2010 to 2014 | 0.904 (0.862, 0.948) |
| Uganda | 2015 to 2019 | 0.898 (0.858, 0.94) |
| Ukraine | 1990 to 1994 | 1 (1, 1) |
| Ukraine | 1995 to 1999 | 0.999 (0.968, 1.031) |
| Ukraine | 2000 to 2004 | 0.995 (0.962, 1.028) |
| Ukraine | 2005 to 2009 | 0.979 (0.948, 1.012) |
| Ukraine | 2010 to 2014 | 0.971 (0.94, 1.003) |
| Ukraine | 2015 to 2019 | 0.966 (0.936, 0.997) |
| United Arab Emirates | 1990 to 1994 | 1 (1, 1) |
| United Arab Emirates | 1995 to 1999 | 0.961 (0.881, 1.048) |
| United Arab Emirates | 2000 to 2004 | 0.928 (0.853, 1.01) |
| United Arab Emirates | 2005 to 2009 | 0.895 (0.828, 0.969) |
| United Arab Emirates | 2010 to 2014 | 0.876 (0.813, 0.943) |
| United Arab Emirates | 2015 to 2019 | 0.861 (0.8, 0.927) |
| United Kingdom | 1990 to 1994 | 1 (1, 1) |
| United Kingdom | 1995 to 1999 | 0.991 (0.967, 1.016) |
| United Kingdom | 2000 to 2004 | 0.986 (0.962, 1.012) |
| United Kingdom | 2005 to 2009 | 0.985 (0.96, 1.01) |
| United Kingdom | 2010 to 2014 | 0.983 (0.959, 1.007) |
| United Kingdom | 2015 to 2019 | 0.981 (0.958, 1.004) |
| United Republic of Tanzania | 1990 to 1994 | 1 (1, 1) |
| United Republic of Tanzania | 1995 to 1999 | 0.966 (0.936, 0.998) |
| United Republic of Tanzania | 2000 to 2004 | 0.928 (0.898, 0.958) |
| United Republic of Tanzania | 2005 to 2009 | 0.904 (0.876, 0.933) |
| United Republic of Tanzania | 2010 to 2014 | 0.861 (0.836, 0.887) |
| United Republic of Tanzania | 2015 to 2019 | 0.811 (0.788, 0.834) |
| United States of America | 1990 to 1994 | 1 (1, 1) |
| United States of America | 1995 to 1999 | 1.021 (0.997, 1.045) |
| United States of America | 2000 to 2004 | 1.033 (1.008, 1.058) |
| United States of America | 2005 to 2009 | 1.032 (1.007, 1.056) |
| United States of America | 2010 to 2014 | 1.021 (0.997, 1.044) |
| United States of America | 2015 to 2019 | 0.998 (0.976, 1.02) |
| United States Virgin Islands | 1990 to 1994 | 1 (1, 1) |
| United States Virgin Islands | 1995 to 1999 | 0.941 (0.522, 1.697) |
| United States Virgin Islands | 2000 to 2004 | 0.942 (0.508, 1.749) |
| United States Virgin Islands | 2005 to 2009 | 0.992 (0.534, 1.842) |
| United States Virgin Islands | 2010 to 2014 | 0.903 (0.488, 1.671) |
| United States Virgin Islands | 2015 to 2019 | 0.837 (0.453, 1.546) |
| Uruguay | 1990 to 1994 | 1 (1, 1) |
| Uruguay | 1995 to 1999 | 0.996 (0.894, 1.108) |
| Uruguay | 2000 to 2004 | 0.987 (0.885, 1.1) |
| Uruguay | 2005 to 2009 | 0.984 (0.883, 1.096) |
| Uruguay | 2010 to 2014 | 0.977 (0.88, 1.085) |
| Uruguay | 2015 to 2019 | 0.965 (0.87, 1.07) |
| Uzbekistan | 1990 to 1994 | 1 (1, 1) |
| Uzbekistan | 1995 to 1999 | 0.989 (0.95, 1.03) |
| Uzbekistan | 2000 to 2004 | 0.975 (0.936, 1.015) |
| Uzbekistan | 2005 to 2009 | 0.961 (0.924, 0.999) |
| Uzbekistan | 2010 to 2014 | 0.935 (0.901, 0.971) |
| Uzbekistan | 2015 to 2019 | 0.904 (0.872, 0.937) |
| Vanuatu | 1990 to 1994 | 1 (1, 1) |
| Vanuatu | 1995 to 1999 | 1.002 (0.555, 1.808) |
| Vanuatu | 2000 to 2004 | 0.958 (0.53, 1.731) |
| Vanuatu | 2005 to 2009 | 0.945 (0.536, 1.667) |
| Vanuatu | 2010 to 2014 | 0.937 (0.548, 1.601) |
| Vanuatu | 2015 to 2019 | 0.869 (0.515, 1.467) |
| Venezuela (Bolivarian Republic of) | 1990 to 1994 | 1 (1, 1) |
| Venezuela (Bolivarian Republic of) | 1995 to 1999 | 0.98 (0.947, 1.013) |
| Venezuela (Bolivarian Republic of) | 2000 to 2004 | 0.971 (0.938, 1.004) |
| Venezuela (Bolivarian Republic of) | 2005 to 2009 | 0.959 (0.927, 0.991) |
| Venezuela (Bolivarian Republic of) | 2010 to 2014 | 0.935 (0.906, 0.965) |
| Venezuela (Bolivarian Republic of) | 2015 to 2019 | 0.932 (0.903, 0.962) |
| Viet Nam | 1990 to 1994 | 1 (1, 1) |
| Viet Nam | 1995 to 1999 | 0.999 (0.965, 1.034) |
| Viet Nam | 2000 to 2004 | 1.004 (0.97, 1.04) |
| Viet Nam | 2005 to 2009 | 0.975 (0.943, 1.009) |
| Viet Nam | 2010 to 2014 | 0.918 (0.888, 0.948) |
| Viet Nam | 2015 to 2019 | 0.84 (0.813, 0.867) |
| Yemen | 1990 to 1994 | 1 (1, 1) |
| Yemen | 1995 to 1999 | 0.998 (0.956, 1.041) |
| Yemen | 2000 to 2004 | 0.977 (0.938, 1.019) |
| Yemen | 2005 to 2009 | 0.945 (0.908, 0.983) |
| Yemen | 2010 to 2014 | 0.911 (0.878, 0.947) |
| Yemen | 2015 to 2019 | 0.897 (0.865, 0.931) |
| Zambia | 1990 to 1994 | 1 (1, 1) |
| Zambia | 1995 to 1999 | 0.98 (0.911, 1.055) |
| Zambia | 2000 to 2004 | 0.949 (0.881, 1.021) |
| Zambia | 2005 to 2009 | 0.909 (0.846, 0.976) |
| Zambia | 2010 to 2014 | 0.847 (0.792, 0.906) |
| Zambia | 2015 to 2019 | 0.776 (0.727, 0.827) |
| Zimbabwe | 1990 to 1994 | 1 (1, 1) |
| Zimbabwe | 1995 to 1999 | 0.986 (0.936, 1.04) |
| Zimbabwe | 2000 to 2004 | 0.984 (0.933, 1.038) |
| Zimbabwe | 2005 to 2009 | 0.999 (0.949, 1.052) |
| Zimbabwe | 2010 to 2014 | 0.997 (0.948, 1.047) |
| Zimbabwe | 2015 to 2019 | 0.946 (0.901, 0.992) |

**Abbreviations:** DALYs, Disability adjusted life years.

**Table S9 Cohort effects on blindness and vision loss DALYs in teenagers and young adults across countries**

| **Location** | **Cohort** | **DALYs rate ratio** |
| --- | --- | --- |
| Afghanistan | 1950 to 1959 | 1.089 (1.005, 1.181) |
| Afghanistan | 1955 to 1964 | 1.067 (1.01, 1.128) |
| Afghanistan | 1960 to 1969 | 1.04 (1.002, 1.078) |
| Afghanistan | 1965 to 1974 | 1.021 (0.993, 1.051) |
| Afghanistan | 1970 to 1979 | 1 (1, 1) |
| Afghanistan | 1975 to 1984 | 0.978 (0.951, 1.006) |
| Afghanistan | 1980 to 1989 | 0.958 (0.931, 0.986) |
| Afghanistan | 1985 to 1994 | 0.938 (0.91, 0.966) |
| Afghanistan | 1990 to 1999 | 0.918 (0.887, 0.949) |
| Afghanistan | 1995 to 2004 | 0.894 (0.853, 0.936) |
| Albania | 1950 to 1959 | 1.067 (0.851, 1.336) |
| Albania | 1955 to 1964 | 1.048 (0.884, 1.242) |
| Albania | 1960 to 1969 | 1.029 (0.882, 1.2) |
| Albania | 1965 to 1974 | 1.016 (0.879, 1.173) |
| Albania | 1970 to 1979 | 1 (1, 1) |
| Albania | 1975 to 1984 | 0.991 (0.861, 1.14) |
| Albania | 1980 to 1989 | 0.979 (0.839, 1.143) |
| Albania | 1985 to 1994 | 0.973 (0.821, 1.153) |
| Albania | 1990 to 1999 | 0.967 (0.789, 1.186) |
| Albania | 1995 to 2004 | 0.97 (0.716, 1.313) |
| Algeria | 1950 to 1959 | 1.167 (1.105, 1.232) |
| Algeria | 1955 to 1964 | 1.12 (1.079, 1.163) |
| Algeria | 1960 to 1969 | 1.076 (1.044, 1.109) |
| Algeria | 1965 to 1974 | 1.035 (1.008, 1.063) |
| Algeria | 1970 to 1979 | 1 (1, 1) |
| Algeria | 1975 to 1984 | 0.968 (0.946, 0.991) |
| Algeria | 1980 to 1989 | 0.939 (0.916, 0.962) |
| Algeria | 1985 to 1994 | 0.913 (0.889, 0.939) |
| Algeria | 1990 to 1999 | 0.891 (0.862, 0.922) |
| Algeria | 1995 to 2004 | 0.868 (0.826, 0.912) |
| American Samoa | 1950 to 1959 | 0.83 (0.151, 4.567) |
| American Samoa | 1955 to 1964 | 1.039 (0.317, 3.401) |
| American Samoa | 1960 to 1969 | 0.911 (0.328, 2.525) |
| American Samoa | 1965 to 1974 | 0.955 (0.382, 2.387) |
| American Samoa | 1970 to 1979 | 1 (1, 1) |
| American Samoa | 1975 to 1984 | 0.876 (0.356, 2.155) |
| American Samoa | 1980 to 1989 | 0.679 (0.225, 2.047) |
| American Samoa | 1985 to 1994 | 0.703 (0.207, 2.384) |
| American Samoa | 1990 to 1999 | 0.837 (0.24, 2.916) |
| American Samoa | 1995 to 2004 | 0.758 (0.127, 4.518) |
| Andorra | 1950 to 1959 | 1.174 (0.338, 4.076) |
| Andorra | 1955 to 1964 | 0.988 (0.4, 2.445) |
| Andorra | 1960 to 1969 | 0.952 (0.44, 2.058) |
| Andorra | 1965 to 1974 | 1.005 (0.503, 2.006) |
| Andorra | 1970 to 1979 | 1 (1, 1) |
| Andorra | 1975 to 1984 | 0.95 (0.481, 1.879) |
| Andorra | 1980 to 1989 | 1.026 (0.474, 2.222) |
| Andorra | 1985 to 1994 | 0.994 (0.394, 2.511) |
| Andorra | 1990 to 1999 | 0.915 (0.28, 2.987) |
| Andorra | 1995 to 2004 | 0.883 (0.163, 4.793) |
| Angola | 1950 to 1959 | 1.198 (1.096, 1.309) |
| Angola | 1955 to 1964 | 1.147 (1.074, 1.224) |
| Angola | 1960 to 1969 | 1.098 (1.038, 1.161) |
| Angola | 1965 to 1974 | 1.047 (0.995, 1.101) |
| Angola | 1970 to 1979 | 1 (1, 1) |
| Angola | 1975 to 1984 | 0.959 (0.917, 1.003) |
| Angola | 1980 to 1989 | 0.912 (0.868, 0.958) |
| Angola | 1985 to 1994 | 0.869 (0.821, 0.919) |
| Angola | 1990 to 1999 | 0.831 (0.776, 0.89) |
| Angola | 1995 to 2004 | 0.801 (0.729, 0.88) |
| Antigua and Barbuda | 1950 to 1959 | 1.02 (0.311, 3.344) |
| Antigua and Barbuda | 1955 to 1964 | 1.149 (0.508, 2.603) |
| Antigua and Barbuda | 1960 to 1969 | 1.149 (0.577, 2.286) |
| Antigua and Barbuda | 1965 to 1974 | 1.04 (0.548, 1.974) |
| Antigua and Barbuda | 1970 to 1979 | 1 (1, 1) |
| Antigua and Barbuda | 1975 to 1984 | 1.04 (0.567, 1.909) |
| Antigua and Barbuda | 1980 to 1989 | 1.02 (0.522, 1.994) |
| Antigua and Barbuda | 1985 to 1994 | 0.985 (0.455, 2.131) |
| Antigua and Barbuda | 1990 to 1999 | 0.961 (0.371, 2.486) |
| Antigua and Barbuda | 1995 to 2004 | 0.925 (0.229, 3.738) |
| Argentina | 1950 to 1959 | 1.069 (1.012, 1.129) |
| Argentina | 1955 to 1964 | 1.05 (1.009, 1.092) |
| Argentina | 1960 to 1969 | 1.031 (0.997, 1.066) |
| Argentina | 1965 to 1974 | 1.013 (0.984, 1.043) |
| Argentina | 1970 to 1979 | 1 (1, 1) |
| Argentina | 1975 to 1984 | 0.989 (0.963, 1.015) |
| Argentina | 1980 to 1989 | 0.978 (0.951, 1.005) |
| Argentina | 1985 to 1994 | 0.967 (0.938, 0.996) |
| Argentina | 1990 to 1999 | 0.959 (0.925, 0.994) |
| Argentina | 1995 to 2004 | 0.948 (0.902, 0.997) |
| Armenia | 1950 to 1959 | 1.091 (0.919, 1.296) |
| Armenia | 1955 to 1964 | 1.069 (0.939, 1.217) |
| Armenia | 1960 to 1969 | 1.048 (0.928, 1.183) |
| Armenia | 1965 to 1974 | 1.024 (0.91, 1.151) |
| Armenia | 1970 to 1979 | 1 (1, 1) |
| Armenia | 1975 to 1984 | 0.98 (0.881, 1.091) |
| Armenia | 1980 to 1989 | 0.966 (0.861, 1.084) |
| Armenia | 1985 to 1994 | 0.952 (0.835, 1.085) |
| Armenia | 1990 to 1999 | 0.937 (0.789, 1.113) |
| Armenia | 1995 to 2004 | 0.926 (0.712, 1.204) |
| Australia | 1950 to 1959 | 1.042 (0.956, 1.136) |
| Australia | 1955 to 1964 | 1.027 (0.964, 1.093) |
| Australia | 1960 to 1969 | 1.012 (0.959, 1.069) |
| Australia | 1965 to 1974 | 1.005 (0.957, 1.055) |
| Australia | 1970 to 1979 | 1 (1, 1) |
| Australia | 1975 to 1984 | 0.994 (0.95, 1.04) |
| Australia | 1980 to 1989 | 0.99 (0.944, 1.039) |
| Australia | 1985 to 1994 | 0.986 (0.935, 1.039) |
| Australia | 1990 to 1999 | 0.982 (0.922, 1.046) |
| Australia | 1995 to 2004 | 0.984 (0.899, 1.076) |
| Austria | 1950 to 1959 | 1.047 (0.921, 1.191) |
| Austria | 1955 to 1964 | 1.036 (0.946, 1.136) |
| Austria | 1960 to 1969 | 1.023 (0.947, 1.104) |
| Austria | 1965 to 1974 | 1.011 (0.941, 1.085) |
| Austria | 1970 to 1979 | 1 (1, 1) |
| Austria | 1975 to 1984 | 0.993 (0.926, 1.065) |
| Austria | 1980 to 1989 | 0.987 (0.915, 1.065) |
| Austria | 1985 to 1994 | 0.985 (0.905, 1.071) |
| Austria | 1990 to 1999 | 0.98 (0.884, 1.087) |
| Austria | 1995 to 2004 | 0.969 (0.831, 1.131) |
| Azerbaijan | 1950 to 1959 | 1.097 (0.983, 1.224) |
| Azerbaijan | 1955 to 1964 | 1.071 (0.991, 1.159) |
| Azerbaijan | 1960 to 1969 | 1.048 (0.977, 1.123) |
| Azerbaijan | 1965 to 1974 | 1.025 (0.96, 1.094) |
| Azerbaijan | 1970 to 1979 | 1 (1, 1) |
| Azerbaijan | 1975 to 1984 | 0.977 (0.919, 1.038) |
| Azerbaijan | 1980 to 1989 | 0.958 (0.898, 1.022) |
| Azerbaijan | 1985 to 1994 | 0.942 (0.877, 1.013) |
| Azerbaijan | 1990 to 1999 | 0.931 (0.85, 1.019) |
| Azerbaijan | 1995 to 2004 | 0.923 (0.806, 1.056) |
| Bahamas | 1950 to 1959 | 1.108 (0.635, 1.934) |
| Bahamas | 1955 to 1964 | 1.052 (0.702, 1.575) |
| Bahamas | 1960 to 1969 | 1.054 (0.748, 1.485) |
| Bahamas | 1965 to 1974 | 1.03 (0.753, 1.41) |
| Bahamas | 1970 to 1979 | 1 (1, 1) |
| Bahamas | 1975 to 1984 | 1.001 (0.741, 1.352) |
| Bahamas | 1980 to 1989 | 0.988 (0.708, 1.379) |
| Bahamas | 1985 to 1994 | 0.98 (0.672, 1.429) |
| Bahamas | 1990 to 1999 | 0.968 (0.612, 1.533) |
| Bahamas | 1995 to 2004 | 1.025 (0.529, 1.988) |
| Bahrain | 1950 to 1959 | 1.191 (0.89, 1.594) |
| Bahrain | 1955 to 1964 | 1.132 (0.925, 1.385) |
| Bahrain | 1960 to 1969 | 1.092 (0.917, 1.301) |
| Bahrain | 1965 to 1974 | 1.044 (0.893, 1.219) |
| Bahrain | 1970 to 1979 | 1 (1, 1) |
| Bahrain | 1975 to 1984 | 0.964 (0.848, 1.096) |
| Bahrain | 1980 to 1989 | 0.934 (0.808, 1.08) |
| Bahrain | 1985 to 1994 | 0.904 (0.757, 1.08) |
| Bahrain | 1990 to 1999 | 0.884 (0.702, 1.114) |
| Bahrain | 1995 to 2004 | 0.851 (0.617, 1.175) |
| Bangladesh | 1950 to 1959 | 1.134 (1.107, 1.162) |
| Bangladesh | 1955 to 1964 | 1.102 (1.082, 1.123) |
| Bangladesh | 1960 to 1969 | 1.072 (1.055, 1.09) |
| Bangladesh | 1965 to 1974 | 1.038 (1.023, 1.054) |
| Bangladesh | 1970 to 1979 | 1 (1, 1) |
| Bangladesh | 1975 to 1984 | 0.96 (0.947, 0.973) |
| Bangladesh | 1980 to 1989 | 0.915 (0.901, 0.929) |
| Bangladesh | 1985 to 1994 | 0.876 (0.861, 0.892) |
| Bangladesh | 1990 to 1999 | 0.846 (0.828, 0.864) |
| Bangladesh | 1995 to 2004 | 0.82 (0.795, 0.845) |
| Barbados | 1950 to 1959 | 1.081 (0.49, 2.384) |
| Barbados | 1955 to 1964 | 1.048 (0.574, 1.914) |
| Barbados | 1960 to 1969 | 1.019 (0.594, 1.747) |
| Barbados | 1965 to 1974 | 0.995 (0.604, 1.64) |
| Barbados | 1970 to 1979 | 1 (1, 1) |
| Barbados | 1975 to 1984 | 0.979 (0.603, 1.589) |
| Barbados | 1980 to 1989 | 0.942 (0.545, 1.628) |
| Barbados | 1985 to 1994 | 0.977 (0.524, 1.821) |
| Barbados | 1990 to 1999 | 1.012 (0.47, 2.178) |
| Barbados | 1995 to 2004 | 1.033 (0.348, 3.067) |
| Belarus | 1950 to 1959 | 1.059 (0.956, 1.174) |
| Belarus | 1955 to 1964 | 1.044 (0.964, 1.13) |
| Belarus | 1960 to 1969 | 1.027 (0.954, 1.107) |
| Belarus | 1965 to 1974 | 1.014 (0.944, 1.088) |
| Belarus | 1970 to 1979 | 1 (1, 1) |
| Belarus | 1975 to 1984 | 0.985 (0.922, 1.052) |
| Belarus | 1980 to 1989 | 0.972 (0.904, 1.045) |
| Belarus | 1985 to 1994 | 0.96 (0.88, 1.046) |
| Belarus | 1990 to 1999 | 0.953 (0.848, 1.071) |
| Belarus | 1995 to 2004 | 0.948 (0.796, 1.128) |
| Belgium | 1950 to 1959 | 1.052 (0.941, 1.176) |
| Belgium | 1955 to 1964 | 1.037 (0.956, 1.126) |
| Belgium | 1960 to 1969 | 1.023 (0.954, 1.097) |
| Belgium | 1965 to 1974 | 1.011 (0.948, 1.078) |
| Belgium | 1970 to 1979 | 1 (1, 1) |
| Belgium | 1975 to 1984 | 0.99 (0.93, 1.053) |
| Belgium | 1980 to 1989 | 0.982 (0.918, 1.05) |
| Belgium | 1985 to 1994 | 0.975 (0.905, 1.051) |
| Belgium | 1990 to 1999 | 0.969 (0.884, 1.062) |
| Belgium | 1995 to 2004 | 0.959 (0.839, 1.095) |
| Belize | 1950 to 1959 | 1.09 (0.549, 2.164) |
| Belize | 1955 to 1964 | 1.073 (0.667, 1.725) |
| Belize | 1960 to 1969 | 1.073 (0.723, 1.59) |
| Belize | 1965 to 1974 | 0.992 (0.698, 1.409) |
| Belize | 1970 to 1979 | 1 (1, 1) |
| Belize | 1975 to 1984 | 0.968 (0.71, 1.319) |
| Belize | 1980 to 1989 | 0.942 (0.675, 1.315) |
| Belize | 1985 to 1994 | 0.931 (0.644, 1.346) |
| Belize | 1990 to 1999 | 0.91 (0.588, 1.41) |
| Belize | 1995 to 2004 | 0.892 (0.488, 1.628) |
| Benin | 1950 to 1959 | 0.768 (0.662, 0.891) |
| Benin | 1955 to 1964 | 0.842 (0.761, 0.931) |
| Benin | 1960 to 1969 | 0.919 (0.847, 0.998) |
| Benin | 1965 to 1974 | 0.979 (0.912, 1.051) |
| Benin | 1970 to 1979 | 1 (1, 1) |
| Benin | 1975 to 1984 | 0.979 (0.92, 1.042) |
| Benin | 1980 to 1989 | 0.954 (0.891, 1.022) |
| Benin | 1985 to 1994 | 0.929 (0.859, 1.005) |
| Benin | 1990 to 1999 | 0.897 (0.817, 0.986) |
| Benin | 1995 to 2004 | 0.864 (0.758, 0.984) |
| Bermuda | 1950 to 1959 | 1.077 (0.325, 3.565) |
| Bermuda | 1955 to 1964 | 1.259 (0.522, 3.036) |
| Bermuda | 1960 to 1969 | 1.077 (0.486, 2.387) |
| Bermuda | 1965 to 1974 | 1.099 (0.517, 2.334) |
| Bermuda | 1970 to 1979 | 1 (1, 1) |
| Bermuda | 1975 to 1984 | 1.05 (0.494, 2.229) |
| Bermuda | 1980 to 1989 | 0.973 (0.41, 2.304) |
| Bermuda | 1985 to 1994 | 0.981 (0.36, 2.673) |
| Bermuda | 1990 to 1999 | 1.127 (0.336, 3.777) |
| Bermuda | 1995 to 2004 | 0.573 (0.063, 5.193) |
| Bhutan | 1950 to 1959 | 1.17 (0.794, 1.723) |
| Bhutan | 1955 to 1964 | 1.135 (0.841, 1.53) |
| Bhutan | 1960 to 1969 | 1.082 (0.825, 1.419) |
| Bhutan | 1965 to 1974 | 1.039 (0.811, 1.331) |
| Bhutan | 1970 to 1979 | 1 (1, 1) |
| Bhutan | 1975 to 1984 | 0.965 (0.773, 1.204) |
| Bhutan | 1980 to 1989 | 0.937 (0.731, 1.201) |
| Bhutan | 1985 to 1994 | 0.903 (0.677, 1.204) |
| Bhutan | 1990 to 1999 | 0.866 (0.606, 1.238) |
| Bhutan | 1995 to 2004 | 0.835 (0.491, 1.42) |
| Bolivia (Plurinational State of) | 1950 to 1959 | 1.114 (1.013, 1.224) |
| Bolivia (Plurinational State of) | 1955 to 1964 | 1.081 (1.009, 1.158) |
| Bolivia (Plurinational State of) | 1960 to 1969 | 1.051 (0.992, 1.113) |
| Bolivia (Plurinational State of) | 1965 to 1974 | 1.025 (0.974, 1.079) |
| Bolivia (Plurinational State of) | 1970 to 1979 | 1 (1, 1) |
| Bolivia (Plurinational State of) | 1975 to 1984 | 0.976 (0.933, 1.021) |
| Bolivia (Plurinational State of) | 1980 to 1989 | 0.953 (0.908, 1) |
| Bolivia (Plurinational State of) | 1985 to 1994 | 0.932 (0.883, 0.983) |
| Bolivia (Plurinational State of) | 1990 to 1999 | 0.913 (0.857, 0.973) |
| Bolivia (Plurinational State of) | 1995 to 2004 | 0.895 (0.817, 0.98) |
| Bosnia and Herzegovina | 1950 to 1959 | 1.094 (0.904, 1.323) |
| Bosnia and Herzegovina | 1955 to 1964 | 1.064 (0.915, 1.237) |
| Bosnia and Herzegovina | 1960 to 1969 | 1.036 (0.903, 1.188) |
| Bosnia and Herzegovina | 1965 to 1974 | 1.014 (0.89, 1.156) |
| Bosnia and Herzegovina | 1970 to 1979 | 1 (1, 1) |
| Bosnia and Herzegovina | 1975 to 1984 | 0.986 (0.872, 1.115) |
| Bosnia and Herzegovina | 1980 to 1989 | 0.977 (0.85, 1.123) |
| Bosnia and Herzegovina | 1985 to 1994 | 0.972 (0.823, 1.148) |
| Bosnia and Herzegovina | 1990 to 1999 | 0.961 (0.781, 1.182) |
| Bosnia and Herzegovina | 1995 to 2004 | 0.965 (0.712, 1.309) |
| Botswana | 1950 to 1959 | 1.17 (0.954, 1.437) |
| Botswana | 1955 to 1964 | 1.13 (0.97, 1.317) |
| Botswana | 1960 to 1969 | 1.089 (0.95, 1.248) |
| Botswana | 1965 to 1974 | 1.043 (0.922, 1.179) |
| Botswana | 1970 to 1979 | 1 (1, 1) |
| Botswana | 1975 to 1984 | 0.961 (0.862, 1.072) |
| Botswana | 1980 to 1989 | 0.915 (0.809, 1.036) |
| Botswana | 1985 to 1994 | 0.876 (0.753, 1.018) |
| Botswana | 1990 to 1999 | 0.837 (0.69, 1.016) |
| Botswana | 1995 to 2004 | 0.81 (0.61, 1.076) |
| Brazil | 1950 to 1959 | 1.077 (1.058, 1.096) |
| Brazil | 1955 to 1964 | 1.044 (1.031, 1.058) |
| Brazil | 1960 to 1969 | 1.023 (1.013, 1.034) |
| Brazil | 1965 to 1974 | 1.011 (1.002, 1.021) |
| Brazil | 1970 to 1979 | 1 (1, 1) |
| Brazil | 1975 to 1984 | 0.991 (0.983, 1) |
| Brazil | 1980 to 1989 | 0.982 (0.973, 0.991) |
| Brazil | 1985 to 1994 | 0.969 (0.959, 0.979) |
| Brazil | 1990 to 1999 | 0.956 (0.944, 0.969) |
| Brazil | 1995 to 2004 | 0.944 (0.927, 0.961) |
| Brunei Darussalam | 1950 to 1959 | 1.033 (0.556, 1.921) |
| Brunei Darussalam | 1955 to 1964 | 1.011 (0.662, 1.543) |
| Brunei Darussalam | 1960 to 1969 | 1.014 (0.715, 1.438) |
| Brunei Darussalam | 1965 to 1974 | 1.015 (0.744, 1.384) |
| Brunei Darussalam | 1970 to 1979 | 1 (1, 1) |
| Brunei Darussalam | 1975 to 1984 | 1 (0.755, 1.325) |
| Brunei Darussalam | 1980 to 1989 | 0.99 (0.736, 1.331) |
| Brunei Darussalam | 1985 to 1994 | 0.973 (0.7, 1.353) |
| Brunei Darussalam | 1990 to 1999 | 0.946 (0.634, 1.411) |
| Brunei Darussalam | 1995 to 2004 | 0.958 (0.548, 1.675) |
| Bulgaria | 1950 to 1959 | 1.047 (0.908, 1.208) |
| Bulgaria | 1955 to 1964 | 1.034 (0.922, 1.158) |
| Bulgaria | 1960 to 1969 | 1.02 (0.919, 1.131) |
| Bulgaria | 1965 to 1974 | 1.01 (0.92, 1.11) |
| Bulgaria | 1970 to 1979 | 1 (1, 1) |
| Bulgaria | 1975 to 1984 | 0.991 (0.906, 1.084) |
| Bulgaria | 1980 to 1989 | 0.982 (0.887, 1.088) |
| Bulgaria | 1985 to 1994 | 0.977 (0.864, 1.105) |
| Bulgaria | 1990 to 1999 | 0.974 (0.825, 1.151) |
| Bulgaria | 1995 to 2004 | 0.966 (0.757, 1.234) |
| Burkina Faso | 1950 to 1959 | 0.793 (0.713, 0.882) |
| Burkina Faso | 1955 to 1964 | 0.916 (0.851, 0.986) |
| Burkina Faso | 1960 to 1969 | 0.985 (0.926, 1.048) |
| Burkina Faso | 1965 to 1974 | 1.009 (0.955, 1.065) |
| Burkina Faso | 1970 to 1979 | 1 (1, 1) |
| Burkina Faso | 1975 to 1984 | 0.977 (0.932, 1.024) |
| Burkina Faso | 1980 to 1989 | 0.96 (0.911, 1.011) |
| Burkina Faso | 1985 to 1994 | 0.933 (0.879, 0.99) |
| Burkina Faso | 1990 to 1999 | 0.904 (0.842, 0.971) |
| Burkina Faso | 1995 to 2004 | 0.867 (0.787, 0.956) |
| Burundi | 1950 to 1959 | 1.086 (0.927, 1.273) |
| Burundi | 1955 to 1964 | 1.076 (0.954, 1.214) |
| Burundi | 1960 to 1969 | 1.055 (0.945, 1.178) |
| Burundi | 1965 to 1974 | 1.031 (0.93, 1.142) |
| Burundi | 1970 to 1979 | 1 (1, 1) |
| Burundi | 1975 to 1984 | 0.968 (0.885, 1.059) |
| Burundi | 1980 to 1989 | 0.93 (0.843, 1.026) |
| Burundi | 1985 to 1994 | 0.891 (0.796, 0.997) |
| Burundi | 1990 to 1999 | 0.852 (0.743, 0.977) |
| Burundi | 1995 to 2004 | 0.83 (0.688, 1.002) |
| Cabo Verde | 1950 to 1959 | 1.207 (0.681, 2.137) |
| Cabo Verde | 1955 to 1964 | 1.15 (0.77, 1.716) |
| Cabo Verde | 1960 to 1969 | 1.099 (0.778, 1.553) |
| Cabo Verde | 1965 to 1974 | 1.041 (0.755, 1.435) |
| Cabo Verde | 1970 to 1979 | 1 (1, 1) |
| Cabo Verde | 1975 to 1984 | 0.953 (0.711, 1.278) |
| Cabo Verde | 1980 to 1989 | 0.925 (0.673, 1.27) |
| Cabo Verde | 1985 to 1994 | 0.882 (0.61, 1.275) |
| Cabo Verde | 1990 to 1999 | 0.873 (0.551, 1.383) |
| Cabo Verde | 1995 to 2004 | 0.853 (0.433, 1.678) |
| Cambodia | 1950 to 1959 | 1.423 (1.323, 1.531) |
| Cambodia | 1955 to 1964 | 1.305 (1.238, 1.377) |
| Cambodia | 1960 to 1969 | 1.191 (1.138, 1.246) |
| Cambodia | 1965 to 1974 | 1.09 (1.045, 1.136) |
| Cambodia | 1970 to 1979 | 1 (1, 1) |
| Cambodia | 1975 to 1984 | 0.917 (0.883, 0.952) |
| Cambodia | 1980 to 1989 | 0.834 (0.801, 0.868) |
| Cambodia | 1985 to 1994 | 0.756 (0.723, 0.79) |
| Cambodia | 1990 to 1999 | 0.685 (0.65, 0.723) |
| Cambodia | 1995 to 2004 | 0.635 (0.59, 0.684) |
| Cameroon | 1950 to 1959 | 0.928 (0.837, 1.028) |
| Cameroon | 1955 to 1964 | 0.949 (0.883, 1.02) |
| Cameroon | 1960 to 1969 | 0.971 (0.915, 1.032) |
| Cameroon | 1965 to 1974 | 0.991 (0.941, 1.045) |
| Cameroon | 1970 to 1979 | 1 (1, 1) |
| Cameroon | 1975 to 1984 | 0.981 (0.938, 1.026) |
| Cameroon | 1980 to 1989 | 0.953 (0.908, 1.001) |
| Cameroon | 1985 to 1994 | 0.899 (0.851, 0.95) |
| Cameroon | 1990 to 1999 | 0.834 (0.78, 0.891) |
| Cameroon | 1995 to 2004 | 0.788 (0.719, 0.863) |
| Canada | 1950 to 1959 | 1.027 (0.953, 1.107) |
| Canada | 1955 to 1964 | 1.017 (0.963, 1.073) |
| Canada | 1960 to 1969 | 1.01 (0.963, 1.058) |
| Canada | 1965 to 1974 | 1.005 (0.962, 1.05) |
| Canada | 1970 to 1979 | 1 (1, 1) |
| Canada | 1975 to 1984 | 0.994 (0.954, 1.036) |
| Canada | 1980 to 1989 | 0.99 (0.948, 1.035) |
| Canada | 1985 to 1994 | 0.988 (0.941, 1.037) |
| Canada | 1990 to 1999 | 0.985 (0.929, 1.044) |
| Canada | 1995 to 2004 | 0.978 (0.898, 1.064) |
| Central African Republic | 1950 to 1959 | 0.724 (0.557, 0.939) |
| Central African Republic | 1955 to 1964 | 0.74 (0.617, 0.889) |
| Central African Republic | 1960 to 1969 | 0.785 (0.676, 0.913) |
| Central African Republic | 1965 to 1974 | 0.879 (0.774, 0.999) |
| Central African Republic | 1970 to 1979 | 1 (1, 1) |
| Central African Republic | 1975 to 1984 | 1.101 (0.988, 1.226) |
| Central African Republic | 1980 to 1989 | 1.204 (1.074, 1.35) |
| Central African Republic | 1985 to 1994 | 1.229 (1.084, 1.394) |
| Central African Republic | 1990 to 1999 | 1.188 (1.024, 1.377) |
| Central African Republic | 1995 to 2004 | 1.148 (0.939, 1.404) |
| Chad | 1950 to 1959 | 0.947 (0.836, 1.072) |
| Chad | 1955 to 1964 | 0.939 (0.858, 1.028) |
| Chad | 1960 to 1969 | 0.95 (0.88, 1.024) |
| Chad | 1965 to 1974 | 0.98 (0.918, 1.047) |
| Chad | 1970 to 1979 | 1 (1, 1) |
| Chad | 1975 to 1984 | 0.996 (0.941, 1.055) |
| Chad | 1980 to 1989 | 0.989 (0.93, 1.052) |
| Chad | 1985 to 1994 | 0.956 (0.893, 1.025) |
| Chad | 1990 to 1999 | 0.905 (0.834, 0.982) |
| Chad | 1995 to 2004 | 0.864 (0.774, 0.964) |
| Chile | 1950 to 1959 | 1.112 (1.021, 1.21) |
| Chile | 1955 to 1964 | 1.075 (1.012, 1.141) |
| Chile | 1960 to 1969 | 1.045 (0.994, 1.098) |
| Chile | 1965 to 1974 | 1.021 (0.976, 1.068) |
| Chile | 1970 to 1979 | 1 (1, 1) |
| Chile | 1975 to 1984 | 0.981 (0.94, 1.023) |
| Chile | 1980 to 1989 | 0.964 (0.922, 1.008) |
| Chile | 1985 to 1994 | 0.95 (0.905, 0.997) |
| Chile | 1990 to 1999 | 0.939 (0.886, 0.996) |
| Chile | 1995 to 2004 | 0.928 (0.853, 1.01) |
| China | 1950 to 1959 | 1.187 (1.132, 1.245) |
| China | 1955 to 1964 | 1.166 (1.123, 1.211) |
| China | 1960 to 1969 | 1.126 (1.091, 1.162) |
| China | 1965 to 1974 | 1.058 (1.028, 1.09) |
| China | 1970 to 1979 | 1 (1, 1) |
| China | 1975 to 1984 | 0.969 (0.94, 0.998) |
| China | 1980 to 1989 | 0.938 (0.908, 0.969) |
| China | 1985 to 1994 | 0.932 (0.899, 0.966) |
| China | 1990 to 1999 | 0.944 (0.901, 0.99) |
| China | 1995 to 2004 | 0.979 (0.915, 1.047) |
| Colombia | 1950 to 1959 | 1.124 (1.078, 1.172) |
| Colombia | 1955 to 1964 | 1.086 (1.054, 1.12) |
| Colombia | 1960 to 1969 | 1.054 (1.028, 1.082) |
| Colombia | 1965 to 1974 | 1.027 (1.003, 1.051) |
| Colombia | 1970 to 1979 | 1 (1, 1) |
| Colombia | 1975 to 1984 | 0.971 (0.95, 0.993) |
| Colombia | 1980 to 1989 | 0.947 (0.924, 0.969) |
| Colombia | 1985 to 1994 | 0.923 (0.898, 0.948) |
| Colombia | 1990 to 1999 | 0.902 (0.873, 0.931) |
| Colombia | 1995 to 2004 | 0.88 (0.839, 0.923) |
| Comoros | 1950 to 1959 | 1.217 (0.782, 1.894) |
| Comoros | 1955 to 1964 | 1.163 (0.834, 1.622) |
| Comoros | 1960 to 1969 | 1.103 (0.827, 1.47) |
| Comoros | 1965 to 1974 | 1.041 (0.803, 1.349) |
| Comoros | 1970 to 1979 | 1 (1, 1) |
| Comoros | 1975 to 1984 | 0.946 (0.743, 1.206) |
| Comoros | 1980 to 1989 | 0.888 (0.673, 1.17) |
| Comoros | 1985 to 1994 | 0.852 (0.617, 1.177) |
| Comoros | 1990 to 1999 | 0.824 (0.552, 1.228) |
| Comoros | 1995 to 2004 | 0.807 (0.455, 1.432) |
| Congo | 1950 to 1959 | 1.187 (0.971, 1.45) |
| Congo | 1955 to 1964 | 1.135 (0.981, 1.314) |
| Congo | 1960 to 1969 | 1.091 (0.964, 1.235) |
| Congo | 1965 to 1974 | 1.042 (0.934, 1.163) |
| Congo | 1970 to 1979 | 1 (1, 1) |
| Congo | 1975 to 1984 | 0.96 (0.87, 1.059) |
| Congo | 1980 to 1989 | 0.92 (0.824, 1.027) |
| Congo | 1985 to 1994 | 0.889 (0.781, 1.011) |
| Congo | 1990 to 1999 | 0.861 (0.734, 1.009) |
| Congo | 1995 to 2004 | 0.848 (0.679, 1.058) |
| Cook Islands | 1950 to 1959 | 1.144 (0.098, 13.364) |
| Cook Islands | 1955 to 1964 | 0.959 (0.161, 5.717) |
| Cook Islands | 1960 to 1969 | 0.977 (0.216, 4.421) |
| Cook Islands | 1965 to 1974 | 0.96 (0.249, 3.692) |
| Cook Islands | 1970 to 1979 | 1 (1, 1) |
| Cook Islands | 1975 to 1984 | 1.111 (0.319, 3.872) |
| Cook Islands | 1980 to 1989 | 1.155 (0.303, 4.407) |
| Cook Islands | 1985 to 1994 | 0.987 (0.199, 4.889) |
| Cook Islands | 1990 to 1999 | 0.912 (0.124, 6.72) |
| Cook Islands | 1995 to 2004 | 0.659 (0.026, 16.887) |
| Costa Rica | 1950 to 1959 | 1.066 (0.924, 1.231) |
| Costa Rica | 1955 to 1964 | 1.044 (0.944, 1.156) |
| Costa Rica | 1960 to 1969 | 1.023 (0.938, 1.115) |
| Costa Rica | 1965 to 1974 | 1.012 (0.935, 1.096) |
| Costa Rica | 1970 to 1979 | 1 (1, 1) |
| Costa Rica | 1975 to 1984 | 0.988 (0.919, 1.062) |
| Costa Rica | 1980 to 1989 | 0.979 (0.906, 1.058) |
| Costa Rica | 1985 to 1994 | 0.969 (0.887, 1.058) |
| Costa Rica | 1990 to 1999 | 0.961 (0.861, 1.071) |
| Costa Rica | 1995 to 2004 | 0.951 (0.81, 1.118) |
| Croatia | 1950 to 1959 | 1.047 (0.87, 1.261) |
| Croatia | 1955 to 1964 | 1.033 (0.89, 1.2) |
| Croatia | 1960 to 1969 | 1.019 (0.888, 1.169) |
| Croatia | 1965 to 1974 | 1.012 (0.889, 1.152) |
| Croatia | 1970 to 1979 | 1 (1, 1) |
| Croatia | 1975 to 1984 | 0.991 (0.877, 1.12) |
| Croatia | 1980 to 1989 | 0.984 (0.856, 1.13) |
| Croatia | 1985 to 1994 | 0.973 (0.825, 1.148) |
| Croatia | 1990 to 1999 | 0.971 (0.792, 1.19) |
| Croatia | 1995 to 2004 | 0.959 (0.712, 1.291) |
| Cuba | 1950 to 1959 | 1.057 (0.976, 1.144) |
| Cuba | 1955 to 1964 | 1.038 (0.98, 1.099) |
| Cuba | 1960 to 1969 | 1.026 (0.979, 1.075) |
| Cuba | 1965 to 1974 | 1.012 (0.969, 1.057) |
| Cuba | 1970 to 1979 | 1 (1, 1) |
| Cuba | 1975 to 1984 | 0.99 (0.945, 1.037) |
| Cuba | 1980 to 1989 | 0.982 (0.935, 1.032) |
| Cuba | 1985 to 1994 | 0.975 (0.922, 1.031) |
| Cuba | 1990 to 1999 | 0.968 (0.9, 1.04) |
| Cuba | 1995 to 2004 | 0.963 (0.866, 1.071) |
| Cyprus | 1950 to 1959 | 1.072 (0.742, 1.549) |
| Cyprus | 1955 to 1964 | 1.052 (0.812, 1.364) |
| Cyprus | 1960 to 1969 | 1.03 (0.826, 1.286) |
| Cyprus | 1965 to 1974 | 1.013 (0.83, 1.236) |
| Cyprus | 1970 to 1979 | 1 (1, 1) |
| Cyprus | 1975 to 1984 | 0.984 (0.826, 1.174) |
| Cyprus | 1980 to 1989 | 0.981 (0.815, 1.181) |
| Cyprus | 1985 to 1994 | 0.965 (0.783, 1.19) |
| Cyprus | 1990 to 1999 | 0.946 (0.725, 1.233) |
| Cyprus | 1995 to 2004 | 0.935 (0.627, 1.394) |
| Czechia | 1950 to 1959 | 1.052 (0.93, 1.19) |
| Czechia | 1955 to 1964 | 1.034 (0.935, 1.145) |
| Czechia | 1960 to 1969 | 1.021 (0.934, 1.117) |
| Czechia | 1965 to 1974 | 1.01 (0.932, 1.095) |
| Czechia | 1970 to 1979 | 1 (1, 1) |
| Czechia | 1975 to 1984 | 0.989 (0.918, 1.065) |
| Czechia | 1980 to 1989 | 0.979 (0.898, 1.069) |
| Czechia | 1985 to 1994 | 0.972 (0.876, 1.078) |
| Czechia | 1990 to 1999 | 0.966 (0.841, 1.108) |
| Czechia | 1995 to 2004 | 0.957 (0.78, 1.174) |
| Côte d'Ivoire | 1950 to 1959 | 0.761 (0.687, 0.842) |
| Côte d'Ivoire | 1955 to 1964 | 0.894 (0.835, 0.956) |
| Côte d'Ivoire | 1960 to 1969 | 0.966 (0.914, 1.02) |
| Côte d'Ivoire | 1965 to 1974 | 0.999 (0.952, 1.049) |
| Côte d'Ivoire | 1970 to 1979 | 1 (1, 1) |
| Côte d'Ivoire | 1975 to 1984 | 0.978 (0.937, 1.02) |
| Côte d'Ivoire | 1980 to 1989 | 0.964 (0.919, 1.01) |
| Côte d'Ivoire | 1985 to 1994 | 0.94 (0.889, 0.994) |
| Côte d'Ivoire | 1990 to 1999 | 0.91 (0.849, 0.976) |
| Côte d'Ivoire | 1995 to 2004 | 0.878 (0.796, 0.969) |
| Democratic People's Republic of Korea | 1950 to 1959 | 1.096 (0.993, 1.21) |
| Democratic People's Republic of Korea | 1955 to 1964 | 1.068 (0.991, 1.151) |
| Democratic People's Republic of Korea | 1960 to 1969 | 1.047 (0.981, 1.118) |
| Democratic People's Republic of Korea | 1965 to 1974 | 1.027 (0.967, 1.09) |
| Democratic People's Republic of Korea | 1970 to 1979 | 1 (1, 1) |
| Democratic People's Republic of Korea | 1975 to 1984 | 0.973 (0.917, 1.032) |
| Democratic People's Republic of Korea | 1980 to 1989 | 0.946 (0.885, 1.011) |
| Democratic People's Republic of Korea | 1985 to 1994 | 0.929 (0.862, 1) |
| Democratic People's Republic of Korea | 1990 to 1999 | 0.917 (0.84, 1.002) |
| Democratic People's Republic of Korea | 1995 to 2004 | 0.902 (0.794, 1.025) |
| Democratic Republic of the Congo | 1950 to 1959 | 0.967 (0.905, 1.033) |
| Democratic Republic of the Congo | 1955 to 1964 | 0.981 (0.935, 1.028) |
| Democratic Republic of the Congo | 1960 to 1969 | 0.994 (0.955, 1.035) |
| Democratic Republic of the Congo | 1965 to 1974 | 0.999 (0.964, 1.035) |
| Democratic Republic of the Congo | 1970 to 1979 | 1 (1, 1) |
| Democratic Republic of the Congo | 1975 to 1984 | 0.994 (0.964, 1.026) |
| Democratic Republic of the Congo | 1980 to 1989 | 0.979 (0.947, 1.013) |
| Democratic Republic of the Congo | 1985 to 1994 | 0.952 (0.917, 0.989) |
| Democratic Republic of the Congo | 1990 to 1999 | 0.924 (0.884, 0.966) |
| Democratic Republic of the Congo | 1995 to 2004 | 0.905 (0.851, 0.962) |
| Denmark | 1950 to 1959 | 1.044 (0.891, 1.223) |
| Denmark | 1955 to 1964 | 1.032 (0.918, 1.159) |
| Denmark | 1960 to 1969 | 1.021 (0.927, 1.125) |
| Denmark | 1965 to 1974 | 1.012 (0.926, 1.105) |
| Denmark | 1970 to 1979 | 1 (1, 1) |
| Denmark | 1975 to 1984 | 0.992 (0.91, 1.082) |
| Denmark | 1980 to 1989 | 0.987 (0.897, 1.086) |
| Denmark | 1985 to 1994 | 0.985 (0.888, 1.093) |
| Denmark | 1990 to 1999 | 0.977 (0.864, 1.105) |
| Denmark | 1995 to 2004 | 0.971 (0.811, 1.164) |
| Djibouti | 1950 to 1959 | 1.214 (0.822, 1.792) |
| Djibouti | 1955 to 1964 | 1.17 (0.874, 1.567) |
| Djibouti | 1960 to 1969 | 1.118 (0.873, 1.434) |
| Djibouti | 1965 to 1974 | 1.056 (0.846, 1.317) |
| Djibouti | 1970 to 1979 | 1 (1, 1) |
| Djibouti | 1975 to 1984 | 0.945 (0.777, 1.149) |
| Djibouti | 1980 to 1989 | 0.885 (0.709, 1.105) |
| Djibouti | 1985 to 1994 | 0.831 (0.638, 1.083) |
| Djibouti | 1990 to 1999 | 0.781 (0.557, 1.094) |
| Djibouti | 1995 to 2004 | 0.74 (0.455, 1.203) |
| Dominica | 1950 to 1959 | 1.085 (0.313, 3.767) |
| Dominica | 1955 to 1964 | 1.186 (0.486, 2.893) |
| Dominica | 1960 to 1969 | 1.064 (0.491, 2.302) |
| Dominica | 1965 to 1974 | 1.102 (0.545, 2.227) |
| Dominica | 1970 to 1979 | 1 (1, 1) |
| Dominica | 1975 to 1984 | 1.036 (0.514, 2.088) |
| Dominica | 1980 to 1989 | 1.046 (0.494, 2.214) |
| Dominica | 1985 to 1994 | 0.997 (0.427, 2.331) |
| Dominica | 1990 to 1999 | 1.063 (0.388, 2.916) |
| Dominica | 1995 to 2004 | 1.139 (0.275, 4.713) |
| Dominican Republic | 1950 to 1959 | 1.144 (1.039, 1.26) |
| Dominican Republic | 1955 to 1964 | 1.099 (1.026, 1.178) |
| Dominican Republic | 1960 to 1969 | 1.063 (1.004, 1.127) |
| Dominican Republic | 1965 to 1974 | 1.03 (0.978, 1.084) |
| Dominican Republic | 1970 to 1979 | 1 (1, 1) |
| Dominican Republic | 1975 to 1984 | 0.972 (0.926, 1.02) |
| Dominican Republic | 1980 to 1989 | 0.946 (0.898, 0.997) |
| Dominican Republic | 1985 to 1994 | 0.923 (0.871, 0.978) |
| Dominican Republic | 1990 to 1999 | 0.905 (0.844, 0.971) |
| Dominican Republic | 1995 to 2004 | 0.887 (0.802, 0.981) |
| Ecuador | 1950 to 1959 | 1.063 (0.973, 1.162) |
| Ecuador | 1955 to 1964 | 1.043 (0.978, 1.111) |
| Ecuador | 1960 to 1969 | 1.027 (0.974, 1.084) |
| Ecuador | 1965 to 1974 | 1.013 (0.965, 1.062) |
| Ecuador | 1970 to 1979 | 1 (1, 1) |
| Ecuador | 1975 to 1984 | 0.987 (0.945, 1.03) |
| Ecuador | 1980 to 1989 | 0.975 (0.931, 1.022) |
| Ecuador | 1985 to 1994 | 0.963 (0.915, 1.015) |
| Ecuador | 1990 to 1999 | 0.954 (0.897, 1.014) |
| Ecuador | 1995 to 2004 | 0.945 (0.867, 1.03) |
| Egypt | 1950 to 1959 | 1.173 (1.133, 1.215) |
| Egypt | 1955 to 1964 | 1.125 (1.098, 1.153) |
| Egypt | 1960 to 1969 | 1.076 (1.054, 1.098) |
| Egypt | 1965 to 1974 | 1.034 (1.016, 1.053) |
| Egypt | 1970 to 1979 | 1 (1, 1) |
| Egypt | 1975 to 1984 | 0.97 (0.955, 0.986) |
| Egypt | 1980 to 1989 | 0.943 (0.928, 0.959) |
| Egypt | 1985 to 1994 | 0.919 (0.903, 0.936) |
| Egypt | 1990 to 1999 | 0.899 (0.88, 0.918) |
| Egypt | 1995 to 2004 | 0.878 (0.853, 0.905) |
| El Salvador | 1950 to 1959 | 1.128 (1.014, 1.255) |
| El Salvador | 1955 to 1964 | 1.085 (1.004, 1.173) |
| El Salvador | 1960 to 1969 | 1.049 (0.983, 1.119) |
| El Salvador | 1965 to 1974 | 1.021 (0.964, 1.082) |
| El Salvador | 1970 to 1979 | 1 (1, 1) |
| El Salvador | 1975 to 1984 | 0.977 (0.926, 1.03) |
| El Salvador | 1980 to 1989 | 0.957 (0.903, 1.015) |
| El Salvador | 1985 to 1994 | 0.936 (0.878, 0.998) |
| El Salvador | 1990 to 1999 | 0.914 (0.848, 0.986) |
| El Salvador | 1995 to 2004 | 0.893 (0.799, 0.998) |
| Equatorial Guinea | 1950 to 1959 | 1.791 (1.262, 2.543) |
| Equatorial Guinea | 1955 to 1964 | 1.552 (1.194, 2.018) |
| Equatorial Guinea | 1960 to 1969 | 1.364 (1.091, 1.704) |
| Equatorial Guinea | 1965 to 1974 | 1.155 (0.943, 1.415) |
| Equatorial Guinea | 1970 to 1979 | 1 (1, 1) |
| Equatorial Guinea | 1975 to 1984 | 0.88 (0.732, 1.056) |
| Equatorial Guinea | 1980 to 1989 | 0.76 (0.625, 0.925) |
| Equatorial Guinea | 1985 to 1994 | 0.671 (0.538, 0.837) |
| Equatorial Guinea | 1990 to 1999 | 0.605 (0.464, 0.79) |
| Equatorial Guinea | 1995 to 2004 | 0.554 (0.385, 0.798) |
| Eritrea | 1950 to 1959 | 1.158 (0.998, 1.344) |
| Eritrea | 1955 to 1964 | 1.139 (1.02, 1.271) |
| Eritrea | 1960 to 1969 | 1.104 (1.003, 1.214) |
| Eritrea | 1965 to 1974 | 1.054 (0.968, 1.148) |
| Eritrea | 1970 to 1979 | 1 (1, 1) |
| Eritrea | 1975 to 1984 | 0.954 (0.884, 1.031) |
| Eritrea | 1980 to 1989 | 0.901 (0.827, 0.982) |
| Eritrea | 1985 to 1994 | 0.849 (0.769, 0.937) |
| Eritrea | 1990 to 1999 | 0.805 (0.714, 0.908) |
| Eritrea | 1995 to 2004 | 0.77 (0.651, 0.912) |
| Estonia | 1950 to 1959 | 1.071 (0.772, 1.486) |
| Estonia | 1955 to 1964 | 1.042 (0.802, 1.355) |
| Estonia | 1960 to 1969 | 1.025 (0.803, 1.308) |
| Estonia | 1965 to 1974 | 1.01 (0.804, 1.269) |
| Estonia | 1970 to 1979 | 1 (1, 1) |
| Estonia | 1975 to 1984 | 0.999 (0.805, 1.241) |
| Estonia | 1980 to 1989 | 0.991 (0.778, 1.262) |
| Estonia | 1985 to 1994 | 0.98 (0.739, 1.3) |
| Estonia | 1990 to 1999 | 0.971 (0.662, 1.425) |
| Estonia | 1995 to 2004 | 0.949 (0.532, 1.693) |
| Eswatini | 1950 to 1959 | 1.132 (0.852, 1.504) |
| Eswatini | 1955 to 1964 | 1.104 (0.891, 1.367) |
| Eswatini | 1960 to 1969 | 1.07 (0.886, 1.292) |
| Eswatini | 1965 to 1974 | 1.029 (0.867, 1.222) |
| Eswatini | 1970 to 1979 | 1 (1, 1) |
| Eswatini | 1975 to 1984 | 0.98 (0.839, 1.145) |
| Eswatini | 1980 to 1989 | 0.947 (0.797, 1.125) |
| Eswatini | 1985 to 1994 | 0.917 (0.75, 1.12) |
| Eswatini | 1990 to 1999 | 0.878 (0.684, 1.127) |
| Eswatini | 1995 to 2004 | 0.859 (0.598, 1.232) |
| Ethiopia | 1950 to 1959 | 1.1 (1.061, 1.141) |
| Ethiopia | 1955 to 1964 | 1.077 (1.048, 1.106) |
| Ethiopia | 1960 to 1969 | 1.053 (1.028, 1.077) |
| Ethiopia | 1965 to 1974 | 1.02 (0.999, 1.042) |
| Ethiopia | 1970 to 1979 | 1 (1, 1) |
| Ethiopia | 1975 to 1984 | 1.009 (0.991, 1.028) |
| Ethiopia | 1980 to 1989 | 1.012 (0.992, 1.033) |
| Ethiopia | 1985 to 1994 | 1.035 (1.011, 1.059) |
| Ethiopia | 1990 to 1999 | 1.084 (1.054, 1.114) |
| Ethiopia | 1995 to 2004 | 1.143 (1.1, 1.187) |
| Fiji | 1950 to 1959 | 1.029 (0.718, 1.473) |
| Fiji | 1955 to 1964 | 1.051 (0.802, 1.377) |
| Fiji | 1960 to 1969 | 1.06 (0.836, 1.344) |
| Fiji | 1965 to 1974 | 1.026 (0.823, 1.279) |
| Fiji | 1970 to 1979 | 1 (1, 1) |
| Fiji | 1975 to 1984 | 0.976 (0.798, 1.194) |
| Fiji | 1980 to 1989 | 0.942 (0.752, 1.18) |
| Fiji | 1985 to 1994 | 0.923 (0.712, 1.197) |
| Fiji | 1990 to 1999 | 0.881 (0.64, 1.214) |
| Fiji | 1995 to 2004 | 0.881 (0.558, 1.392) |
| Finland | 1950 to 1959 | 1.05 (0.897, 1.229) |
| Finland | 1955 to 1964 | 1.039 (0.923, 1.169) |
| Finland | 1960 to 1969 | 1.022 (0.924, 1.132) |
| Finland | 1965 to 1974 | 1.009 (0.917, 1.109) |
| Finland | 1970 to 1979 | 1 (1, 1) |
| Finland | 1975 to 1984 | 0.991 (0.908, 1.083) |
| Finland | 1980 to 1989 | 0.981 (0.892, 1.08) |
| Finland | 1985 to 1994 | 0.974 (0.875, 1.083) |
| Finland | 1990 to 1999 | 0.965 (0.847, 1.099) |
| Finland | 1995 to 2004 | 0.96 (0.793, 1.161) |
| France | 1950 to 1959 | 1.039 (0.99, 1.091) |
| France | 1955 to 1964 | 1.027 (0.991, 1.065) |
| France | 1960 to 1969 | 1.018 (0.988, 1.05) |
| France | 1965 to 1974 | 1.009 (0.982, 1.037) |
| France | 1970 to 1979 | 1 (1, 1) |
| France | 1975 to 1984 | 0.992 (0.966, 1.018) |
| France | 1980 to 1989 | 0.985 (0.957, 1.013) |
| France | 1985 to 1994 | 0.981 (0.95, 1.012) |
| France | 1990 to 1999 | 0.977 (0.94, 1.016) |
| France | 1995 to 2004 | 0.97 (0.919, 1.024) |
| Gabon | 1950 to 1959 | 1.191 (0.858, 1.655) |
| Gabon | 1955 to 1964 | 1.148 (0.9, 1.465) |
| Gabon | 1960 to 1969 | 1.105 (0.895, 1.364) |
| Gabon | 1965 to 1974 | 1.053 (0.87, 1.275) |
| Gabon | 1970 to 1979 | 1 (1, 1) |
| Gabon | 1975 to 1984 | 0.96 (0.805, 1.144) |
| Gabon | 1980 to 1989 | 0.919 (0.756, 1.117) |
| Gabon | 1985 to 1994 | 0.883 (0.706, 1.103) |
| Gabon | 1990 to 1999 | 0.866 (0.66, 1.135) |
| Gabon | 1995 to 2004 | 0.847 (0.576, 1.247) |
| Gambia | 1950 to 1959 | 1.197 (0.91, 1.573) |
| Gambia | 1955 to 1964 | 1.157 (0.949, 1.41) |
| Gambia | 1960 to 1969 | 1.115 (0.941, 1.32) |
| Gambia | 1965 to 1974 | 1.059 (0.909, 1.234) |
| Gambia | 1970 to 1979 | 1 (1, 1) |
| Gambia | 1975 to 1984 | 0.939 (0.817, 1.079) |
| Gambia | 1980 to 1989 | 0.873 (0.749, 1.017) |
| Gambia | 1985 to 1994 | 0.821 (0.69, 0.976) |
| Gambia | 1990 to 1999 | 0.764 (0.62, 0.942) |
| Gambia | 1995 to 2004 | 0.714 (0.534, 0.956) |
| Georgia | 1950 to 1959 | 1.441 (1.245, 1.668) |
| Georgia | 1955 to 1964 | 1.355 (1.211, 1.517) |
| Georgia | 1960 to 1969 | 1.205 (1.087, 1.335) |
| Georgia | 1965 to 1974 | 1.049 (0.951, 1.158) |
| Georgia | 1970 to 1979 | 1 (1, 1) |
| Georgia | 1975 to 1984 | 0.913 (0.831, 1.004) |
| Georgia | 1980 to 1989 | 0.859 (0.773, 0.953) |
| Georgia | 1985 to 1994 | 0.733 (0.65, 0.828) |
| Georgia | 1990 to 1999 | 0.577 (0.493, 0.675) |
| Georgia | 1995 to 2004 | 0.461 (0.364, 0.583) |
| Germany | 1950 to 1959 | 1.043 (1.001, 1.087) |
| Germany | 1955 to 1964 | 1.03 (1, 1.061) |
| Germany | 1960 to 1969 | 1.02 (0.995, 1.046) |
| Germany | 1965 to 1974 | 1.01 (0.986, 1.035) |
| Germany | 1970 to 1979 | 1 (1, 1) |
| Germany | 1975 to 1984 | 0.992 (0.969, 1.016) |
| Germany | 1980 to 1989 | 0.988 (0.963, 1.013) |
| Germany | 1985 to 1994 | 0.986 (0.959, 1.014) |
| Germany | 1990 to 1999 | 0.982 (0.948, 1.017) |
| Germany | 1995 to 2004 | 0.974 (0.925, 1.025) |
| Ghana | 1950 to 1959 | 0.885 (0.816, 0.959) |
| Ghana | 1955 to 1964 | 0.942 (0.89, 0.997) |
| Ghana | 1960 to 1969 | 0.983 (0.937, 1.03) |
| Ghana | 1965 to 1974 | 1.001 (0.96, 1.044) |
| Ghana | 1970 to 1979 | 1 (1, 1) |
| Ghana | 1975 to 1984 | 0.974 (0.939, 1.011) |
| Ghana | 1980 to 1989 | 0.959 (0.921, 0.999) |
| Ghana | 1985 to 1994 | 0.947 (0.905, 0.992) |
| Ghana | 1990 to 1999 | 0.933 (0.883, 0.987) |
| Ghana | 1995 to 2004 | 0.924 (0.854, 0.999) |
| Greece | 1950 to 1959 | 1.073 (0.957, 1.204) |
| Greece | 1955 to 1964 | 1.053 (0.968, 1.145) |
| Greece | 1960 to 1969 | 1.034 (0.963, 1.109) |
| Greece | 1965 to 1974 | 1.015 (0.953, 1.082) |
| Greece | 1970 to 1979 | 1 (1, 1) |
| Greece | 1975 to 1984 | 0.989 (0.932, 1.05) |
| Greece | 1980 to 1989 | 0.975 (0.911, 1.044) |
| Greece | 1985 to 1994 | 0.965 (0.891, 1.046) |
| Greece | 1990 to 1999 | 0.954 (0.862, 1.056) |
| Greece | 1995 to 2004 | 0.939 (0.808, 1.09) |
| Greenland | 1950 to 1959 | 0.718 (0.128, 4.032) |
| Greenland | 1955 to 1964 | 0.887 (0.289, 2.721) |
| Greenland | 1960 to 1969 | 0.789 (0.304, 2.047) |
| Greenland | 1965 to 1974 | 0.68 (0.263, 1.758) |
| Greenland | 1970 to 1979 | 1 (1, 1) |
| Greenland | 1975 to 1984 | 0.927 (0.384, 2.239) |
| Greenland | 1980 to 1989 | 0.83 (0.323, 2.138) |
| Greenland | 1985 to 1994 | 0.744 (0.261, 2.122) |
| Greenland | 1990 to 1999 | 0.784 (0.225, 2.74) |
| Greenland | 1995 to 2004 | 0.913 (0.159, 5.236) |
| Grenada | 1950 to 1959 | 0.994 (0.34, 2.905) |
| Grenada | 1955 to 1964 | 1.067 (0.502, 2.269) |
| Grenada | 1960 to 1969 | 0.996 (0.517, 1.917) |
| Grenada | 1965 to 1974 | 1.062 (0.606, 1.86) |
| Grenada | 1970 to 1979 | 1 (1, 1) |
| Grenada | 1975 to 1984 | 0.953 (0.565, 1.607) |
| Grenada | 1980 to 1989 | 0.945 (0.537, 1.662) |
| Grenada | 1985 to 1994 | 0.986 (0.527, 1.844) |
| Grenada | 1990 to 1999 | 0.919 (0.422, 2.003) |
| Grenada | 1995 to 2004 | 0.839 (0.258, 2.728) |
| Guam | 1950 to 1959 | 1.116 (0.441, 2.819) |
| Guam | 1955 to 1964 | 1.114 (0.548, 2.264) |
| Guam | 1960 to 1969 | 1.06 (0.562, 1.998) |
| Guam | 1965 to 1974 | 1.072 (0.596, 1.926) |
| Guam | 1970 to 1979 | 1 (1, 1) |
| Guam | 1975 to 1984 | 1.083 (0.609, 1.926) |
| Guam | 1980 to 1989 | 1.06 (0.559, 2.012) |
| Guam | 1985 to 1994 | 0.967 (0.468, 1.998) |
| Guam | 1990 to 1999 | 0.993 (0.43, 2.294) |
| Guam | 1995 to 2004 | 0.926 (0.272, 3.15) |
| Guatemala | 1950 to 1959 | 1.063 (0.981, 1.151) |
| Guatemala | 1955 to 1964 | 1.041 (0.983, 1.102) |
| Guatemala | 1960 to 1969 | 1.026 (0.979, 1.077) |
| Guatemala | 1965 to 1974 | 1.014 (0.973, 1.057) |
| Guatemala | 1970 to 1979 | 1 (1, 1) |
| Guatemala | 1975 to 1984 | 0.979 (0.945, 1.014) |
| Guatemala | 1980 to 1989 | 0.96 (0.924, 0.996) |
| Guatemala | 1985 to 1994 | 0.936 (0.899, 0.976) |
| Guatemala | 1990 to 1999 | 0.913 (0.87, 0.958) |
| Guatemala | 1995 to 2004 | 0.892 (0.834, 0.955) |
| Guinea | 1950 to 1959 | 0.918 (0.818, 1.03) |
| Guinea | 1955 to 1964 | 0.963 (0.886, 1.048) |
| Guinea | 1960 to 1969 | 0.999 (0.93, 1.074) |
| Guinea | 1965 to 1974 | 1.01 (0.947, 1.078) |
| Guinea | 1970 to 1979 | 1 (1, 1) |
| Guinea | 1975 to 1984 | 0.967 (0.912, 1.025) |
| Guinea | 1980 to 1989 | 0.934 (0.877, 0.995) |
| Guinea | 1985 to 1994 | 0.898 (0.837, 0.964) |
| Guinea | 1990 to 1999 | 0.859 (0.788, 0.936) |
| Guinea | 1995 to 2004 | 0.817 (0.725, 0.92) |
| Guinea-Bissau | 1950 to 1959 | 1.028 (0.772, 1.369) |
| Guinea-Bissau | 1955 to 1964 | 1.045 (0.849, 1.288) |
| Guinea-Bissau | 1960 to 1969 | 1.044 (0.873, 1.248) |
| Guinea-Bissau | 1965 to 1974 | 1.029 (0.878, 1.207) |
| Guinea-Bissau | 1970 to 1979 | 1 (1, 1) |
| Guinea-Bissau | 1975 to 1984 | 0.961 (0.834, 1.108) |
| Guinea-Bissau | 1980 to 1989 | 0.925 (0.793, 1.079) |
| Guinea-Bissau | 1985 to 1994 | 0.877 (0.736, 1.045) |
| Guinea-Bissau | 1990 to 1999 | 0.835 (0.673, 1.036) |
| Guinea-Bissau | 1995 to 2004 | 0.786 (0.579, 1.068) |
| Guyana | 1950 to 1959 | 1.096 (0.777, 1.547) |
| Guyana | 1955 to 1964 | 1.053 (0.816, 1.359) |
| Guyana | 1960 to 1969 | 1.041 (0.838, 1.295) |
| Guyana | 1965 to 1974 | 1.021 (0.837, 1.244) |
| Guyana | 1970 to 1979 | 1 (1, 1) |
| Guyana | 1975 to 1984 | 0.995 (0.822, 1.205) |
| Guyana | 1980 to 1989 | 0.973 (0.783, 1.207) |
| Guyana | 1985 to 1994 | 0.964 (0.76, 1.222) |
| Guyana | 1990 to 1999 | 0.954 (0.727, 1.253) |
| Guyana | 1995 to 2004 | 0.934 (0.624, 1.398) |
| Haiti | 1950 to 1959 | 1.1 (0.992, 1.22) |
| Haiti | 1955 to 1964 | 1.068 (0.991, 1.152) |
| Haiti | 1960 to 1969 | 1.045 (0.98, 1.115) |
| Haiti | 1965 to 1974 | 1.02 (0.963, 1.081) |
| Haiti | 1970 to 1979 | 1 (1, 1) |
| Haiti | 1975 to 1984 | 0.981 (0.933, 1.031) |
| Haiti | 1980 to 1989 | 0.962 (0.912, 1.015) |
| Haiti | 1985 to 1994 | 0.943 (0.887, 1.001) |
| Haiti | 1990 to 1999 | 0.924 (0.858, 0.994) |
| Haiti | 1995 to 2004 | 0.905 (0.817, 1.003) |
| Honduras | 1950 to 1959 | 1.101 (0.976, 1.241) |
| Honduras | 1955 to 1964 | 1.07 (0.983, 1.166) |
| Honduras | 1960 to 1969 | 1.045 (0.972, 1.122) |
| Honduras | 1965 to 1974 | 1.021 (0.959, 1.087) |
| Honduras | 1970 to 1979 | 1 (1, 1) |
| Honduras | 1975 to 1984 | 0.982 (0.93, 1.037) |
| Honduras | 1980 to 1989 | 0.966 (0.912, 1.023) |
| Honduras | 1985 to 1994 | 0.952 (0.894, 1.013) |
| Honduras | 1990 to 1999 | 0.933 (0.867, 1.005) |
| Honduras | 1995 to 2004 | 0.918 (0.828, 1.018) |
| Hungary | 1950 to 1959 | 1.061 (0.942, 1.194) |
| Hungary | 1955 to 1964 | 1.038 (0.94, 1.146) |
| Hungary | 1960 to 1969 | 1.021 (0.932, 1.118) |
| Hungary | 1965 to 1974 | 1.01 (0.931, 1.095) |
| Hungary | 1970 to 1979 | 1 (1, 1) |
| Hungary | 1975 to 1984 | 0.99 (0.918, 1.068) |
| Hungary | 1980 to 1989 | 0.983 (0.899, 1.075) |
| Hungary | 1985 to 1994 | 0.976 (0.878, 1.084) |
| Hungary | 1990 to 1999 | 0.97 (0.85, 1.107) |
| Hungary | 1995 to 2004 | 0.968 (0.794, 1.18) |
| Iceland | 1950 to 1959 | 1.094 (0.564, 2.124) |
| Iceland | 1955 to 1964 | 1.023 (0.627, 1.668) |
| Iceland | 1960 to 1969 | 1.023 (0.675, 1.55) |
| Iceland | 1965 to 1974 | 1.016 (0.694, 1.486) |
| Iceland | 1970 to 1979 | 1 (1, 1) |
| Iceland | 1975 to 1984 | 0.978 (0.688, 1.39) |
| Iceland | 1980 to 1989 | 0.949 (0.647, 1.393) |
| Iceland | 1985 to 1994 | 0.943 (0.619, 1.438) |
| Iceland | 1990 to 1999 | 0.917 (0.549, 1.531) |
| Iceland | 1995 to 2004 | 0.967 (0.462, 2.023) |
| India | 1950 to 1959 | 1.274 (1.257, 1.291) |
| India | 1955 to 1964 | 1.207 (1.194, 1.219) |
| India | 1960 to 1969 | 1.14 (1.13, 1.151) |
| India | 1965 to 1974 | 1.072 (1.064, 1.081) |
| India | 1970 to 1979 | 1 (1, 1) |
| India | 1975 to 1984 | 0.931 (0.924, 0.938) |
| India | 1980 to 1989 | 0.871 (0.863, 0.879) |
| India | 1985 to 1994 | 0.813 (0.805, 0.822) |
| India | 1990 to 1999 | 0.768 (0.758, 0.778) |
| India | 1995 to 2004 | 0.733 (0.72, 0.746) |
| Indonesia | 1950 to 1959 | 1.208 (1.19, 1.227) |
| Indonesia | 1955 to 1964 | 1.157 (1.144, 1.171) |
| Indonesia | 1960 to 1969 | 1.106 (1.095, 1.117) |
| Indonesia | 1965 to 1974 | 1.051 (1.042, 1.061) |
| Indonesia | 1970 to 1979 | 1 (1, 1) |
| Indonesia | 1975 to 1984 | 0.956 (0.948, 0.964) |
| Indonesia | 1980 to 1989 | 0.909 (0.9, 0.917) |
| Indonesia | 1985 to 1994 | 0.866 (0.857, 0.876) |
| Indonesia | 1990 to 1999 | 0.835 (0.824, 0.846) |
| Indonesia | 1995 to 2004 | 0.81 (0.794, 0.826) |
| Iran (Islamic Republic of) | 1950 to 1959 | 1.139 (1.103, 1.177) |
| Iran (Islamic Republic of) | 1955 to 1964 | 1.085 (1.061, 1.11) |
| Iran (Islamic Republic of) | 1960 to 1969 | 1.043 (1.023, 1.062) |
| Iran (Islamic Republic of) | 1965 to 1974 | 1.018 (1.002, 1.035) |
| Iran (Islamic Republic of) | 1970 to 1979 | 1 (1, 1) |
| Iran (Islamic Republic of) | 1975 to 1984 | 0.979 (0.966, 0.993) |
| Iran (Islamic Republic of) | 1980 to 1989 | 0.962 (0.949, 0.976) |
| Iran (Islamic Republic of) | 1985 to 1994 | 0.948 (0.933, 0.964) |
| Iran (Islamic Republic of) | 1990 to 1999 | 0.937 (0.917, 0.957) |
| Iran (Islamic Republic of) | 1995 to 2004 | 0.926 (0.898, 0.955) |
| Iraq | 1950 to 1959 | 1.171 (1.097, 1.25) |
| Iraq | 1955 to 1964 | 1.125 (1.076, 1.176) |
| Iraq | 1960 to 1969 | 1.078 (1.04, 1.117) |
| Iraq | 1965 to 1974 | 1.036 (1.006, 1.067) |
| Iraq | 1970 to 1979 | 1 (1, 1) |
| Iraq | 1975 to 1984 | 0.964 (0.938, 0.99) |
| Iraq | 1980 to 1989 | 0.933 (0.908, 0.96) |
| Iraq | 1985 to 1994 | 0.908 (0.881, 0.935) |
| Iraq | 1990 to 1999 | 0.884 (0.854, 0.915) |
| Iraq | 1995 to 2004 | 0.862 (0.822, 0.904) |
| Ireland | 1950 to 1959 | 1.067 (0.892, 1.277) |
| Ireland | 1955 to 1964 | 1.047 (0.919, 1.193) |
| Ireland | 1960 to 1969 | 1.031 (0.924, 1.15) |
| Ireland | 1965 to 1974 | 1.015 (0.923, 1.117) |
| Ireland | 1970 to 1979 | 1 (1, 1) |
| Ireland | 1975 to 1984 | 0.987 (0.906, 1.074) |
| Ireland | 1980 to 1989 | 0.973 (0.885, 1.069) |
| Ireland | 1985 to 1994 | 0.967 (0.866, 1.08) |
| Ireland | 1990 to 1999 | 0.96 (0.839, 1.098) |
| Ireland | 1995 to 2004 | 0.946 (0.785, 1.14) |
| Israel | 1950 to 1959 | 1.055 (0.909, 1.224) |
| Israel | 1955 to 1964 | 1.036 (0.93, 1.154) |
| Israel | 1960 to 1969 | 1.022 (0.934, 1.118) |
| Israel | 1965 to 1974 | 1.011 (0.935, 1.092) |
| Israel | 1970 to 1979 | 1 (1, 1) |
| Israel | 1975 to 1984 | 0.992 (0.926, 1.062) |
| Israel | 1980 to 1989 | 0.984 (0.915, 1.059) |
| Israel | 1985 to 1994 | 0.979 (0.902, 1.063) |
| Israel | 1990 to 1999 | 0.973 (0.883, 1.072) |
| Israel | 1995 to 2004 | 0.965 (0.844, 1.102) |
| Italy | 1950 to 1959 | 1.09 (1.05, 1.131) |
| Italy | 1955 to 1964 | 1.059 (1.03, 1.088) |
| Italy | 1960 to 1969 | 1.033 (1.01, 1.057) |
| Italy | 1965 to 1974 | 1.016 (0.995, 1.037) |
| Italy | 1970 to 1979 | 1 (1, 1) |
| Italy | 1975 to 1984 | 0.984 (0.965, 1.005) |
| Italy | 1980 to 1989 | 0.969 (0.947, 0.992) |
| Italy | 1985 to 1994 | 0.957 (0.932, 0.982) |
| Italy | 1990 to 1999 | 0.943 (0.913, 0.975) |
| Italy | 1995 to 2004 | 0.927 (0.883, 0.973) |
| Jamaica | 1950 to 1959 | 1.123 (0.915, 1.378) |
| Jamaica | 1955 to 1964 | 1.078 (0.931, 1.248) |
| Jamaica | 1960 to 1969 | 1.048 (0.926, 1.186) |
| Jamaica | 1965 to 1974 | 1.02 (0.912, 1.141) |
| Jamaica | 1970 to 1979 | 1 (1, 1) |
| Jamaica | 1975 to 1984 | 0.979 (0.88, 1.09) |
| Jamaica | 1980 to 1989 | 0.965 (0.86, 1.082) |
| Jamaica | 1985 to 1994 | 0.944 (0.831, 1.073) |
| Jamaica | 1990 to 1999 | 0.931 (0.799, 1.086) |
| Jamaica | 1995 to 2004 | 0.922 (0.736, 1.155) |
| Japan | 1950 to 1959 | 1.069 (1.032, 1.108) |
| Japan | 1955 to 1964 | 1.048 (1.021, 1.077) |
| Japan | 1960 to 1969 | 1.027 (1.004, 1.051) |
| Japan | 1965 to 1974 | 1.011 (0.992, 1.031) |
| Japan | 1970 to 1979 | 1 (1, 1) |
| Japan | 1975 to 1984 | 0.99 (0.971, 1.009) |
| Japan | 1980 to 1989 | 0.98 (0.959, 1.001) |
| Japan | 1985 to 1994 | 0.97 (0.946, 0.995) |
| Japan | 1990 to 1999 | 0.961 (0.932, 0.991) |
| Japan | 1995 to 2004 | 0.954 (0.913, 0.997) |
| Jordan | 1950 to 1959 | 1.166 (0.985, 1.38) |
| Jordan | 1955 to 1964 | 1.117 (0.996, 1.251) |
| Jordan | 1960 to 1969 | 1.075 (0.984, 1.175) |
| Jordan | 1965 to 1974 | 1.036 (0.961, 1.116) |
| Jordan | 1970 to 1979 | 1 (1, 1) |
| Jordan | 1975 to 1984 | 0.971 (0.91, 1.036) |
| Jordan | 1980 to 1989 | 0.944 (0.881, 1.01) |
| Jordan | 1985 to 1994 | 0.919 (0.854, 0.99) |
| Jordan | 1990 to 1999 | 0.9 (0.826, 0.98) |
| Jordan | 1995 to 2004 | 0.881 (0.786, 0.987) |
| Kazakhstan | 1950 to 1959 | 1.088 (1.008, 1.174) |
| Kazakhstan | 1955 to 1964 | 1.066 (1.006, 1.129) |
| Kazakhstan | 1960 to 1969 | 1.042 (0.988, 1.098) |
| Kazakhstan | 1965 to 1974 | 1.021 (0.971, 1.073) |
| Kazakhstan | 1970 to 1979 | 1 (1, 1) |
| Kazakhstan | 1975 to 1984 | 0.982 (0.938, 1.028) |
| Kazakhstan | 1980 to 1989 | 0.966 (0.92, 1.015) |
| Kazakhstan | 1985 to 1994 | 0.954 (0.902, 1.008) |
| Kazakhstan | 1990 to 1999 | 0.944 (0.88, 1.013) |
| Kazakhstan | 1995 to 2004 | 0.94 (0.848, 1.043) |
| Kenya | 1950 to 1959 | 1.312 (1.241, 1.388) |
| Kenya | 1955 to 1964 | 1.237 (1.187, 1.288) |
| Kenya | 1960 to 1969 | 1.155 (1.114, 1.196) |
| Kenya | 1965 to 1974 | 1.072 (1.038, 1.106) |
| Kenya | 1970 to 1979 | 1 (1, 1) |
| Kenya | 1975 to 1984 | 0.947 (0.92, 0.974) |
| Kenya | 1980 to 1989 | 0.89 (0.862, 0.918) |
| Kenya | 1985 to 1994 | 0.842 (0.812, 0.872) |
| Kenya | 1990 to 1999 | 0.804 (0.77, 0.84) |
| Kenya | 1995 to 2004 | 0.772 (0.727, 0.82) |
| Kiribati | 1950 to 1959 | 0.988 (0.298, 3.271) |
| Kiribati | 1955 to 1964 | 1.088 (0.465, 2.542) |
| Kiribati | 1960 to 1969 | 1.03 (0.487, 2.177) |
| Kiribati | 1965 to 1974 | 0.976 (0.485, 1.963) |
| Kiribati | 1970 to 1979 | 1 (1, 1) |
| Kiribati | 1975 to 1984 | 0.967 (0.515, 1.814) |
| Kiribati | 1980 to 1989 | 0.96 (0.486, 1.899) |
| Kiribati | 1985 to 1994 | 0.938 (0.441, 1.993) |
| Kiribati | 1990 to 1999 | 0.885 (0.354, 2.215) |
| Kiribati | 1995 to 2004 | 0.93 (0.26, 3.331) |
| Kuwait | 1950 to 1959 | 1.132 (0.948, 1.353) |
| Kuwait | 1955 to 1964 | 1.093 (0.962, 1.243) |
| Kuwait | 1960 to 1969 | 1.054 (0.947, 1.173) |
| Kuwait | 1965 to 1974 | 1.025 (0.931, 1.129) |
| Kuwait | 1970 to 1979 | 1 (1, 1) |
| Kuwait | 1975 to 1984 | 0.975 (0.896, 1.061) |
| Kuwait | 1980 to 1989 | 0.955 (0.871, 1.048) |
| Kuwait | 1985 to 1994 | 0.936 (0.838, 1.045) |
| Kuwait | 1990 to 1999 | 0.914 (0.792, 1.054) |
| Kuwait | 1995 to 2004 | 0.894 (0.726, 1.101) |
| Kyrgyzstan | 1950 to 1959 | 1.061 (0.907, 1.242) |
| Kyrgyzstan | 1955 to 1964 | 1.047 (0.932, 1.175) |
| Kyrgyzstan | 1960 to 1969 | 1.034 (0.933, 1.145) |
| Kyrgyzstan | 1965 to 1974 | 1.015 (0.923, 1.116) |
| Kyrgyzstan | 1970 to 1979 | 1 (1, 1) |
| Kyrgyzstan | 1975 to 1984 | 0.982 (0.9, 1.072) |
| Kyrgyzstan | 1980 to 1989 | 0.97 (0.884, 1.065) |
| Kyrgyzstan | 1985 to 1994 | 0.96 (0.866, 1.064) |
| Kyrgyzstan | 1990 to 1999 | 0.948 (0.835, 1.076) |
| Kyrgyzstan | 1995 to 2004 | 0.944 (0.785, 1.134) |
| Lao People's Democratic Republic | 1950 to 1959 | 1.175 (0.996, 1.387) |
| Lao People's Democratic Republic | 1955 to 1964 | 1.136 (1.006, 1.283) |
| Lao People's Democratic Republic | 1960 to 1969 | 1.09 (0.981, 1.211) |
| Lao People's Democratic Republic | 1965 to 1974 | 1.046 (0.952, 1.149) |
| Lao People's Democratic Republic | 1970 to 1979 | 1 (1, 1) |
| Lao People's Democratic Republic | 1975 to 1984 | 0.956 (0.88, 1.039) |
| Lao People's Democratic Republic | 1980 to 1989 | 0.908 (0.83, 0.992) |
| Lao People's Democratic Republic | 1985 to 1994 | 0.879 (0.798, 0.969) |
| Lao People's Democratic Republic | 1990 to 1999 | 0.862 (0.769, 0.966) |
| Lao People's Democratic Republic | 1995 to 2004 | 0.851 (0.728, 0.995) |
| Latvia | 1950 to 1959 | 1.065 (0.852, 1.33) |
| Latvia | 1955 to 1964 | 1.047 (0.88, 1.246) |
| Latvia | 1960 to 1969 | 1.025 (0.873, 1.203) |
| Latvia | 1965 to 1974 | 1.016 (0.874, 1.181) |
| Latvia | 1970 to 1979 | 1 (1, 1) |
| Latvia | 1975 to 1984 | 0.988 (0.854, 1.144) |
| Latvia | 1980 to 1989 | 0.971 (0.826, 1.142) |
| Latvia | 1985 to 1994 | 0.965 (0.799, 1.165) |
| Latvia | 1990 to 1999 | 0.959 (0.743, 1.239) |
| Latvia | 1995 to 2004 | 0.96 (0.649, 1.421) |
| Lebanon | 1950 to 1959 | 1.217 (1.044, 1.42) |
| Lebanon | 1955 to 1964 | 1.154 (1.037, 1.285) |
| Lebanon | 1960 to 1969 | 1.095 (1.003, 1.197) |
| Lebanon | 1965 to 1974 | 1.042 (0.964, 1.126) |
| Lebanon | 1970 to 1979 | 1 (1, 1) |
| Lebanon | 1975 to 1984 | 0.955 (0.89, 1.024) |
| Lebanon | 1980 to 1989 | 0.919 (0.854, 0.988) |
| Lebanon | 1985 to 1994 | 0.876 (0.809, 0.948) |
| Lebanon | 1990 to 1999 | 0.827 (0.749, 0.912) |
| Lebanon | 1995 to 2004 | 0.784 (0.679, 0.905) |
| Lesotho | 1950 to 1959 | 1.101 (0.916, 1.324) |
| Lesotho | 1955 to 1964 | 1.074 (0.932, 1.237) |
| Lesotho | 1960 to 1969 | 1.05 (0.924, 1.193) |
| Lesotho | 1965 to 1974 | 1.025 (0.911, 1.153) |
| Lesotho | 1970 to 1979 | 1 (1, 1) |
| Lesotho | 1975 to 1984 | 0.976 (0.878, 1.085) |
| Lesotho | 1980 to 1989 | 0.949 (0.843, 1.069) |
| Lesotho | 1985 to 1994 | 0.922 (0.801, 1.06) |
| Lesotho | 1990 to 1999 | 0.899 (0.754, 1.071) |
| Lesotho | 1995 to 2004 | 0.871 (0.674, 1.126) |
| Liberia | 1950 to 1959 | 1.082 (0.891, 1.314) |
| Liberia | 1955 to 1964 | 1.075 (0.931, 1.242) |
| Liberia | 1960 to 1969 | 1.056 (0.93, 1.198) |
| Liberia | 1965 to 1974 | 1.033 (0.926, 1.152) |
| Liberia | 1970 to 1979 | 1 (1, 1) |
| Liberia | 1975 to 1984 | 0.958 (0.873, 1.052) |
| Liberia | 1980 to 1989 | 0.915 (0.826, 1.014) |
| Liberia | 1985 to 1994 | 0.866 (0.769, 0.976) |
| Liberia | 1990 to 1999 | 0.816 (0.706, 0.943) |
| Liberia | 1995 to 2004 | 0.777 (0.635, 0.951) |
| Libya | 1950 to 1959 | 1.143 (0.994, 1.315) |
| Libya | 1955 to 1964 | 1.107 (1.005, 1.219) |
| Libya | 1960 to 1969 | 1.068 (0.989, 1.153) |
| Libya | 1965 to 1974 | 1.033 (0.969, 1.101) |
| Libya | 1970 to 1979 | 1 (1, 1) |
| Libya | 1975 to 1984 | 0.97 (0.917, 1.025) |
| Libya | 1980 to 1989 | 0.942 (0.887, 1) |
| Libya | 1985 to 1994 | 0.916 (0.855, 0.981) |
| Libya | 1990 to 1999 | 0.894 (0.822, 0.972) |
| Libya | 1995 to 2004 | 0.87 (0.773, 0.98) |
| Lithuania | 1950 to 1959 | 1.054 (0.874, 1.271) |
| Lithuania | 1955 to 1964 | 1.04 (0.901, 1.199) |
| Lithuania | 1960 to 1969 | 1.022 (0.898, 1.164) |
| Lithuania | 1965 to 1974 | 1.012 (0.895, 1.144) |
| Lithuania | 1970 to 1979 | 1 (1, 1) |
| Lithuania | 1975 to 1984 | 0.987 (0.873, 1.115) |
| Lithuania | 1980 to 1989 | 0.973 (0.849, 1.115) |
| Lithuania | 1985 to 1994 | 0.967 (0.829, 1.13) |
| Lithuania | 1990 to 1999 | 0.959 (0.788, 1.166) |
| Lithuania | 1995 to 2004 | 0.958 (0.71, 1.294) |
| Luxembourg | 1950 to 1959 | 1.031 (0.605, 1.757) |
| Luxembourg | 1955 to 1964 | 1.025 (0.697, 1.506) |
| Luxembourg | 1960 to 1969 | 1.04 (0.752, 1.438) |
| Luxembourg | 1965 to 1974 | 1.031 (0.763, 1.393) |
| Luxembourg | 1970 to 1979 | 1 (1, 1) |
| Luxembourg | 1975 to 1984 | 0.984 (0.738, 1.313) |
| Luxembourg | 1980 to 1989 | 0.975 (0.714, 1.332) |
| Luxembourg | 1985 to 1994 | 0.997 (0.704, 1.412) |
| Luxembourg | 1990 to 1999 | 0.984 (0.644, 1.504) |
| Luxembourg | 1995 to 2004 | 0.943 (0.509, 1.747) |
| Madagascar | 1950 to 1959 | 1.14 (1.043, 1.246) |
| Madagascar | 1955 to 1964 | 1.107 (1.036, 1.182) |
| Madagascar | 1960 to 1969 | 1.075 (1.014, 1.139) |
| Madagascar | 1965 to 1974 | 1.04 (0.987, 1.096) |
| Madagascar | 1970 to 1979 | 1 (1, 1) |
| Madagascar | 1975 to 1984 | 0.964 (0.92, 1.011) |
| Madagascar | 1980 to 1989 | 0.924 (0.876, 0.975) |
| Madagascar | 1985 to 1994 | 0.889 (0.836, 0.945) |
| Madagascar | 1990 to 1999 | 0.857 (0.796, 0.923) |
| Madagascar | 1995 to 2004 | 0.834 (0.753, 0.923) |
| Malawi | 1950 to 1959 | 1.181 (1.079, 1.293) |
| Malawi | 1955 to 1964 | 1.126 (1.051, 1.207) |
| Malawi | 1960 to 1969 | 1.086 (1.023, 1.153) |
| Malawi | 1965 to 1974 | 1.044 (0.989, 1.102) |
| Malawi | 1970 to 1979 | 1 (1, 1) |
| Malawi | 1975 to 1984 | 0.963 (0.917, 1.011) |
| Malawi | 1980 to 1989 | 0.918 (0.869, 0.969) |
| Malawi | 1985 to 1994 | 0.875 (0.822, 0.932) |
| Malawi | 1990 to 1999 | 0.839 (0.777, 0.905) |
| Malawi | 1995 to 2004 | 0.812 (0.733, 0.899) |
| Malaysia | 1950 to 1959 | 1.257 (1.182, 1.336) |
| Malaysia | 1955 to 1964 | 1.197 (1.143, 1.253) |
| Malaysia | 1960 to 1969 | 1.132 (1.087, 1.178) |
| Malaysia | 1965 to 1974 | 1.066 (1.027, 1.105) |
| Malaysia | 1970 to 1979 | 1 (1, 1) |
| Malaysia | 1975 to 1984 | 0.947 (0.916, 0.979) |
| Malaysia | 1980 to 1989 | 0.904 (0.872, 0.937) |
| Malaysia | 1985 to 1994 | 0.874 (0.839, 0.91) |
| Malaysia | 1990 to 1999 | 0.864 (0.823, 0.906) |
| Malaysia | 1995 to 2004 | 0.871 (0.815, 0.931) |
| Maldives | 1950 to 1959 | 1.297 (0.636, 2.644) |
| Maldives | 1955 to 1964 | 1.249 (0.753, 2.071) |
| Maldives | 1960 to 1969 | 1.135 (0.737, 1.749) |
| Maldives | 1965 to 1974 | 1.074 (0.733, 1.574) |
| Maldives | 1970 to 1979 | 1 (1, 1) |
| Maldives | 1975 to 1984 | 0.956 (0.693, 1.32) |
| Maldives | 1980 to 1989 | 0.923 (0.657, 1.296) |
| Maldives | 1985 to 1994 | 0.898 (0.619, 1.303) |
| Maldives | 1990 to 1999 | 0.875 (0.553, 1.383) |
| Maldives | 1995 to 2004 | 0.88 (0.453, 1.709) |
| Mali | 1950 to 1959 | 1.05 (0.963, 1.144) |
| Mali | 1955 to 1964 | 1.074 (1.008, 1.143) |
| Mali | 1960 to 1969 | 1.072 (1.015, 1.131) |
| Mali | 1965 to 1974 | 1.043 (0.994, 1.095) |
| Mali | 1970 to 1979 | 1 (1, 1) |
| Mali | 1975 to 1984 | 0.955 (0.915, 0.997) |
| Mali | 1980 to 1989 | 0.906 (0.865, 0.95) |
| Mali | 1985 to 1994 | 0.853 (0.809, 0.898) |
| Mali | 1990 to 1999 | 0.801 (0.753, 0.852) |
| Mali | 1995 to 2004 | 0.75 (0.69, 0.816) |
| Malta | 1950 to 1959 | 1.078 (0.622, 1.868) |
| Malta | 1955 to 1964 | 1.05 (0.7, 1.574) |
| Malta | 1960 to 1969 | 1.018 (0.709, 1.461) |
| Malta | 1965 to 1974 | 0.981 (0.706, 1.362) |
| Malta | 1970 to 1979 | 1 (1, 1) |
| Malta | 1975 to 1984 | 0.986 (0.736, 1.32) |
| Malta | 1980 to 1989 | 0.983 (0.716, 1.348) |
| Malta | 1985 to 1994 | 0.988 (0.693, 1.408) |
| Malta | 1990 to 1999 | 0.973 (0.623, 1.521) |
| Malta | 1995 to 2004 | 0.98 (0.499, 1.922) |
| Marshall Islands | 1950 to 1959 | 0.913 (0.172, 4.843) |
| Marshall Islands | 1955 to 1964 | 1.213 (0.386, 3.816) |
| Marshall Islands | 1960 to 1969 | 1.193 (0.439, 3.242) |
| Marshall Islands | 1965 to 1974 | 1.133 (0.456, 2.813) |
| Marshall Islands | 1970 to 1979 | 1 (1, 1) |
| Marshall Islands | 1975 to 1984 | 0.959 (0.42, 2.192) |
| Marshall Islands | 1980 to 1989 | 0.963 (0.387, 2.392) |
| Marshall Islands | 1985 to 1994 | 0.94 (0.332, 2.661) |
| Marshall Islands | 1990 to 1999 | 0.988 (0.286, 3.412) |
| Marshall Islands | 1995 to 2004 | 1.024 (0.181, 5.802) |
| Mauritania | 1950 to 1959 | 1.199 (0.986, 1.458) |
| Mauritania | 1955 to 1964 | 1.153 (0.996, 1.335) |
| Mauritania | 1960 to 1969 | 1.103 (0.971, 1.253) |
| Mauritania | 1965 to 1974 | 1.051 (0.937, 1.18) |
| Mauritania | 1970 to 1979 | 1 (1, 1) |
| Mauritania | 1975 to 1984 | 0.948 (0.852, 1.054) |
| Mauritania | 1980 to 1989 | 0.895 (0.796, 1.005) |
| Mauritania | 1985 to 1994 | 0.847 (0.741, 0.968) |
| Mauritania | 1990 to 1999 | 0.806 (0.686, 0.947) |
| Mauritania | 1995 to 2004 | 0.767 (0.613, 0.959) |
| Mauritius | 1950 to 1959 | 1.208 (0.919, 1.587) |
| Mauritius | 1955 to 1964 | 1.147 (0.93, 1.414) |
| Mauritius | 1960 to 1969 | 1.087 (0.903, 1.308) |
| Mauritius | 1965 to 1974 | 1.032 (0.867, 1.228) |
| Mauritius | 1970 to 1979 | 1 (1, 1) |
| Mauritius | 1975 to 1984 | 0.97 (0.826, 1.139) |
| Mauritius | 1980 to 1989 | 0.949 (0.788, 1.142) |
| Mauritius | 1985 to 1994 | 0.93 (0.756, 1.144) |
| Mauritius | 1990 to 1999 | 0.921 (0.721, 1.177) |
| Mauritius | 1995 to 2004 | 0.924 (0.655, 1.303) |
| Mexico | 1950 to 1959 | 1.065 (1.036, 1.095) |
| Mexico | 1955 to 1964 | 1.043 (1.023, 1.064) |
| Mexico | 1960 to 1969 | 1.026 (1.01, 1.043) |
| Mexico | 1965 to 1974 | 1.012 (0.997, 1.027) |
| Mexico | 1970 to 1979 | 1 (1, 1) |
| Mexico | 1975 to 1984 | 0.976 (0.963, 0.989) |
| Mexico | 1980 to 1989 | 0.952 (0.938, 0.966) |
| Mexico | 1985 to 1994 | 0.93 (0.915, 0.946) |
| Mexico | 1990 to 1999 | 0.907 (0.889, 0.926) |
| Mexico | 1995 to 2004 | 0.892 (0.866, 0.919) |
| Micronesia (Federated States of) | 1950 to 1959 | 1.016 (0.335, 3.081) |
| Micronesia (Federated States of) | 1955 to 1964 | 1.066 (0.467, 2.433) |
| Micronesia (Federated States of) | 1960 to 1969 | 1.041 (0.504, 2.152) |
| Micronesia (Federated States of) | 1965 to 1974 | 1.04 (0.541, 2) |
| Micronesia (Federated States of) | 1970 to 1979 | 1 (1, 1) |
| Micronesia (Federated States of) | 1975 to 1984 | 0.935 (0.509, 1.718) |
| Micronesia (Federated States of) | 1980 to 1989 | 0.924 (0.475, 1.796) |
| Micronesia (Federated States of) | 1985 to 1994 | 0.771 (0.354, 1.677) |
| Micronesia (Federated States of) | 1990 to 1999 | 0.769 (0.31, 1.905) |
| Micronesia (Federated States of) | 1995 to 2004 | 0.82 (0.244, 2.76) |
| Monaco | 1950 to 1959 | 0.786 (0.069, 8.975) |
| Monaco | 1955 to 1964 | 0.852 (0.144, 5.038) |
| Monaco | 1960 to 1969 | 1.002 (0.242, 4.141) |
| Monaco | 1965 to 1974 | 0.98 (0.27, 3.562) |
| Monaco | 1970 to 1979 | 1 (1, 1) |
| Monaco | 1975 to 1984 | 1.004 (0.289, 3.496) |
| Monaco | 1980 to 1989 | 0.996 (0.262, 3.792) |
| Monaco | 1985 to 1994 | 0.911 (0.206, 4.02) |
| Monaco | 1990 to 1999 | 0.885 (0.151, 5.171) |
| Monaco | 1995 to 2004 | 0.793 (0.067, 9.361) |
| Mongolia | 1950 to 1959 | 1.149 (0.942, 1.401) |
| Mongolia | 1955 to 1964 | 1.111 (0.967, 1.276) |
| Mongolia | 1960 to 1969 | 1.072 (0.952, 1.208) |
| Mongolia | 1965 to 1974 | 1.037 (0.93, 1.155) |
| Mongolia | 1970 to 1979 | 1 (1, 1) |
| Mongolia | 1975 to 1984 | 0.964 (0.874, 1.064) |
| Mongolia | 1980 to 1989 | 0.93 (0.836, 1.035) |
| Mongolia | 1985 to 1994 | 0.9 (0.797, 1.016) |
| Mongolia | 1990 to 1999 | 0.876 (0.749, 1.025) |
| Mongolia | 1995 to 2004 | 0.849 (0.673, 1.072) |
| Montenegro | 1950 to 1959 | 1.048 (0.633, 1.737) |
| Montenegro | 1955 to 1964 | 1.023 (0.689, 1.518) |
| Montenegro | 1960 to 1969 | 1.009 (0.706, 1.442) |
| Montenegro | 1965 to 1974 | 1.008 (0.723, 1.405) |
| Montenegro | 1970 to 1979 | 1 (1, 1) |
| Montenegro | 1975 to 1984 | 0.995 (0.731, 1.356) |
| Montenegro | 1980 to 1989 | 0.99 (0.701, 1.4) |
| Montenegro | 1985 to 1994 | 0.963 (0.64, 1.45) |
| Montenegro | 1990 to 1999 | 0.971 (0.591, 1.597) |
| Montenegro | 1995 to 2004 | 0.97 (0.477, 1.976) |
| Morocco | 1950 to 1959 | 1.161 (1.095, 1.23) |
| Morocco | 1955 to 1964 | 1.115 (1.07, 1.162) |
| Morocco | 1960 to 1969 | 1.072 (1.036, 1.109) |
| Morocco | 1965 to 1974 | 1.033 (1.002, 1.064) |
| Morocco | 1970 to 1979 | 1 (1, 1) |
| Morocco | 1975 to 1984 | 0.969 (0.943, 0.995) |
| Morocco | 1980 to 1989 | 0.942 (0.915, 0.97) |
| Morocco | 1985 to 1994 | 0.918 (0.889, 0.948) |
| Morocco | 1990 to 1999 | 0.899 (0.865, 0.935) |
| Morocco | 1995 to 2004 | 0.878 (0.831, 0.928) |
| Mozambique | 1950 to 1959 | 1.151 (1.068, 1.24) |
| Mozambique | 1955 to 1964 | 1.125 (1.064, 1.19) |
| Mozambique | 1960 to 1969 | 1.089 (1.038, 1.144) |
| Mozambique | 1965 to 1974 | 1.044 (1, 1.091) |
| Mozambique | 1970 to 1979 | 1 (1, 1) |
| Mozambique | 1975 to 1984 | 0.961 (0.924, 1) |
| Mozambique | 1980 to 1989 | 0.916 (0.877, 0.957) |
| Mozambique | 1985 to 1994 | 0.875 (0.832, 0.921) |
| Mozambique | 1990 to 1999 | 0.841 (0.792, 0.893) |
| Mozambique | 1995 to 2004 | 0.809 (0.744, 0.879) |
| Myanmar | 1950 to 1959 | 1.281 (1.235, 1.328) |
| Myanmar | 1955 to 1964 | 1.212 (1.179, 1.245) |
| Myanmar | 1960 to 1969 | 1.139 (1.112, 1.166) |
| Myanmar | 1965 to 1974 | 1.067 (1.044, 1.09) |
| Myanmar | 1970 to 1979 | 1 (1, 1) |
| Myanmar | 1975 to 1984 | 0.944 (0.925, 0.963) |
| Myanmar | 1980 to 1989 | 0.888 (0.868, 0.908) |
| Myanmar | 1985 to 1994 | 0.843 (0.822, 0.865) |
| Myanmar | 1990 to 1999 | 0.816 (0.791, 0.841) |
| Myanmar | 1995 to 2004 | 0.803 (0.77, 0.837) |
| Namibia | 1950 to 1959 | 1.157 (0.94, 1.425) |
| Namibia | 1955 to 1964 | 1.119 (0.958, 1.307) |
| Namibia | 1960 to 1969 | 1.082 (0.944, 1.24) |
| Namibia | 1965 to 1974 | 1.041 (0.92, 1.177) |
| Namibia | 1970 to 1979 | 1 (1, 1) |
| Namibia | 1975 to 1984 | 0.962 (0.859, 1.078) |
| Namibia | 1980 to 1989 | 0.921 (0.81, 1.046) |
| Namibia | 1985 to 1994 | 0.878 (0.756, 1.02) |
| Namibia | 1990 to 1999 | 0.84 (0.697, 1.013) |
| Namibia | 1995 to 2004 | 0.807 (0.614, 1.061) |
| Nauru | 1950 to 1959 | 1.092 (0.042, 28.425) |
| Nauru | 1955 to 1964 | 1.091 (0.086, 13.864) |
| Nauru | 1960 to 1969 | 1.067 (0.113, 10.08) |
| Nauru | 1965 to 1974 | 1.041 (0.136, 7.985) |
| Nauru | 1970 to 1979 | 1 (1, 1) |
| Nauru | 1975 to 1984 | 0.98 (0.165, 5.826) |
| Nauru | 1980 to 1989 | 0.949 (0.139, 6.469) |
| Nauru | 1985 to 1994 | 0.933 (0.117, 7.419) |
| Nauru | 1990 to 1999 | 0.94 (0.098, 9.005) |
| Nauru | 1995 to 2004 | 0.984 (0.068, 14.314) |
| Nepal | 1950 to 1959 | 1.022 (0.97, 1.076) |
| Nepal | 1955 to 1964 | 1.057 (1.016, 1.101) |
| Nepal | 1960 to 1969 | 1.052 (1.014, 1.091) |
| Nepal | 1965 to 1974 | 1.025 (0.992, 1.059) |
| Nepal | 1970 to 1979 | 1 (1, 1) |
| Nepal | 1975 to 1984 | 0.973 (0.944, 1.003) |
| Nepal | 1980 to 1989 | 0.949 (0.916, 0.983) |
| Nepal | 1985 to 1994 | 0.912 (0.875, 0.95) |
| Nepal | 1990 to 1999 | 0.878 (0.836, 0.923) |
| Nepal | 1995 to 2004 | 0.846 (0.789, 0.907) |
| Netherlands | 1950 to 1959 | 1.052 (0.955, 1.16) |
| Netherlands | 1955 to 1964 | 1.037 (0.966, 1.113) |
| Netherlands | 1960 to 1969 | 1.024 (0.965, 1.086) |
| Netherlands | 1965 to 1974 | 1.011 (0.958, 1.068) |
| Netherlands | 1970 to 1979 | 1 (1, 1) |
| Netherlands | 1975 to 1984 | 0.989 (0.937, 1.045) |
| Netherlands | 1980 to 1989 | 0.981 (0.925, 1.041) |
| Netherlands | 1985 to 1994 | 0.976 (0.914, 1.041) |
| Netherlands | 1990 to 1999 | 0.97 (0.896, 1.049) |
| Netherlands | 1995 to 2004 | 0.961 (0.859, 1.076) |
| New Zealand | 1950 to 1959 | 1.074 (0.888, 1.299) |
| New Zealand | 1955 to 1964 | 1.051 (0.916, 1.206) |
| New Zealand | 1960 to 1969 | 1.031 (0.916, 1.159) |
| New Zealand | 1965 to 1974 | 1.015 (0.913, 1.129) |
| New Zealand | 1970 to 1979 | 1 (1, 1) |
| New Zealand | 1975 to 1984 | 0.988 (0.892, 1.093) |
| New Zealand | 1980 to 1989 | 0.978 (0.877, 1.09) |
| New Zealand | 1985 to 1994 | 0.97 (0.862, 1.091) |
| New Zealand | 1990 to 1999 | 0.96 (0.836, 1.102) |
| New Zealand | 1995 to 2004 | 0.955 (0.787, 1.158) |
| Nicaragua | 1950 to 1959 | 1.124 (0.99, 1.277) |
| Nicaragua | 1955 to 1964 | 1.084 (0.991, 1.187) |
| Nicaragua | 1960 to 1969 | 1.052 (0.976, 1.134) |
| Nicaragua | 1965 to 1974 | 1.026 (0.961, 1.095) |
| Nicaragua | 1970 to 1979 | 1 (1, 1) |
| Nicaragua | 1975 to 1984 | 0.976 (0.921, 1.034) |
| Nicaragua | 1980 to 1989 | 0.957 (0.9, 1.019) |
| Nicaragua | 1985 to 1994 | 0.939 (0.877, 1.006) |
| Nicaragua | 1990 to 1999 | 0.918 (0.845, 0.998) |
| Nicaragua | 1995 to 2004 | 0.898 (0.796, 1.013) |
| Niger | 1950 to 1959 | 0.995 (0.91, 1.088) |
| Niger | 1955 to 1964 | 1.022 (0.958, 1.089) |
| Niger | 1960 to 1969 | 1.021 (0.965, 1.08) |
| Niger | 1965 to 1974 | 1.011 (0.961, 1.065) |
| Niger | 1970 to 1979 | 1 (1, 1) |
| Niger | 1975 to 1984 | 0.986 (0.942, 1.032) |
| Niger | 1980 to 1989 | 0.968 (0.919, 1.019) |
| Niger | 1985 to 1994 | 0.952 (0.898, 1.01) |
| Niger | 1990 to 1999 | 0.938 (0.875, 1.006) |
| Niger | 1995 to 2004 | 0.919 (0.836, 1.01) |
| Nigeria | 1950 to 1959 | 0.999 (0.972, 1.026) |
| Nigeria | 1955 to 1964 | 1.009 (0.99, 1.029) |
| Nigeria | 1960 to 1969 | 1.007 (0.991, 1.023) |
| Nigeria | 1965 to 1974 | 1.003 (0.989, 1.017) |
| Nigeria | 1970 to 1979 | 1 (1, 1) |
| Nigeria | 1975 to 1984 | 0.994 (0.981, 1.006) |
| Nigeria | 1980 to 1989 | 0.983 (0.97, 0.997) |
| Nigeria | 1985 to 1994 | 0.962 (0.947, 0.977) |
| Nigeria | 1990 to 1999 | 0.935 (0.918, 0.952) |
| Nigeria | 1995 to 2004 | 0.925 (0.902, 0.948) |
| Niue | 1950 to 1959 | 1.18 (0, 6402.318) |
| Niue | 1955 to 1964 | 1.138 (0.001, 1041.049) |
| Niue | 1960 to 1969 | 1.105 (0.002, 531.251) |
| Niue | 1965 to 1974 | 1.05 (0.003, 325.756) |
| Niue | 1970 to 1979 | 1 (1, 1) |
| Niue | 1975 to 1984 | 0.986 (0.004, 243.382) |
| Niue | 1980 to 1989 | 0.96 (0.002, 517.935) |
| Niue | 1985 to 1994 | 1.14 (0.001, 960.92) |
| Niue | 1990 to 1999 | 0.886 (0, 4709.382) |
| Niue | 1995 to 2004 | 0.968 (0, 135892.151) |
| North Macedonia | 1950 to 1959 | 1.074 (0.82, 1.407) |
| North Macedonia | 1955 to 1964 | 1.049 (0.849, 1.295) |
| North Macedonia | 1960 to 1969 | 1.035 (0.856, 1.251) |
| North Macedonia | 1965 to 1974 | 1.018 (0.854, 1.213) |
| North Macedonia | 1970 to 1979 | 1 (1, 1) |
| North Macedonia | 1975 to 1984 | 0.989 (0.84, 1.165) |
| North Macedonia | 1980 to 1989 | 0.97 (0.808, 1.164) |
| North Macedonia | 1985 to 1994 | 0.968 (0.785, 1.195) |
| North Macedonia | 1990 to 1999 | 0.949 (0.729, 1.236) |
| North Macedonia | 1995 to 2004 | 0.958 (0.647, 1.419) |
| Northern Mariana Islands | 1950 to 1959 | 1.032 (0.211, 5.051) |
| Northern Mariana Islands | 1955 to 1964 | 1.062 (0.349, 3.237) |
| Northern Mariana Islands | 1960 to 1969 | 0.903 (0.359, 2.268) |
| Northern Mariana Islands | 1965 to 1974 | 0.858 (0.361, 2.04) |
| Northern Mariana Islands | 1970 to 1979 | 1 (1, 1) |
| Northern Mariana Islands | 1975 to 1984 | 0.904 (0.333, 2.455) |
| Northern Mariana Islands | 1980 to 1989 | 0.998 (0.315, 3.164) |
| Northern Mariana Islands | 1985 to 1994 | 0.959 (0.252, 3.645) |
| Northern Mariana Islands | 1990 to 1999 | 0.887 (0.169, 4.662) |
| Northern Mariana Islands | 1995 to 2004 | 0.797 (0.071, 8.964) |
| Norway | 1950 to 1959 | 1.023 (0.87, 1.202) |
| Norway | 1955 to 1964 | 1.015 (0.901, 1.142) |
| Norway | 1960 to 1969 | 1.011 (0.915, 1.117) |
| Norway | 1965 to 1974 | 1.004 (0.918, 1.098) |
| Norway | 1970 to 1979 | 1 (1, 1) |
| Norway | 1975 to 1984 | 0.995 (0.913, 1.085) |
| Norway | 1980 to 1989 | 0.992 (0.904, 1.089) |
| Norway | 1985 to 1994 | 0.993 (0.897, 1.1) |
| Norway | 1990 to 1999 | 0.987 (0.873, 1.117) |
| Norway | 1995 to 2004 | 0.98 (0.821, 1.17) |
| Oman | 1950 to 1959 | 1.036 (0.902, 1.19) |
| Oman | 1955 to 1964 | 1.056 (0.958, 1.164) |
| Oman | 1960 to 1969 | 1.049 (0.965, 1.139) |
| Oman | 1965 to 1974 | 1.029 (0.955, 1.109) |
| Oman | 1970 to 1979 | 1 (1, 1) |
| Oman | 1975 to 1984 | 0.964 (0.908, 1.025) |
| Oman | 1980 to 1989 | 0.936 (0.879, 0.997) |
| Oman | 1985 to 1994 | 0.905 (0.844, 0.971) |
| Oman | 1990 to 1999 | 0.873 (0.797, 0.956) |
| Oman | 1995 to 2004 | 0.848 (0.74, 0.972) |
| Pakistan | 1950 to 1959 | 1.071 (1.047, 1.095) |
| Pakistan | 1955 to 1964 | 1.056 (1.038, 1.074) |
| Pakistan | 1960 to 1969 | 1.036 (1.021, 1.051) |
| Pakistan | 1965 to 1974 | 1.018 (1.005, 1.032) |
| Pakistan | 1970 to 1979 | 1 (1, 1) |
| Pakistan | 1975 to 1984 | 0.974 (0.963, 0.986) |
| Pakistan | 1980 to 1989 | 0.953 (0.94, 0.965) |
| Pakistan | 1985 to 1994 | 0.931 (0.917, 0.944) |
| Pakistan | 1990 to 1999 | 0.91 (0.894, 0.925) |
| Pakistan | 1995 to 2004 | 0.887 (0.867, 0.908) |
| Palau | 1950 to 1959 | 1.039 (0.088, 12.3) |
| Palau | 1955 to 1964 | 0.945 (0.158, 5.647) |
| Palau | 1960 to 1969 | 0.92 (0.202, 4.193) |
| Palau | 1965 to 1974 | 0.932 (0.241, 3.61) |
| Palau | 1970 to 1979 | 1 (1, 1) |
| Palau | 1975 to 1984 | 0.99 (0.273, 3.597) |
| Palau | 1980 to 1989 | 1.098 (0.288, 4.19) |
| Palau | 1985 to 1994 | 0.74 (0.122, 4.494) |
| Palau | 1990 to 1999 | 0.494 (0.04, 6.142) |
| Palau | 1995 to 2004 | 0.477 (0.012, 18.683) |
| Palestine | 1950 to 1959 | 1.189 (0.965, 1.465) |
| Palestine | 1955 to 1964 | 1.123 (0.978, 1.29) |
| Palestine | 1960 to 1969 | 1.075 (0.964, 1.199) |
| Palestine | 1965 to 1974 | 1.034 (0.942, 1.135) |
| Palestine | 1970 to 1979 | 1 (1, 1) |
| Palestine | 1975 to 1984 | 0.967 (0.891, 1.05) |
| Palestine | 1980 to 1989 | 0.939 (0.863, 1.022) |
| Palestine | 1985 to 1994 | 0.915 (0.836, 1.001) |
| Palestine | 1990 to 1999 | 0.894 (0.805, 0.992) |
| Palestine | 1995 to 2004 | 0.876 (0.759, 1.01) |
| Panama | 1950 to 1959 | 1.087 (0.933, 1.266) |
| Panama | 1955 to 1964 | 1.058 (0.948, 1.181) |
| Panama | 1960 to 1969 | 1.034 (0.943, 1.134) |
| Panama | 1965 to 1974 | 1.017 (0.937, 1.105) |
| Panama | 1970 to 1979 | 1 (1, 1) |
| Panama | 1975 to 1984 | 0.984 (0.911, 1.062) |
| Panama | 1980 to 1989 | 0.966 (0.888, 1.051) |
| Panama | 1985 to 1994 | 0.953 (0.866, 1.048) |
| Panama | 1990 to 1999 | 0.94 (0.837, 1.054) |
| Panama | 1995 to 2004 | 0.929 (0.788, 1.096) |
| Papua New Guinea | 1950 to 1959 | 1.058 (0.923, 1.213) |
| Papua New Guinea | 1955 to 1964 | 1.049 (0.951, 1.157) |
| Papua New Guinea | 1960 to 1969 | 1.034 (0.952, 1.123) |
| Papua New Guinea | 1965 to 1974 | 1.018 (0.946, 1.094) |
| Papua New Guinea | 1970 to 1979 | 1 (1, 1) |
| Papua New Guinea | 1975 to 1984 | 0.984 (0.922, 1.049) |
| Papua New Guinea | 1980 to 1989 | 0.963 (0.898, 1.033) |
| Papua New Guinea | 1985 to 1994 | 0.945 (0.875, 1.022) |
| Papua New Guinea | 1990 to 1999 | 0.932 (0.85, 1.021) |
| Papua New Guinea | 1995 to 2004 | 0.919 (0.811, 1.042) |
| Paraguay | 1950 to 1959 | 1.082 (0.969, 1.209) |
| Paraguay | 1955 to 1964 | 1.056 (0.975, 1.142) |
| Paraguay | 1960 to 1969 | 1.035 (0.968, 1.105) |
| Paraguay | 1965 to 1974 | 1.016 (0.958, 1.078) |
| Paraguay | 1970 to 1979 | 1 (1, 1) |
| Paraguay | 1975 to 1984 | 0.983 (0.934, 1.036) |
| Paraguay | 1980 to 1989 | 0.971 (0.919, 1.026) |
| Paraguay | 1985 to 1994 | 0.958 (0.902, 1.017) |
| Paraguay | 1990 to 1999 | 0.946 (0.88, 1.016) |
| Paraguay | 1995 to 2004 | 0.935 (0.845, 1.034) |
| Peru | 1950 to 1959 | 1.148 (1.093, 1.205) |
| Peru | 1955 to 1964 | 1.106 (1.068, 1.145) |
| Peru | 1960 to 1969 | 1.065 (1.034, 1.097) |
| Peru | 1965 to 1974 | 1.03 (1.003, 1.057) |
| Peru | 1970 to 1979 | 1 (1, 1) |
| Peru | 1975 to 1984 | 0.974 (0.951, 0.997) |
| Peru | 1980 to 1989 | 0.952 (0.928, 0.977) |
| Peru | 1985 to 1994 | 0.933 (0.906, 0.96) |
| Peru | 1990 to 1999 | 0.912 (0.881, 0.945) |
| Peru | 1995 to 2004 | 0.891 (0.848, 0.937) |
| Philippines | 1950 to 1959 | 1.107 (1.067, 1.148) |
| Philippines | 1955 to 1964 | 1.083 (1.054, 1.113) |
| Philippines | 1960 to 1969 | 1.055 (1.031, 1.08) |
| Philippines | 1965 to 1974 | 1.026 (1.005, 1.048) |
| Philippines | 1970 to 1979 | 1 (1, 1) |
| Philippines | 1975 to 1984 | 0.975 (0.957, 0.993) |
| Philippines | 1980 to 1989 | 0.952 (0.933, 0.971) |
| Philippines | 1985 to 1994 | 0.935 (0.914, 0.956) |
| Philippines | 1990 to 1999 | 0.927 (0.903, 0.951) |
| Philippines | 1995 to 2004 | 0.929 (0.897, 0.962) |
| Poland | 1950 to 1959 | 1.015 (0.954, 1.081) |
| Poland | 1955 to 1964 | 1.01 (0.961, 1.062) |
| Poland | 1960 to 1969 | 1.006 (0.96, 1.055) |
| Poland | 1965 to 1974 | 1.003 (0.961, 1.047) |
| Poland | 1970 to 1979 | 1 (1, 1) |
| Poland | 1975 to 1984 | 0.995 (0.958, 1.034) |
| Poland | 1980 to 1989 | 0.992 (0.95, 1.035) |
| Poland | 1985 to 1994 | 0.986 (0.937, 1.037) |
| Poland | 1990 to 1999 | 0.982 (0.92, 1.049) |
| Poland | 1995 to 2004 | 0.98 (0.889, 1.081) |
| Portugal | 1950 to 1959 | 1.084 (0.972, 1.208) |
| Portugal | 1955 to 1964 | 1.06 (0.98, 1.148) |
| Portugal | 1960 to 1969 | 1.038 (0.972, 1.109) |
| Portugal | 1965 to 1974 | 1.018 (0.96, 1.08) |
| Portugal | 1970 to 1979 | 1 (1, 1) |
| Portugal | 1975 to 1984 | 0.986 (0.932, 1.042) |
| Portugal | 1980 to 1989 | 0.972 (0.913, 1.036) |
| Portugal | 1985 to 1994 | 0.961 (0.892, 1.035) |
| Portugal | 1990 to 1999 | 0.952 (0.87, 1.042) |
| Portugal | 1995 to 2004 | 0.939 (0.824, 1.069) |
| Puerto Rico | 1950 to 1959 | 1.102 (0.933, 1.303) |
| Puerto Rico | 1955 to 1964 | 1.07 (0.944, 1.214) |
| Puerto Rico | 1960 to 1969 | 1.041 (0.934, 1.161) |
| Puerto Rico | 1965 to 1974 | 1.016 (0.919, 1.123) |
| Puerto Rico | 1970 to 1979 | 1 (1, 1) |
| Puerto Rico | 1975 to 1984 | 0.986 (0.896, 1.084) |
| Puerto Rico | 1980 to 1989 | 0.971 (0.873, 1.081) |
| Puerto Rico | 1985 to 1994 | 0.963 (0.853, 1.086) |
| Puerto Rico | 1990 to 1999 | 0.952 (0.822, 1.104) |
| Puerto Rico | 1995 to 2004 | 0.947 (0.762, 1.176) |
| Qatar | 1950 to 1959 | 1.227 (0.934, 1.611) |
| Qatar | 1955 to 1964 | 1.159 (0.956, 1.405) |
| Qatar | 1960 to 1969 | 1.1 (0.927, 1.305) |
| Qatar | 1965 to 1974 | 1.044 (0.903, 1.208) |
| Qatar | 1970 to 1979 | 1 (1, 1) |
| Qatar | 1975 to 1984 | 0.967 (0.862, 1.085) |
| Qatar | 1980 to 1989 | 0.921 (0.81, 1.047) |
| Qatar | 1985 to 1994 | 0.885 (0.757, 1.035) |
| Qatar | 1990 to 1999 | 0.848 (0.689, 1.043) |
| Qatar | 1995 to 2004 | 0.82 (0.595, 1.13) |
| Republic of Korea | 1950 to 1959 | 1.154 (1.099, 1.21) |
| Republic of Korea | 1955 to 1964 | 1.102 (1.065, 1.14) |
| Republic of Korea | 1960 to 1969 | 1.059 (1.028, 1.091) |
| Republic of Korea | 1965 to 1974 | 1.024 (0.998, 1.052) |
| Republic of Korea | 1970 to 1979 | 1 (1, 1) |
| Republic of Korea | 1975 to 1984 | 0.979 (0.954, 1.004) |
| Republic of Korea | 1980 to 1989 | 0.962 (0.935, 0.99) |
| Republic of Korea | 1985 to 1994 | 0.946 (0.916, 0.977) |
| Republic of Korea | 1990 to 1999 | 0.931 (0.896, 0.967) |
| Republic of Korea | 1995 to 2004 | 0.916 (0.865, 0.971) |
| Republic of Moldova | 1950 to 1959 | 1.077 (0.938, 1.236) |
| Republic of Moldova | 1955 to 1964 | 1.056 (0.948, 1.176) |
| Republic of Moldova | 1960 to 1969 | 1.034 (0.935, 1.144) |
| Republic of Moldova | 1965 to 1974 | 1.015 (0.922, 1.118) |
| Republic of Moldova | 1970 to 1979 | 1 (1, 1) |
| Republic of Moldova | 1975 to 1984 | 0.983 (0.9, 1.073) |
| Republic of Moldova | 1980 to 1989 | 0.968 (0.879, 1.066) |
| Republic of Moldova | 1985 to 1994 | 0.954 (0.852, 1.069) |
| Republic of Moldova | 1990 to 1999 | 0.949 (0.816, 1.104) |
| Republic of Moldova | 1995 to 2004 | 0.947 (0.755, 1.187) |
| Romania | 1950 to 1959 | 1.056 (0.973, 1.146) |
| Romania | 1955 to 1964 | 1.036 (0.97, 1.108) |
| Romania | 1960 to 1969 | 1.021 (0.96, 1.086) |
| Romania | 1965 to 1974 | 1.01 (0.958, 1.064) |
| Romania | 1970 to 1979 | 1 (1, 1) |
| Romania | 1975 to 1984 | 0.988 (0.939, 1.04) |
| Romania | 1980 to 1989 | 0.977 (0.921, 1.037) |
| Romania | 1985 to 1994 | 0.972 (0.906, 1.044) |
| Romania | 1990 to 1999 | 0.965 (0.879, 1.06) |
| Romania | 1995 to 2004 | 0.961 (0.84, 1.098) |
| Russian Federation | 1950 to 1959 | 1.03 (1.006, 1.056) |
| Russian Federation | 1955 to 1964 | 1.033 (1.014, 1.053) |
| Russian Federation | 1960 to 1969 | 1.027 (1.008, 1.045) |
| Russian Federation | 1965 to 1974 | 1.014 (0.997, 1.031) |
| Russian Federation | 1970 to 1979 | 1 (1, 1) |
| Russian Federation | 1975 to 1984 | 0.985 (0.97, 1.001) |
| Russian Federation | 1980 to 1989 | 0.973 (0.957, 0.989) |
| Russian Federation | 1985 to 1994 | 0.962 (0.943, 0.982) |
| Russian Federation | 1990 to 1999 | 0.955 (0.929, 0.982) |
| Russian Federation | 1995 to 2004 | 0.952 (0.914, 0.991) |
| Rwanda | 1950 to 1959 | 1.217 (1.066, 1.389) |
| Rwanda | 1955 to 1964 | 1.163 (1.052, 1.285) |
| Rwanda | 1960 to 1969 | 1.112 (1.016, 1.217) |
| Rwanda | 1965 to 1974 | 1.06 (0.976, 1.152) |
| Rwanda | 1970 to 1979 | 1 (1, 1) |
| Rwanda | 1975 to 1984 | 0.948 (0.882, 1.019) |
| Rwanda | 1980 to 1989 | 0.894 (0.826, 0.968) |
| Rwanda | 1985 to 1994 | 0.849 (0.773, 0.932) |
| Rwanda | 1990 to 1999 | 0.815 (0.726, 0.914) |
| Rwanda | 1995 to 2004 | 0.79 (0.674, 0.925) |
| Saint Kitts and Nevis | 1950 to 1959 | 1.115 (0.272, 4.57) |
| Saint Kitts and Nevis | 1955 to 1964 | 1.067 (0.378, 3.01) |
| Saint Kitts and Nevis | 1960 to 1969 | 1.112 (0.449, 2.753) |
| Saint Kitts and Nevis | 1965 to 1974 | 1.043 (0.454, 2.401) |
| Saint Kitts and Nevis | 1970 to 1979 | 1 (1, 1) |
| Saint Kitts and Nevis | 1975 to 1984 | 0.96 (0.438, 2.104) |
| Saint Kitts and Nevis | 1980 to 1989 | 0.977 (0.416, 2.29) |
| Saint Kitts and Nevis | 1985 to 1994 | 0.997 (0.374, 2.657) |
| Saint Kitts and Nevis | 1990 to 1999 | 0.906 (0.265, 3.089) |
| Saint Kitts and Nevis | 1995 to 2004 | 0.949 (0.17, 5.314) |
| Saint Lucia | 1950 to 1959 | 1.081 (0.469, 2.488) |
| Saint Lucia | 1955 to 1964 | 1.099 (0.61, 1.981) |
| Saint Lucia | 1960 to 1969 | 1.062 (0.646, 1.747) |
| Saint Lucia | 1965 to 1974 | 1.016 (0.648, 1.591) |
| Saint Lucia | 1970 to 1979 | 1 (1, 1) |
| Saint Lucia | 1975 to 1984 | 0.958 (0.63, 1.458) |
| Saint Lucia | 1980 to 1989 | 0.956 (0.602, 1.517) |
| Saint Lucia | 1985 to 1994 | 0.951 (0.566, 1.598) |
| Saint Lucia | 1990 to 1999 | 0.899 (0.477, 1.693) |
| Saint Lucia | 1995 to 2004 | 0.812 (0.312, 2.115) |
| Saint Vincent and the Grenadines | 1950 to 1959 | 1.19 (0.469, 3.019) |
| Saint Vincent and the Grenadines | 1955 to 1964 | 1.042 (0.525, 2.069) |
| Saint Vincent and the Grenadines | 1960 to 1969 | 1.03 (0.575, 1.845) |
| Saint Vincent and the Grenadines | 1965 to 1974 | 0.989 (0.58, 1.687) |
| Saint Vincent and the Grenadines | 1970 to 1979 | 1 (1, 1) |
| Saint Vincent and the Grenadines | 1975 to 1984 | 0.98 (0.594, 1.615) |
| Saint Vincent and the Grenadines | 1980 to 1989 | 0.934 (0.532, 1.641) |
| Saint Vincent and the Grenadines | 1985 to 1994 | 0.959 (0.507, 1.815) |
| Saint Vincent and the Grenadines | 1990 to 1999 | 0.995 (0.464, 2.132) |
| Saint Vincent and the Grenadines | 1995 to 2004 | 0.871 (0.273, 2.782) |
| Samoa | 1950 to 1959 | 1.011 (0.406, 2.517) |
| Samoa | 1955 to 1964 | 1.042 (0.53, 2.051) |
| Samoa | 1960 to 1969 | 1.046 (0.584, 1.875) |
| Samoa | 1965 to 1974 | 1.051 (0.622, 1.776) |
| Samoa | 1970 to 1979 | 1 (1, 1) |
| Samoa | 1975 to 1984 | 0.954 (0.579, 1.572) |
| Samoa | 1980 to 1989 | 0.962 (0.555, 1.669) |
| Samoa | 1985 to 1994 | 0.929 (0.504, 1.713) |
| Samoa | 1990 to 1999 | 0.967 (0.484, 1.934) |
| Samoa | 1995 to 2004 | 0.865 (0.334, 2.24) |
| San Marino | 1950 to 1959 | 1.253 (0.109, 14.359) |
| San Marino | 1955 to 1964 | 1.188 (0.201, 7.031) |
| San Marino | 1960 to 1969 | 1.039 (0.232, 4.651) |
| San Marino | 1965 to 1974 | 1.206 (0.332, 4.385) |
| San Marino | 1970 to 1979 | 1 (1, 1) |
| San Marino | 1975 to 1984 | 1.086 (0.312, 3.781) |
| San Marino | 1980 to 1989 | 1.143 (0.3, 4.353) |
| San Marino | 1985 to 1994 | 1.075 (0.243, 4.757) |
| San Marino | 1990 to 1999 | 1.049 (0.179, 6.133) |
| San Marino | 1995 to 2004 | 0.983 (0.083, 11.624) |
| Sao Tome and Principe | 1950 to 1959 | 1.237 (0.507, 3.019) |
| Sao Tome and Principe | 1955 to 1964 | 1.141 (0.594, 2.193) |
| Sao Tome and Principe | 1960 to 1969 | 1.117 (0.639, 1.955) |
| Sao Tome and Principe | 1965 to 1974 | 1.035 (0.626, 1.711) |
| Sao Tome and Principe | 1970 to 1979 | 1 (1, 1) |
| Sao Tome and Principe | 1975 to 1984 | 0.912 (0.582, 1.432) |
| Sao Tome and Principe | 1980 to 1989 | 0.908 (0.555, 1.488) |
| Sao Tome and Principe | 1985 to 1994 | 0.873 (0.493, 1.544) |
| Sao Tome and Principe | 1990 to 1999 | 0.836 (0.414, 1.689) |
| Sao Tome and Principe | 1995 to 2004 | 0.755 (0.276, 2.07) |
| Saudi Arabia | 1950 to 1959 | 1.412 (1.348, 1.479) |
| Saudi Arabia | 1955 to 1964 | 1.293 (1.251, 1.336) |
| Saudi Arabia | 1960 to 1969 | 1.184 (1.153, 1.217) |
| Saudi Arabia | 1965 to 1974 | 1.089 (1.063, 1.115) |
| Saudi Arabia | 1970 to 1979 | 1 (1, 1) |
| Saudi Arabia | 1975 to 1984 | 0.917 (0.899, 0.937) |
| Saudi Arabia | 1980 to 1989 | 0.842 (0.823, 0.861) |
| Saudi Arabia | 1985 to 1994 | 0.774 (0.755, 0.793) |
| Saudi Arabia | 1990 to 1999 | 0.713 (0.691, 0.735) |
| Saudi Arabia | 1995 to 2004 | 0.661 (0.632, 0.691) |
| Senegal | 1950 to 1959 | 1.177 (1.063, 1.302) |
| Senegal | 1955 to 1964 | 1.139 (1.058, 1.227) |
| Senegal | 1960 to 1969 | 1.099 (1.031, 1.171) |
| Senegal | 1965 to 1974 | 1.053 (0.995, 1.115) |
| Senegal | 1970 to 1979 | 1 (1, 1) |
| Senegal | 1975 to 1984 | 0.949 (0.901, 0.999) |
| Senegal | 1980 to 1989 | 0.894 (0.844, 0.946) |
| Senegal | 1985 to 1994 | 0.842 (0.789, 0.899) |
| Senegal | 1990 to 1999 | 0.789 (0.729, 0.854) |
| Senegal | 1995 to 2004 | 0.74 (0.662, 0.827) |
| Serbia | 1950 to 1959 | 1.049 (0.921, 1.195) |
| Serbia | 1955 to 1964 | 1.034 (0.933, 1.146) |
| Serbia | 1960 to 1969 | 1.021 (0.93, 1.121) |
| Serbia | 1965 to 1974 | 1.011 (0.927, 1.102) |
| Serbia | 1970 to 1979 | 1 (1, 1) |
| Serbia | 1975 to 1984 | 0.988 (0.911, 1.072) |
| Serbia | 1980 to 1989 | 0.983 (0.896, 1.078) |
| Serbia | 1985 to 1994 | 0.978 (0.878, 1.09) |
| Serbia | 1990 to 1999 | 0.974 (0.851, 1.114) |
| Serbia | 1995 to 2004 | 0.974 (0.801, 1.184) |
| Seychelles | 1950 to 1959 | 1.212 (0.413, 3.559) |
| Seychelles | 1955 to 1964 | 1.062 (0.478, 2.357) |
| Seychelles | 1960 to 1969 | 1.125 (0.574, 2.205) |
| Seychelles | 1965 to 1974 | 1.061 (0.573, 1.962) |
| Seychelles | 1970 to 1979 | 1 (1, 1) |
| Seychelles | 1975 to 1984 | 0.973 (0.547, 1.732) |
| Seychelles | 1980 to 1989 | 0.959 (0.505, 1.823) |
| Seychelles | 1985 to 1994 | 0.953 (0.46, 1.976) |
| Seychelles | 1990 to 1999 | 0.867 (0.348, 2.16) |
| Seychelles | 1995 to 2004 | 1.039 (0.305, 3.543) |
| Sierra Leone | 1950 to 1959 | 0.802 (0.678, 0.949) |
| Sierra Leone | 1955 to 1964 | 0.832 (0.738, 0.938) |
| Sierra Leone | 1960 to 1969 | 0.886 (0.801, 0.979) |
| Sierra Leone | 1965 to 1974 | 0.952 (0.871, 1.039) |
| Sierra Leone | 1970 to 1979 | 1 (1, 1) |
| Sierra Leone | 1975 to 1984 | 1.002 (0.929, 1.081) |
| Sierra Leone | 1980 to 1989 | 0.993 (0.915, 1.079) |
| Sierra Leone | 1985 to 1994 | 0.97 (0.883, 1.065) |
| Sierra Leone | 1990 to 1999 | 0.939 (0.839, 1.051) |
| Sierra Leone | 1995 to 2004 | 0.91 (0.78, 1.062) |
| Singapore | 1950 to 1959 | 1.053 (0.902, 1.23) |
| Singapore | 1955 to 1964 | 1.041 (0.934, 1.16) |
| Singapore | 1960 to 1969 | 1.026 (0.937, 1.123) |
| Singapore | 1965 to 1974 | 1.014 (0.936, 1.099) |
| Singapore | 1970 to 1979 | 1 (1, 1) |
| Singapore | 1975 to 1984 | 0.985 (0.917, 1.058) |
| Singapore | 1980 to 1989 | 0.973 (0.897, 1.055) |
| Singapore | 1985 to 1994 | 0.962 (0.88, 1.052) |
| Singapore | 1990 to 1999 | 0.956 (0.851, 1.075) |
| Singapore | 1995 to 2004 | 0.94 (0.784, 1.126) |
| Slovakia | 1950 to 1959 | 1.048 (0.889, 1.236) |
| Slovakia | 1955 to 1964 | 1.03 (0.903, 1.175) |
| Slovakia | 1960 to 1969 | 1.021 (0.906, 1.151) |
| Slovakia | 1965 to 1974 | 1.01 (0.905, 1.129) |
| Slovakia | 1970 to 1979 | 1 (1, 1) |
| Slovakia | 1975 to 1984 | 0.994 (0.9, 1.098) |
| Slovakia | 1980 to 1989 | 0.986 (0.88, 1.105) |
| Slovakia | 1985 to 1994 | 0.981 (0.857, 1.122) |
| Slovakia | 1990 to 1999 | 0.974 (0.817, 1.161) |
| Slovakia | 1995 to 2004 | 0.968 (0.743, 1.261) |
| Slovenia | 1950 to 1959 | 1.05 (0.793, 1.389) |
| Slovenia | 1955 to 1964 | 1.034 (0.826, 1.293) |
| Slovenia | 1960 to 1969 | 1.015 (0.83, 1.243) |
| Slovenia | 1965 to 1974 | 1.009 (0.835, 1.219) |
| Slovenia | 1970 to 1979 | 1 (1, 1) |
| Slovenia | 1975 to 1984 | 0.992 (0.831, 1.183) |
| Slovenia | 1980 to 1989 | 0.982 (0.802, 1.204) |
| Slovenia | 1985 to 1994 | 0.979 (0.767, 1.25) |
| Slovenia | 1990 to 1999 | 0.973 (0.709, 1.335) |
| Slovenia | 1995 to 2004 | 0.957 (0.601, 1.522) |
| Solomon Islands | 1950 to 1959 | 1.101 (0.622, 1.949) |
| Solomon Islands | 1955 to 1964 | 1.104 (0.734, 1.662) |
| Solomon Islands | 1960 to 1969 | 1.057 (0.749, 1.491) |
| Solomon Islands | 1965 to 1974 | 1.044 (0.777, 1.402) |
| Solomon Islands | 1970 to 1979 | 1 (1, 1) |
| Solomon Islands | 1975 to 1984 | 0.985 (0.756, 1.283) |
| Solomon Islands | 1980 to 1989 | 0.95 (0.709, 1.275) |
| Solomon Islands | 1985 to 1994 | 0.938 (0.674, 1.305) |
| Solomon Islands | 1990 to 1999 | 0.932 (0.631, 1.378) |
| Solomon Islands | 1995 to 2004 | 0.904 (0.528, 1.547) |
| Somalia | 1950 to 1959 | 1.031 (0.931, 1.141) |
| Somalia | 1955 to 1964 | 1.027 (0.948, 1.112) |
| Somalia | 1960 to 1969 | 1.023 (0.952, 1.099) |
| Somalia | 1965 to 1974 | 1.015 (0.956, 1.078) |
| Somalia | 1970 to 1979 | 1 (1, 1) |
| Somalia | 1975 to 1984 | 0.989 (0.941, 1.039) |
| Somalia | 1980 to 1989 | 0.963 (0.91, 1.019) |
| Somalia | 1985 to 1994 | 0.935 (0.875, 1) |
| Somalia | 1990 to 1999 | 0.915 (0.845, 0.99) |
| Somalia | 1995 to 2004 | 0.892 (0.8, 0.993) |
| South Africa | 1950 to 1959 | 1.072 (1.032, 1.114) |
| South Africa | 1955 to 1964 | 1.091 (1.06, 1.123) |
| South Africa | 1960 to 1969 | 1.072 (1.044, 1.1) |
| South Africa | 1965 to 1974 | 1.035 (1.011, 1.06) |
| South Africa | 1970 to 1979 | 1 (1, 1) |
| South Africa | 1975 to 1984 | 0.963 (0.942, 0.985) |
| South Africa | 1980 to 1989 | 0.925 (0.902, 0.948) |
| South Africa | 1985 to 1994 | 0.888 (0.861, 0.915) |
| South Africa | 1990 to 1999 | 0.861 (0.827, 0.896) |
| South Africa | 1995 to 2004 | 0.841 (0.791, 0.894) |
| South Sudan | 1950 to 1959 | 1.01 (0.891, 1.146) |
| South Sudan | 1955 to 1964 | 1.033 (0.944, 1.13) |
| South Sudan | 1960 to 1969 | 1.042 (0.966, 1.124) |
| South Sudan | 1965 to 1974 | 1.027 (0.961, 1.097) |
| South Sudan | 1970 to 1979 | 1 (1, 1) |
| South Sudan | 1975 to 1984 | 0.971 (0.915, 1.031) |
| South Sudan | 1980 to 1989 | 0.942 (0.882, 1.006) |
| South Sudan | 1985 to 1994 | 0.922 (0.856, 0.992) |
| South Sudan | 1990 to 1999 | 0.908 (0.834, 0.988) |
| South Sudan | 1995 to 2004 | 0.903 (0.81, 1.008) |
| Spain | 1950 to 1959 | 1.02 (0.981, 1.061) |
| Spain | 1955 to 1964 | 1.012 (0.985, 1.041) |
| Spain | 1960 to 1969 | 1.004 (0.982, 1.028) |
| Spain | 1965 to 1974 | 1 (0.98, 1.021) |
| Spain | 1970 to 1979 | 1 (1, 1) |
| Spain | 1975 to 1984 | 0.998 (0.979, 1.017) |
| Spain | 1980 to 1989 | 0.994 (0.973, 1.016) |
| Spain | 1985 to 1994 | 0.994 (0.969, 1.02) |
| Spain | 1990 to 1999 | 0.994 (0.962, 1.027) |
| Spain | 1995 to 2004 | 0.99 (0.944, 1.039) |
| Sri Lanka | 1950 to 1959 | 1.151 (1.074, 1.233) |
| Sri Lanka | 1955 to 1964 | 1.118 (1.06, 1.179) |
| Sri Lanka | 1960 to 1969 | 1.079 (1.03, 1.131) |
| Sri Lanka | 1965 to 1974 | 1.038 (0.995, 1.083) |
| Sri Lanka | 1970 to 1979 | 1 (1, 1) |
| Sri Lanka | 1975 to 1984 | 0.969 (0.932, 1.008) |
| Sri Lanka | 1980 to 1989 | 0.943 (0.902, 0.985) |
| Sri Lanka | 1985 to 1994 | 0.925 (0.88, 0.972) |
| Sri Lanka | 1990 to 1999 | 0.917 (0.865, 0.971) |
| Sri Lanka | 1995 to 2004 | 0.914 (0.845, 0.989) |
| Sudan | 1950 to 1959 | 1.172 (1.105, 1.244) |
| Sudan | 1955 to 1964 | 1.128 (1.082, 1.176) |
| Sudan | 1960 to 1969 | 1.084 (1.048, 1.121) |
| Sudan | 1965 to 1974 | 1.041 (1.011, 1.072) |
| Sudan | 1970 to 1979 | 1 (1, 1) |
| Sudan | 1975 to 1984 | 0.961 (0.937, 0.986) |
| Sudan | 1980 to 1989 | 0.924 (0.899, 0.949) |
| Sudan | 1985 to 1994 | 0.886 (0.86, 0.912) |
| Sudan | 1990 to 1999 | 0.842 (0.814, 0.871) |
| Sudan | 1995 to 2004 | 0.8 (0.765, 0.838) |
| Suriname | 1950 to 1959 | 1.097 (0.708, 1.699) |
| Suriname | 1955 to 1964 | 1.082 (0.797, 1.469) |
| Suriname | 1960 to 1969 | 1.047 (0.811, 1.352) |
| Suriname | 1965 to 1974 | 1.025 (0.814, 1.291) |
| Suriname | 1970 to 1979 | 1 (1, 1) |
| Suriname | 1975 to 1984 | 0.981 (0.789, 1.219) |
| Suriname | 1980 to 1989 | 0.973 (0.768, 1.233) |
| Suriname | 1985 to 1994 | 0.959 (0.729, 1.26) |
| Suriname | 1990 to 1999 | 0.955 (0.683, 1.335) |
| Suriname | 1995 to 2004 | 0.946 (0.588, 1.522) |
| Sweden | 1950 to 1959 | 1.024 (0.886, 1.184) |
| Sweden | 1955 to 1964 | 1.018 (0.915, 1.134) |
| Sweden | 1960 to 1969 | 1.011 (0.924, 1.106) |
| Sweden | 1965 to 1974 | 1.005 (0.926, 1.092) |
| Sweden | 1970 to 1979 | 1 (1, 1) |
| Sweden | 1975 to 1984 | 0.997 (0.921, 1.079) |
| Sweden | 1980 to 1989 | 0.997 (0.915, 1.085) |
| Sweden | 1985 to 1994 | 0.999 (0.911, 1.097) |
| Sweden | 1990 to 1999 | 0.998 (0.888, 1.122) |
| Sweden | 1995 to 2004 | 1.004 (0.846, 1.192) |
| Switzerland | 1950 to 1959 | 1.028 (0.899, 1.175) |
| Switzerland | 1955 to 1964 | 1.021 (0.927, 1.124) |
| Switzerland | 1960 to 1969 | 1.013 (0.933, 1.099) |
| Switzerland | 1965 to 1974 | 1.005 (0.931, 1.085) |
| Switzerland | 1970 to 1979 | 1 (1, 1) |
| Switzerland | 1975 to 1984 | 0.994 (0.924, 1.07) |
| Switzerland | 1980 to 1989 | 0.988 (0.913, 1.069) |
| Switzerland | 1985 to 1994 | 0.985 (0.901, 1.076) |
| Switzerland | 1990 to 1999 | 0.981 (0.879, 1.095) |
| Switzerland | 1995 to 2004 | 0.972 (0.827, 1.142) |
| Syrian Arab Republic | 1950 to 1959 | 1.158 (1.069, 1.255) |
| Syrian Arab Republic | 1955 to 1964 | 1.115 (1.055, 1.178) |
| Syrian Arab Republic | 1960 to 1969 | 1.071 (1.025, 1.119) |
| Syrian Arab Republic | 1965 to 1974 | 1.034 (0.996, 1.073) |
| Syrian Arab Republic | 1970 to 1979 | 1 (1, 1) |
| Syrian Arab Republic | 1975 to 1984 | 0.972 (0.94, 1.004) |
| Syrian Arab Republic | 1980 to 1989 | 0.947 (0.913, 0.982) |
| Syrian Arab Republic | 1985 to 1994 | 0.923 (0.884, 0.963) |
| Syrian Arab Republic | 1990 to 1999 | 0.897 (0.854, 0.942) |
| Syrian Arab Republic | 1995 to 2004 | 0.87 (0.815, 0.928) |
| Taiwan (Province of China) | 1950 to 1959 | 1.074 (0.969, 1.191) |
| Taiwan (Province of China) | 1955 to 1964 | 1.051 (0.969, 1.14) |
| Taiwan (Province of China) | 1960 to 1969 | 1.03 (0.956, 1.11) |
| Taiwan (Province of China) | 1965 to 1974 | 1.016 (0.947, 1.089) |
| Taiwan (Province of China) | 1970 to 1979 | 1 (1, 1) |
| Taiwan (Province of China) | 1975 to 1984 | 0.985 (0.924, 1.051) |
| Taiwan (Province of China) | 1980 to 1989 | 0.969 (0.899, 1.046) |
| Taiwan (Province of China) | 1985 to 1994 | 0.953 (0.871, 1.042) |
| Taiwan (Province of China) | 1990 to 1999 | 0.942 (0.846, 1.048) |
| Taiwan (Province of China) | 1995 to 2004 | 0.927 (0.793, 1.083) |
| Tajikistan | 1950 to 1959 | 1.051 (0.916, 1.205) |
| Tajikistan | 1955 to 1964 | 1.042 (0.945, 1.149) |
| Tajikistan | 1960 to 1969 | 1.028 (0.945, 1.119) |
| Tajikistan | 1965 to 1974 | 1.013 (0.938, 1.095) |
| Tajikistan | 1970 to 1979 | 1 (1, 1) |
| Tajikistan | 1975 to 1984 | 0.987 (0.921, 1.057) |
| Tajikistan | 1980 to 1989 | 0.976 (0.907, 1.049) |
| Tajikistan | 1985 to 1994 | 0.965 (0.89, 1.045) |
| Tajikistan | 1990 to 1999 | 0.958 (0.87, 1.055) |
| Tajikistan | 1995 to 2004 | 0.952 (0.832, 1.089) |
| Thailand | 1950 to 1959 | 1.161 (1.12, 1.204) |
| Thailand | 1955 to 1964 | 1.122 (1.091, 1.153) |
| Thailand | 1960 to 1969 | 1.078 (1.052, 1.105) |
| Thailand | 1965 to 1974 | 1.036 (1.014, 1.059) |
| Thailand | 1970 to 1979 | 1 (1, 1) |
| Thailand | 1975 to 1984 | 0.971 (0.951, 0.992) |
| Thailand | 1980 to 1989 | 0.941 (0.918, 0.963) |
| Thailand | 1985 to 1994 | 0.922 (0.897, 0.947) |
| Thailand | 1990 to 1999 | 0.911 (0.882, 0.941) |
| Thailand | 1995 to 2004 | 0.904 (0.863, 0.946) |
| Timor-Leste | 1950 to 1959 | 1.259 (0.963, 1.646) |
| Timor-Leste | 1955 to 1964 | 1.206 (0.985, 1.477) |
| Timor-Leste | 1960 to 1969 | 1.142 (0.957, 1.362) |
| Timor-Leste | 1965 to 1974 | 1.066 (0.907, 1.254) |
| Timor-Leste | 1970 to 1979 | 1 (1, 1) |
| Timor-Leste | 1975 to 1984 | 0.946 (0.812, 1.102) |
| Timor-Leste | 1980 to 1989 | 0.893 (0.758, 1.052) |
| Timor-Leste | 1985 to 1994 | 0.849 (0.711, 1.013) |
| Timor-Leste | 1990 to 1999 | 0.818 (0.669, 1) |
| Timor-Leste | 1995 to 2004 | 0.792 (0.607, 1.034) |
| Togo | 1950 to 1959 | 0.975 (0.835, 1.138) |
| Togo | 1955 to 1964 | 1.006 (0.903, 1.12) |
| Togo | 1960 to 1969 | 1.017 (0.929, 1.113) |
| Togo | 1965 to 1974 | 1.014 (0.936, 1.099) |
| Togo | 1970 to 1979 | 1 (1, 1) |
| Togo | 1975 to 1984 | 0.974 (0.907, 1.047) |
| Togo | 1980 to 1989 | 0.95 (0.877, 1.029) |
| Togo | 1985 to 1994 | 0.921 (0.839, 1.01) |
| Togo | 1990 to 1999 | 0.889 (0.793, 0.996) |
| Togo | 1995 to 2004 | 0.856 (0.729, 1.004) |
| Tokelau | 1950 to 1959 | 1.199 (0, 28697.591) |
| Tokelau | 1955 to 1964 | 1.145 (0.001, 2155.747) |
| Tokelau | 1960 to 1969 | 1.091 (0.001, 959.952) |
| Tokelau | 1965 to 1974 | 1.037 (0.002, 543.289) |
| Tokelau | 1970 to 1979 | 1 (1, 1) |
| Tokelau | 1975 to 1984 | 0.956 (0.002, 395.318) |
| Tokelau | 1980 to 1989 | 0.93 (0.001, 649.244) |
| Tokelau | 1985 to 1994 | 0.895 (0, 1802.556) |
| Tokelau | 1990 to 1999 | 0.878 (0, 6033.25) |
| Tokelau | 1995 to 2004 | 0.864 (0, 205354.914) |
| Tonga | 1950 to 1959 | 1.276 (0.305, 5.34) |
| Tonga | 1955 to 1964 | 1.121 (0.363, 3.462) |
| Tonga | 1960 to 1969 | 1.02 (0.384, 2.708) |
| Tonga | 1965 to 1974 | 1.225 (0.543, 2.763) |
| Tonga | 1970 to 1979 | 1 (1, 1) |
| Tonga | 1975 to 1984 | 1.033 (0.472, 2.26) |
| Tonga | 1980 to 1989 | 0.946 (0.393, 2.279) |
| Tonga | 1985 to 1994 | 1.013 (0.386, 2.655) |
| Tonga | 1990 to 1999 | 1.016 (0.334, 3.088) |
| Tonga | 1995 to 2004 | 0.732 (0.139, 3.861) |
| Trinidad and Tobago | 1950 to 1959 | 1.055 (0.816, 1.364) |
| Trinidad and Tobago | 1955 to 1964 | 1.051 (0.871, 1.268) |
| Trinidad and Tobago | 1960 to 1969 | 1.042 (0.883, 1.23) |
| Trinidad and Tobago | 1965 to 1974 | 1.022 (0.873, 1.195) |
| Trinidad and Tobago | 1970 to 1979 | 1 (1, 1) |
| Trinidad and Tobago | 1975 to 1984 | 0.985 (0.855, 1.136) |
| Trinidad and Tobago | 1980 to 1989 | 0.971 (0.831, 1.133) |
| Trinidad and Tobago | 1985 to 1994 | 0.967 (0.804, 1.162) |
| Trinidad and Tobago | 1990 to 1999 | 0.96 (0.756, 1.219) |
| Trinidad and Tobago | 1995 to 2004 | 0.939 (0.658, 1.338) |
| Tunisia | 1950 to 1959 | 1.098 (0.99, 1.217) |
| Tunisia | 1955 to 1964 | 1.068 (0.994, 1.148) |
| Tunisia | 1960 to 1969 | 1.048 (0.986, 1.112) |
| Tunisia | 1965 to 1974 | 1.024 (0.971, 1.08) |
| Tunisia | 1970 to 1979 | 1 (1, 1) |
| Tunisia | 1975 to 1984 | 0.978 (0.932, 1.026) |
| Tunisia | 1980 to 1989 | 0.956 (0.908, 1.007) |
| Tunisia | 1985 to 1994 | 0.936 (0.883, 0.993) |
| Tunisia | 1990 to 1999 | 0.922 (0.857, 0.992) |
| Tunisia | 1995 to 2004 | 0.91 (0.817, 1.013) |
| Turkey | 1950 to 1959 | 1.191 (1.142, 1.241) |
| Turkey | 1955 to 1964 | 1.128 (1.095, 1.162) |
| Turkey | 1960 to 1969 | 1.07 (1.044, 1.097) |
| Turkey | 1965 to 1974 | 1.029 (1.006, 1.052) |
| Turkey | 1970 to 1979 | 1 (1, 1) |
| Turkey | 1975 to 1984 | 0.976 (0.957, 0.995) |
| Turkey | 1980 to 1989 | 0.955 (0.935, 0.976) |
| Turkey | 1985 to 1994 | 0.938 (0.916, 0.961) |
| Turkey | 1990 to 1999 | 0.926 (0.9, 0.953) |
| Turkey | 1995 to 2004 | 0.914 (0.877, 0.952) |
| Turkmenistan | 1950 to 1959 | 1.1 (0.946, 1.28) |
| Turkmenistan | 1955 to 1964 | 1.076 (0.964, 1.2) |
| Turkmenistan | 1960 to 1969 | 1.047 (0.952, 1.153) |
| Turkmenistan | 1965 to 1974 | 1.025 (0.939, 1.119) |
| Turkmenistan | 1970 to 1979 | 1 (1, 1) |
| Turkmenistan | 1975 to 1984 | 0.979 (0.903, 1.061) |
| Turkmenistan | 1980 to 1989 | 0.961 (0.881, 1.048) |
| Turkmenistan | 1985 to 1994 | 0.943 (0.856, 1.038) |
| Turkmenistan | 1990 to 1999 | 0.929 (0.825, 1.046) |
| Turkmenistan | 1995 to 2004 | 0.92 (0.775, 1.092) |
| Tuvalu | 1950 to 1959 | 1.077 (0.069, 16.872) |
| Tuvalu | 1955 to 1964 | 1.092 (0.104, 11.457) |
| Tuvalu | 1960 to 1969 | 1.073 (0.119, 9.68) |
| Tuvalu | 1965 to 1974 | 1.045 (0.131, 8.336) |
| Tuvalu | 1970 to 1979 | 1 (1, 1) |
| Tuvalu | 1975 to 1984 | 0.952 (0.133, 6.808) |
| Tuvalu | 1980 to 1989 | 0.943 (0.1, 8.893) |
| Tuvalu | 1985 to 1994 | 0.89 (0.071, 11.114) |
| Tuvalu | 1990 to 1999 | 0.866 (0.044, 16.983) |
| Tuvalu | 1995 to 2004 | 0.841 (0.013, 54.379) |
| Uganda | 1950 to 1959 | 0.992 (0.908, 1.084) |
| Uganda | 1955 to 1964 | 1.01 (0.949, 1.076) |
| Uganda | 1960 to 1969 | 1.024 (0.971, 1.08) |
| Uganda | 1965 to 1974 | 1.023 (0.976, 1.073) |
| Uganda | 1970 to 1979 | 1 (1, 1) |
| Uganda | 1975 to 1984 | 0.966 (0.926, 1.008) |
| Uganda | 1980 to 1989 | 0.926 (0.884, 0.97) |
| Uganda | 1985 to 1994 | 0.89 (0.844, 0.937) |
| Uganda | 1990 to 1999 | 0.863 (0.811, 0.918) |
| Uganda | 1995 to 2004 | 0.835 (0.767, 0.909) |
| Ukraine | 1950 to 1959 | 1.032 (0.984, 1.082) |
| Ukraine | 1955 to 1964 | 1.027 (0.989, 1.065) |
| Ukraine | 1960 to 1969 | 1.017 (0.983, 1.053) |
| Ukraine | 1965 to 1974 | 1.009 (0.977, 1.042) |
| Ukraine | 1970 to 1979 | 1 (1, 1) |
| Ukraine | 1975 to 1984 | 0.991 (0.962, 1.021) |
| Ukraine | 1980 to 1989 | 0.984 (0.953, 1.017) |
| Ukraine | 1985 to 1994 | 0.977 (0.939, 1.015) |
| Ukraine | 1990 to 1999 | 0.97 (0.921, 1.022) |
| Ukraine | 1995 to 2004 | 0.97 (0.897, 1.049) |
| United Arab Emirates | 1950 to 1959 | 1.156 (1.016, 1.315) |
| United Arab Emirates | 1955 to 1964 | 1.118 (1.018, 1.227) |
| United Arab Emirates | 1960 to 1969 | 1.071 (0.995, 1.153) |
| United Arab Emirates | 1965 to 1974 | 1.035 (0.973, 1.1) |
| United Arab Emirates | 1970 to 1979 | 1 (1, 1) |
| United Arab Emirates | 1975 to 1984 | 0.978 (0.932, 1.027) |
| United Arab Emirates | 1980 to 1989 | 0.945 (0.894, 0.999) |
| United Arab Emirates | 1985 to 1994 | 0.919 (0.852, 0.991) |
| United Arab Emirates | 1990 to 1999 | 0.905 (0.806, 1.016) |
| United Arab Emirates | 1995 to 2004 | 0.877 (0.749, 1.027) |
| United Kingdom | 1950 to 1959 | 1.018 (0.973, 1.065) |
| United Kingdom | 1955 to 1964 | 1.013 (0.981, 1.047) |
| United Kingdom | 1960 to 1969 | 1.009 (0.982, 1.036) |
| United Kingdom | 1965 to 1974 | 1.004 (0.979, 1.029) |
| United Kingdom | 1970 to 1979 | 1 (1, 1) |
| United Kingdom | 1975 to 1984 | 0.996 (0.973, 1.02) |
| United Kingdom | 1980 to 1989 | 0.994 (0.969, 1.019) |
| United Kingdom | 1985 to 1994 | 0.992 (0.964, 1.02) |
| United Kingdom | 1990 to 1999 | 0.989 (0.956, 1.024) |
| United Kingdom | 1995 to 2004 | 0.983 (0.935, 1.034) |
| United Republic of Tanzania | 1950 to 1959 | 1.13 (1.077, 1.184) |
| United Republic of Tanzania | 1955 to 1964 | 1.094 (1.056, 1.133) |
| United Republic of Tanzania | 1960 to 1969 | 1.062 (1.029, 1.095) |
| United Republic of Tanzania | 1965 to 1974 | 1.032 (1.004, 1.061) |
| United Republic of Tanzania | 1970 to 1979 | 1 (1, 1) |
| United Republic of Tanzania | 1975 to 1984 | 0.964 (0.94, 0.989) |
| United Republic of Tanzania | 1980 to 1989 | 0.918 (0.892, 0.945) |
| United Republic of Tanzania | 1985 to 1994 | 0.868 (0.839, 0.898) |
| United Republic of Tanzania | 1990 to 1999 | 0.824 (0.79, 0.859) |
| United Republic of Tanzania | 1995 to 2004 | 0.775 (0.731, 0.822) |
| United States of America | 1950 to 1959 | 1.049 (1.005, 1.095) |
| United States of America | 1955 to 1964 | 1.023 (0.992, 1.055) |
| United States of America | 1960 to 1969 | 1.007 (0.981, 1.034) |
| United States of America | 1965 to 1974 | 1 (0.976, 1.025) |
| United States of America | 1970 to 1979 | 1 (1, 1) |
| United States of America | 1975 to 1984 | 1.011 (0.988, 1.034) |
| United States of America | 1980 to 1989 | 1.016 (0.992, 1.04) |
| United States of America | 1985 to 1994 | 1.022 (0.995, 1.049) |
| United States of America | 1990 to 1999 | 1.016 (0.984, 1.048) |
| United States of America | 1995 to 2004 | 0.999 (0.955, 1.045) |
| United States Virgin Islands | 1950 to 1959 | 1.11 (0.432, 2.848) |
| United States Virgin Islands | 1955 to 1964 | 1.1 (0.529, 2.285) |
| United States Virgin Islands | 1960 to 1969 | 1.07 (0.559, 2.048) |
| United States Virgin Islands | 1965 to 1974 | 0.918 (0.495, 1.703) |
| United States Virgin Islands | 1970 to 1979 | 1 (1, 1) |
| United States Virgin Islands | 1975 to 1984 | 1.002 (0.561, 1.791) |
| United States Virgin Islands | 1980 to 1989 | 0.949 (0.496, 1.815) |
| United States Virgin Islands | 1985 to 1994 | 0.864 (0.401, 1.862) |
| United States Virgin Islands | 1990 to 1999 | 0.844 (0.326, 2.184) |
| United States Virgin Islands | 1995 to 2004 | 0.93 (0.245, 3.534) |
| Uruguay | 1950 to 1959 | 1.043 (0.851, 1.277) |
| Uruguay | 1955 to 1964 | 1.031 (0.89, 1.194) |
| Uruguay | 1960 to 1969 | 1.018 (0.899, 1.152) |
| Uruguay | 1965 to 1974 | 1.006 (0.902, 1.123) |
| Uruguay | 1970 to 1979 | 1 (1, 1) |
| Uruguay | 1975 to 1984 | 0.995 (0.9, 1.1) |
| Uruguay | 1980 to 1989 | 0.989 (0.887, 1.102) |
| Uruguay | 1985 to 1994 | 0.985 (0.874, 1.11) |
| Uruguay | 1990 to 1999 | 0.98 (0.852, 1.126) |
| Uruguay | 1995 to 2004 | 0.979 (0.804, 1.191) |
| Uzbekistan | 1950 to 1959 | 1.092 (1.022, 1.167) |
| Uzbekistan | 1955 to 1964 | 1.067 (1.017, 1.12) |
| Uzbekistan | 1960 to 1969 | 1.044 (1.001, 1.089) |
| Uzbekistan | 1965 to 1974 | 1.021 (0.983, 1.062) |
| Uzbekistan | 1970 to 1979 | 1 (1, 1) |
| Uzbekistan | 1975 to 1984 | 0.979 (0.946, 1.014) |
| Uzbekistan | 1980 to 1989 | 0.96 (0.925, 0.997) |
| Uzbekistan | 1985 to 1994 | 0.943 (0.905, 0.983) |
| Uzbekistan | 1990 to 1999 | 0.931 (0.885, 0.98) |
| Uzbekistan | 1995 to 2004 | 0.92 (0.856, 0.989) |
| Vanuatu | 1950 to 1959 | 1.236 (0.496, 3.076) |
| Vanuatu | 1955 to 1964 | 1.08 (0.533, 2.188) |
| Vanuatu | 1960 to 1969 | 1.045 (0.566, 1.926) |
| Vanuatu | 1965 to 1974 | 1.006 (0.582, 1.741) |
| Vanuatu | 1970 to 1979 | 1 (1, 1) |
| Vanuatu | 1975 to 1984 | 1.007 (0.62, 1.635) |
| Vanuatu | 1980 to 1989 | 0.957 (0.562, 1.63) |
| Vanuatu | 1985 to 1994 | 0.949 (0.526, 1.713) |
| Vanuatu | 1990 to 1999 | 0.892 (0.441, 1.806) |
| Vanuatu | 1995 to 2004 | 0.88 (0.331, 2.341) |
| Venezuela (Bolivarian Republic of) | 1950 to 1959 | 1.05 (0.99, 1.113) |
| Venezuela (Bolivarian Republic of) | 1955 to 1964 | 1.034 (0.991, 1.078) |
| Venezuela (Bolivarian Republic of) | 1960 to 1969 | 1.026 (0.99, 1.064) |
| Venezuela (Bolivarian Republic of) | 1965 to 1974 | 1.016 (0.984, 1.048) |
| Venezuela (Bolivarian Republic of) | 1970 to 1979 | 1 (1, 1) |
| Venezuela (Bolivarian Republic of) | 1975 to 1984 | 0.983 (0.955, 1.011) |
| Venezuela (Bolivarian Republic of) | 1980 to 1989 | 0.968 (0.938, 0.999) |
| Venezuela (Bolivarian Republic of) | 1985 to 1994 | 0.955 (0.921, 0.989) |
| Venezuela (Bolivarian Republic of) | 1990 to 1999 | 0.939 (0.898, 0.982) |
| Venezuela (Bolivarian Republic of) | 1995 to 2004 | 0.923 (0.864, 0.985) |
| Viet Nam | 1950 to 1959 | 1.288 (1.215, 1.366) |
| Viet Nam | 1955 to 1964 | 1.199 (1.149, 1.252) |
| Viet Nam | 1960 to 1969 | 1.112 (1.071, 1.154) |
| Viet Nam | 1965 to 1974 | 1.047 (1.013, 1.083) |
| Viet Nam | 1970 to 1979 | 1 (1, 1) |
| Viet Nam | 1975 to 1984 | 0.975 (0.945, 1.005) |
| Viet Nam | 1980 to 1989 | 0.95 (0.919, 0.983) |
| Viet Nam | 1985 to 1994 | 0.944 (0.909, 0.979) |
| Viet Nam | 1990 to 1999 | 0.945 (0.903, 0.989) |
| Viet Nam | 1995 to 2004 | 0.957 (0.896, 1.023) |
| Yemen | 1950 to 1959 | 1.039 (0.956, 1.13) |
| Yemen | 1955 to 1964 | 1.048 (0.989, 1.111) |
| Yemen | 1960 to 1969 | 1.047 (0.999, 1.098) |
| Yemen | 1965 to 1974 | 1.028 (0.987, 1.071) |
| Yemen | 1970 to 1979 | 1 (1, 1) |
| Yemen | 1975 to 1984 | 0.969 (0.937, 1.002) |
| Yemen | 1980 to 1989 | 0.941 (0.909, 0.975) |
| Yemen | 1985 to 1994 | 0.916 (0.881, 0.952) |
| Yemen | 1990 to 1999 | 0.891 (0.851, 0.932) |
| Yemen | 1995 to 2004 | 0.868 (0.817, 0.922) |
| Zambia | 1950 to 1959 | 1.202 (1.072, 1.348) |
| Zambia | 1955 to 1964 | 1.152 (1.058, 1.254) |
| Zambia | 1960 to 1969 | 1.105 (1.027, 1.19) |
| Zambia | 1965 to 1974 | 1.054 (0.987, 1.125) |
| Zambia | 1970 to 1979 | 1 (1, 1) |
| Zambia | 1975 to 1984 | 0.952 (0.898, 1.01) |
| Zambia | 1980 to 1989 | 0.9 (0.843, 0.961) |
| Zambia | 1985 to 1994 | 0.849 (0.788, 0.916) |
| Zambia | 1990 to 1999 | 0.814 (0.743, 0.893) |
| Zambia | 1995 to 2004 | 0.787 (0.692, 0.894) |
| Zimbabwe | 1950 to 1959 | 1.024 (0.946, 1.109) |
| Zimbabwe | 1955 to 1964 | 1.018 (0.958, 1.082) |
| Zimbabwe | 1960 to 1969 | 1.011 (0.958, 1.067) |
| Zimbabwe | 1965 to 1974 | 1.006 (0.958, 1.056) |
| Zimbabwe | 1970 to 1979 | 1 (1, 1) |
| Zimbabwe | 1975 to 1984 | 0.995 (0.953, 1.039) |
| Zimbabwe | 1980 to 1989 | 0.986 (0.939, 1.036) |
| Zimbabwe | 1985 to 1994 | 0.977 (0.922, 1.035) |
| Zimbabwe | 1990 to 1999 | 0.971 (0.905, 1.042) |
| Zimbabwe | 1995 to 2004 | 0.967 (0.876, 1.067) |

**Abbreviations:** DALYs, Disability adjusted life years.
